# Multimodal Molecular Motion in the Rotaxanes and Catenanes Incorporating Flexible Calix[*n*]phyrin Stations

**DOI:** 10.1002/anie.202413579

**Published:** 2024-10-23

**Authors:** Rafał A. Grzelczak, Tymoteusz Basak, Bartosz Trzaskowski, Vasyl Kinzhybalo, Bartosz Szyszko

**Affiliations:** ^1^ Faculty of Chemistry University of Wrocław 14 F. Joliot-Curie St. 50-387 Wrocław Poland; ^2^ Centre of New Technologies University of Warsaw 2c Banach St. 02-097 Warsaw Poland; ^3^ Institute of Low Temperature and Structure Research Polish Academy of Sciences 2 Okólna St. 50-422 Wrocław Poland

**Keywords:** supramolecular chemistry, mechanically interlocked molecules, catenanes, rotaxanes, porphyrinoids

## Abstract

The synthesis of [2]rotaxanes stoppered with one or two dipyrromethane groups has opened a route for the construction of mechanically interlocked molecules incorporating various porphyrinoid stations. The exploitation of those precursors allowed the creation of [3]rotaxanes and [2]catenanes based on the calix[4]phyrin motif, presenting intriguing molecular dynamics. The intrinsic flexibility of the porphyrinoid allowed the introduction of a new type of molecular motion within the rotaxanes, termed fluttering. The latter involved a bending of the axle, interconverting two angular‐shaped stereoisomers of the rotaxane through a planarised transition state. Simple chemical transformations, i.e. methylation and (de)protonation of the [3]rotaxane and [2]catenane allowed controllable transformations within the conformationally flexible calix[4]phyrin‐incorporated mechanically interlocked porphyrinoids.

## Graphical abstract

The synthesis of [2]rotaxanes stoppered with dipyrromethane has opened a route to rotaxanes and catenanes incorporating calix[4]phyrin units. A new type of molecular motion, termed fluttering, has been observed and simple chemical transformations such as methylation and (de)protonation of the [3]rotaxane and [2]catenane enables controllable transformations within the conformationally flexible mechanically interlocked compounds.

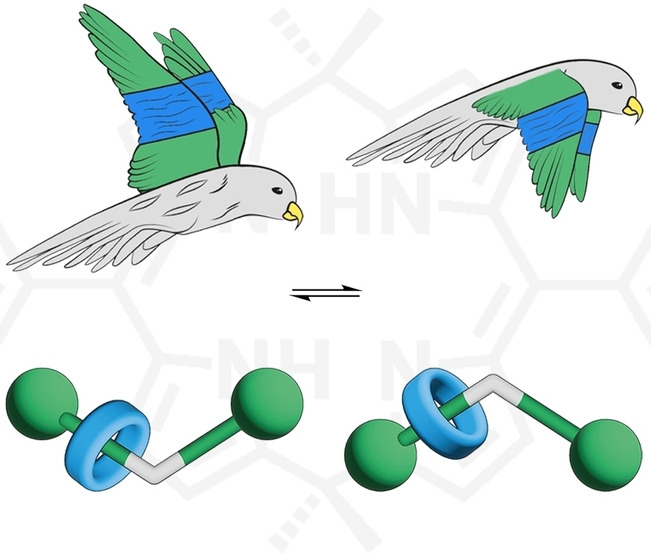

## Introduction

Catenanes and rotaxanes constitute the archetypical examples of Mechanically Interlocked Molecules (MIMs).[^1^, ^2^] The continuous development of molecular systems incorporating mechanical bond has allowed for their use in various areas of chemical sciences, such as molecular recognition and sensing,[^3^, ^4^, ^5^] supramolecular catalysis,[^6^, ^7^, ^8^] stimuli‐responsive polymers[^9^, ^10^] or biomedicine.[^11^, ^12^, ^13^] Potentially, the most prominent application of molecular links and rotaxanes is their use for constructing artificial synthetic machines.[^2^, ^14^, ^15^] The latter comprises a discrete number of components which can perform mechanical‐like movements in response to external stimuli.[^16^, ^17^]

**Figure 1:**
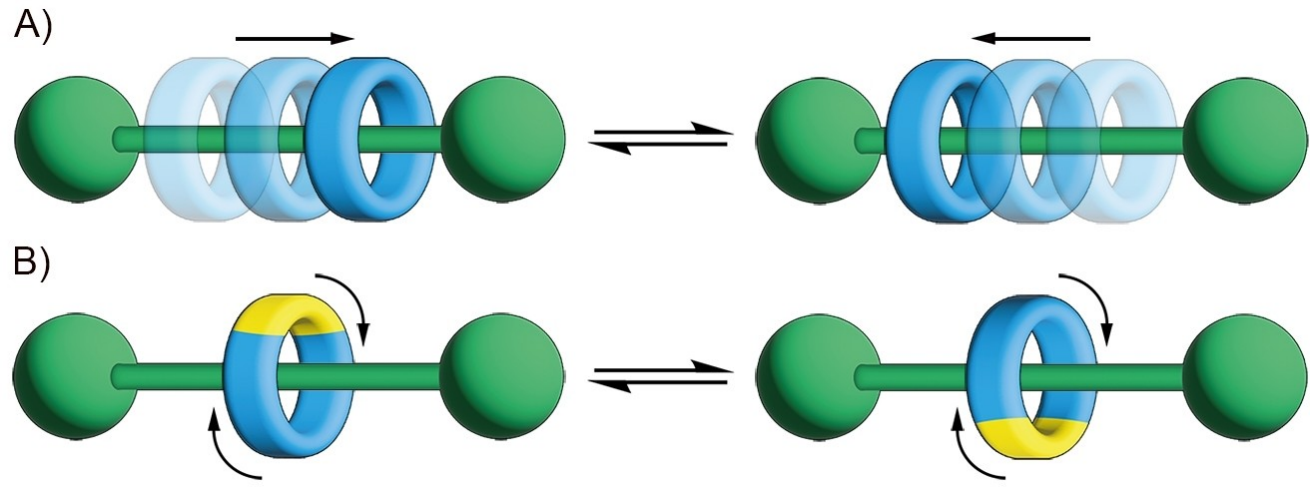
The fundamental types of molecular motion in [2]rotaxanes: A) shuttling and B) pirouetting.

The crucial feature of rotaxanes, which allows for their exploitation in the construction of molecular machinery, is a high degree of molecular motion, which can be realized through two fundamental types of movement (Figure 1).[^17^, ^18^, ^19^] The sliding of the macrocyclic component along the linear axle is termed shuttling.^[20]^ The shift of the macrocycle is non‐directional unless a specific axle/ring design is applied or external stimuli enforce the movement‘s directionality. Rotation of the macrocyclic ring around the axle is referred to as pirouetting, and a similar process could also be identified in molecular links wherein one macrocyclic component rotates around the other.[^21^, ^22^] Introducing directionality into these fundamental types of conformational rearrangements remains one of the most important aspects of modern supramolecular chemistry.^[16]^

Porphyrins have been particularly popular among various macrocyclic motifs exploited for constructing MIMs due to their energy and electron transfer properties.[^23^, ^24^] The versatile coordination chemistry of metalloporphyrins allowed their application in molecular machines where the motion could be controlled through the ligand exchange mechanism or anion coordination.[^23^, ^25^, ^26^, ^27^] They were also demonstrated to act as catalytic platforms for the MIMs synthesis[^28^, ^29^] and form interlocked anion sensors.[^24^, ^27^] Despite considerable interest in the rotaxanes and catenanes incorporating porphyrin macrocycle, the number of MIMs based on other porphyrinoids remains limited.[^30^, ^31^, ^32^, ^33^] Developing functional molecular machines incorporating porphyrinoid motifs requires finding suitable macrocycles with intrinsic, well‐understood, and controllable intramolecular dynamics. In search of macrocyclic components to be used as structural elements in molecular machines, we have realised that those criteria are met by a specific group of tetrapyrroles, namely calix[4]phyrins.[^34^, ^35^, ^36^, ^37^]

In this work, the synthesis of dipyrromethane‐stoppered [2]rotaxanes, constituting principal building blocks of a series of mechanically interlocked molecules containing the calix[n]phyrin moiety, is described. The potential of porphyrinoid‐incorporating architectures in constructing stimuli‐responsive dynamic systems presenting multiple modes of molecular motion is demonstrated by exploiting facile acid‐base chemistry, which turns the dynamics in various regions of the interlocked architecture on and off.

## Results and Discussion

The construction of mechanically interlocked molecules incorporating tetrapyrroles, e.g., porphyrin or corrole,^[38]^ typically required the formation of a *meso*‐functionalised macrocycle that acts as a substrate for more complex architecture.[^25^, ^39^, ^40^] However, this approach is often problematic, primarily when the synthetic pathway aiming to create a MIM relies on the reactions that require a catalyst based on the transition metal. Under such circumstances, the porphyrinoid, typically constituting an excellent coordination motif, acts as a competing ligand toward the reagents necessary to create new bonds, and the additional synthetic steps, i.e. metalation of the macrocycle and the subsequent demetalation, are required. In many cases, the removal of the metal cation from the porphyrinoid cavity is challenging and sometimes even impossible. These limitations often preclude the use of the prefunctionalized porphyrinoids in the synthetic pathways based on the active template (AT) approach, constituting one of the most significant advances in the synthesis of interlocked molecules in recent years.[^41^, ^42^, ^43^] The copper(I)‐catalysed alkynes to azide cycloaddition (CuAAC) is the most commonly used reaction belonging to the AT repertoire, especially for the synthesis of rotaxanes.^[44]^ In this approach, copper(I) acts not only as a template, geometrically organising the components, but it also creates a catalytically active moiety, which takes part in the formation of the covalent bond.

Our approach relies on a fundamentally different principle: the mechanical bond is introduced early in the synthetic pathway, preventing the competition of copper(I) for several coordination motifs. To realise such a synthetic scenario, the precursor [2]rotaxanes, which encompasses dipyrromethane unit(s), were designed. Their exploitation in the [2+2] macrocyclisation carried out in the presence of carbonyl compounds opens a route for creating a variety of porphyrinoid‐incorporating MIMs with tailored functions.

**Scheme 1:**
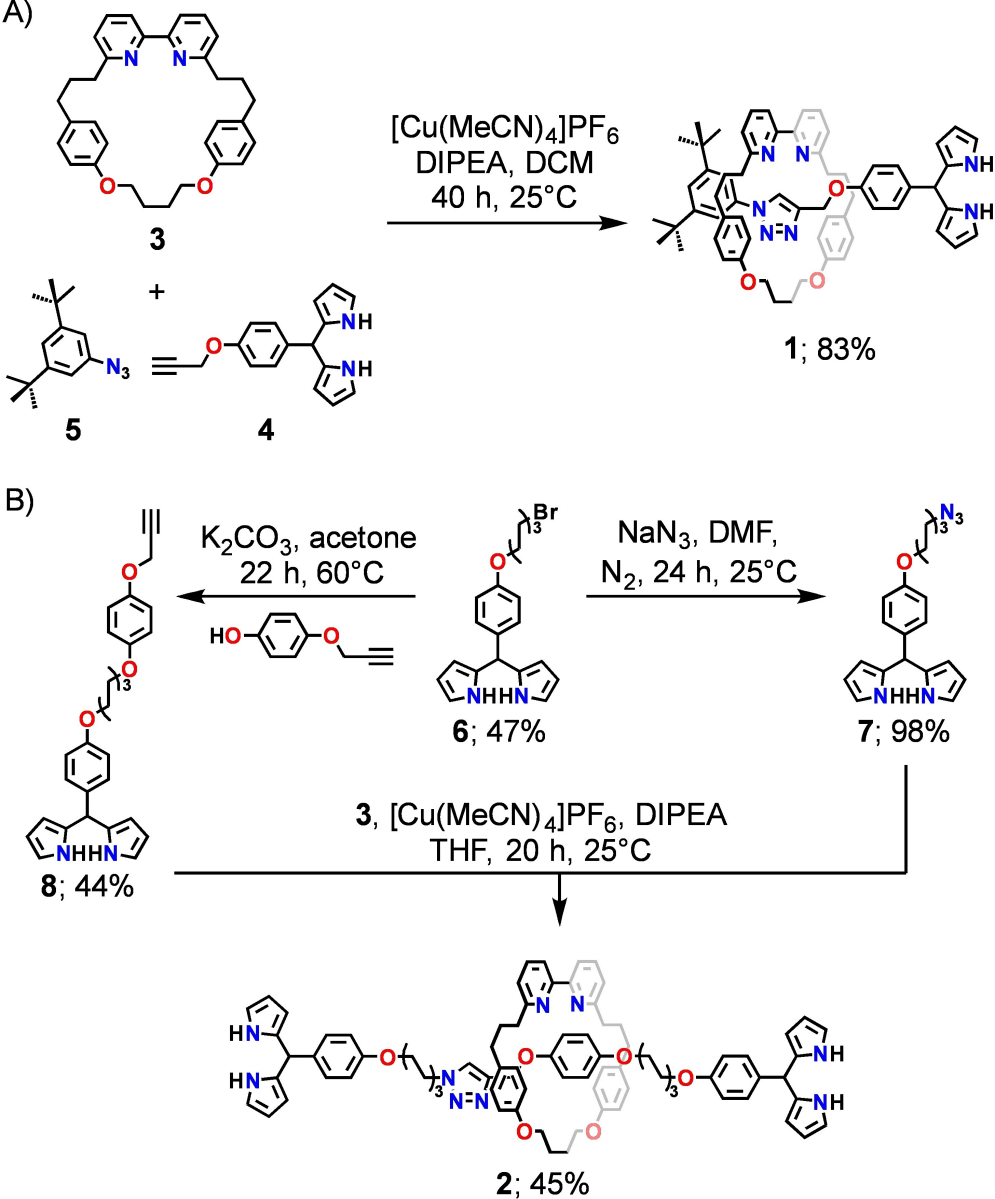
The synthesis of **1** and **2**.

To illustrate the versatility of the proposed methodology, the two fundamental precursor rotaxanes were targeted, incorporating an axle terminated with a single **1** or two dipyrromethane stoppers **2** (Scheme 1). It was anticipated that **1** may be a suitable synthon for the construction of higher‐order rotaxanes, whereas **2** will act as a reagent for the catenanes formation.

The mechanically interlocked dipyrromethane **1** was formed using the CuAAC active template strategy, exploiting macrocycle **3** developed by Goldup.^[45]^ The required alkyne **4** could be easily obtained in the two‐step procedure (see Supporting Information). The reaction of **3**, **4**, and 1‐azido‐3,5‐di‐*tert*‐butylbenzene **5** was carried out in the presence of tetrakis(acetonitrile)copper(I) hexafluorophosphate and N,N‐diisopropylethylamine (DIPEA) in DCM. Upon post‐synthetic workup and chromatographic separation, the [2]rotaxane **1** was isolated in a high 83 % yield, indicating that neither dipyrromethane‐incorporating substrate **4** nor **1** significantly competed with macrocyclic bipyridine ligand **3**, leading to a good quantity of the target [2]rotaxane.

The synthesis of **2** required the formation of azide‐ **7** and alkyne‐incorporating dipyrromethane **8**. The former was synthesised from *p*‐hydroxybenzaldehyde, which, once reacted with 1,6‐dibromohexane, formed a bromoderivative (see Supporting Information). The trifluoroacetic acid (TFAH)‐catalysed condensation of the latter with excess pyrrole provided dipyrromethane **6**, which was transformed into **7** in reaction with sodium azide in DMF. The substitution of **6** with 4‐(prop‐2‐yn‐1‐yloxy)phenol under basic conditions provided **8** in 44 % yield. Once **7** and **8** were reacted following the developed CuAAC conditions, the bis‐dipyrromethane stoppered [2]rotaxane **2** could be isolated in a 45 % yield.

The identity of [2]rotaxanes was corroborated by the combination of the ^1^H, ^13^C and 2D NMR spectroscopy and high‐resolution mass spectrometry (Figures S22–37, Supporting Information). The ^1^H NMR spectrum of **1** in [D]chloroform at 300 K demonstrated features expected from a [2]rotaxane incorporating an axle terminated with a single dipyrromethane moiety (Figure S22, Supporting Information). The three α and β pyrrolic protons resonated in the 6.65–5.84 ppm range. The broad NH signal at 7.87 ppm and *meso*‐H resonance at 5.29 ppm indicated that under the applied reaction conditions, **1** did not oxidise to the corresponding dipyrrin. The chemical shift of the triazole CH resonance equal to 9.86 ppm suggested its involvement in the hydrogen bonding (HB) with the bipyridine unit of the macrocycle **3**.^[46]^ In the case of **2**, the triazole resonance was identified at 8.02 ppm (Figure S31, Supporting Information).

Density functional theory (DFT) and tight‐binding calculations, including conformational search followed by geometry optimisation, were carried out to get an insight into the structural features of **1** and **2** (Figure S171–176, Supporting Information). Both lowest‐energy structures have revealed the co‐conformation of a rotaxane wherein the triazole ring of the axle is incorporated within the cavity of the macrocyclic component **3** and stabilised through the CH⋅⋅⋅N hydrogen bonding, as inferred from the ^1^H NMR spectroscopy.

**Scheme 2:**
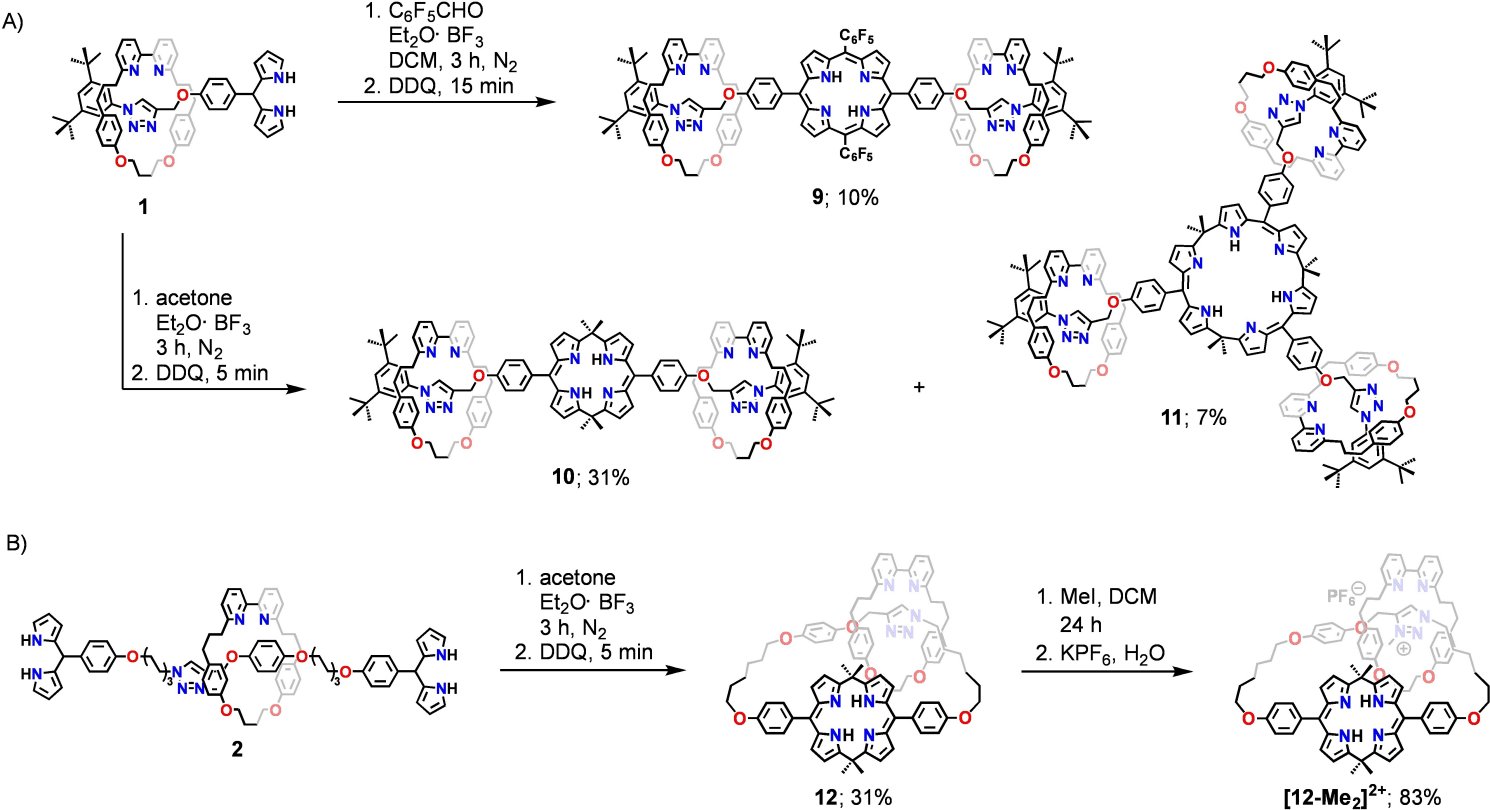
Synthesis and reactivity of **9**–**12**.

Mechanically interlocked dipyrromethane **1** was subjected to condensation aiming for the porphyrin‐incorporating [3]rotaxane **9** not only to evaluate its value in reactions targeting various porphyrinoids but also to gain access to a valuable reference compound for structural analyses (Scheme 2). The reaction of **1** with a stoichiometric amount of pentafluorobenzaldehyde in DCM was carried out in the presence of boron trifluoride diethyl etherate. The oxidation of intermediate porphyrinogens with 2,3‐dichloro‐5,6‐dicyanobenzoquinone (DDQ) and chromatographic separation allowed for the isolation of **9** in 10 % yield. The macrocycle **3** was detected in the product mixture through NMR spectroscopy, consistent with the observed reactivity of **1** under acidic conditions and in agreement with earlier reports on the scrambling of dipyrromethanes (Figures S30, Supporting Information).^[47]^

The elemental composition of **9** established through ESI MS (electrospray ionisation mass spectrometry) fitted the proposed product structure (Figure S48, Supporting Information). The ^1^H NMR spectrum of **9** in [D]chloroform at 300 K confirmed the formation of a [3]rotaxane incorporating porphyrin macrocycle within the axle (Figure S40, Supporting Information). Two signals at 8.88 and 8.77 ppm corresponding to deshielded β‐H pyrrolic positions of the aromatic macrocycle were accompanied by the NH resonance at −2.83 ppm. The triazole CH gave rise to a singlet at 9.86 ppm, indicating the CH⋅⋅⋅N HB with the macrocyclic bipyridine.

**Figure 2:**
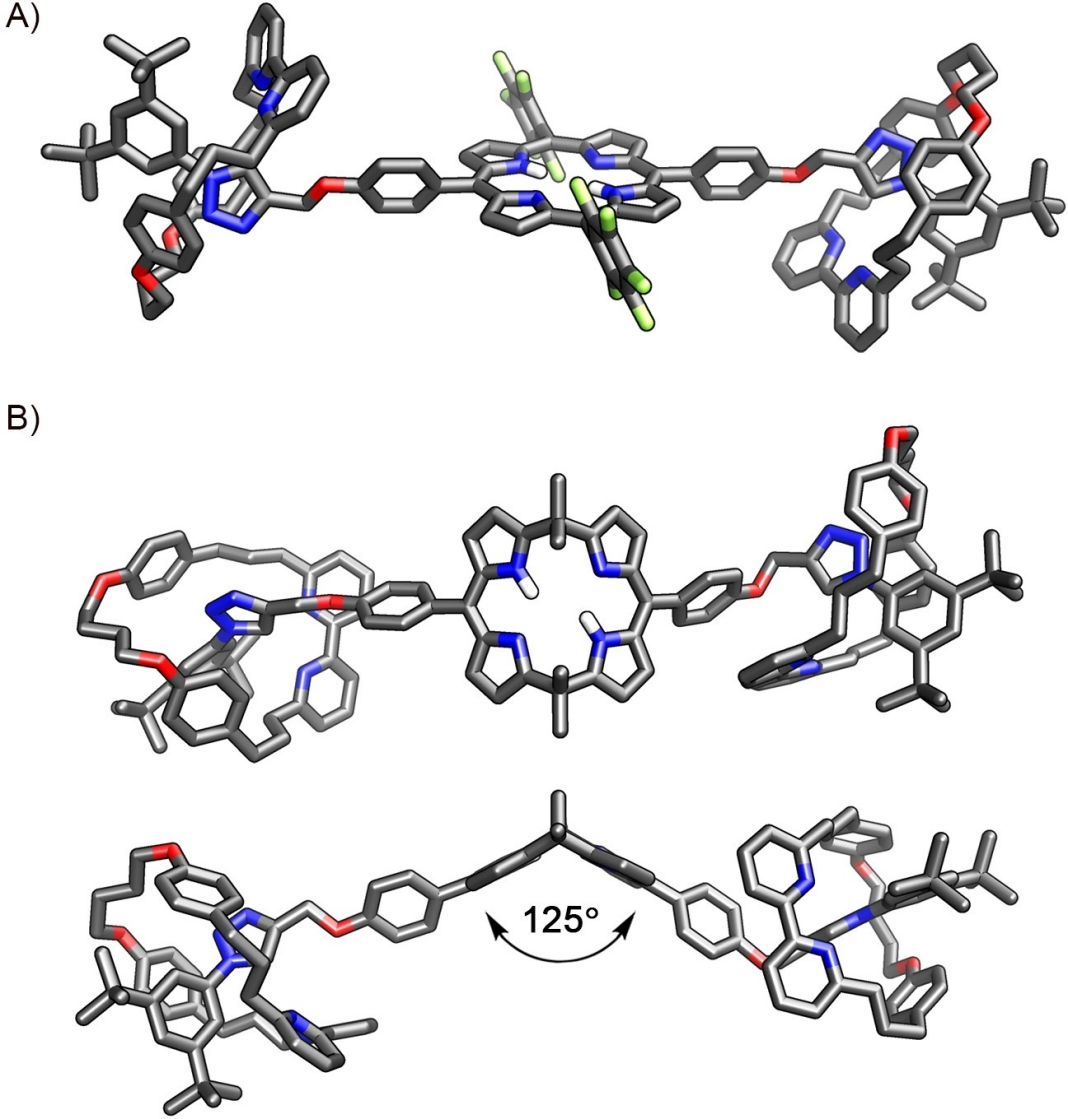
A) The X‐ray molecular structure of **9**, and B) the DFT‐optimized (B3LYP‐D3, double‐zeta 6‐31G**) model of **10**. Only selected protons are shown for clarity.^[48]^

The molecular structure of **9** was confirmed by X‐ray crystallography (Figure 2A).^[48]^ Single crystals were obtained by slow evaporation of the solution of **9** in dichloromethane/*n*‐hexane. [3]Rotaxane crystallised in the triclinic system in the *P* $\bar{1}$ group. The molecule comprised two macrocyclic bipyridine **3** components threaded onto a linear axle incorporating a planar porphyrin ring. The macrocycles were located on both sides of the porphyrin and interacted with triazole rings through the CH⋅⋅⋅N hydrogen bond (CH⋅⋅⋅N=2.65, 2.32 Å).

Once the initial experimentation proved the value of **1** in the reactions aiming for porphyrinoids, the calix[4]phyrin‐incorporating [3]rotaxane **10** was targeted (Scheme 2). The calix[4]phyrin(**1**.*1*.**1**.*1*) is a hybrid macrocycle, that constitutes a link between the calix[4]pyrrole and porphyrin.[^36^, ^37^] The connection of the two dipyrrin moieties through the *sp* ^3^ *meso*‐carbon atoms results in unusual architecture with intriguing dynamics. X‐ray studies have revealed that in the solid state, the macrocycle adopts a roof‐shaped, gable conformation.[^34^, ^49^, ^50^, ^51^, ^52^, ^53^] However, in solution, the switching between the V‐shaped stereoisomers might occur, depending on the type and pattern of the substitution.[^49^, ^54^] Thus, it was envisaged that the introduction of the calix[4]phyrin moiety into the framework of a mechanically interlocked molecule, e.g. rotaxane or catenane, could activate a new type of molecular motion.

The formation of the target calix[4]phyrin‐incorporating [3]rotaxane **10** required the condensation of **1** with acetone in the presence of boron trifluoride diethyl etherate (Scheme 2). Oxidation of the intermediates with DDQ and chromatographic separation allowed for isolating **10** in 31 % yield. Surprisingly, **10** was not the sole product of the condensation involving **1**—a careful chromatographic separation allowed for isolating [4]rotaxane **11** with a 7 % yield. The latter formed through the condensation of three molecules of **1** with acetone, resulting in the Y‐shaped rotaxane incorporating the calix[6]phyrin(**1**.*1*.**1**.*1*.**1**.*1*) moiety.[^34^, ^35^, ^50^] The ^1^H NMR spectrum of **11** was very similar to **10**, and the identification of **11** was based on the high effective symmetry observed in the NMR spectroscopy and utilising ESI MS (Figure S59, Supporting Information). The signal corresponding to the molecular ion [M+H]^+^ was observed at *m/z*=3073.7550 and fit well with the isotopic pattern simulated for C_201_H_220_N_21_O_9_^+^ (Figure S65, Supporting Information).

The ESI MS analysis and ^1^H NMR spectrum of **10** confirmed the formation of [3]rotaxane incorporating two macrocyclic bipyridines threaded onto an axle with a central calix[4]phyrin ring (Figure S49, S56, Supporting Information). The most down‐field shifted signal at 14.15 ppm corresponded to the NH proton, indicating the intramolecular NH⋅⋅⋅N hydrogen bonding within the porphyrinoid cavity. The triazole singlet at 9.71 ppm suggested, similarly to **1** and **9**, the involvement of the heterocycle in hydrogen bonding.

To gain insight into the structural features of calix[4]phyrin‐incorporating [3]rotaxane **10**, geometry optimisation was performed at the DFT (B3LYP‐D3, double‐zeta 6‐31G**) level of theory. The optimized model of **10** revealed structural features strikingly different from those detected for the porphyrin‐based **9** (Figure 2B). The [3]rotaxane was angular shaped, resulting from a specific, roof‐like conformation of the calix[4]phyrin macrocycle. The dihedral angle between the two dipyrrin planes was equal to ca. 125°. Two arms of the calix[4]phyrin macrocycle contained a macrocyclic bipyridine component located in close proximity to the triazole. The specific co‐conformation was stabilised through the CH⋅⋅⋅N(bpy) HB.

**Figure 3:**
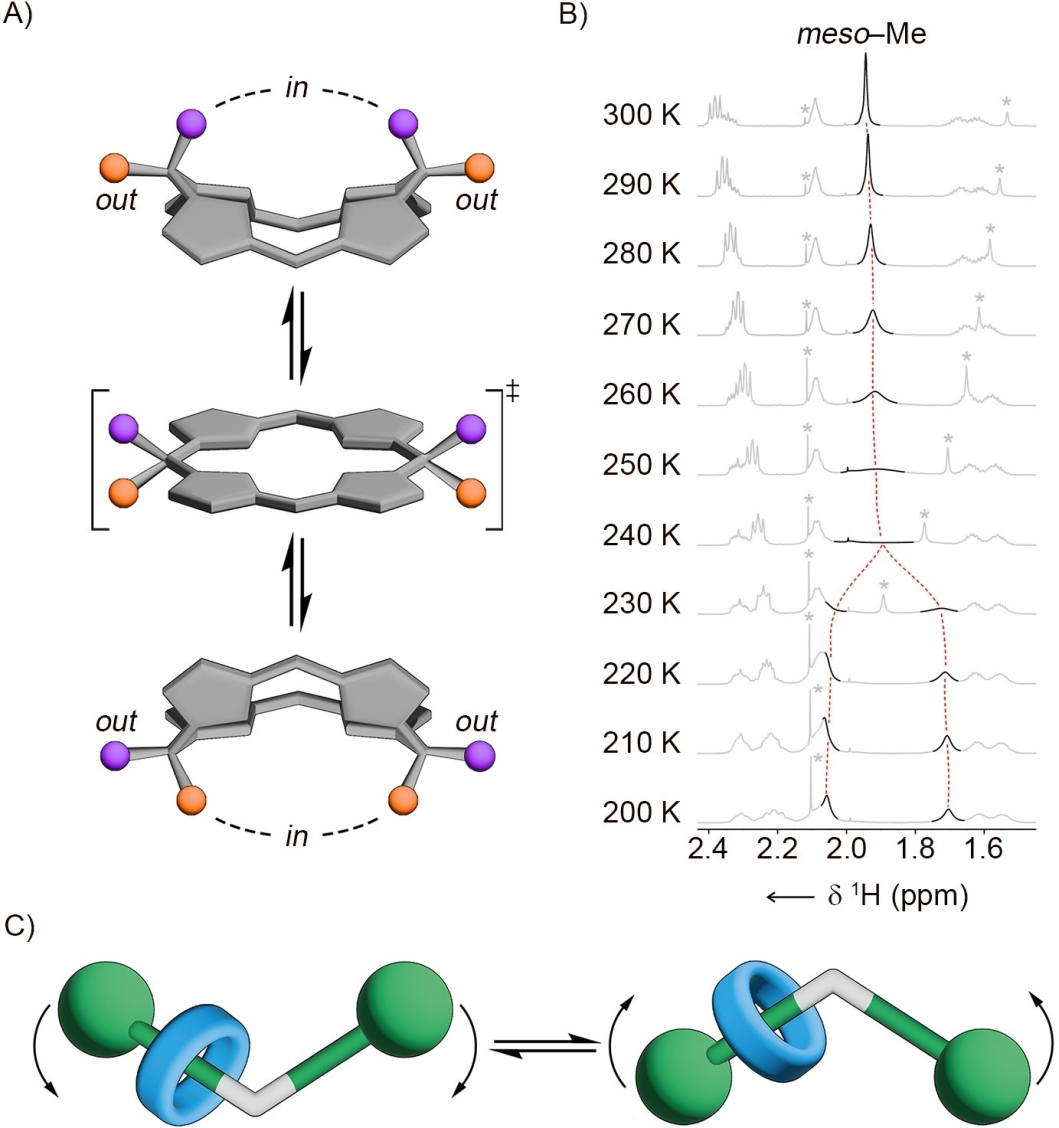
A) The conformational transformations of the calix[4]phyrin macrocycle with methyl groups represented by purple and orange spheres. B) The aliphatic region of the ^1^H NMR spectra of **10** recorded in the 300–200 K range ([D_2_]DCM, 600 MHz). C) Illustration of the fluttering motion in [2]rotaxane incorporating calix[4]phyrin.

Assuming a stable V‐shaped conformation of [3]rotaxane, the ^1^H NMR spectrum of **10** should present two separate singlets corresponding to *in*‐ and *out*‐oriented *meso*‐methyl substituents in regard to the cavity of the calix[4]phyrin moiety (Figure 3A). However, only a single *meso*‐CH_3_ resonance at 1.93 ppm was identified in the spectrum recorded at 300 K in [D_2_]DCM. Thus, ^1^H NMR spectra were recorded in the 300–180 K range to assess the dynamic process increasing the effective symmetry of **10** in the solution (Figure 3B, S57–58, Supporting Information). Upon lowering the temperature, the *meso*‐Me signal underwent continuous broadening, and eventually, it decoalesced into two resonances at 2.07 and 1.71 ppm, identified at 210 K. This behaviour can be rationalised by considering the dynamics of the calix[4]phyrin incorporated into the axle where two roof‐shaped stereoisomers exchange through a planar transition state (Figure 3A).^[49]^ This observation has profound implications, as it indicates that rotaxanes incorporating calix[4]phyrin(**1**.*1*.**1**.*1*) stations, for example, **10**, may demonstrate a multimodal molecular motion. In addition to shuttling and pirouetting, typical for rotaxanes, the toggling transformation involving the porphyrinoid incorporated into the axle enforces a new type of molecular motion, reminiscent of the flapping of butterfly wings, namely the fluttering (Figure 3C, Animation S1, Supporting Information).

The fluttering motion in **10** has been addressed by means of computational methods. First, the transition barrier for the model calix[4]phyrin(**1**.*1*.**1**.*1*) was calculated at 5.6 kcal/mol at the tight‐binding level of theory, and 5.5 kcal/mol at the DFT level, showing the very good accuracy of the tight‐binding growing strings approach (Figure S179, Supporting Information). These results, as well as the DFT value of the activation‐free energy of 5.5 kcal/mol, were also in agreement with previous estimates of the activation free energies and transition barriers for calix[4]phyrins.^[54]^ A similar tight‐binding calculation for **10** gave the transition barrier of ΔG^≠^=12.8 kcal/mol, indicating that the fluttering motion is not only accessible at room temperature but also relatively fast (Figure S182, Supporting Information). A close value of 11.5 kcal/mol was estimated by means of the variable temperature ^1^H NMR spectroscopy.

**Figure 4:**
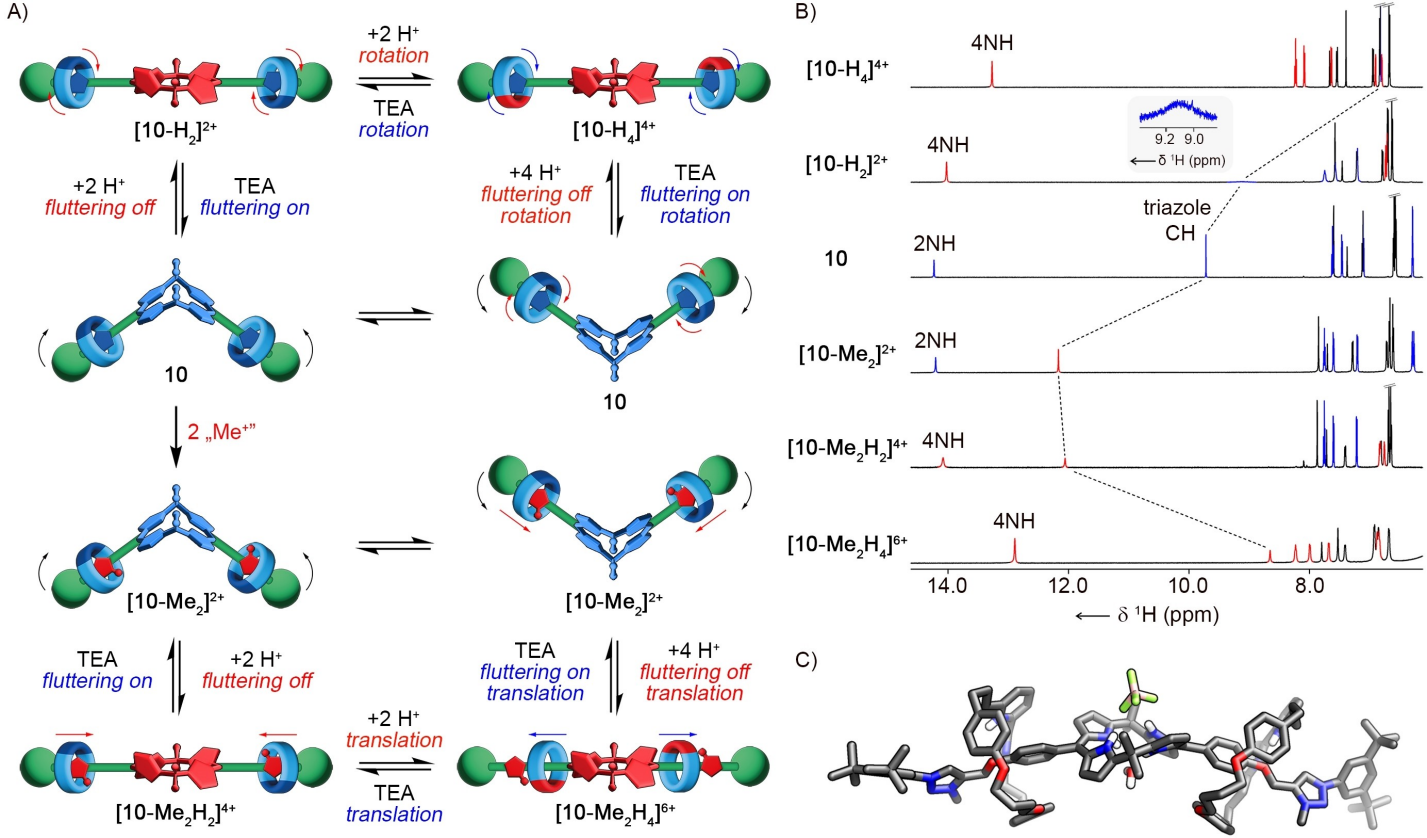
A) The reactivity of **10**. B) The ^1^H NMR spectra of **[10‐H_4_]^4+^**, **[10‐H_2_]^2+^**, **10**, **[10‐Me_2_]^2+^**, **[10‐Me_2_H_2_]^4+^**, and **[10‐Me_2_H_4_]^6+^**. C) The X‐ray molecular structure of **[10‐Me_2_H_4_][BF_4_]_6_**; only selected protons are shown for clarity.^[48]^

Three independent reactive sites in **10**, namely the bipyridine moiety, the triazole ring, and the porphyrinoid cavity, enabled the studies on the stimuli‐induced controlled molecular transformations of the [3]rotaxane (Figure 4A). It was envisaged that the conformational rearrangements of **10** could be controlled through relatively simple chemical transformations involving the alkylation of the triazole ring and acid‐base chemistry carried out in various regions of the molecule.

In the neutral form, **10** interconverts between two roof‐like conformations involved in the the fluttering motion. Titration of **10** with TFAH was monitored by ^1^H NMR spectroscopy (Figure 4B, S72, S80, Supporting Information). The protonation of **10** was a two‐step process. The continuous addition of TFAH carried out at 300 K in [D_2_]DCM initially resulted in a decrease in the intensity of the resonances of **10** and was accompanied by the rise of a new set of signals corresponding to the rotaxane dication **[10‐H_2_]^2+^**. The selective protonation of the cavity of calix[4]phyrin upon adding 2 equivalents of acid was evident as the intensity of the NH signal rose from 2H to 4H. The incorporation of four protons within the porphyrinoid cavity was expected to alter its conformation to minimise steric overcrowding. The triazole CH resonance at 9.44 ppm suggested a similar co‐conformation of the rotaxane, as detected for **10**. Upon the addition of excess TFAH, the tetracation **[10‐H_4_]^4+^** was formed. Its ^1^H NMR spectrum revealed a striking relocation of the triazole signal from 9.44 to 6.81 ppm—the result of the macrocyclic bipyridine rotation rearranging the HB network.[^55^, ^56^] This observation was confirmed by DFT‐level molecular modelling of **[10‐H_4_]^4+^** (Figure S177, Supporting Information). The optimised geometry of the tetracation revealed a twisted 1,3‐alternate conformation of calix[4]phyrin, with the pyrrole rings of each dipyrrin unit pointing in the opposite direction. Both macrocycles incorporating the monoprotonated bipyridine moiety interacted with the triazole ring through the NH⋅⋅⋅N hydrogen bond. Protonation of the calix[4]phyrin cavity resulted in the deactivation of the fluttering motion. The addition of triethylamine (TEA) to the solution of **[10‐H_4_]^4+^** resulted in deprotonation of the rotaxane retrieving **10** (Figure 4A, S80, Supporting Information).

Next, the positive charge was selectively introduced into **10** through the N‐methylation of the triazole rings (Figure 4A). The reaction of **10** with methyl iodide was carried out in DCM for 48 hours at room temperature, followed by the counterion exchange to hexafluorophosphate. The doubly N‐methylated [3]rotaxane **[10‐Me_2_][PF_6_]_2_** was isolated in a 48 % yield. The ^1^H NMR spectrum ([D_2_]DCM, 300 K) showed the N−Me resonance at 4.28 ppm (Figure S113, Supporting Information). The NHs of the calix[4]phyrin cavity gave rise to a narrow resonance at 14.18 ppm, whereas the triazole singlet was identified at 12.15 ppm (Figure 4B). The down‐field relocation of the latter, compared to **10**, indicated the increased strength of the CH⋅⋅⋅N HB between triazole in the axle and macrocyclic bipyridine. The single resonance of the *meso*‐Me groups of calix[4]phyrin at 1.94 ppm observed at 300 K split into two broad signals at 2.05 and 1.68 ppm at 200 K, indicating that the fluttering motion was not affected by the alkylation of the axle (Figure S121, Supporting Information).

Similarly to **10**, acidification of **[10‐Me_2_]^2+^** with TFAH was a two‐stage process (Figure 4B, S137, Supporting Information). Upon the addition of 2 equivalents of TFAH, the calixphyrin‐protonated **[10‐Me_2_H_2_]^4+^** was formed, showing structural features analogous to **[10‐H_2_]^2+^**. The addition of excess acid yielded **[10‐Me_2_H_4_]^6+^**, which was reflected by the significant up‐field relocation of the triazole signal to 8.57 ppm, as expected for the N,N‐dialkylated 1,2,3‐triazolium salt[^57^, ^58^] ([D_2_]DCM, 300 K). No significant HB interaction between the triazole and bipyridine resulted from the coulombic repulsion between the protonated macrocycle and the positively charged N‐methyltriazole in the axle. At the same time, the presence of positive charge in different regions of the rotaxane enforced a specific position of the protonated macrocyclic bipyridine on the rotaxane axle, separated from the N‐methyl triazolium unit on one side, and protonated calix[4]phyrin on the other. Concomitantly, protonation of the porphyrinoid turned off the fluttering motion, as inferred from the ^1^H NMR spectrum containing a single *meso*‐Me resonance in the 300–180 K temperature range (Figure S138, Supporting Information). Deprotonation of **[10‐Me_2_H_4_]^6+^** with TEA yielded **[10‐Me_2_]^2+^** (Figure 4A, S136, Supporting Information).

The X‐ray molecular structure of **[10‐Me_2_H_4_]^6+^** was consistent with the NMR spectroscopy‐based analysis providing specific geometric constraints (Figure 4C).^[48]^ Single crystals of **[10‐Me_2_H_4_][BF_4_]_6_** were obtained by slow evaporation of the solution of **[10‐Me_2_]^2+^** in the DCM/ethyl acetate mixture acidified with the excess of tetrafluoroboric acid. The salt crystallised in the monoclinic *P*2_1_/c group. The molecular structure confirmed the diprotonation of the calix[4]phyrin, which adopted a twisted 1,3‐alternate conformation with two pairs of pyrrole rings pointing up and down. The conformation of the porphyrinoid was stabilised through the HB between the two opposite‐located NH groups and BF_4_^−^ anion on one side, and water molecule on the other face of the macrocycle. Both macrocyclic bipyridines were monoprotonated and shifted away from the positively charged N‐methyltriazolium rings. One of them was involved in the HB with water, and the other interacted through the NH⋅⋅⋅O HB with the oxygen atom of the axle, further stabilising the observed conformation of the rotaxane. The solid‐state structure demonstrated an interesting feature of calix[4]phyrin‐incorporated MIMs as potential anion‐binding agents.

**Figure 5:**
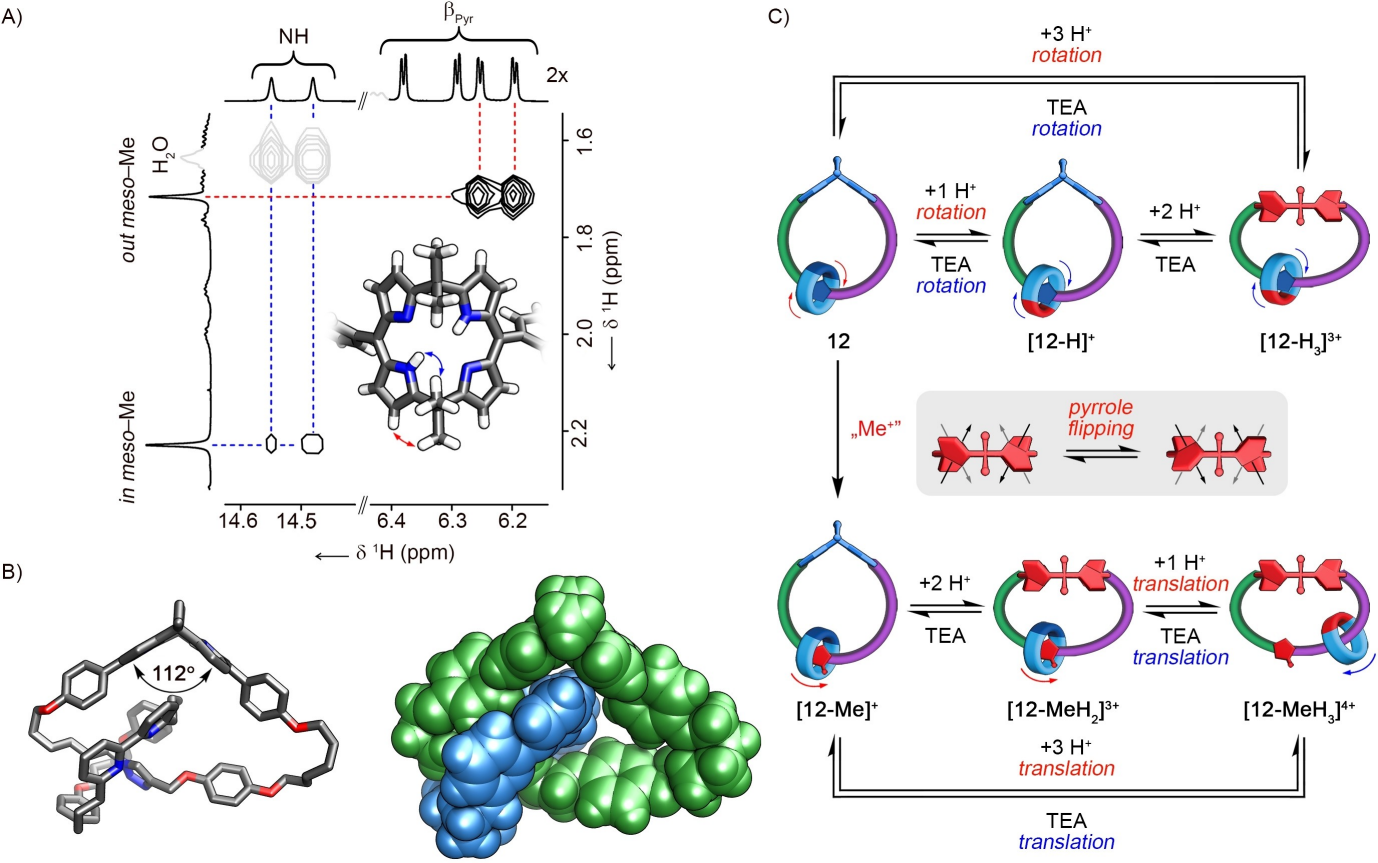
A) The part of the ^1^H−^1^H NOESY (nuclear Overhauser effect spectroscopy) spectrum of **12** showing the *in‐* and *out*‐*meso*‐Me groups correlations. B) The X‐ray molecular structure of **12**. Protons, except for those in the calix[4]phyrin cavity, are omitted for clarity.^[48]^ C) The reactivity of **12**.

Eventually, the effect of incorporating the calix[4]phyrin moiety into the catenane architecture on the system‘s dynamics was evaluated. The condensation of bis‐dipyrromethane stoppered [2]rotaxane **2** with acetone catalysed by boron trifluoride diethyl etherate, and the subsequent oxidation, resulted in the formation of the [2]catenane **12** in 31 % yield (Scheme 2). The elemental composition of the product was confirmed by MALDI MS (matrix‐assisted laser desorption/ionisation mass spectrometry), which showed a [M+H]^+^ signal at 1386.7428 (Figure S88, Supporting Information). The ^1^H NMR spectrum ([D_2_]DCM, 260 K) confirmed the [2]heterocatenane architecture of **12** composed of two different macrocyclic components, namely macrocyclic bipyridine **3** and *meso*‐*meso*‐strapped calix[4]phyrin (Figure S82, Supporting Information). Two NH signals at 14.55 and 14.48 ppm and four corresponding β‐pyrrolic resonances were identified in the 6.19–6.38 ppm range, consistent with the non‐equivalence of the two sides of calix[4]phyrin. The triazole signal at 8.13 ppm suggested an HB interaction with bipyridine of the second macrocycle. A series of NOE (nuclear Overhauser effect) peaks between the signals of *in‐* and *out‐*oriented *meso*‐Me groups of calix[4]phyrin at 2.23 and 1.72 ppm and the intracavity NH and β‐pyrrolic protons, respectively, corroborated the roof‐like conformation of the tetrapyrrole (Figure 5A).

X‐ray studies confirmed the bent conformation of calix[4]phyrin in heterocatenane **12** (Figure 5B).^[48]^ Single crystals were grown by slowly evaporating the solution in a DCM/methanol. The [2]catenane crystallised in the monoclinic system in the *P*2/*c* group. The molecular structure revealed a heterocatenane composed of a large calix[4]phyrin‐incorporating ring interlocked with a smaller macrocyclic bipyridine **3**. Both ring components differed in shape and size—the large one had an ellipsoid 19×10 Å cavity, whereas **3** was more circular with 11×10 Å size. The location of the macrocyclic bipyridine in the proximity of the triazole ring was a consequence of the CH⋅⋅⋅N hydrogen bonding stabilising the particular co‐conformation in the solid state. The calix[4]phyrin adopted the expected roof‐like conformation with the 112° dihedral angle between dipyrrins. The bending of the porphyrinoid formed a cleft occupied by a part of the macrocyclic bipyridine **3**.

To probe the dynamics of **12**, the ^1^H NMR spectra of the catenane were recorded in the 300–360 K in [D_8_]toluene (Figure S90, Supporting Information). Unlike the case of **10**, the *in‐* and *out‐meso‐*Me groups produced two well‐separated singlets in the whole evaluated temperature range, indicating that the incorporation of the calix[4]phyrin into catenane **12** deactivated the fluttering motion. This was likely due to the considerable strain introduced into the macrocycle through the ring‐closing reaction or the overcrowding of the cavity of the large ring with macrocyclic bipyridine, which prohibited this particular type of molecular motion.

Finally, the dynamics of **12** in response to external stimuli was probed (Figure 5C). Inspection of the ^1^H NMR spectra ([D_2_]DCM, 240 K) recorded during the titration of **12** with TFAH indicated that the protonation can be separated into two key phases (Figure 5C, S101, S110, Supporting Information). Unlike for **10**, the first step involved the protonation of the macrocyclic bipyridine yielding **[12‐H]^+^**, as evidenced by the rise of a new set of NH signals at 14.86 and 14.58 ppm. The spectral changes indicated that bipyridine protonation in **12** was associated with the rotation of the macrocyclic component **3**, as evidenced by the relocation of the triazole resonance from 8.26 to 6.35 ppm (Figure S96–100, Supporting Information). Once excess acid was introduced, the initially formed **[12‐H]^+^** transformed into **[12‐H_3_]^3+^**, as evidenced by four calixphyrin NH resonances in the 13.8–11.9 ppm range and the triazole signal at 6.12 ppm ([D_2_]DCM, 240 K; Figure S111, Supporting Information). Eight signals corresponding to the β‐pyrrolic protons could be identified, which implied that at low temperature, the calixphyrin acquired a conformation with tilted pyrrole rings pointing up and down in an alternate fashion. Upon a rise in the temperature, the NH and β‐pyrrolic signals broadened until their number reduced, indicating the increased rate of pyrrole rings flipping, being likely the dynamic process that resulted in averaging the positions of the respective resonances at 300 K (Figure 5C, S111, Supporting Information). The computational model of **[12‐H_3_]^3+^** retained the crucial structural features which were inferred from the NMR spectrum (Figure S178, Supporting Information). Upon the addition of a base to **[12‐H_3_]^3+^**, the neutral **12** was formed (Figure 5C, S112, Supporting Information).

The introduction of a positive charge on triazole in **12** yielding **[12‐Me]^+^** was achieved through N‐methylation and subsequent anion exchange (Scheme 2). The ^1^H NMR spectrum of **[12‐Me]^+^** did not show significant differences compared to **12**, except for the downfield shift of the triazole resonance from 8.04 to 10.26 ppm, correspondingly with the increased acidity of the CH proton in the triazolium incorporating species (Figure S139, Supporting Information).

Differently than for **12**, the acidification of **[12‐Me]^+^** with TFAH initially yielded **[12‐MeH_2_]^3+^** protonated at the calix[4]phyrin core, as evidenced by the four NH resonances at 13.92, 13.90, 13.66, and 13.33 ppm, and the triazole signal at 10.24 ppm detected in the ^1^H NMR spectrum recorded in [D_2_]DCM at 180 K (Figure 5C, S149, Supporting Information). The eight β‐pyrrolic signals identified through their correlations with four non‐equivalent NH resonances in the ^1^H−^1^H COSY (correlated spectroscopy) indicated that calix[4]phyrin adopted a conformation with tilted pyrrole rings, similar to **[12‐H_3_]^3+^** (Figure S156, Supporting Information).

The further addition of acid resulted in the formation of **[12‐MeH_3_]^4+^**. The NH groups of the protonated calix[4]phyrin produced broad resonance at 12.19 ppm, and the triazole signal was found at 8.35 ppm at 300 K ([D_2_]DCM, Figure S168, Supporting Information). Upon lowering the temperature to 230 K, the signals of the macrocyclic bipyridine and calix[4]phyrin ring were doubled, implying that the pyrrole‐flipping process was slowed down sufficiently to induce the non‐equivalence of both sides of macrocyclic components (Figure S169, Supporting Information). The protonation of component **3** in **[12‐MeH_3_]^4+^** resulted in the repulsion between the macrocyclic bipyridine and the positively charged triazolium ring. However, it was envisaged that such transformation might lead to the three principal co‐conformers differing in the orientation of the macrocyclic bipyridine with respect to the N−Me‐triazole (Figure S181, Supporting Information). Each form was stabilised through π–π stacking between the *p*‐phenylenes of **3** and one of the aromatic units incorporated into the strap of the large calix[4]phyrin‐incorporating macrocycle. In addition, the stabilising effect of the NH⋅⋅⋅O HB involving protonated bipyridine and oxygen atoms within the strap was expected to be of importance. The relative energies calculated for the three major co‐conformers ranged from 0 to 14.7 kcal/mol, indicating that the lowest‐energy form was characterised by the close proximity between protonated macrocyclic bipyridine and *p*‐dialkoxyphenylene unit next to the N‐methyltriazolium. This species was also identified in the solution, based on the NOE between the *p*‐dialkoxyphenylene protons and *p*‐phenylene signals of macrocyclic bipyridine (Figure S165–167, Supporting Information). The estimated transition barrier between the co‐conformations differing in the position of the small macrocycle in regard to the side of N−Me triazolium indicated that passing through this station is practically impossible, and thus, the translation accompanying the formation of **[12‐MeH_3_]^4+^** is unidirectional (Animation S2, Figure S181, Supporting Information). Deprotonation of **[12‐MeH_3_]^4+^** with TEA retrieved **[12‐Me]^+^** (Figure 5C, S170, Supporting Information).

## Conclusion

The synthesis of [2]rotaxanes incorporating one or two dipyrromethane‐based stoppers opened a route for the construction of a new group of mechanically interlocked porphyrinoids. These building blocks were demonstrated to easily undergo condensation to yield, depending on the carbonyl reactant, porphyrin‐ or calix[4]phyrin‐incorporating architectures, including [3]rotaxane, Y‐shaped [4]rotaxane, and [2]catenane.

The presence of a calix[4]phyrin moiety, introduced into the rotaxane molecule as a structural element of the axle, activated a new mode of molecular motion, termed fluttering, which supplements the well‐known type of movements, i.e., shuttling and pirouetting. The facile and reversible transformations of [3]rotaxane and [2]catenane allowed for control over the dynamic behaviour of the system, which involved turning on and off the fluttering process and associated shuttling/pirouetting of the bipyridine‐based macrocycle. The protonated calix[4]phyrin‐incorporating rotaxane revealed a potential for anion‐binding, encouraging the use of this feature as a trigger to control dynamic behaviour.

The research described herein opens a route to the construction of a new class of mechanically interlocked systems incorporating porphyrinoid frameworks. It is envisaged that modifying mechanically interlocked dipyrromethane will allow the creation of MIMs encompassing other than porphyrin/calix[4]phyrin stations and will extend the repertoire of exciting interlocked architectures. This, on the other hand, will reveal new possibilities in terms of the generation of the mechanically interlocked porphyrinoids possessing tunable redox properties, unusual aromaticity, cation and anion binding properties, peculiar conformational dynamics, or intriguing reactivity stimulated by the presence of mechanical bond. Eventually, the introduction of a new type of molecular motion achieved due to the intrinsic dynamics of calix[4]phyrin moiety enables the design of novel molecular machines with predictable and well‐controlled behaviour.

## Supporting Information

The authors declare that the data supporting the findings of this study are available within the paper, its Supporting Information files, and the Cambridge Crystallographic Data Centre (deposition numbers 2357365 for **[10‐Me_2_H_4_](BF_4_)_6_**, 2357366 for **9** and 2357367 for **12**). The crystal structures and structure factor data can be obtained free of charge from the Cambridge Crystallographic Data Centre via www.ccdc.cam.ac.uk/data_request/cif.

## Conflict of Interests

The authors declare no conflict of interest.

1

## Supporting information

As a service to our authors and readers, this journal provides supporting information supplied by the authors. Such materials are peer reviewed and may be re‐organized for online delivery, but are not copy‐edited or typeset. Technical support issues arising from supporting information (other than missing files) should be addressed to the authors.

Supporting Information

Supporting Information

Supporting Information

## Data Availability

The data that support the findings of this study are available in the supplementary material of this article.
