# Supplementary material for: Multimodal Molecular Motion in the Rotaxanes and Catenanes Incorporating Flexible Calix[n]phyrin Stations
Source: Angew Chem Int Ed Engl. 2024 Oct 23;64(1):e202413579. doi: 10.1002/anie.202413579 (PMC11701352; doi:10.1002/anie.202413579)
Supplement: Supplementary file 3 — Supporting Information [file ANIE-64-e202413579-s001.pdf]

Angewandte Chemie
Eine Zeitschrift der Gesellschaft Deutscher Chemiker

Supporting Information

Multimodal Molecular Motion in the Rotaxanes and Catenanes Incorporating Flexible Calix[*n*]phyrin Stations

*R. A. Grzelczak, T. Basak, B. Trzaskowski, V. Kinzhybalo, B. Szyszko**

Supporting Information

©Wiley-VCH 2021

69451 Weinheim, Germany

Multimodal Molecular Motion in the Rotaxanes and Catenanes Incorporating Flexible Calix[*n*]phyrin Stations

Rafał Grzelczak, Tymoteusz Basak, Bartosz Trzaskowski, Vasyl Kinzhybalo, Bartosz Szyszko*

Abstract: The synthesis of [2]rotaxanes stoppered with one or two dipyrromethane groups opened a route for the construction of mechanically interlocked molecules incorporating various porphyrinoid stations. The exploitation of those precursors allowed for the creation of [3]rotaxanes and [2]catenanes based on the calix[4]phyrin motif, presenting intriguing molecular dynamics. The intrinsic flexibility of the porphyrinoid allowed the introduction of a new type of molecular motion within the rotaxanes, termed fluttering. The latter involved a bending of the axle, interconverting two angular-shaped stereoisomers of the rotaxane through a planarised transition state. Simple chemical transformations, i.e. methylation and (de)protonation of [3]rotaxane and [2]catenane allowed for the controllable transformations within the conformationally flexible calix[4]phyrin-incorporated mechanically interlocked porphyrinoids.

DOI:

SUPPORTING INFORMATION

Experimental Procedures 1
Instrumentation 1
Synthesis 2
Macrocycle synthesis 2
Half-axles synthesis 3
Compound S10 4
Compound 4 4
Compound 6 5
Compound 7 9
Compound 8 13
Rotaxane synthesis 18
Compound 1 19
Compound 2 26
Synthesis of porphyrinoid-based interlocked molecules 32
Compound 9 33
Compounds 10 and 11 39
Compound $[10\text{-}H_2]^{2+}$ 52
Compound $[10\text{-}H_4]^{4+}$ 58
Compound 12 65
Compound $[12\text{-}H]^{+}$ 73
Compound $[12\text{-}H_3]^{3+}$ 82
Synthesis of methylated compounds 90
Compound $[10\text{-}Me_2]^{2+}$ 91
Compound $[10\text{-}Me_2H_2]^{4+}$ 99
Compound $[10\text{-}Me_2H_4]^{6+}$ 105
Compound $[12\text{-}Me]^{+}$ 112
Compound $[12\text{-}MeH_2]^{3+}$ 119
Compound $[12\text{-}MeH_3]^{4+}$ 127
Computational studies 135
References 172

Experimental Procedures

Instrumentation

NMR spectroscopy
The ^{1}H NMR, ^{13}C NMR, and ^{19}F NMR spectra were recorded on high-field Bruker spectrometers (600 and 500 MHz) equipped with a broadband inverse gradient probe head and a high-field JEOL spectrometer (500 MHz), equipped with a 5 mm wide wideband probe. The spectra were referenced to the residual solvent signal ([D]chloroform – 7.24 ppm, [D_2]dichloromethane – 5.32 ppm, [D_8] toluene – 7.00 ppm). Two-dimensional NMR spectra were recorded with 2048 data points in the t_2 domain and up to 1024 points in the t_1 domain, with a 1 s recovery delay.

Mass spectrometry
The MALDI mass spectra were recorded on JEOL JMS-S3000 SpiralTOF™-plus Ultra-High Mass Resolution MALDI-TOF MS. ESI mass spectra were recorded on Bruker qTOF compact.

X-ray diffraction data
Single-crystal X-ray diffraction data for all crystals were collected at 100 K with the use of Cu Kα radiation on Rigaku XtaLAB Synergy R DW system equipped with HyPix-Arc 150 hybrid detector and Oxford Cryosystems 800 temperature unit. Data reduction was carried out with the use of the CrysAlis Pro program.[1] The crystal structures were solved with ShelxT[2] and refined with ShelxL programs.[3] All crystal structures include some disorder.

In **[10-Me$_2$H$_4$][BF$_4$]$_6$**, some BF_4^- anions were restrained with B–F and F–F distances of 1.40(2) and 2.25(2) Å, respectively. Two out of four *tert*-butyl group substituents are disordered over two sites. Some minor disorder was modelled on one of two bipyridine macrocycles. The positions of four ethyl acetate, one DCM and two water molecules were located, whereas the remaining disordered solvent moieties were treated with BYPASS procedure[4] implemented in Olex2.[5]
In **9**, all *tert*-butyl group substituents are disordered over two sites. Some disorder is modelled on bipyridine macrocycle and pentafluorophenyl ring. The only solvent molecule – DCM, is disordered over two sites.
In **12**, both macrocyclic rings reveal some minor disorder. The solvent DCM molecule is disordered over two sites due to a two-fold axis passing through one of the chlorine atoms.
Detailed information on disorder treatment is included in the CIF file.

Computational studies
Two different computational methods were used to obtain theoretical information about the studied systems. For the model calix[4]phyrin molecule, **1**, **2** and **10**, **[10-H$_4$]$^{4+}$** and **[12-H$_3$]$^{3+}$** the DFT calculations were performed using the B3LYP functional[6,7] with the D3 dispersion correction[8] and the 6-31G** basis set.[9,10] These calculations have been performed in $CHCl_3$ solution modelled at the PCM level of theory[11] using Jaguar ver. 11.2 software.[12] Calculations for all other systems have been performed at the GFN2-xTB level of theory[13] using the analytical linearised Poisson-Boltzmann model of implicit solvent for $CHCl_3$. Conformational searches have been performed using the Conformer–Rotamer Ensemble Sampling Tool (CREST)[14] and the following settings: RMSD threshold between conformer pairs of 3.0 Å, energy threshold of 0.5 kcal/mol and energy sampling window of 30 kcal/mol. For selected larger systems, the –quick or –squick options to perform the search with reduced settings for a quicker conformational search were used. To obtain potential energy surface scans the growing string method with the default values and either 50 or 100 steps was used; in each growing strings calculations, the start and end points were first subject to geometry optimisation at the GFN2-xTB level.[15] Finally, in the molecular dynamics calculations, standard parameters implemented in the xtb ver. 6.4.1 software were used and performed 5 ns of NVT dynamics at either 210 or 300 K. For cationic forms a conformational search at the GFN2-xTB level was performed for the charged system as well as a neutral system containing acetate anions, and the resulting conformation was very similar, therefore, in all other calculations, no counterions were used for charged systems. These calculations have been performed either in no solution or in $CHCl_3$ solution modelled at the PCM level of theory[11] using Jaguar ver. 11.2 software.12.

Synthesis

Macrocycle synthesis

Compounds **S3**,[16] **S4-3**[17] were synthesised as described in the literature.

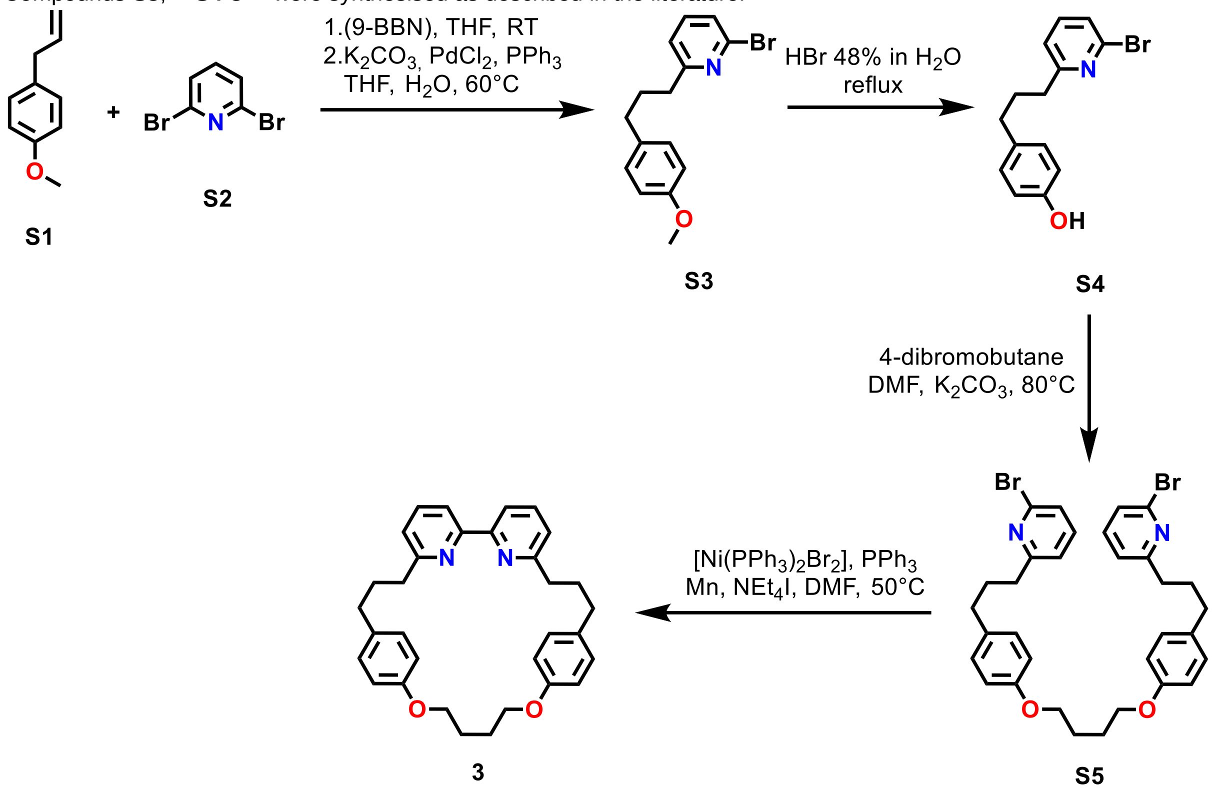

Scheme S 1. The synthesis of **3**.

Half-axles synthesis

Compounds **5**,[18] **S8**,[19] **S13**[20] were synthesised as described in the literature.

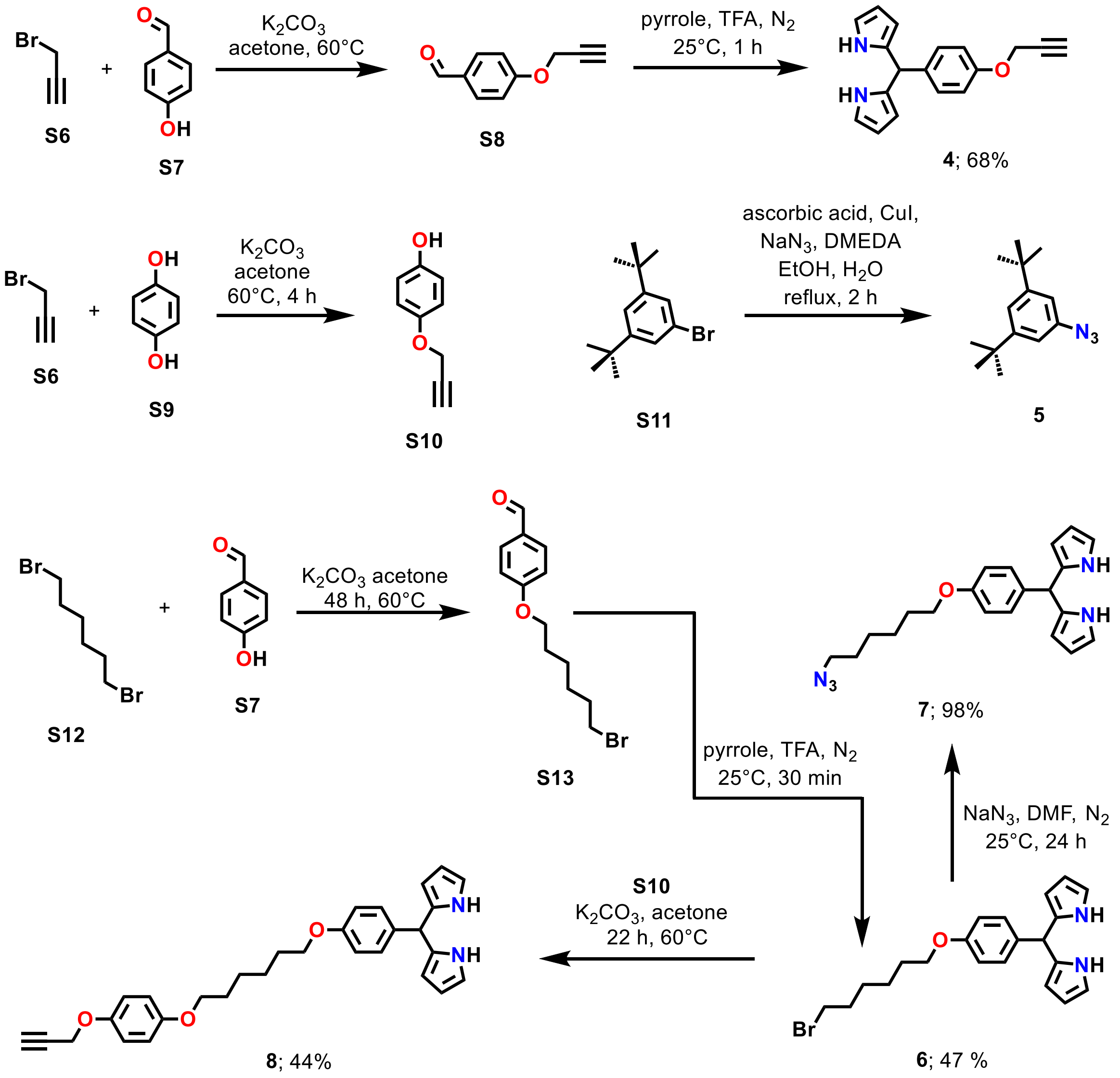

Scheme S 2. The synthesis of **4**, **5**, **7** and **8**.

Compound S10

S10 was obtained under modified literature conditions.[21]

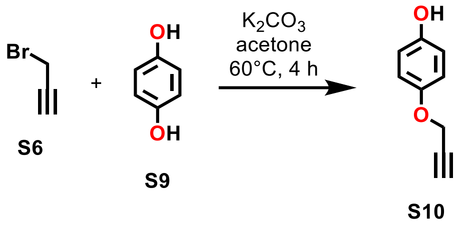

In a 100 ml round bottom flask, hydroquinone (3.73 g, 34 mmol), potassium carbonate (7.02 g, 50.8 mmol) and acetone (170 ml) was introduced. The mixture was then stirred for 30 minutes. Then, propargyl bromide 80% in toluene (2.4 ml, 21.7 mmol) was added. After this, the reaction mixture was stirred at 60°C for 4 hours. After cooling, the mixture was filtered, and the solvent was evaporated under vacuum. The residue was purified *via* column chromatography (hexane:ethyl acetate 20:1) to provide **S10** (1.2 g, 8 mmol, 37%) as a pale yellow oil.
Analytical data are in agreement with the published one.

Compound 4

4 was obtained under modified literature conditions.[22]

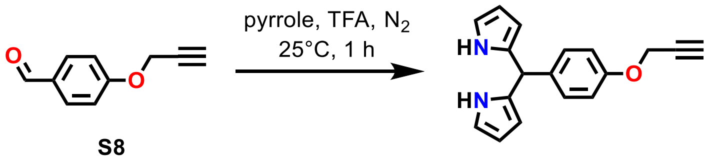

4; 68%

In a 50 ml round bottom flask, **S8** (2.80 g, 17.48 mmol) and pyrrole (25 ml, 0.35 mol) were introduced. The pyrrole was previously purified by passing through a column filled with aluminum oxide. The mixture was flushed via nitrogen bubbling under a sealed flask for 10 minutes. Then TFA (140 µl, 1.83 mmol) was added by a syringe. The mixture was then stirred for 1 h under a nitrogen atmosphere. After this, the reaction was quenched by 1 M NaOH (25 ml). The product was extracted with ethyl acetate (100 ml). The organic layer was washed with distilled water and then with brine. The aqueous phase was again extracted with ethyl acetate (100 ml). Collected organic layers were dried over Na_2SO_4. The filtrate was collected *via* gravity filtration, and the solvent was removed under reduced pressure. The dark oil was purified *via* column chromatography (silica gel, DCM) to provide **4** (3.31 g, 11.98 mmol, 68%) as a yellowish oil which turned dark over time.
Analytical data are in agreement with the published one.

Compound 6

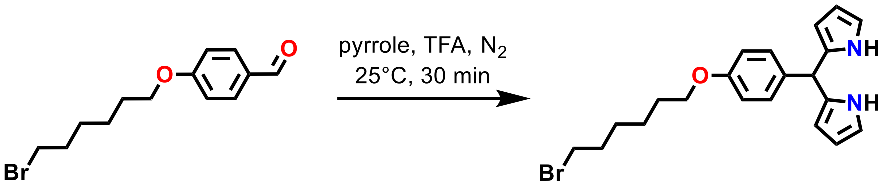

S13 **6**; 47 %

In a 50 ml round bottom flask, **S13** (2.50 g, 8.77 mmol) and pyrrole (25 ml, 0.35 mol) were introduced. The pyrrole was previously purified by passing through a column filled with aluminum oxide. The mixture was flushed via nitrogen bubbling under a sealed flask for 10 minutes. Then TFA (140 µl, 1.83 mmol) was added by a syringe. The mixture was then stirred for 30 min under a nitrogen atmosphere. After this, the reaction was quenched by 1 M NaOH (25 ml). The product was extracted with ethyl acetate (100 ml). The organic layer was washed with distilled water and then with brine. The aqueous phase was again extracted with ethyl acetate (100 ml). Collected organic layers were dried over Na_2SO_4. The filtrate was collected *via* gravity filtration, and the solvent was removed under reduced pressure. The dark oil was purified *via* column chromatography (DCM:hexane 4:1) to provide **6** (1.65 g, 4.12 mmol, 47%) as a yellowish oil which turned dark after time.

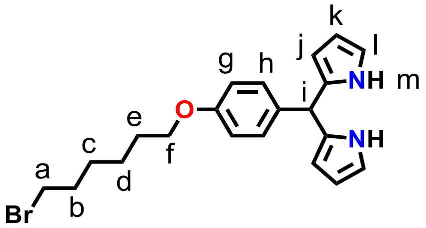

^{1}H NMR (500 MHz, [D]chloroform, 300 K, ppm): δ 7.90 (b, 2H, H_m), 7.10 (d, ^3J = 8.7 Hz, 2H, H_h), 6.82 (d, ^3J = 8.7 Hz, 2H, H_g), 6.68–6.66 (m, 2H, H_l), 6.15–6.12 (m, 2H, H_k), 5.91–5.88 (m, 2H, H_j), 5.41 (s, 1H, H_i), 3.92 (t, ^3J = 6.4 Hz, 2H, H_f), 3.40 (t, ^3J = 6.8 Hz, 2H, H_a), 1.92–1.84 (m, 2H, H_b), 1.80–1.74 (m, 2H, H_e), 1.52–1.45 (m, 4H, H_c, H_d); **^{13}C NMR** (125 MHz, [D]chloroform, 300 K, ppm): δ 158.1, 134.1, 132.9, 129.4, 117.1, 114.6, 108.4, 107.0, 67.8, 43.2, 33.8, 32.7, 29.1, 27.9, 25.3; **HR–MALDI–MS** (*m/z*): ***6*** *was oxidised to dipyrrin with DDQ to record the MS spectrum*, $[M+H]^+$ calcd. for $C_{21}H_{24}BrN_2O^+$, 399.1067; found, 399.1009.

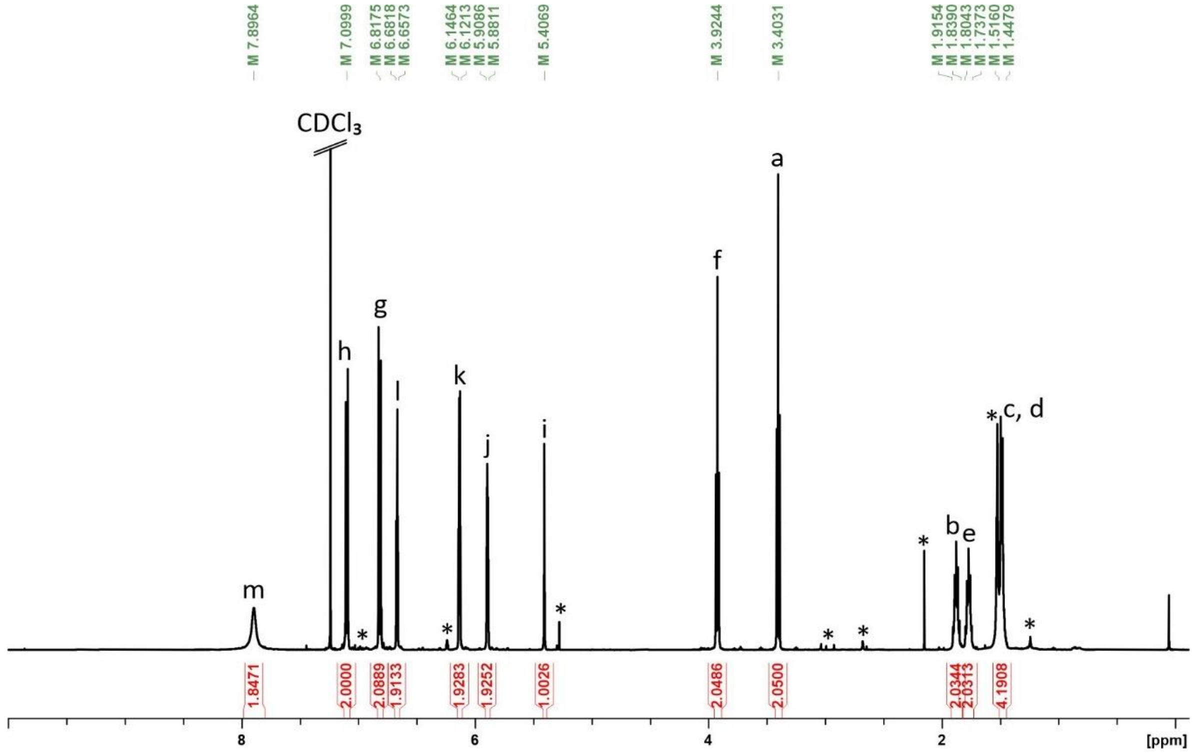

Figure S 1. The ^{1}H NMR spectrum of **6** (500 MHz, [D]chloroform, 300 K).

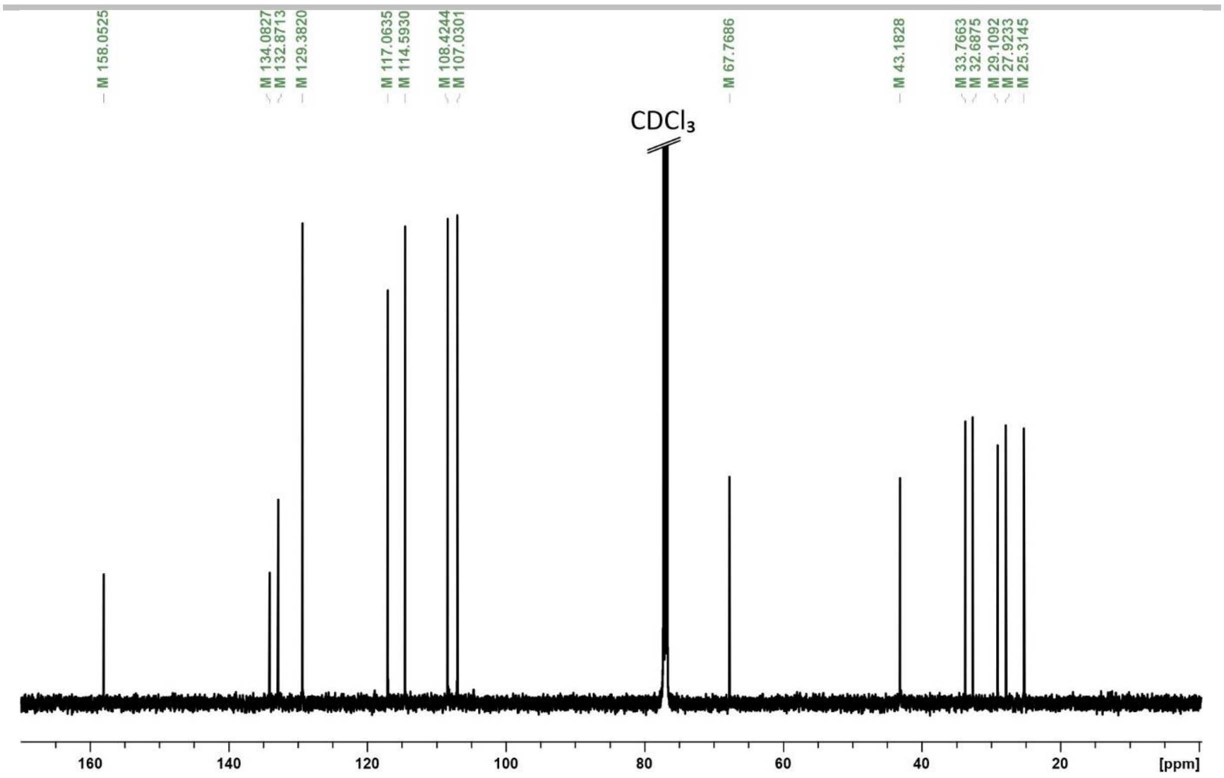

Figure S 2. The ^{13}C NMR spectrum of **6** (125 MHz, [D]chloroform, 300 K).

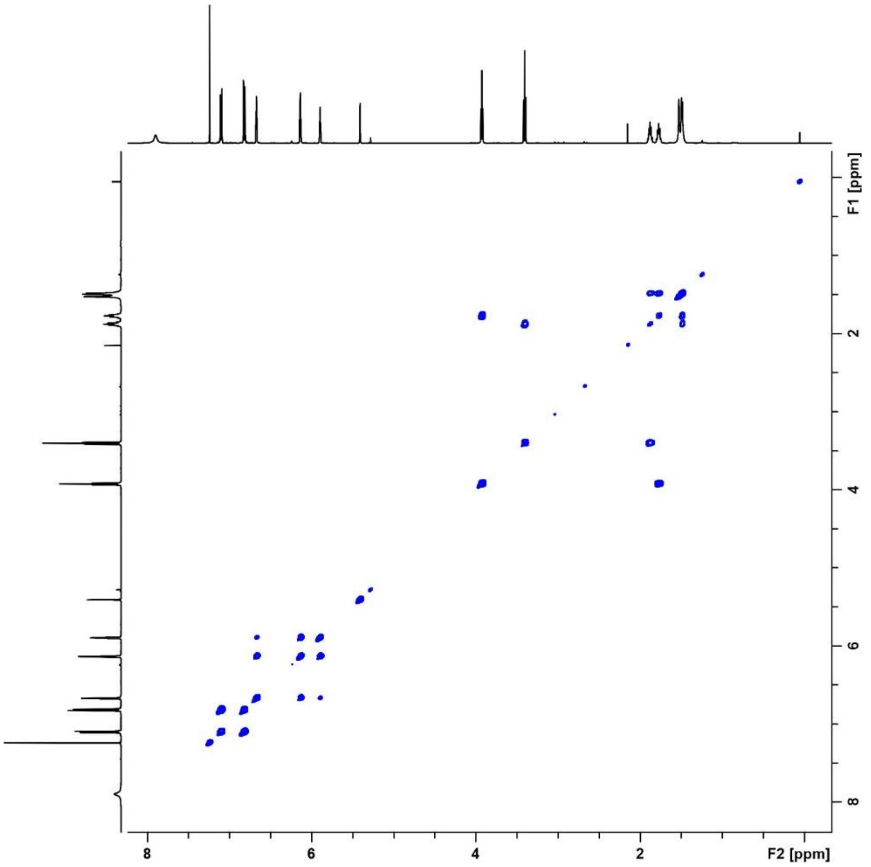

Figure S 3. The ^{1}H-^{1}H COSY NMR spectrum of **6** (500 MHz, [D]chloroform, 300 K).

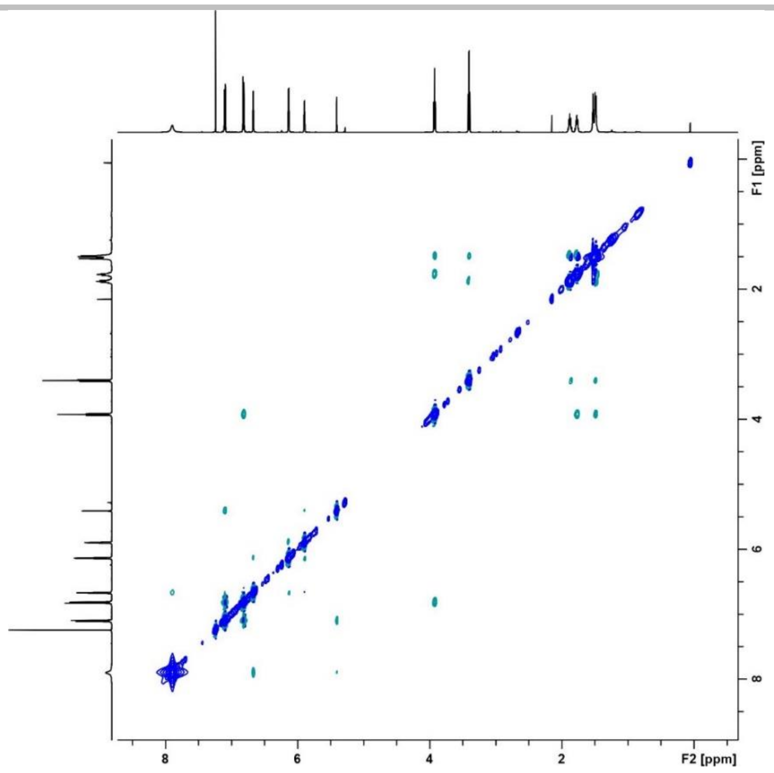

Figure S 4. The ^{1}H-^{1}H NOESY NMR spectrum of **6** (500 MHz, [D]chloroform, 300 K).

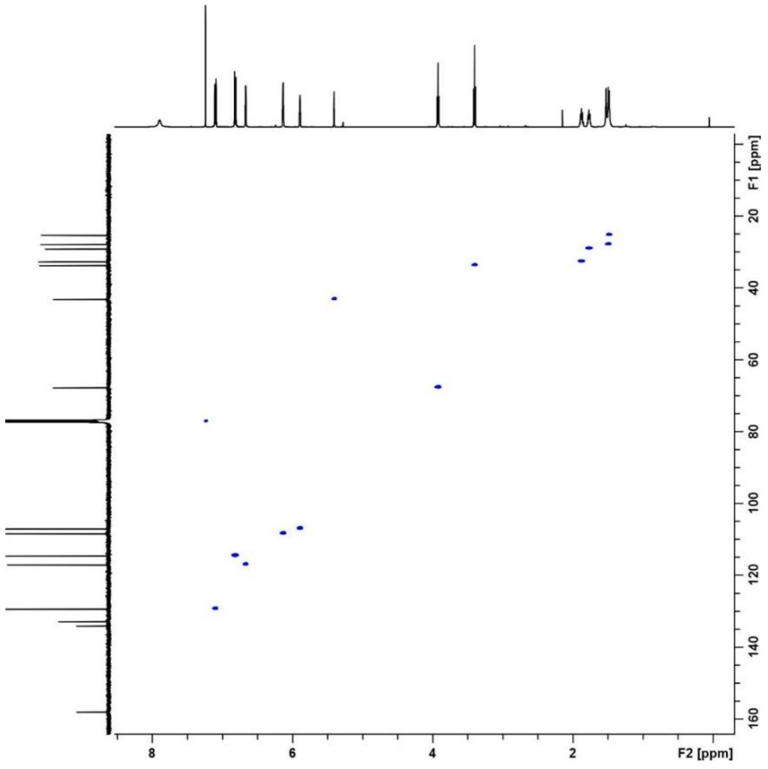

Figure S 5. The ^{1}H-^{13}C HSQC NMR spectrum of **6** (500 MHz, [D]chloroform, 300 K).

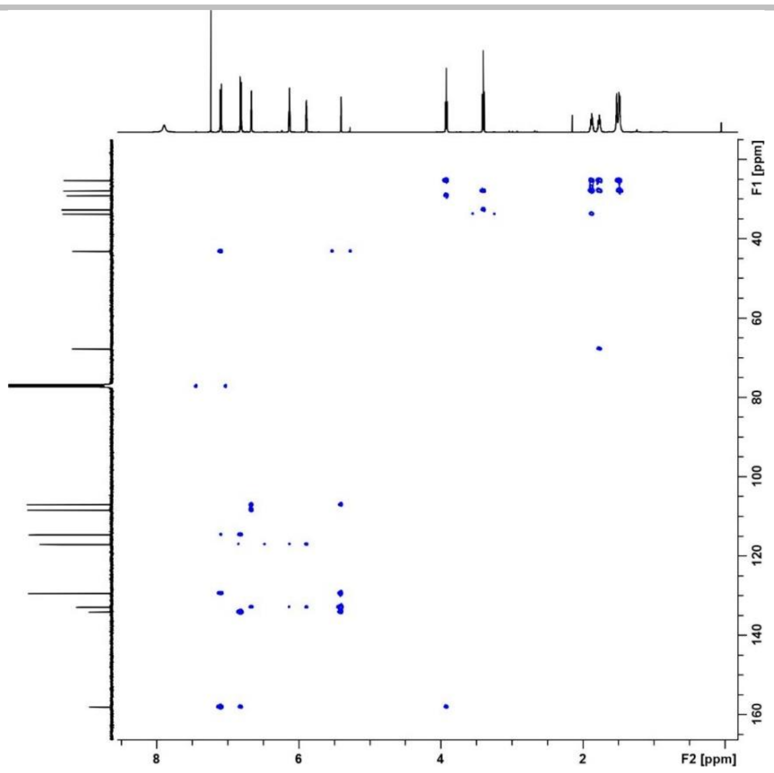

Figure S 6. The ^{1}H-^{13}C HMBC NMR spectrum of **6** (500 MHz, [D]chloroform, 300 K).

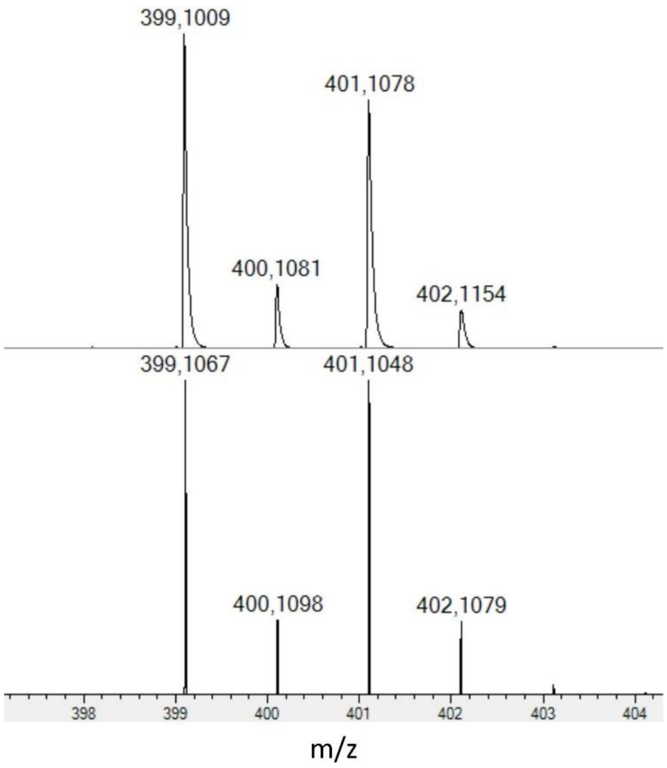

Figure S 7. The high-resolution MALDI mass spectrum of **6**. The spectrum was recorded upon the addition of DDQ to **6** in order to form dipyrrin, which improved ionisation. Top: experimental spectrum, bottom: simulated isotopic pattern.

Compound 7

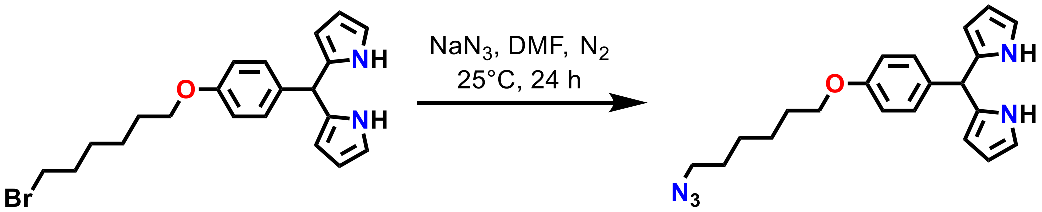

6 **7**; 98%

In a 25 ml round-bottom flask, **6** (380 mg, 0.95 mmol), sodium azide (100 mg, 1.53 mmol), and DMF (15 ml, 0.93 mmol) were introduced. The flask was sealed with a rubber septum, and the solution was stirred at room temperature for 24 hours under a nitrogen atmosphere. The solvent was removed under reduced pressure. The residue was partitioned between ethyl acetate (50 ml) and distilled water (50 ml). The water phase was extracted with ethyl acetate (2 x 50 ml). Collected organic extracts were washed with brine and dried over anhydrous Na_2SO_4. The drying agent was removed *via* gravity filtration, and the filtrate was evaporated to dryness to provide **7** (337 mg, 98%) as a greenish oil. The product was used without any additional purification.

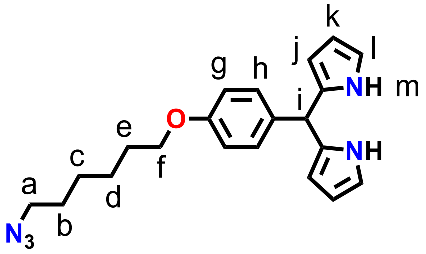

^{1}**H NMR** (500 MHz, [D]chloroform, 300 K, ppm): δ 7.90 (b, 2H, H_m), 7.10 (d, ^3J = 8.7 Hz, 2H, H_h), 6.82 (d, ^3J = 8.7 Hz, 2H, H_g), 6.68–6.66 (m, 2H, H_l), 6.15–6.11 (m, 2H, H_k), 5.91–5.88 (m, 2H, H_j), 5.40 (s, 1H, H_i), 3.92 (t, ^3J = 6.4 Hz, 2H, H_f), 3.26 (t, ^3J = 6.9 Hz, 2H, H_a), 1.81–1.73 (m, 2H, H_e), 1.66–1.58 (m, 2H, H_b), 1.52–1.39 (m, 4H, H_c, H_d); ^{13}**C NMR** (125 MHz, [D]chloroform, 300 K, ppm): δ 158.0, 134.1, 132.9, 129.3, 117.0, 114.5, 108.3, 107.0, 67.7, 51.3, 43.1, 29.1, 28.7, 26.4, 25.6; **HR–MALDI–MS** (*m/z*): ***7*** *was oxidised to dipyrrin with DDQ to record the MS spectrum*, $[M+H]^+$ calcd. for $C_{21}H_{24}N_5O^+$, 362.1975; found, 362.1970.

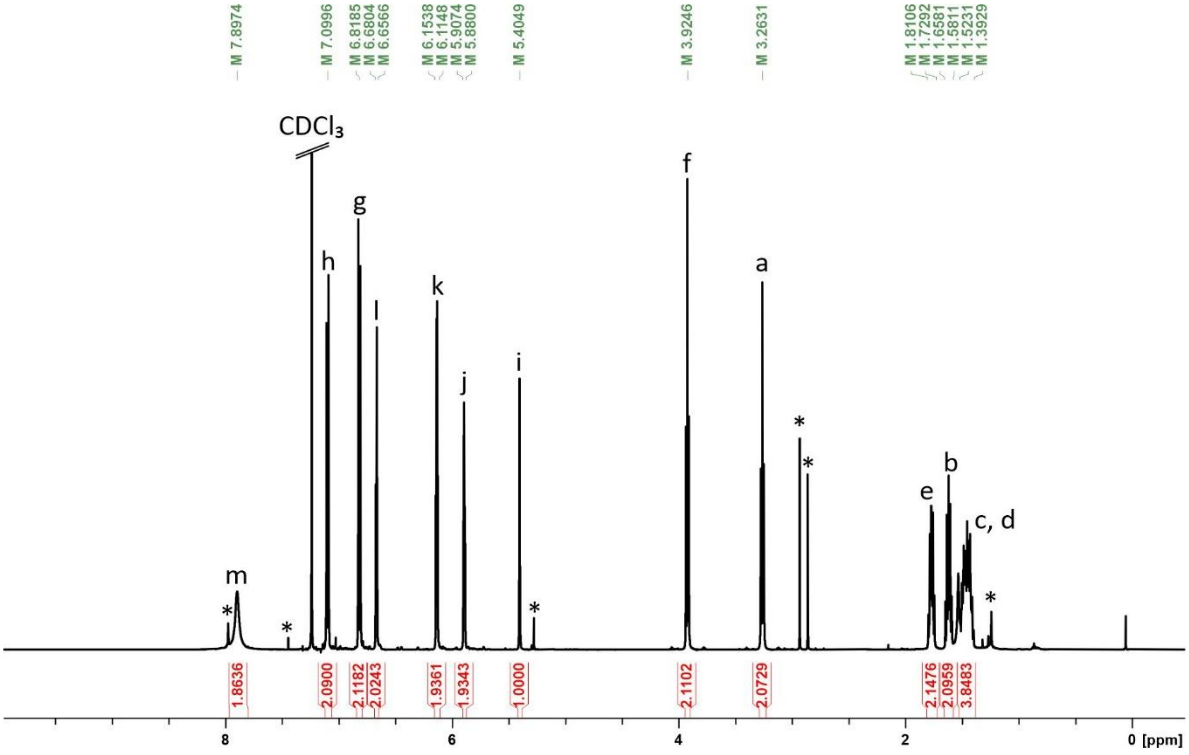

Figure S 8. The ^{1}H NMR spectrum of **7** (500 MHz, [D]chloroform, 300 K).

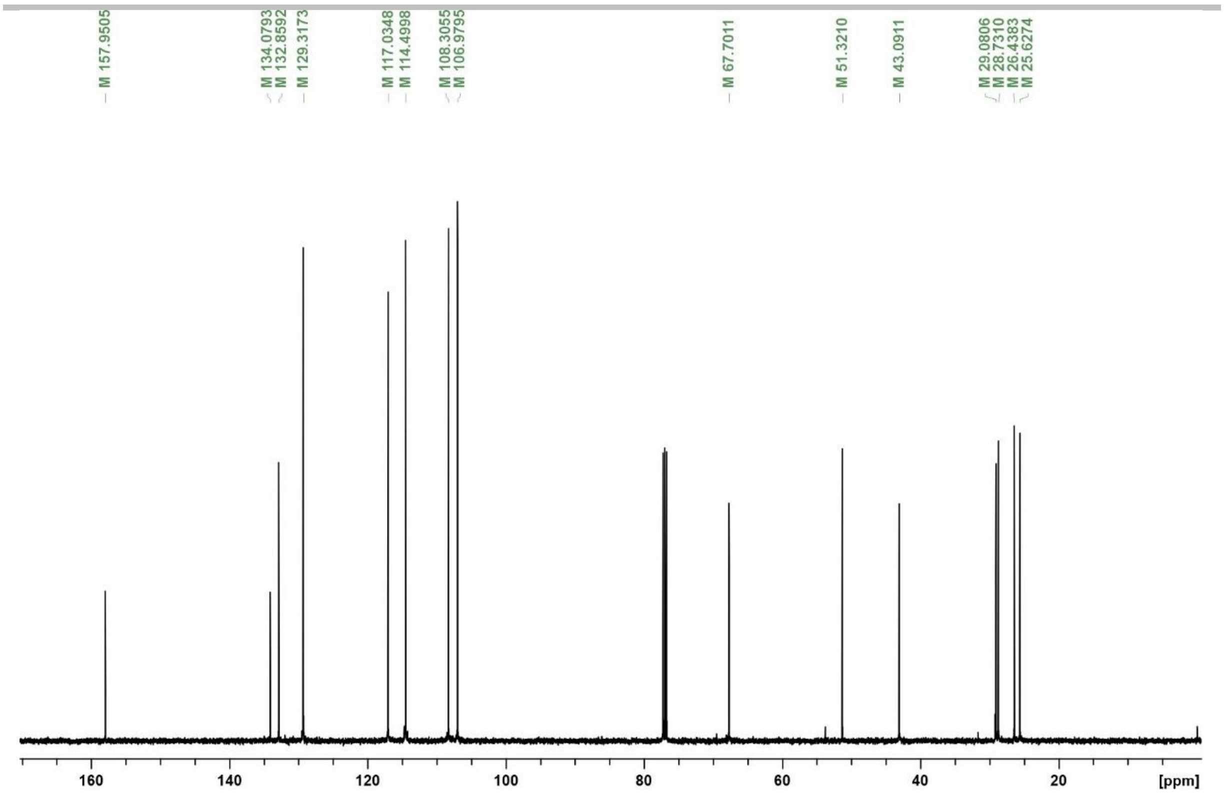

Figure S 9. The ^{13}C NMR spectrum of **7** (125 MHz, [D]chloroform, 300 K).

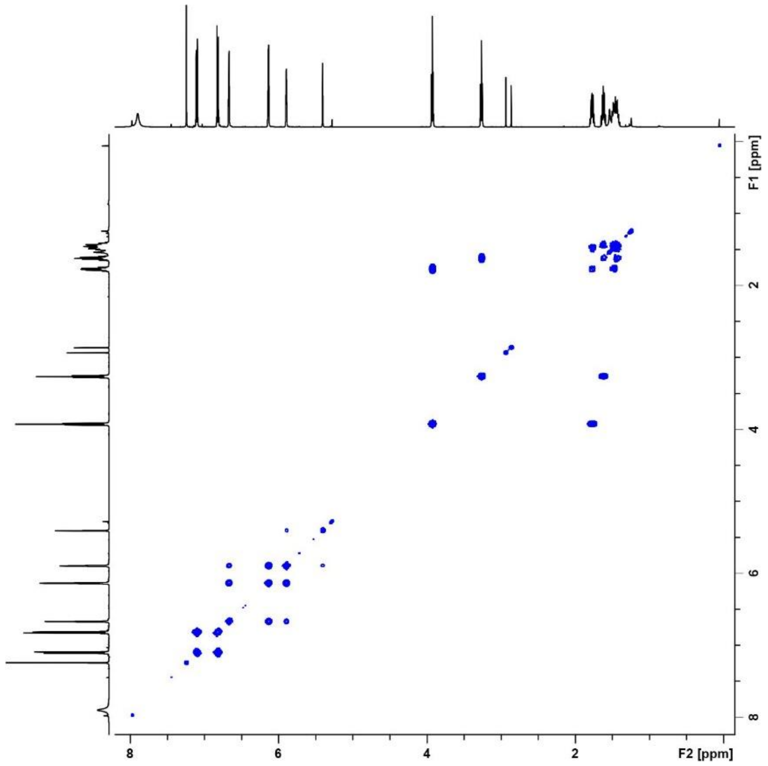

Figure S 10. The ^{1}H-^{1}H COSY NMR spectrum of **7** (500 MHz, [D]chloroform, 300 K).

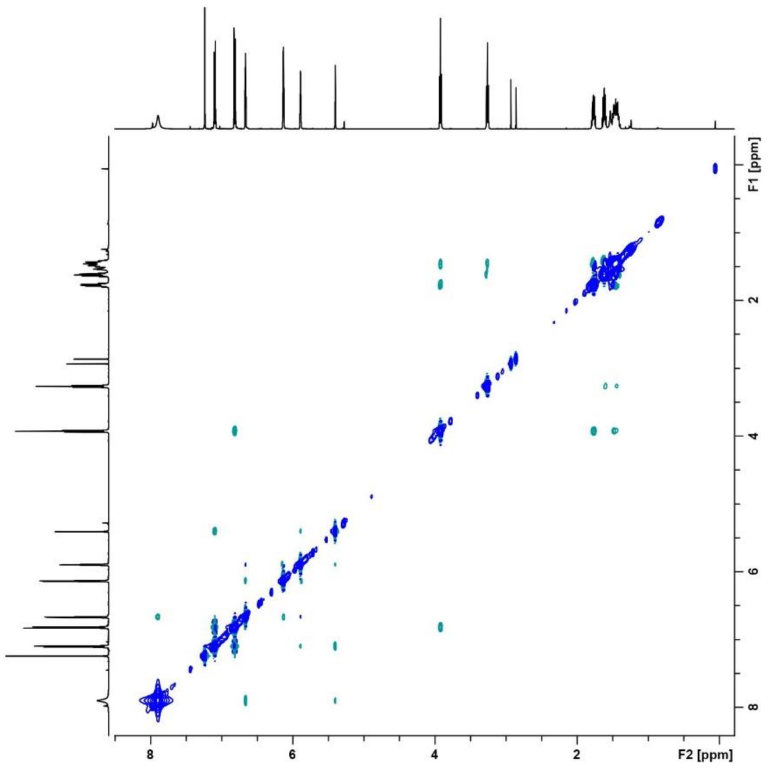

Figure S 11. The ^{1}H-^{1}H NOESY NMR spectrum of **7** (500 MHz, [D]chloroform, 300 K).

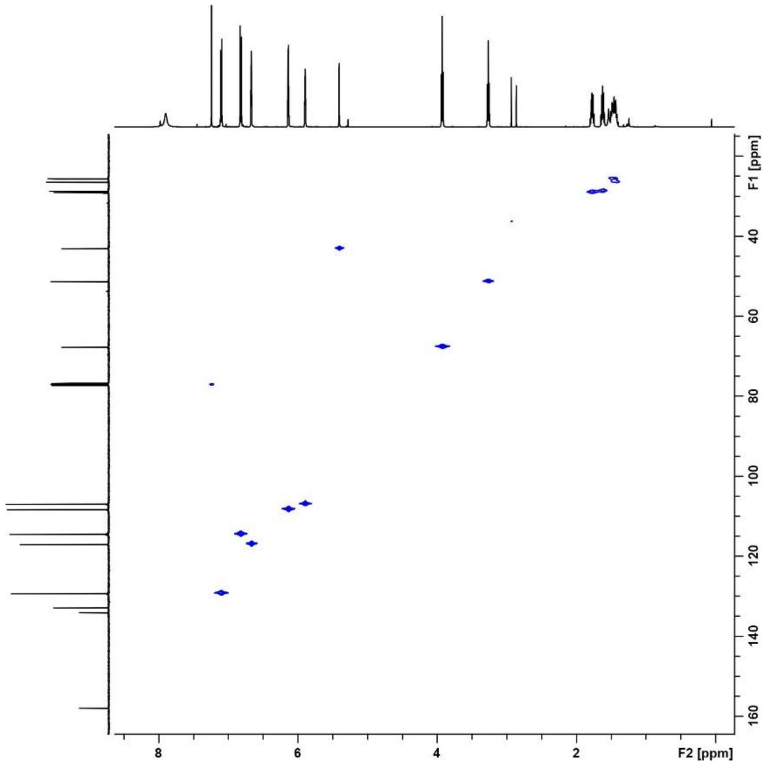

Figure S 12. The ^{1}H-^{13}C HSQC NMR spectrum of **7** (500 MHz, [D]chloroform, 300 K).

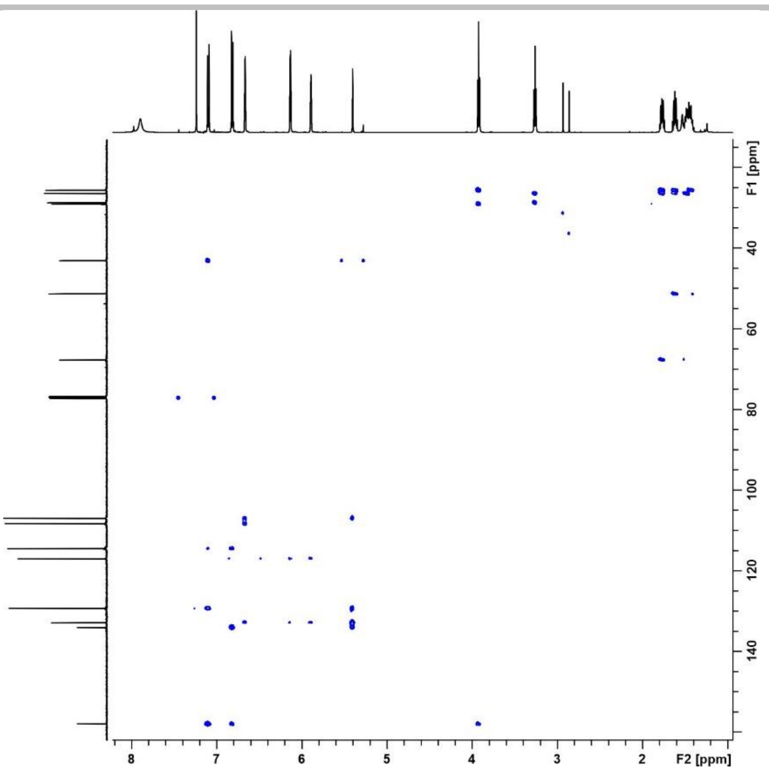

Figure S 13. The ^{1}H-^{13}C HMBC NMR spectrum of **7** (500 MHz, [D]chloroform, 300 K).

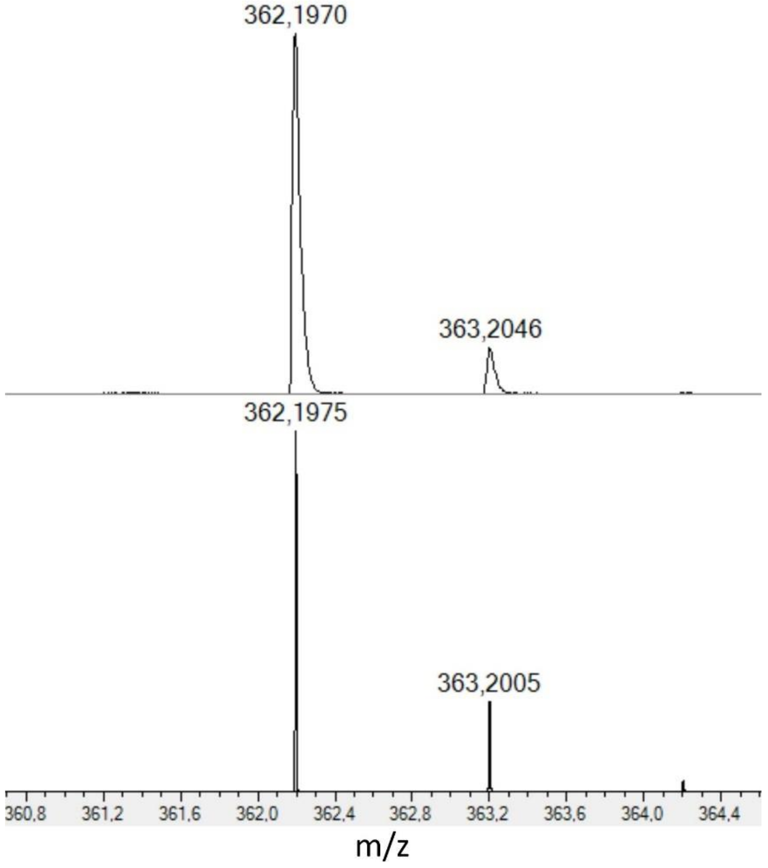

Figure S 14. The high-resolution MALDI mass spectrum of **7.** The spectrum was recorded upon the addition of DDQ to **7** in order to form dipyrrin, which improved ionisation. Top: experimental spectrum, bottom: simulated isotopic pattern.

Compound 8

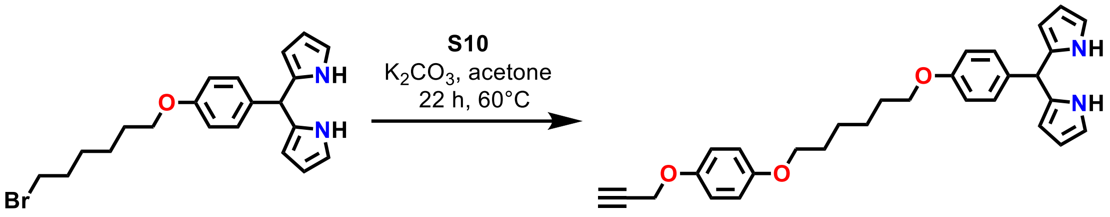

6

8; 44%

In a 50 ml round-bottom flask **6** (341 mg, 0.85 mmol), **S10** (151.6 mg, 1.02 mmol), K_2CO_3 (1.125 g, 8.14 mmol) and acetone (30 ml) were introduced. The mixture was then stirred at 60 °C for 22 h. The solvent was removed under reduced pressure. The residue was partitioned between DCM (50 ml) and distilled water (50 ml). The water phase was extracted with DCM (50 ml). The collected organic extracts were washed with brine and dried over anhydrous Na_2SO_4. The drying agent was removed *via* gravity filtration, and the filtrate was evaporated to dryness. The dark oil was purified *via* column chromatography (DCM 4:1 hexane) and then recrystallised (DCM/hexane) and placed in the fridge. The precipitate was separated from the solvent *via* gravity filtration to provide **8** (173.3 mg, 0.37 mmol, 44%) as an off-white precipitate, which turned dark after time.

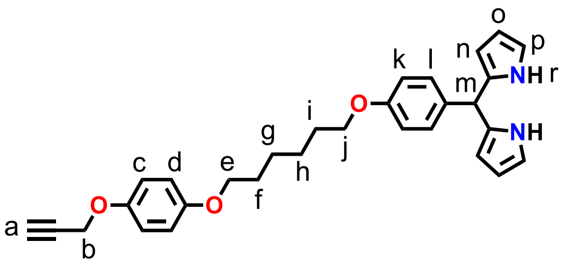

^{1}H NMR (500 MHz, [D]chloroform, 300 K, ppm): δ 7.90 (b, 2H, H_r), 7.10 (d, ^3J = 8.7 Hz, 2H, H_l), 6.89 (d, ^3J = 9.1 Hz, 2H, H_c), 6.84–6.80 (m, 4H, H_k, H_d), 6.68–6.65 (m, 2H, H_p), 6.15–6.12 (m, 2H, H_o), 5.91–5.88 (m, 2H, H_n), 5.40 (s, 1H, H_m), 3.93 (t, ^3J = 6.4 Hz, 2H, H_e/H_j), 3.91 (t, ^3J = 6.4 Hz, 2H, H_e/H_j), 2.48 (t, ^3J = 2.4 Hz 1H, H_a), 1.82–1.74 (m, 4H, H_f, H_i), 1.54–1.49 (m, 4H, H_g, H_h); **^{13}C NMR** (125 MHz, [D]chloroform, 300 K, ppm): δ 158.0, 153.9, 151.6, 134.0, 132.9, 129.3, 117.0, 116.1, 115.3, 114.5, 108.3, 107.0, 78.9, 75.3, 68.3, 67.8, 56.6, 43.1, 29.23, 29.16, 25.8; **HR–MALDI–MS** (*m/z*): ***8*** *was oxidised to dipyrrin with DDQ to record the MS spectrum*, $[M+H]^+$calcd. for $C_{30}H_{31}N_2O_3^+$, 467.2329; found, 467.2378.

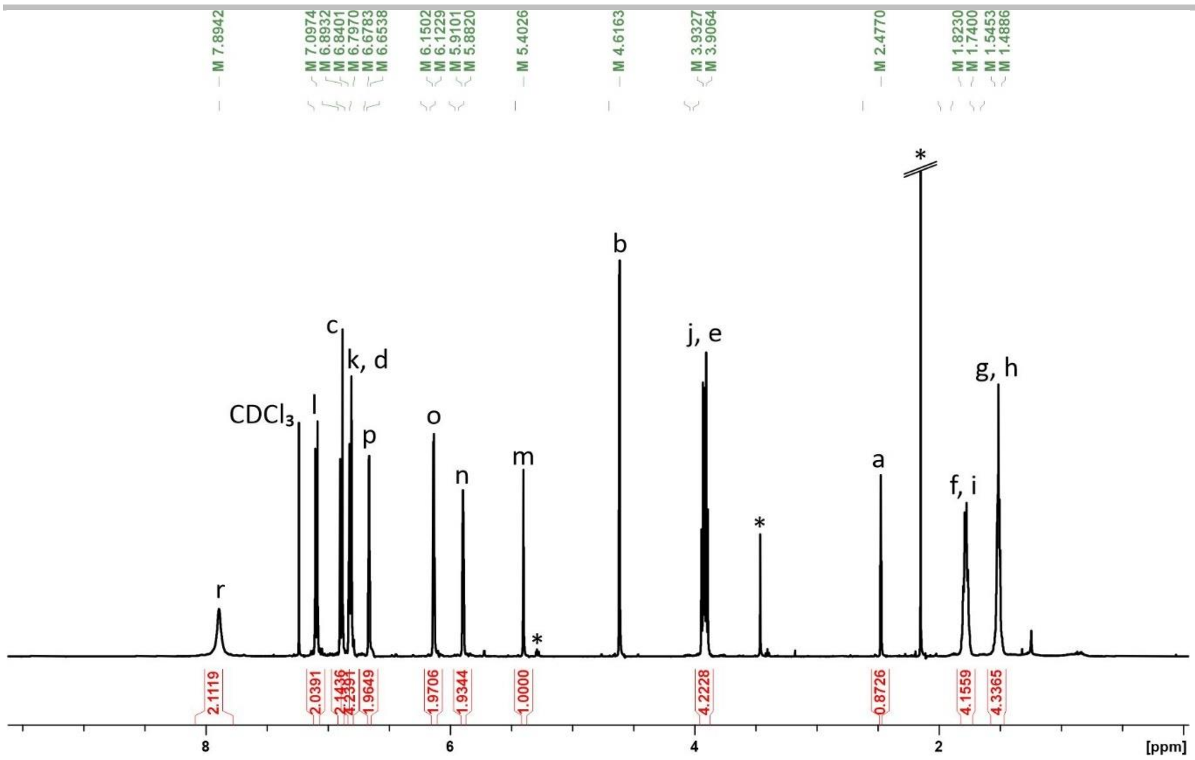

Figure S 15. The ^{1}H NMR spectrum of **8** (500 MHz, [D]chloroform, 300 K).

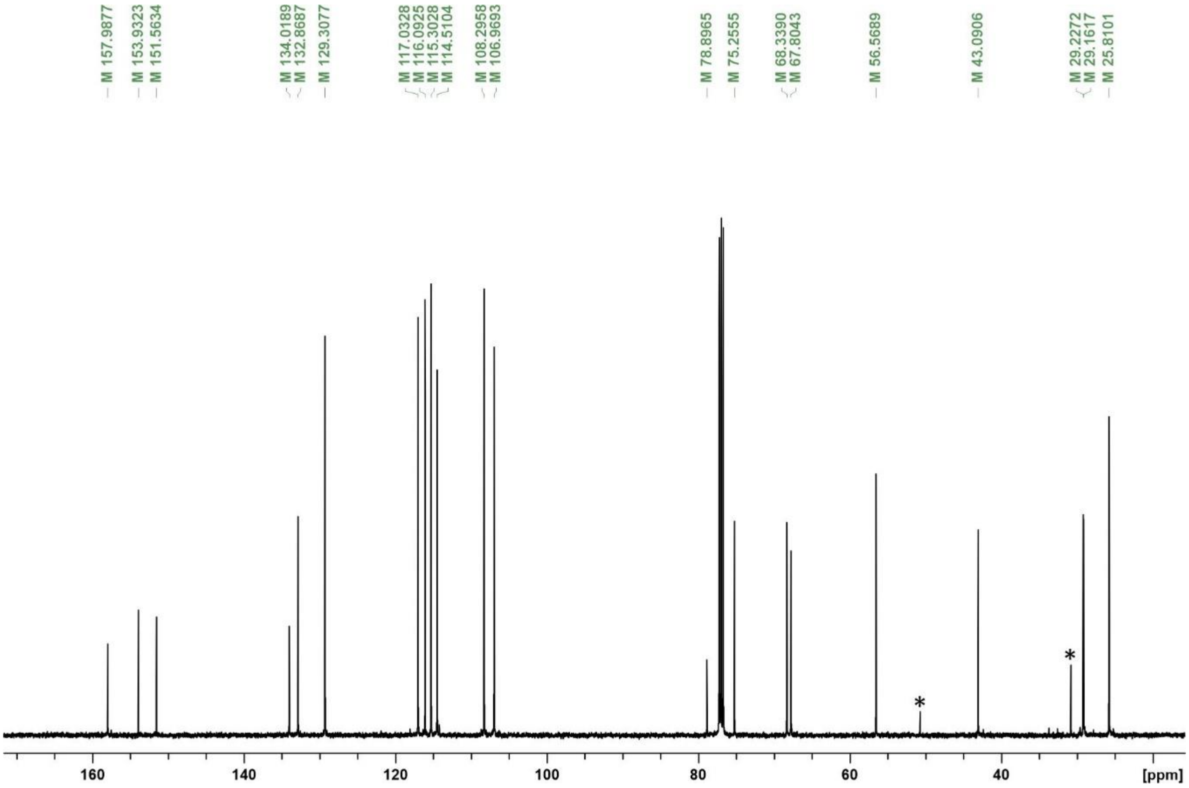

Figure S 16. The ^{1}H NMR spectrum of **8** (125 MHz, [D]chloroform, 300 K).

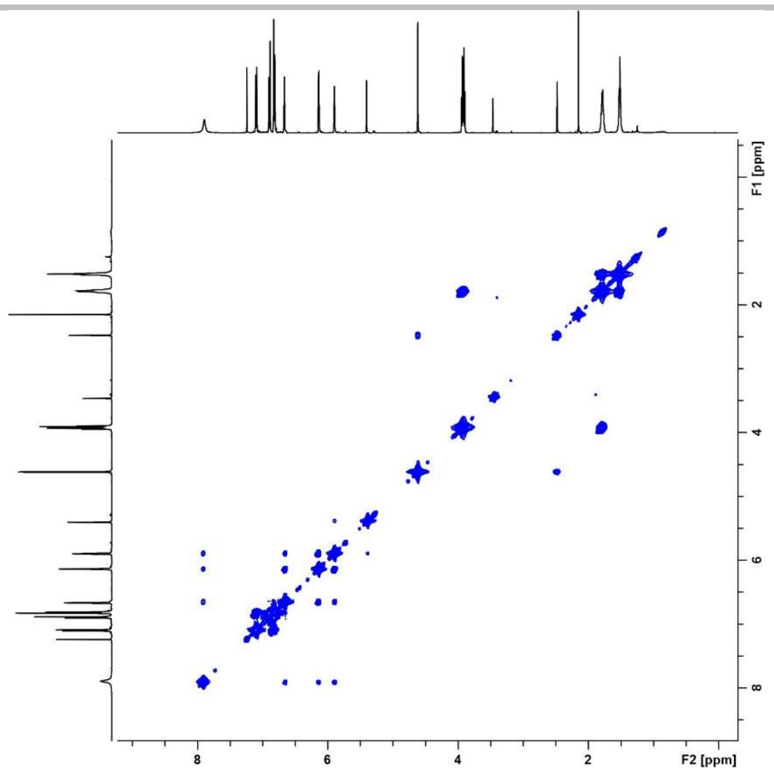

Figure S 17. The ^{1}H-^{1}H COSY NMR spectrum of **8** (500 MHz, [D]chloroform, 300 K).

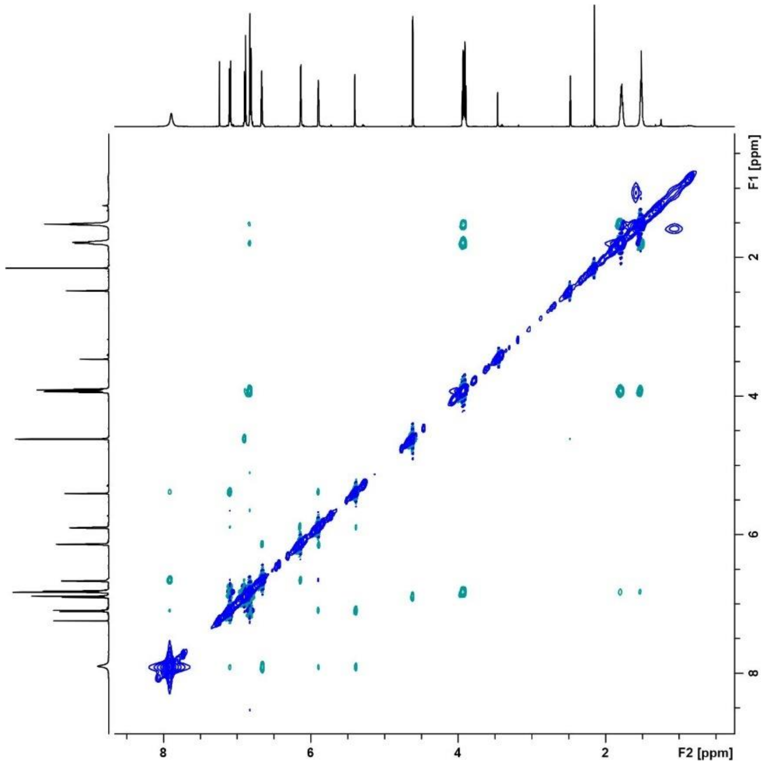

Figure S 18. The ^{1}H-^{1}H NOESY NMR spectrum of **8** (500 MHz, [D]chloroform, 300 K).

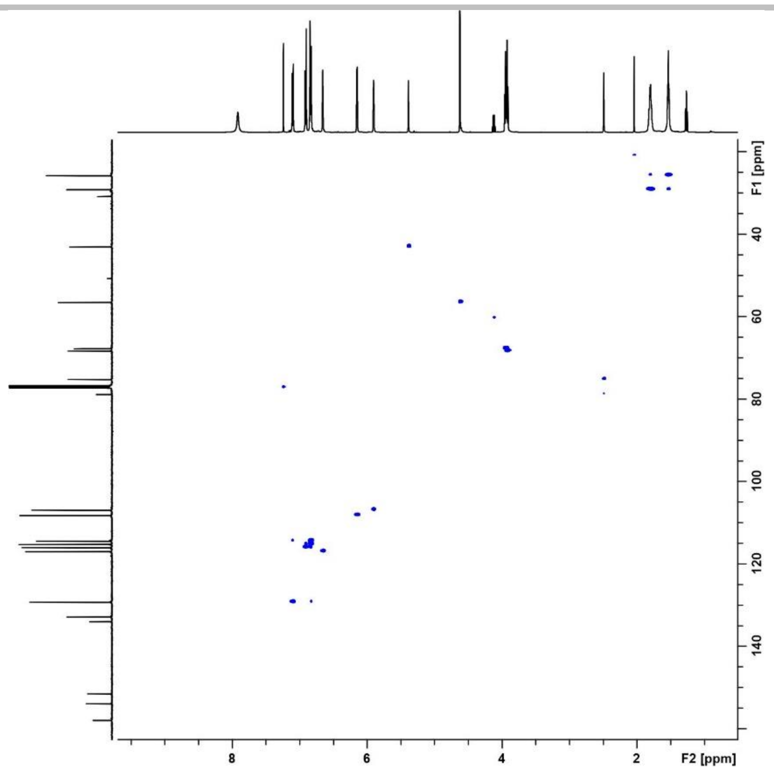

Figure S 19. The ^{1}H-^{13}C HSQC NMR spectrum of **8** (500 MHz, [D]chloroform, 300 K).

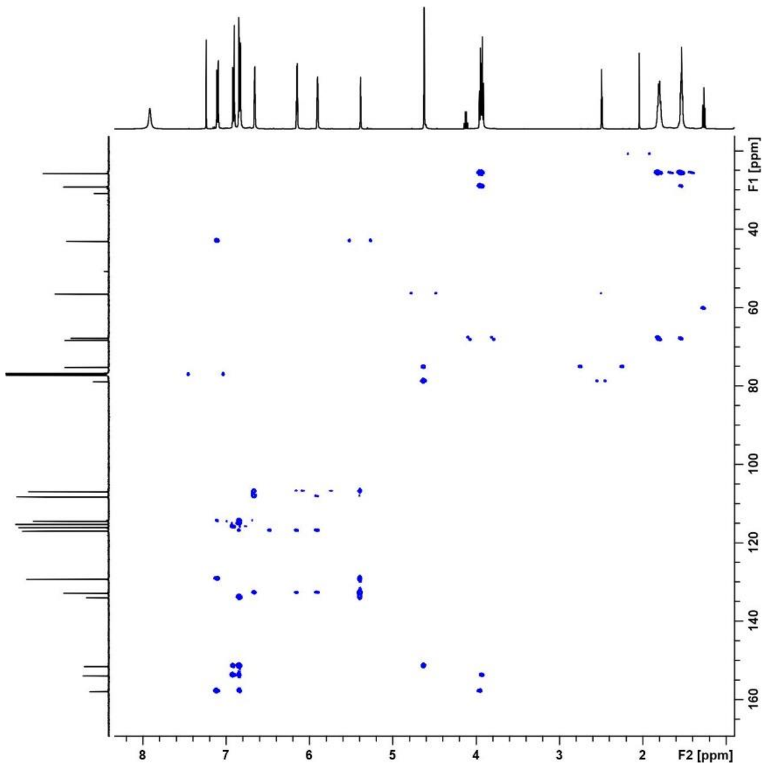

Figure S 20. The ^{1}H-^{13}C HMBC NMR spectrum of **8** (500 MHz, [D]chloroform, 300 K).

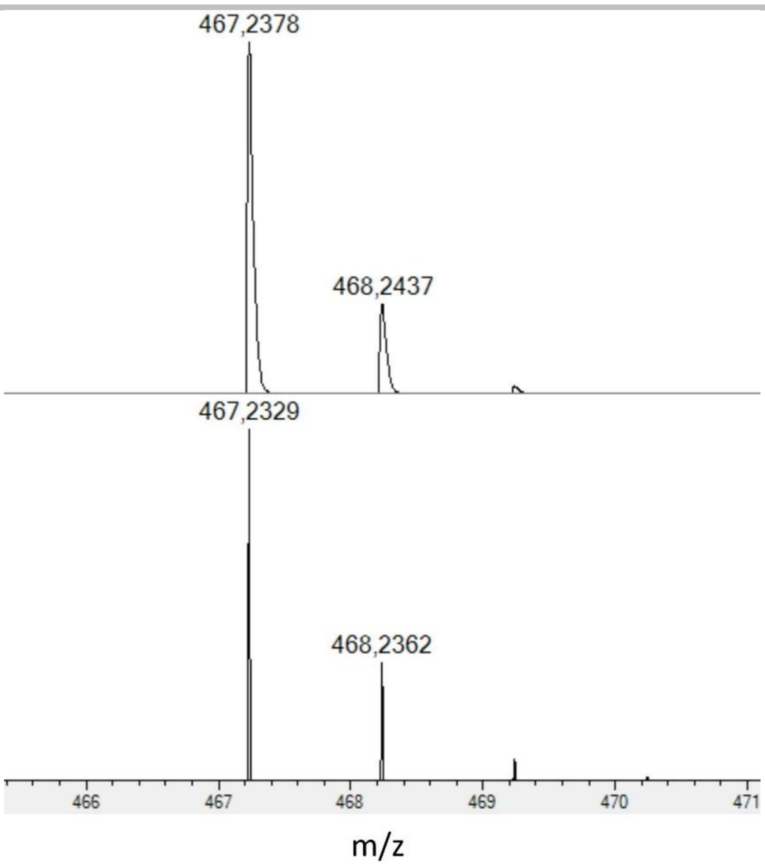

Figure S 21. The high-resolution mass spectrum of **8.** The spectrum was recorded upon the addition of DDQ to **8** in order to form dipyrrin, which improved ionisation. Top: experimental spectrum, bottom: simulated isotopic pattern.

Rotaxane synthesis

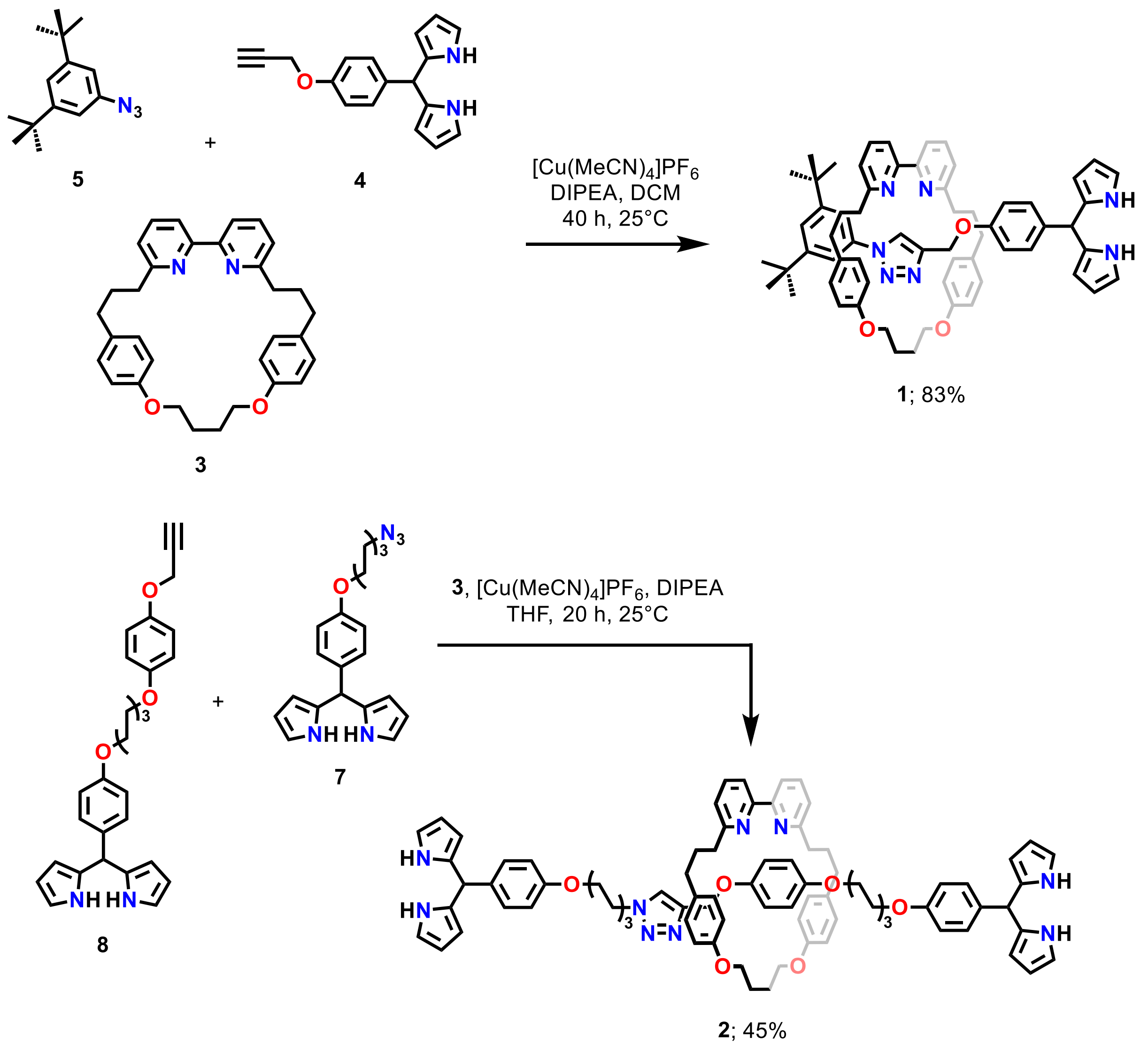

Scheme S 3. The synthesis of **1** and **2**.

Compound 1

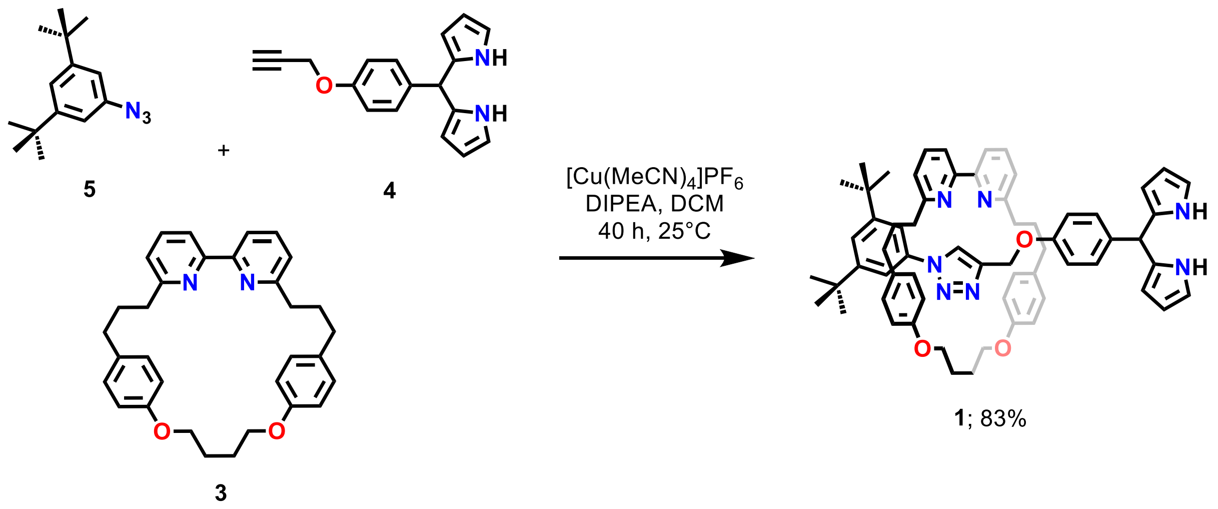

In a 10 ml vial with a screw cap, macrocycle **3** (203 mg, 425 μmol), **4** (140 mg, 507 μmol), **5** (118 mg, 510 μmol), $[Cu(CH_3CN)_4]PF_6$ (31 mg, 83 μmol), DIPEA (78 μl, 448 μmol) were dissolved in DCM (4 ml). After mixing the reagents, the solution immediately turned dark orange. The vial was sealed, and the cap was secured with a parafilm. The mixture was stirred for 40 hours at room temperature. After this time, the mixture was transferred into a separatory funnel, and DCM (50 ml) was introduced. Subsequently, aqueous ammonia solution (50 ml) and EDTA (500 mg, 1.71 mmol) were added to the solution in a separatory funnel, and upon the one-minute-long shaking, the solution turned dark. The aqueous phase was extracted with DCM (50 ml). The collected organic extracts were washed with water and brine. The aqueous phase was once more extracted with DCM (50 ml). The collected organic layers were combined and dried over anhydrous Na_2SO_4. The filtrate was collected *via* gravity filtration, and the solvent was removed under reduced pressure. The obtained dark green oil was purified *via* flash chromatography (DCM with 5-25% ethyl acetate gradient) to provide **1** (349 mg, 354 μmol, 83%) as a yellowish oil which turned dark after time.

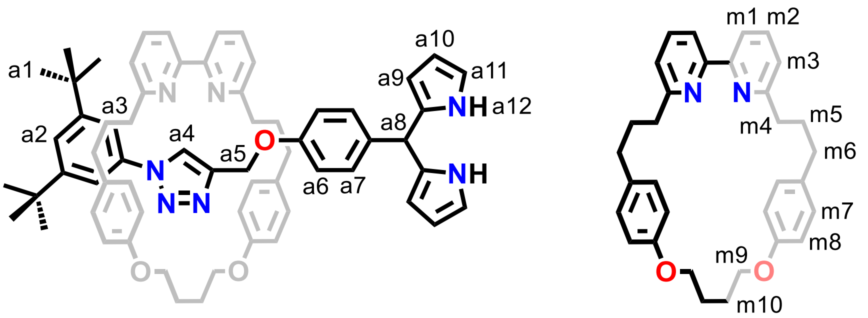

^{1}H NMR (500 MHz, [D]chloroform, 300 K, ppm): δ 9.86 (s, 1H, H_{a4}), 7.87 (b, 2H, H_{a12}), 7.56 (d, ^3J = 1.7 Hz, 2H, H_{a3}), 7.55 (t, ^3J = 7.8, 2H, H_{m2}), 7.40 (d, ^3J = 7.7, 2H, H_{m1}), 7.29 (t, ^3J = 1.7, 1H, H_{a2}), 7.02 (d, ^3J = 7.7, 2H, H_{m3}), 6.82 (d, ^3J = 8.6, 2H, H_{a7}), 6.65–6.63 (m, 2H, H_{a11}), 6.54 (d, ^3J = 8.8, 4H, H_{m8}), 6.51 (d, ^3J = 8.8, 4H, H_{m7}), 6.42 (d, ^3J = 8.6, 2H, H_{a6}), 6.15–6.12 (m, 2H, H_{a10}), 5.88–5.85 (m, 2H, H_{a9}), 5.29 (s, 1H, H_{a8}), 4.33–4.24 (m, 4H, H_{m9}), 4.22 (s, 2H, H_{a5}), 2.51–2.44 (m, 2H, H_{m6}), 2.36–2.27 (m, 6H, H_{m4}, H_{m6}), 2.15–2.05 (m, 4H, H_{m10}), 1.69–1.52 (m, 4H, H_{m5}), 1.20 (s, 18H, H_{a1}); **^{13}C NMR** (125 MHz, [D]chloroform, 300 K, ppm): δ 171.1, 163.0, 157.5, 157.4, 157.2, 151.6, 143.0, 137.1, 136.6, 133.1, 133.0, 132.6, 128.6, 124.4, 121.5, 121.3, 119.8, 116.9, 115.0, 114.80, 114.7, 108.3, 106.8, 66.7, 61.1, 43.1, 36.9, 35.00, 34.97, 31.7, 31.3, 24.9; **HR–MALDI–MS** (*m/z*): $[M+H]^+$calcd. for $C_{64}H_{72}N_7O_3^+$, 986.5691; found, 986.5571.

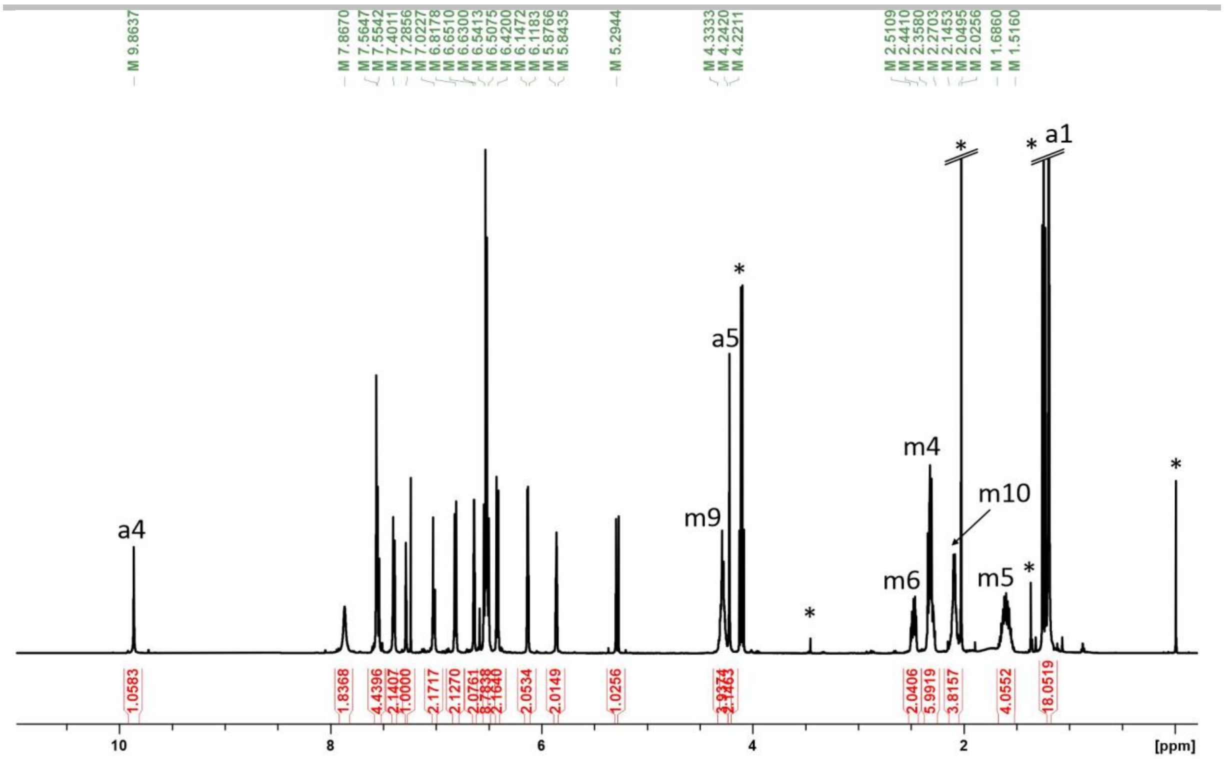

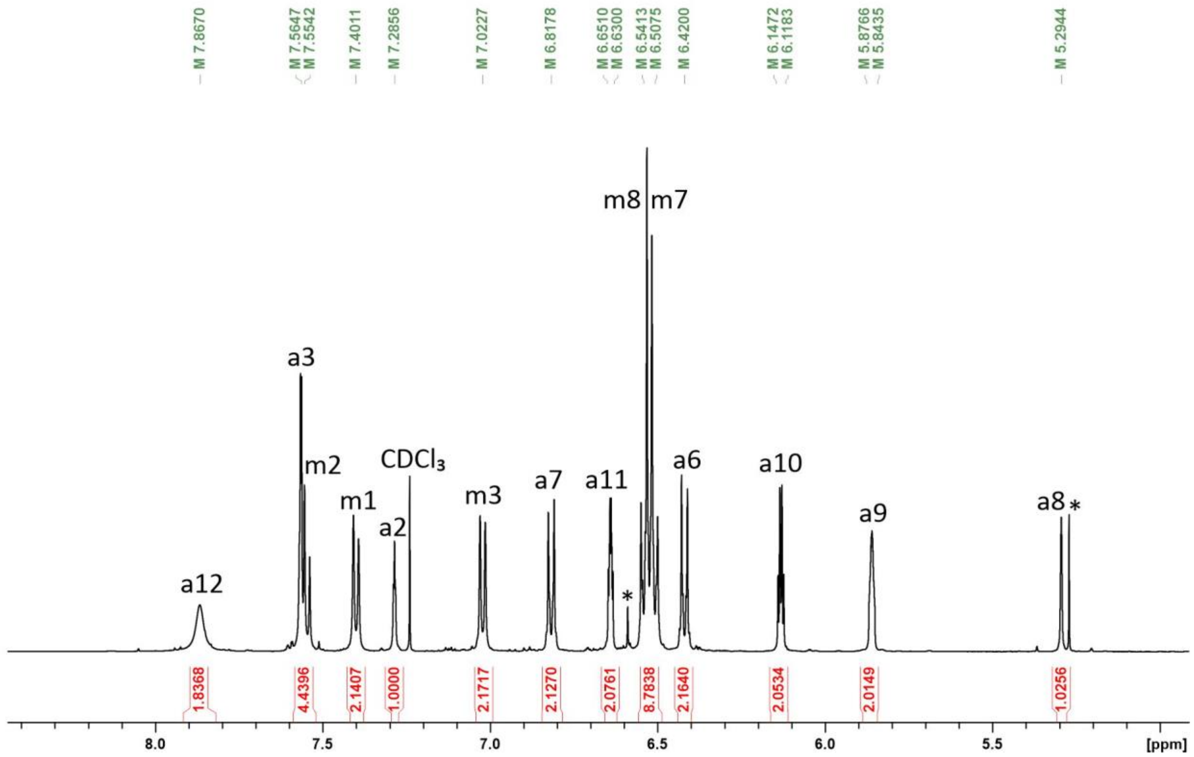

Figure S 22. The ^{1}H NMR spectrum of **1** (500 MHz, [D]chloroform, 300 K).

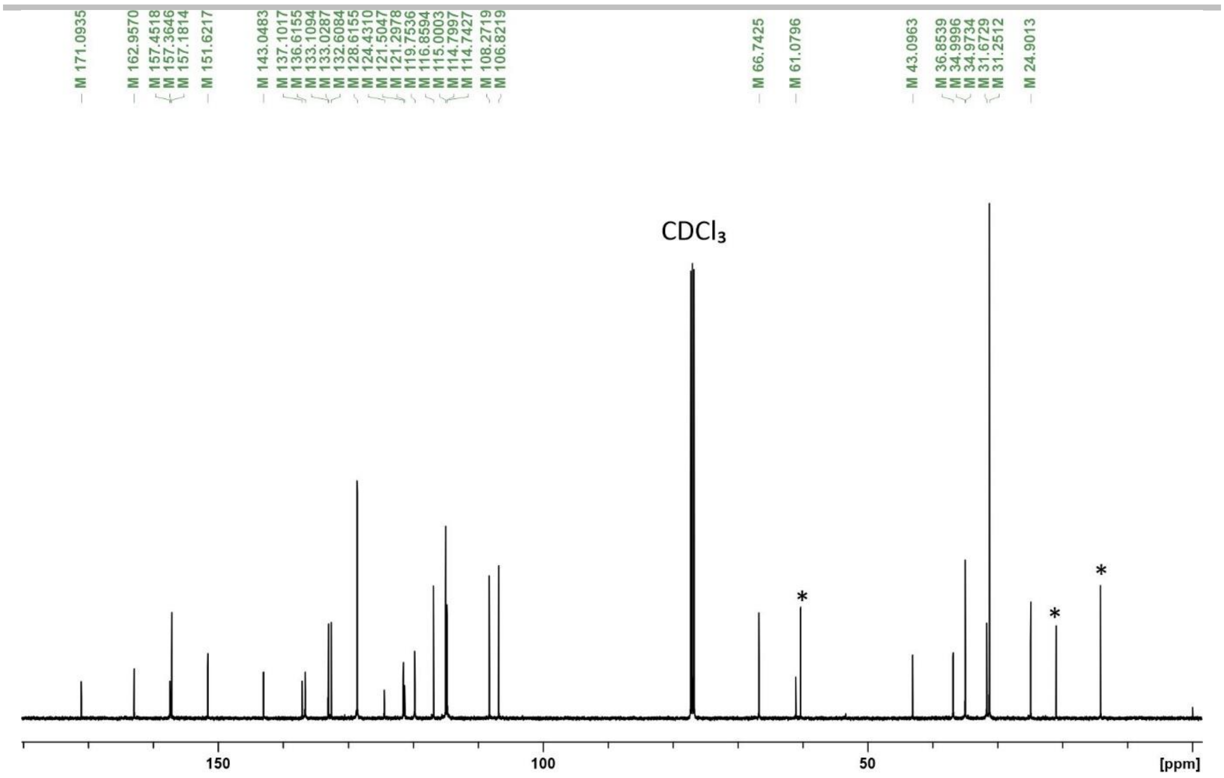

Figure S 23. The ^{13}C NMR spectrum of **1** (125 MHz, [D]chloroform, 300 K).

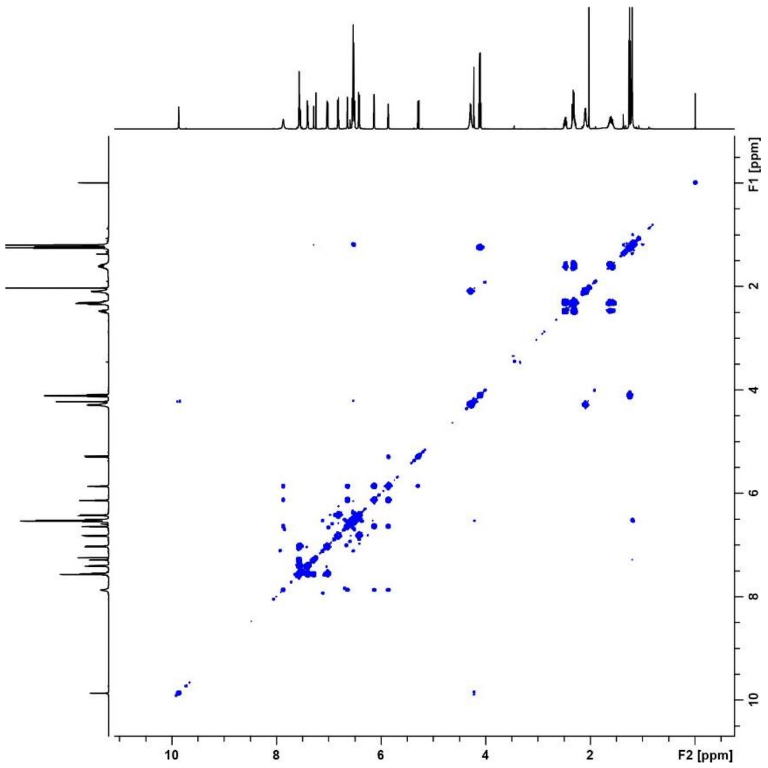

Figure S 24. The ^{1}H-^{1}H COSY NMR spectrum of **1** (500 MHz, [D]chloroform, 300 K).

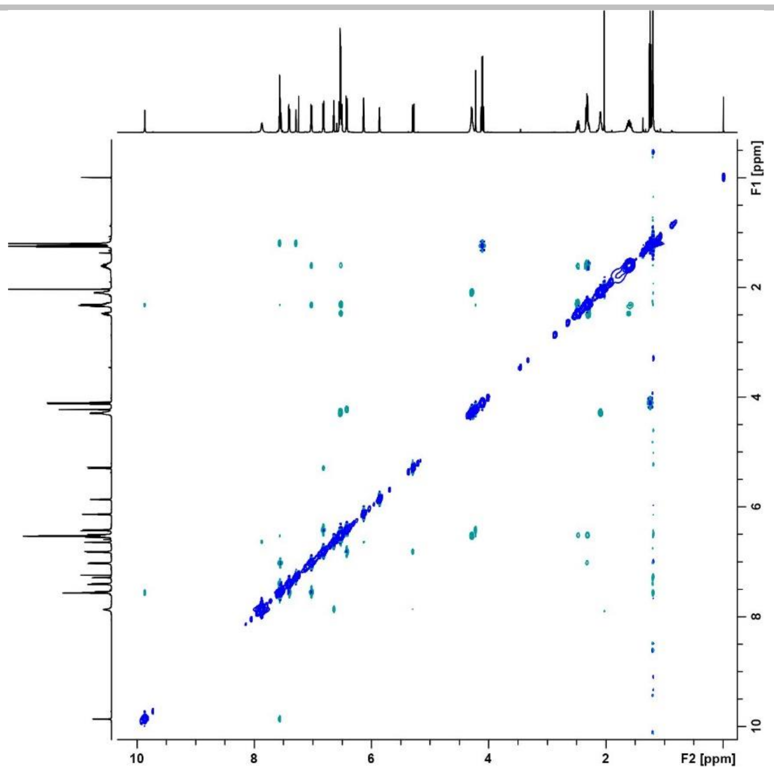

Figure S 25. The ^{1}H-^{1}H NOESY NMR spectrum of **1** (500 MHz, [D]chloroform, 300 K).

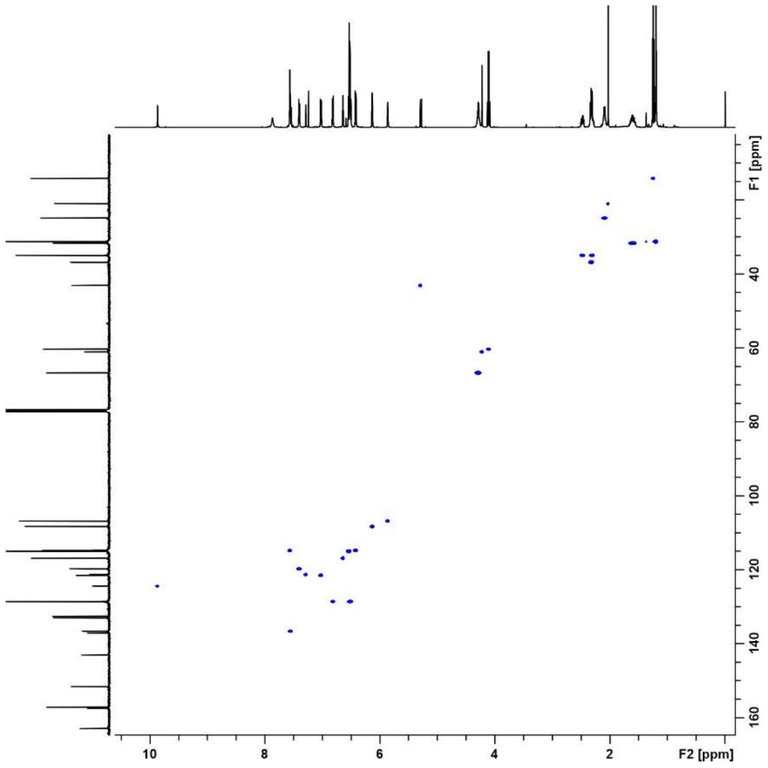

Figure S 26. The ^{1}H-^{13}C HSQC NMR spectrum of **1** (500 MHz, [D]chloroform, 300 K).

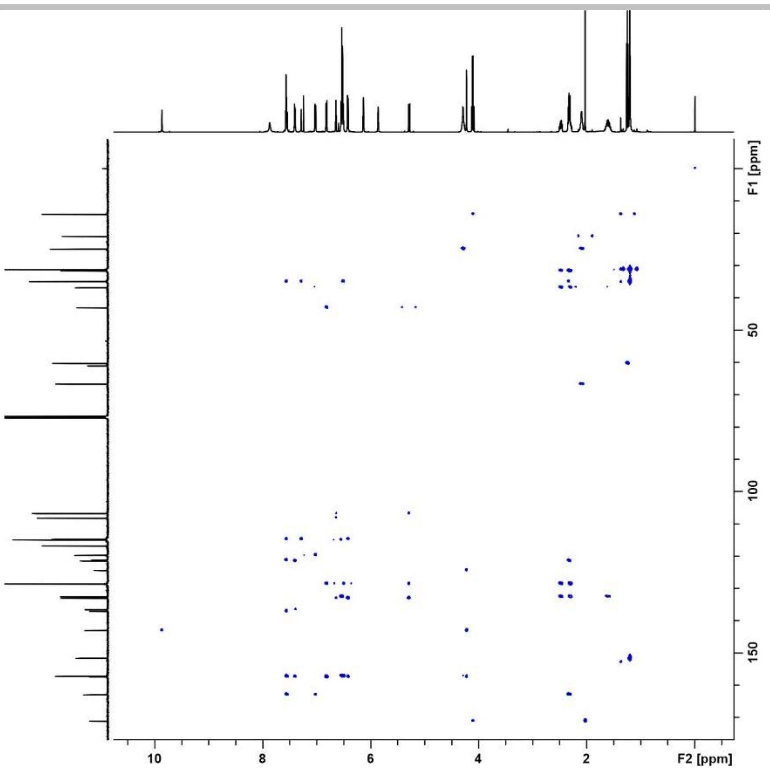

Figure S 27. The ^{1}H-^{13}C HMBC NMR spectrum of **1** (500 MHz, [D]chloroform, 300 K).

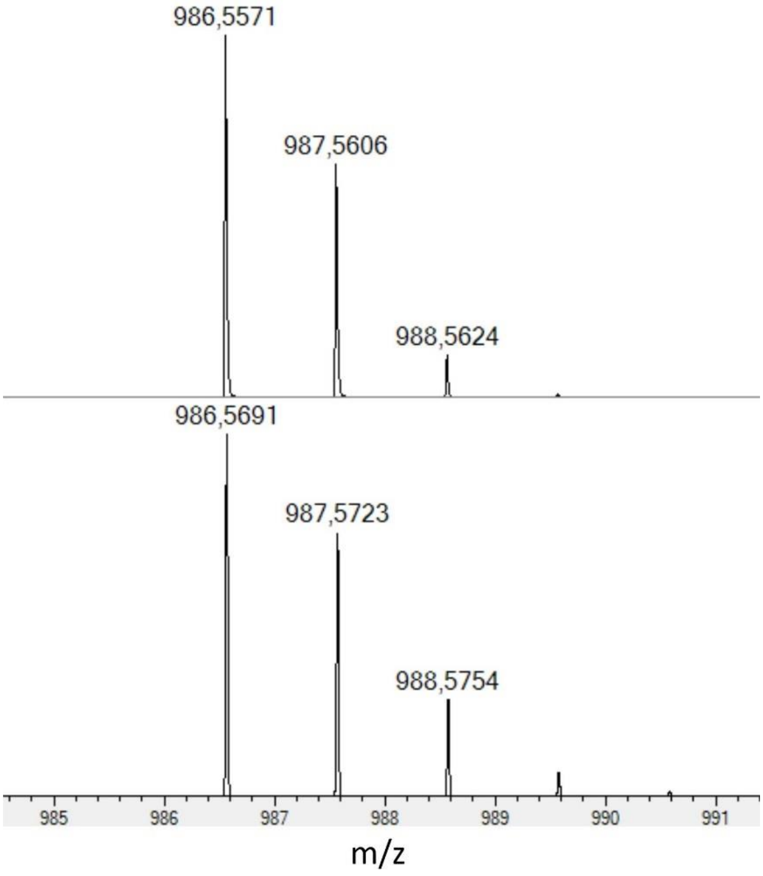

Figure S 28. The high-resolution mass spectrum of **1** (MALDI MS). Top: experimental spectrum, bottom: simulated isotopic pattern.

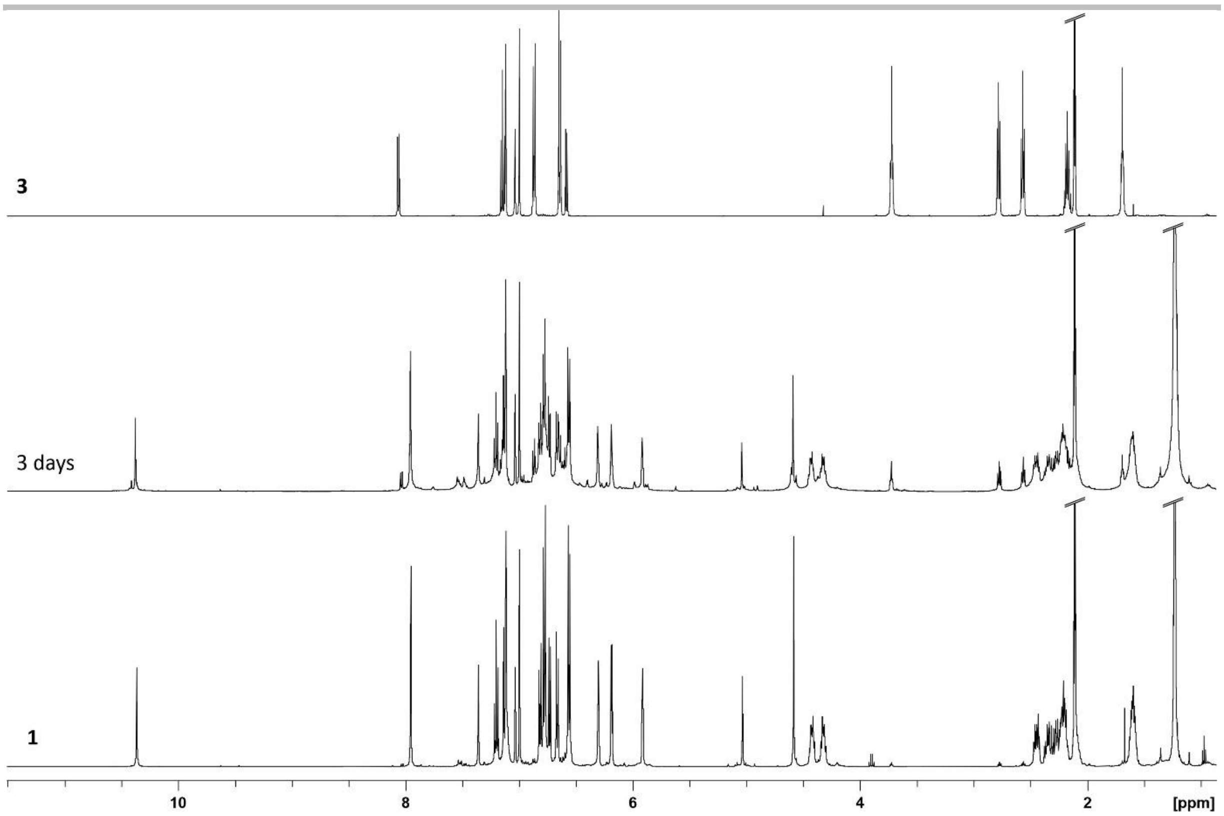

Figure S 29. The stability of **1** at elevated temperature. The ^{1}H NMR spectra (500 MHz, [D$_8$]toluene, 300 K) were recorded for the solution of **1** in toluene heating at 100°C over 3 days. The presence of the **3** macrocycle indicated a partial dethreading.

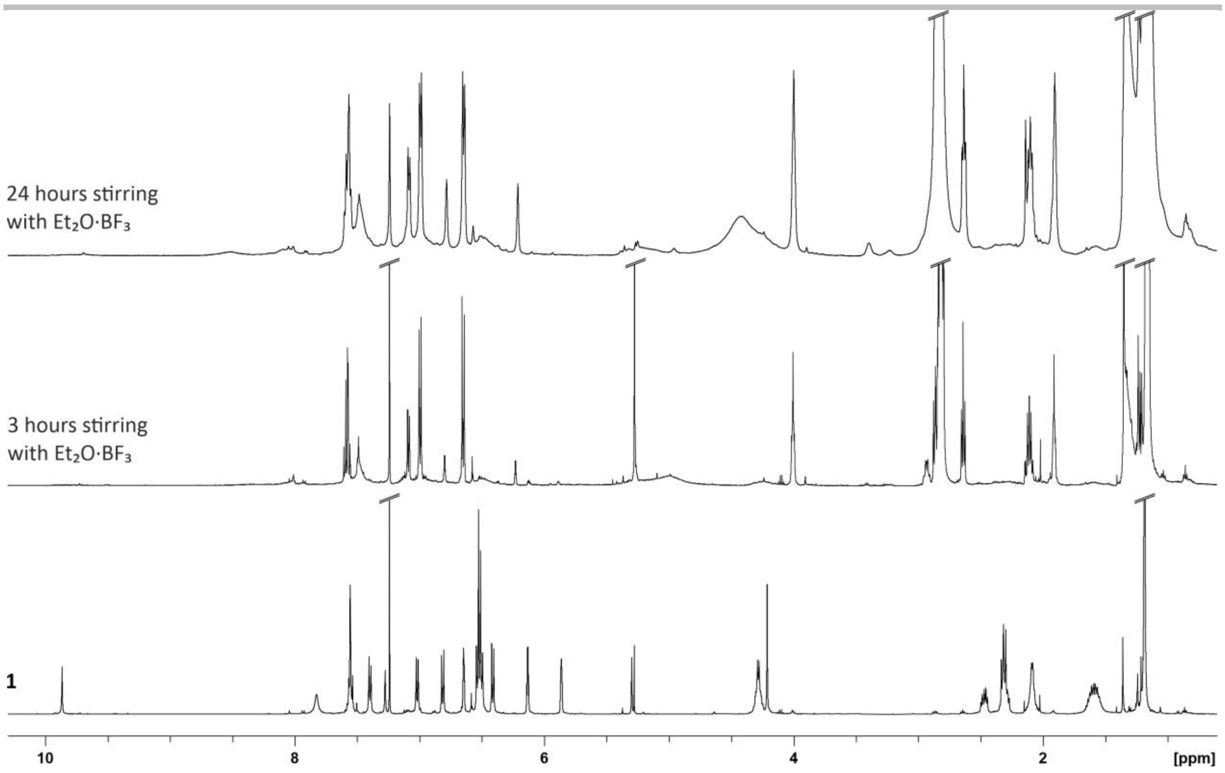

Figure S 30. The stability of **1** in the presence of $Et_2O{\cdot}BF_3$. The solution of **1** in deacidified DCM was stirred with $Et_2O{\cdot}BF_3$ (2.8 equiv.) for 3 hours and 24 hours. Both reactions were quenched by the addition of excess TEA, and the ^1H NMR spectra were recorded (500 MHz, [D]chloroform, 300 K). The formation of black precipitate was observed in both cases. The macrocycle **3** was detected in both samples.

Compound 2

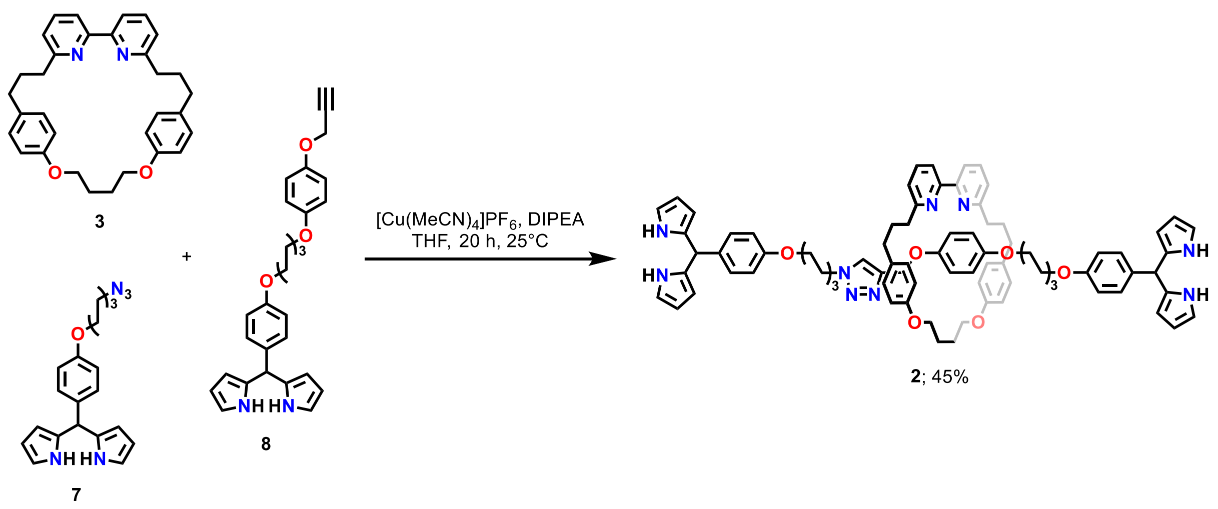

In a 10 ml vial with a screw cap, macrocycle **3** (60mg, 126 μmol), **7** (48 mg, 132 μmol), **8** (59 mg, 126 μmol), $[Cu(CH_3CN)_4]PF_6$ (4.6 mg, 12 μmol), DIPEA (23.5 μl, 135 μmol) were dissolved in THF (1.5 ml). After mixing the reagents, the solution immediately turned dark orange. The vial was sealed, and the cap was secured with a parafilm. The mixture was stirred for 20 hours at room temperature. After this time, the mixture was transferred into a separatory funnel, and DCM (30 ml) was introduced. Subsequently, aqueous ammonia solution (30 ml) and EDTA (300 mg, 1.03 mmol) were added to the solution in a separatory funnel, and upon the one-minute-long shaking, the solution turned dark. The aqueous phase was extracted with DCM (30 ml). The collected organic extracts were washed with water and brine. The aqueous phase was once more extracted with DCM (30 ml). The collected organic layers were combined and dried over anhydrous Na_2SO_4. The filtrate was collected *via* gravity filtration, and the solvent was removed under reduced pressure. The obtained dark green oil was purified *via* flash chromatography (DCM with 0-20% ethyl acetate gradient) to provide **2** (74 mg, 56.7 μmol, 45%) as a yellowish oil which turned dark after time.

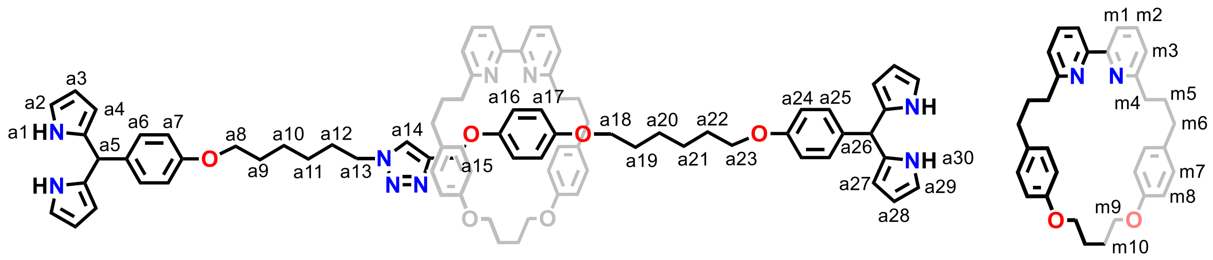

^{1}H NMR (500 MHz, [D]chloroform, 300 K, ppm): δ 8.16 (s, 1H, H_{a14}), 7.91 (s, 4H, H_{a1}, H_{a30}), 7.58 (t, ^3J = 7.7 Hz, 2H, H_{m2}), 7.36 (t, ^3J = 7.7 Hz, 2H, H_{m2}), 7.08 (overlapped d, 4H, H_{a6}, H_{a25}, H_{m3}), 6.81–6.71 (overlapped m, 12H, H_{a7}, H_{a16}, H_{a17}, H_{a24}, H_{m7}), 6.67–6.65 (m, 4H, H_{a2}, H_{a29}), 6.63 (d, ^3J = 7.7 Hz, 4H, H_{m8}), 6.15 (m, 4H, H_{a3}, H_{a28}), 5.91–5.88 (m, 4H, H_{a4}, H_{a27}), 5.404 (s, 1H, H_{a5}, H_{a26}), 5.397 (s, 1H, H_{a5}), 4.90 (s, 2H, H_{a15}), 4.24–4.17 (m, 2H, H_{m9}), 4.02– 3.95 (m, 2H, H_{m9}), 3.85 (t, ^3J = 6.5 Hz, 2H, H_{a18} or H_{a23}), 3.80 (t, ^3J = 6.5 Hz, 2H, H_{a18} or H_{a23}), 3.65(t, ^3J = 6.5 Hz, 2H, H_{a8}), 3.29 (t, ^3J = 6.5 Hz, 2H, H_{a13}), 2.60–2.44 (m, 8H, H_{m4}, H_{m6}), 2.07–1.98 (m, 2H, H_{m10}), 1.91–1.82 (m, 2H, H_{m10}), 1.82–1.63 (m, 8H, H_{a19}, H_{a22}), 1.43–1.31 (m, 6H, H_{a9}, H_{a20}, H_{21}), 0.95–0.86 (m, 2H, H_{a10}), 0.86–0.76 (m, 2H, H_{a12}), 0.69–0.61 (m, 2H, H_{a11}); **^{13}C NMR** (125 MHz, [D]chloroform, 300 K, ppm): δ 162.5, 158.09, 158.06, 157.7, 157.4, 153.4, 152.5, 142.7, 136.7, 133.9, 133.8, 133.3, 132.9, 129.3, 124.2, 121.4, 120.2, 117.0, 116.7, 115.5, 115.3, 114.9, 114.7, 114.6, 114.5, 108.4, 107.0, 68.4, 67.8, 67.6, 66.5, 62.3, 49.4, 43.2, 36.9, 34.6, 31.9, 29.2, 29.1, 28.8, 28.7, 25.84, 25.76, 25.0, 24.8; **HR–MALDI–MS** (*m/z*): ***2*** *was oxidised to bis-dipyrrin with DDQ to record the MS spectrum*, $[M+H]^+$ calcd. for $C_{83}H_{88}N_9O_6^+$, 1306.6852; found, 1306.6891.

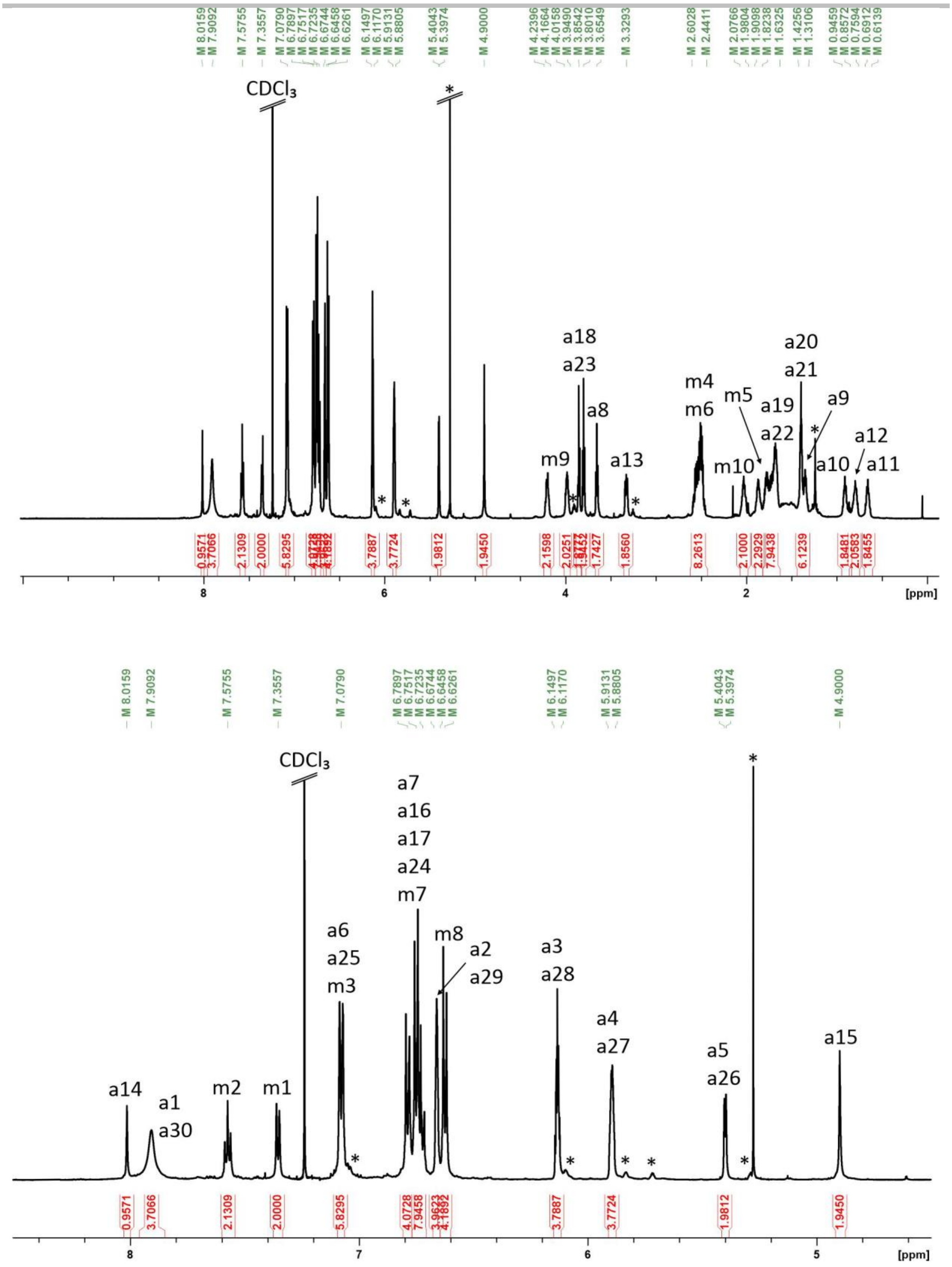

Figure S 31. The ^{1}H NMR spectrum of **2** (500 MHz, [D]chloroform, 300 K).

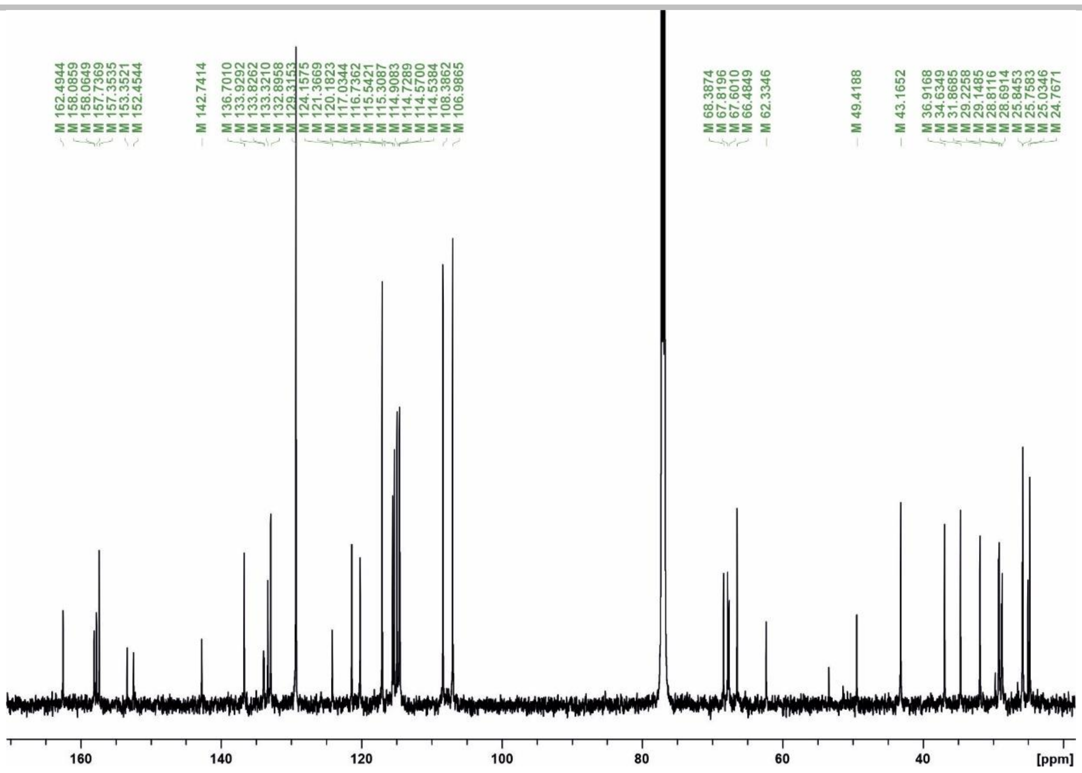

Figure S 32. The ^{13}C NMR spectrum of **2** (125 MHz, [D]chloroform, 300 K).

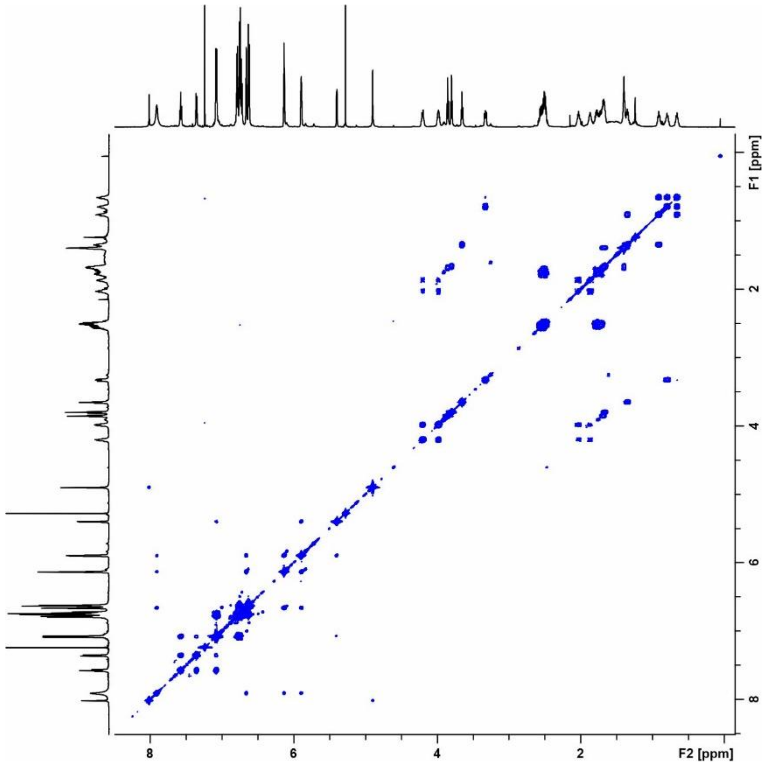

Figure S 33. The ^{1}H-^{1}H COSY NMR spectrum of **2** (500 MHz, [D]chloroform, 300 K).

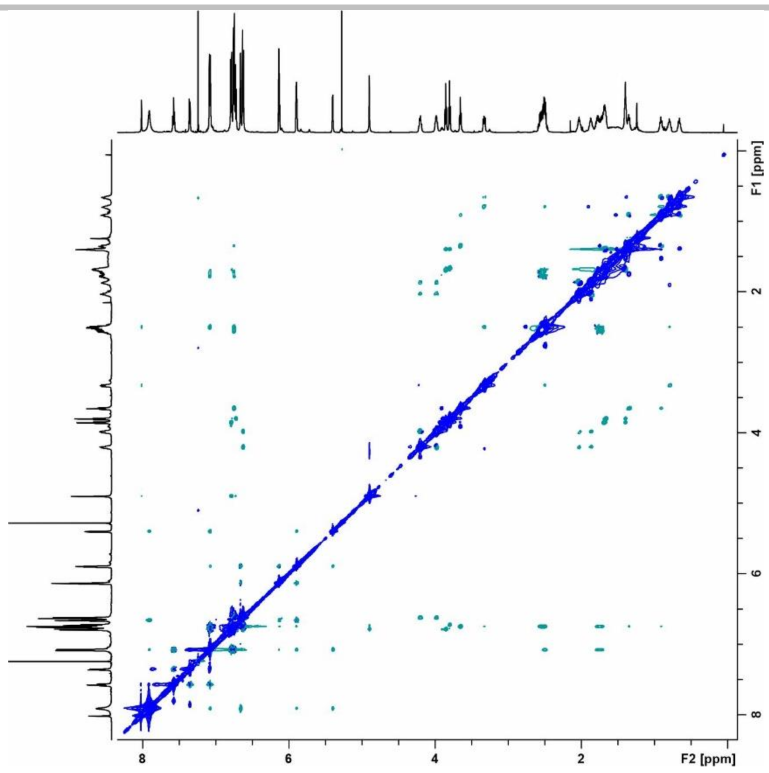

Figure S 34. The ^{1}H-^{1}H NOESY NMR spectrum of **2** (500 MHz, [D]chloroform, 300 K).

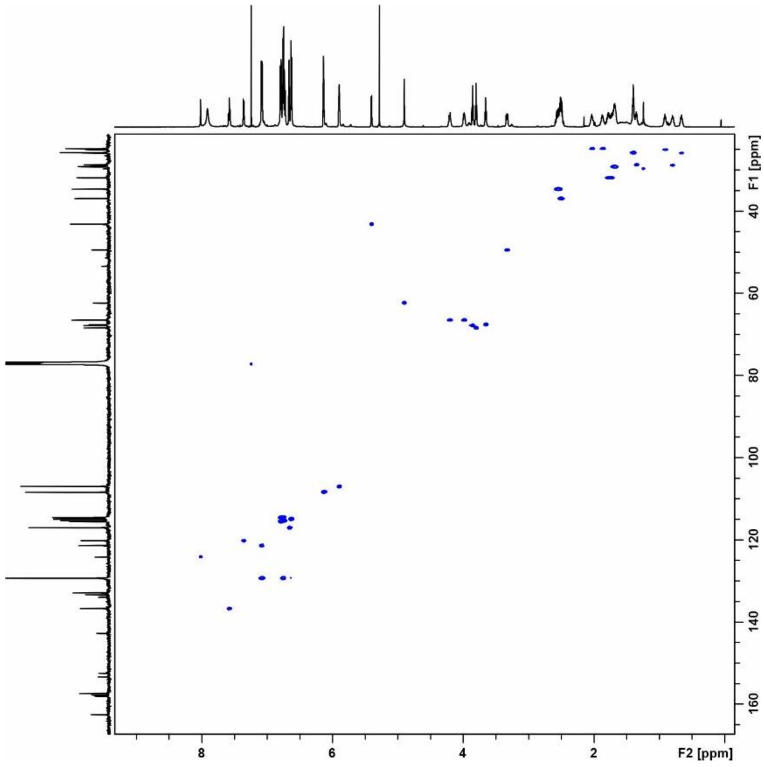

Figure S 35. The ^{1}H-^{13}C HSQC NMR spectrum of **2** (500 MHz, [D]chloroform, 300 K).

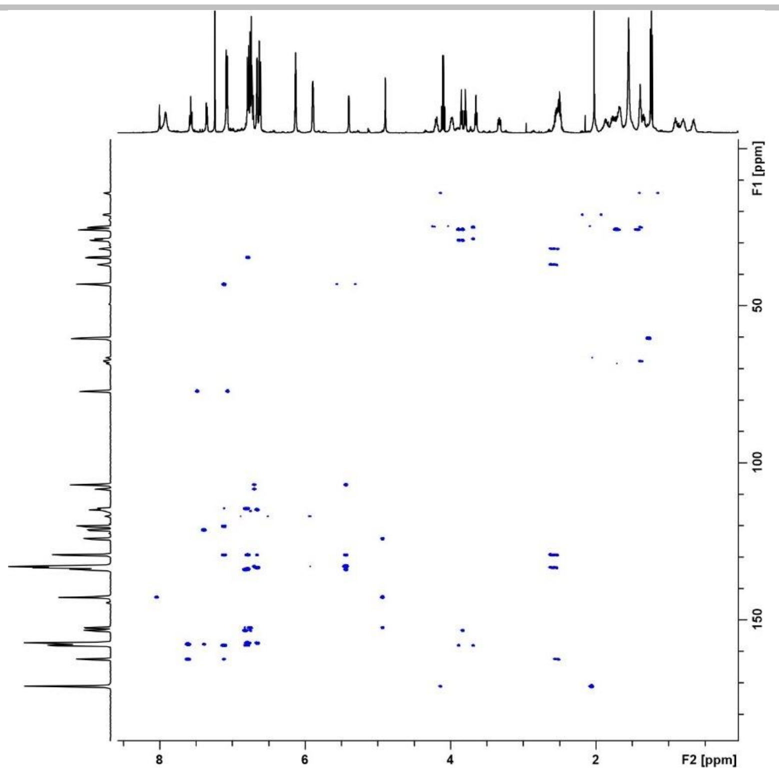

Figure S 36. The ^{1}H-^{13}C HMBC spectrum of **2** (500 MHz, [D]chloroform, 300 K).

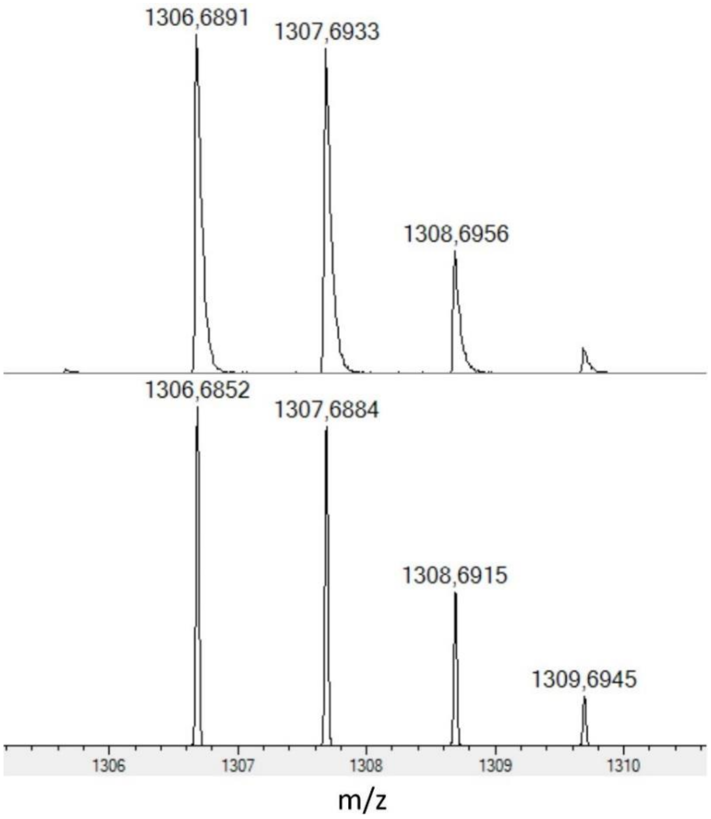

Figure S 37. The high-resolution MALDI mass spectrum of **2**. The spectrum was recorded upon the addition of DDQ to **2** in order to form bis-dipyrrin, which improved ionisation. Top: experimental spectrum, bottom: simulated isotopic pattern.

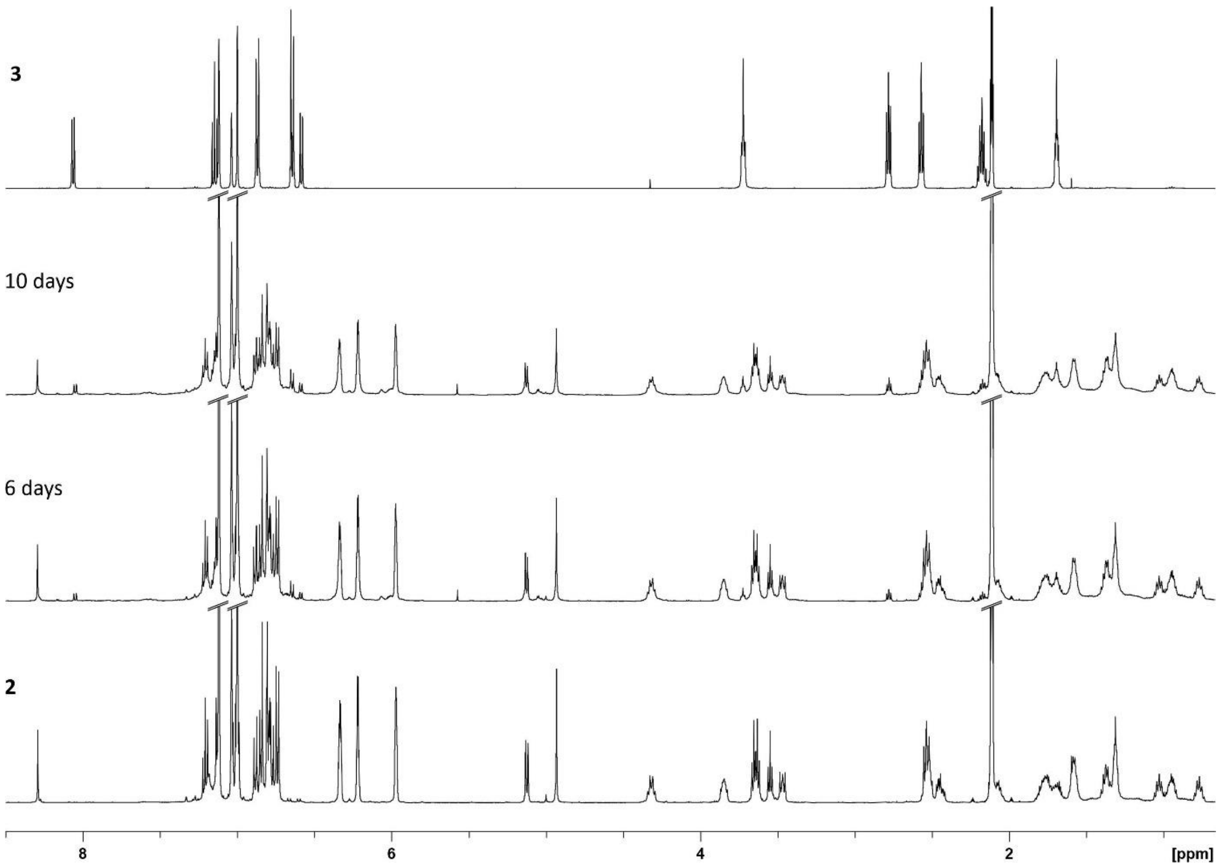

Figure S 38. The stability of **2** at elevated temperature. The ^{1}H NMR spectra (500 MHz, [D_8]toluene, 300 K) were recorded for the solution of **2** in toluene heating at 100°C over six and ten days. The presence of the **3** macrocycle indicated a partial dethreading.

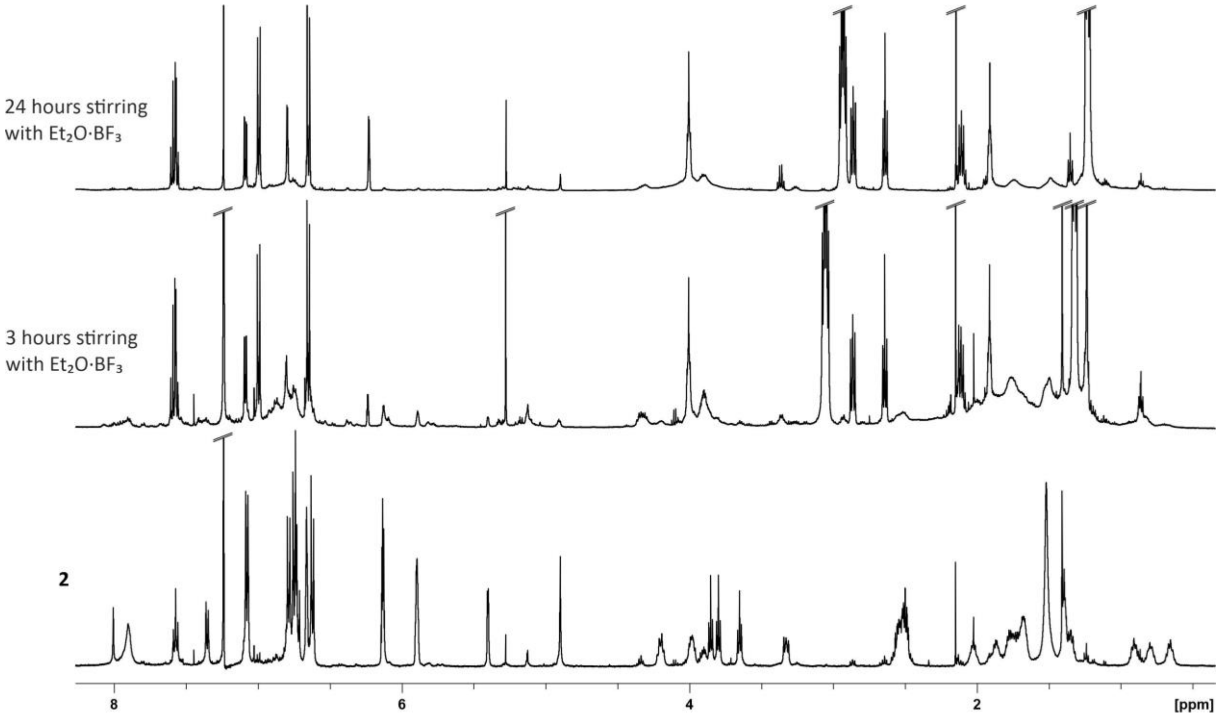

Figure S 39. The stability of **2** in the presence of $Et_2O{\cdot}BF_3$. The solution of **2** in deacidified DCM was stirred with $Et_2O{\cdot}BF_3$ (2.6 equiv.) for 3 hours and 24 hours. Both reactions were quenched by the addition of excess TEA, and the ^{1}H NMR spectra were recorded (500 MHz, [D]chloroform, 300 K). The formation of black precipitate was observed in both cases. The macrocycle **3** was detected in both samples.

Synthesis of porphyrinoid-based interlocked molecules

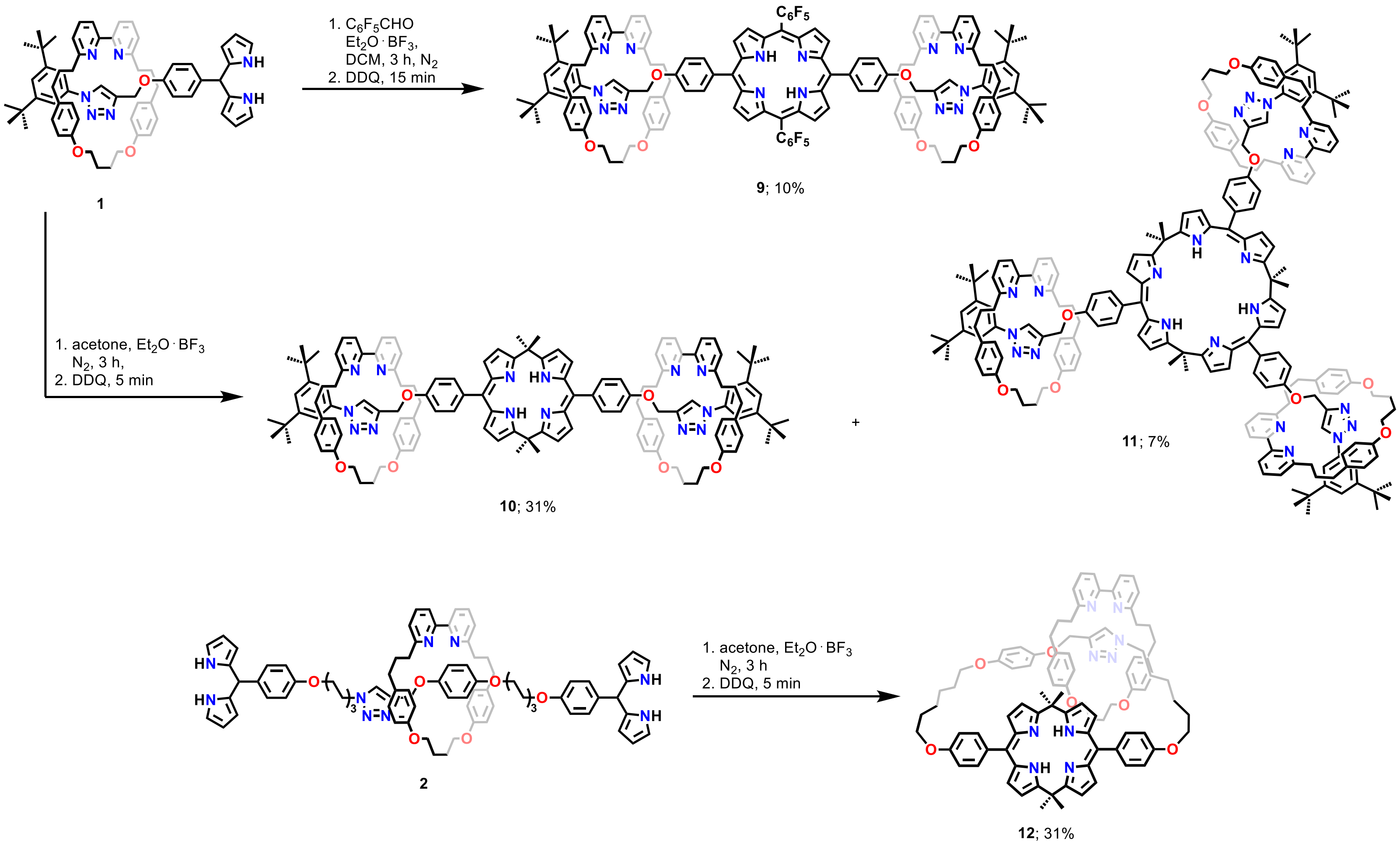

Scheme S 4. Synthesis of **9**-**12**.

Compound 9

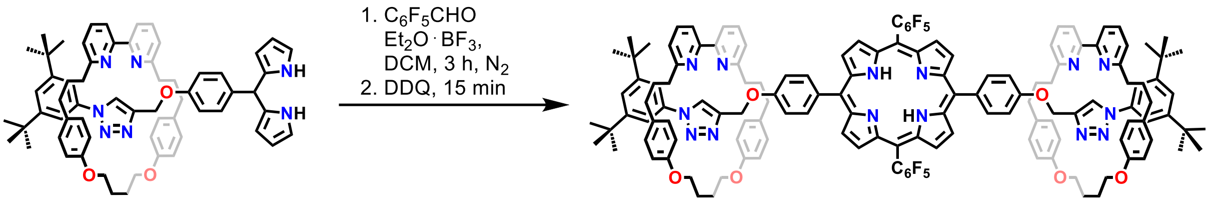

1 **9**; 10%

In a 25 ml round bottom flask, **1** (60 mg, 61 µmol) and DCM (6 ml) were introduced. DCM was previously purified by passing through a column filled with alkaline aluminum oxide. The mixture was flushed via nitrogen bubbling under a sealed flask for 10 minutes. Then $Et_2O{\cdot}BF_3$ (15 µl, 122 µmol) was added via the syringe. The flask was covered with aluminium foil to protect the reaction from light. The mixture was then stirred for 3 h under a nitrogen atmosphere. After this time, the septum was removed, DDQ (49 mg, 214 µmol) was introduced, and the reaction was carried out for an additional 15 minutes. Then, the acid was quenched by TEA (0.5 ml), and the mixture was passed through a short column with deactivated aluminum oxide. Residues on the column were washed out with ethyl acetate. The solvent was removed under reduced pressure. The dark oil was purified *via* flash chromatography (hexane with 5-20% ethyl acetate gradient) to provide **9** (7.4 mg, 3.19 µmol, 10%) as a dark violet solid.

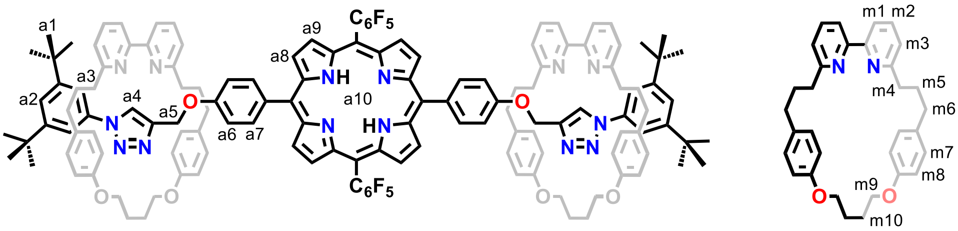

^{1}H NMR (600 MHz, [D]chloroform, 300 K, ppm): δ 9.86 (s, 2H, H_{a4}), 8.89 (d, ^{3}J = 4.6 Hz, 4H, H_{a8}), 8.77 (d, ^{3}J = 4.6 Hz, 4H, H_{a9}), 7.79 (d, ^{3}J = 8.3 Hz, 4H, H_{a7}), 7.66 (d, ^{3}J = 1.6 Hz, 4H, H_{a3}), 7.54 (t, ^{3}J = 7.8 Hz, 4H, H_{m2}), 7.42 (d, ^{3}J = 7.5 Hz, 4H, H_{m1}), 7.35 (t, ^{3}J = 1.6 Hz, 2H, H_{a2}), 7.11 (d, ^{3}J = 7.7, 4H, H_{m3}), 6.86 (d, ^{3}J = 8.4 Hz, 4H, H_{a6}), 6.75–6.71 (m, 16H, H_{m7}, H_{m8}), 4.46 (s, 4H, H_{a5}), 4.44–4.30 (m, 8H, H_{m9}), 2.69–2.60 (m, 4H, H_{m6}), 2.60–2.47 (m, 8H, H_{m4}), 2.47–2.38 (m, 4H, H_{m6}), 2.21–2.11 (m, 8H, H_{m10}), 1.86–1.73 (m, 8H, H_{m5}), 1.25 (s, 36H, H_{a1}), –2.83 (s, 2H, H_{a10}); **^{13}C NMR** (151 MHz, [D]chloroform, 300 K, ppm): δ 163.1, 158.6, 157.7, 157.4, 152.0, 143.5, 137.2, 136.7, 135.0, 133.0, 132.6, 128.9, 124.3, 121.8, 121.6, 120.0, 115.2, 114.9, 113.3, 66.9, 61.5, 37.2, 35.2, 35.1, 32.1, 31.3, 29.7, 25.0; **^{19}F NMR** (470 MHz, [D]chloroform, 300 K, ppm): δ –136.7, –155.5, –162.0; **HR–ESI–MS** (*m/z*): $[M+2H]^{2+}$ calcd. for $C_{142}H_{136}F_{10}N_{14}O_6^{2+}$, 1162.0314; found, 1162.0399.

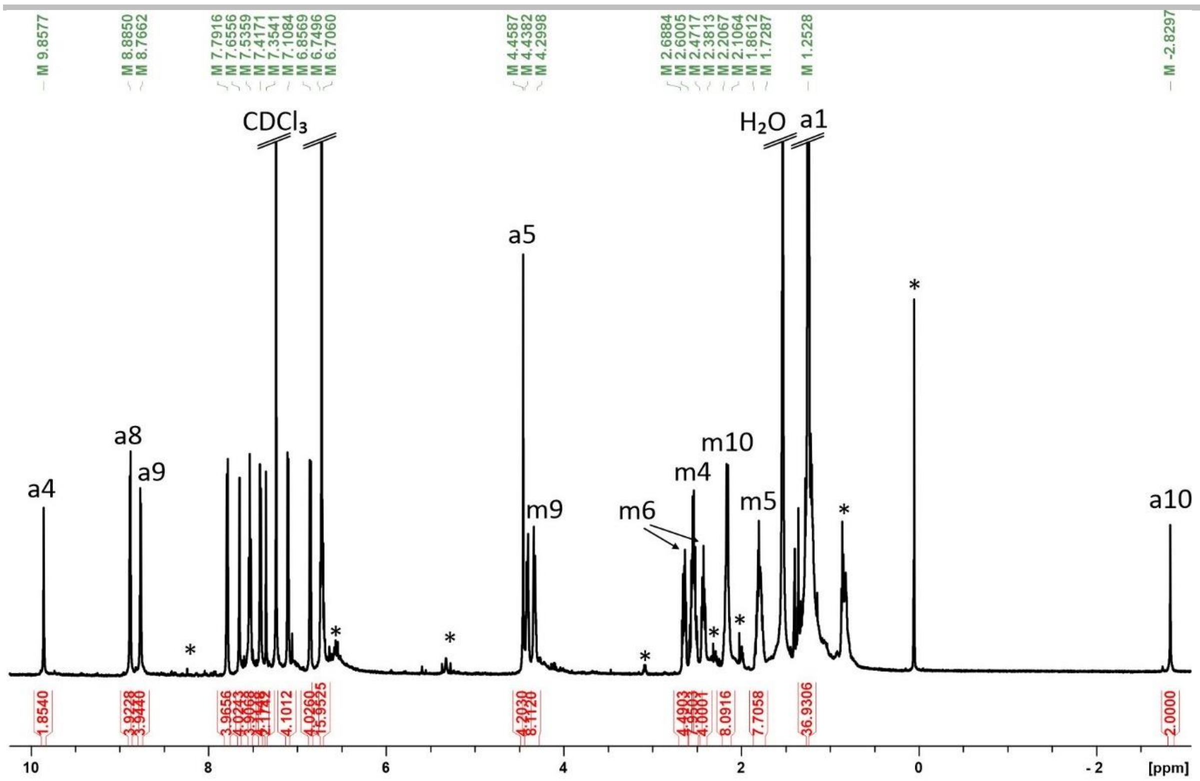

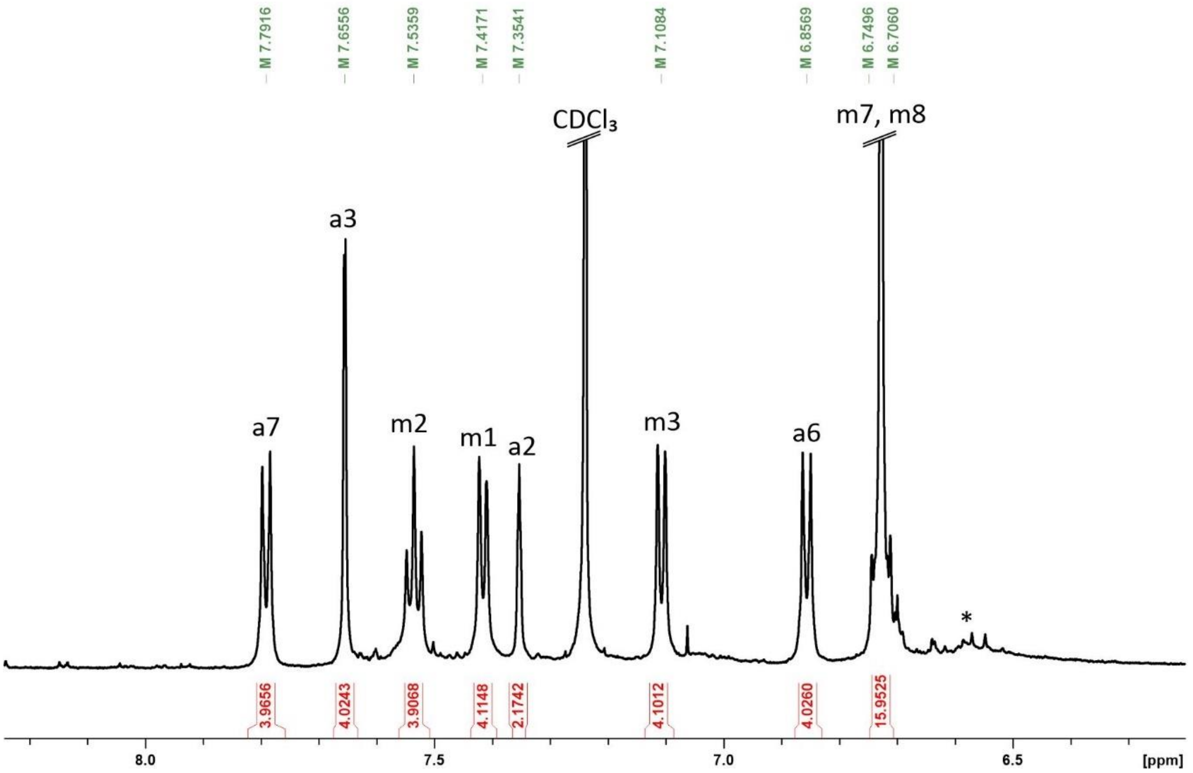

Figure S 40. The ^{1}H NMR spectrum of **9** (600 MHz, [D]chloroform, 300 K).

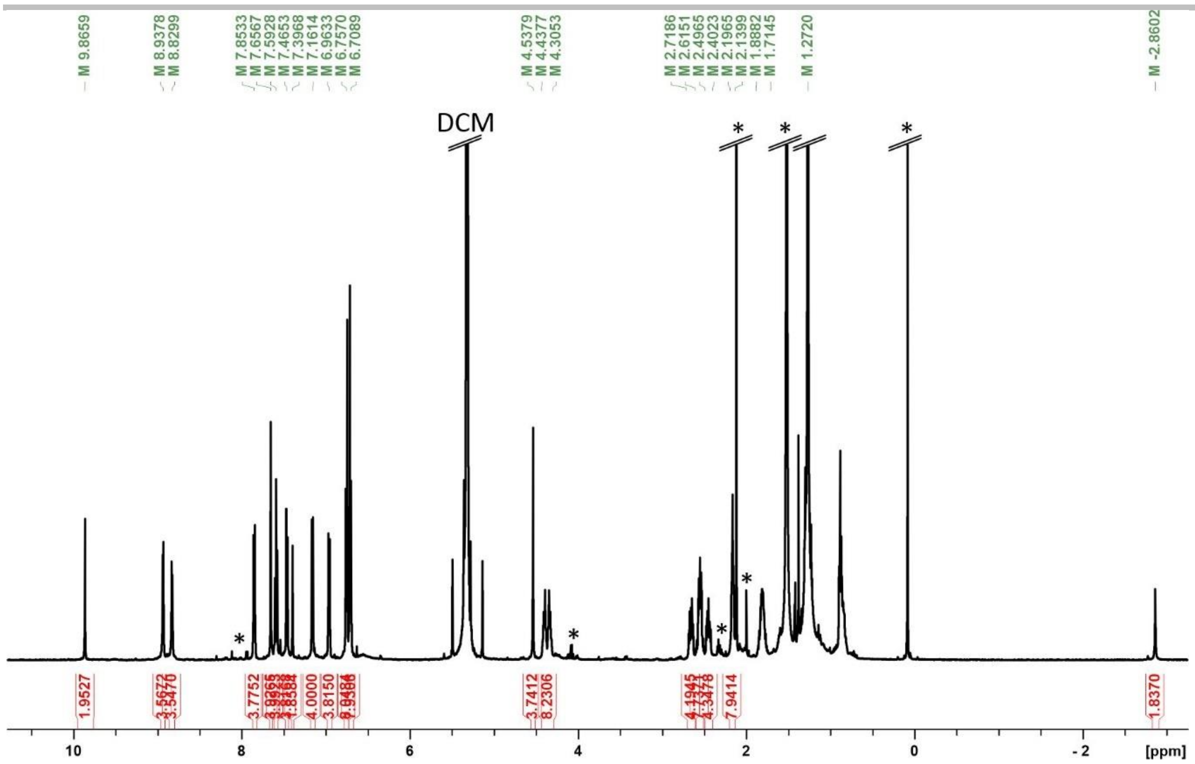

Figure S 41. The ^{1}H NMR spectrum of **9** (500 MHz, [D_2]dichloromethane, 300 K).

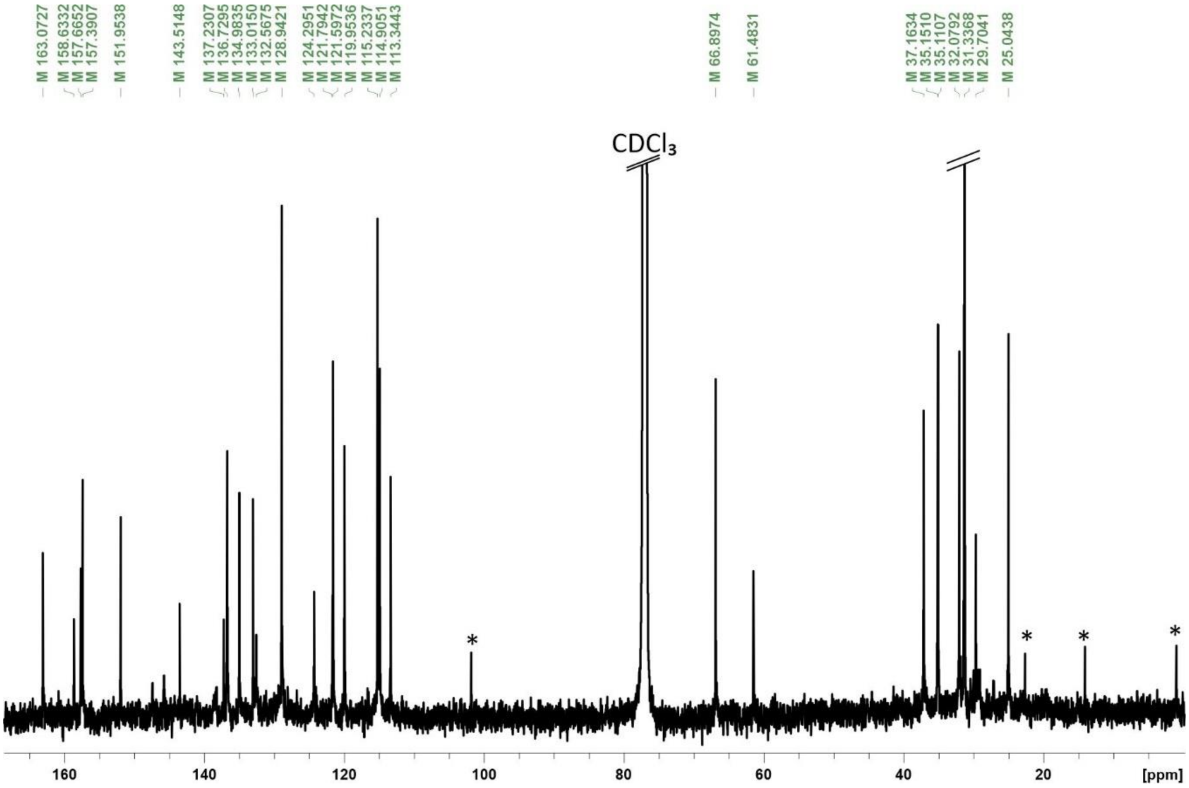

Figure S 42. The ^{13}C NMR spectrum of **9** (151 MHz, [D]chloroform, 300 K). Not all of the resonances could be identified due to the broadening.[23]

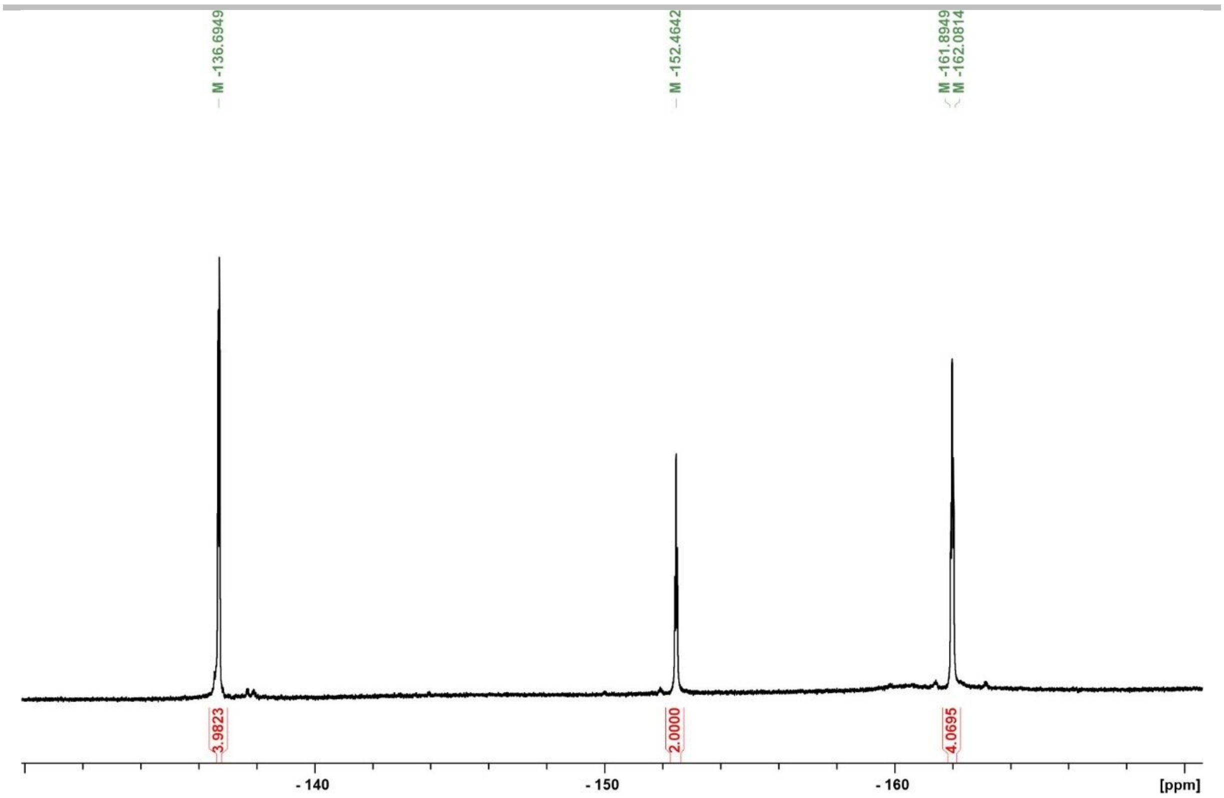

Figure S 43. The ^{19}F NMR spectrum of **9** (470 MHz, [D]chloroform, 300 K).

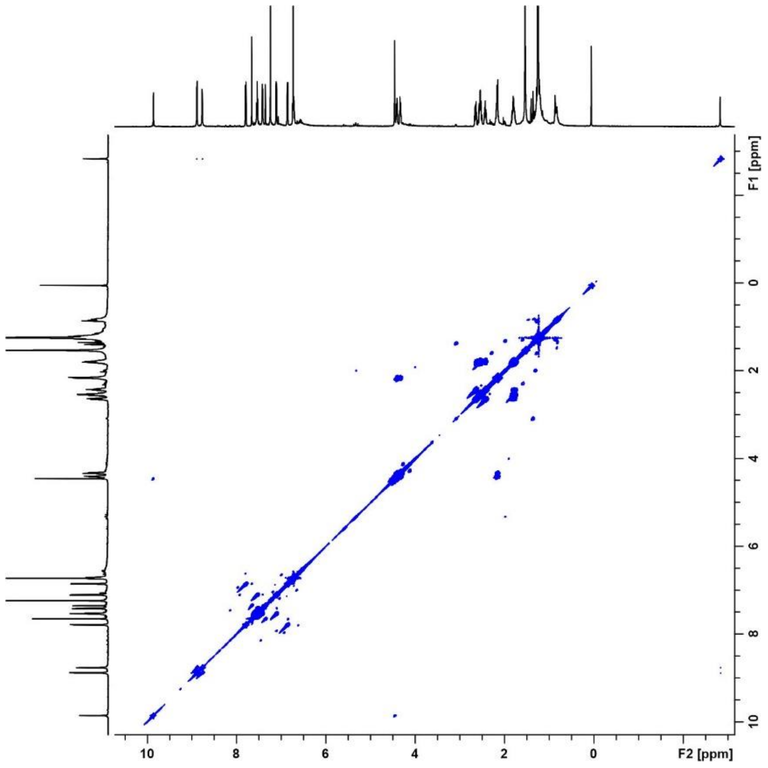

Figure S 44. The ^{1}H-^{1}H COSY NMR spectrum of **9** (500 MHz, [D]chloroform, 300 K).

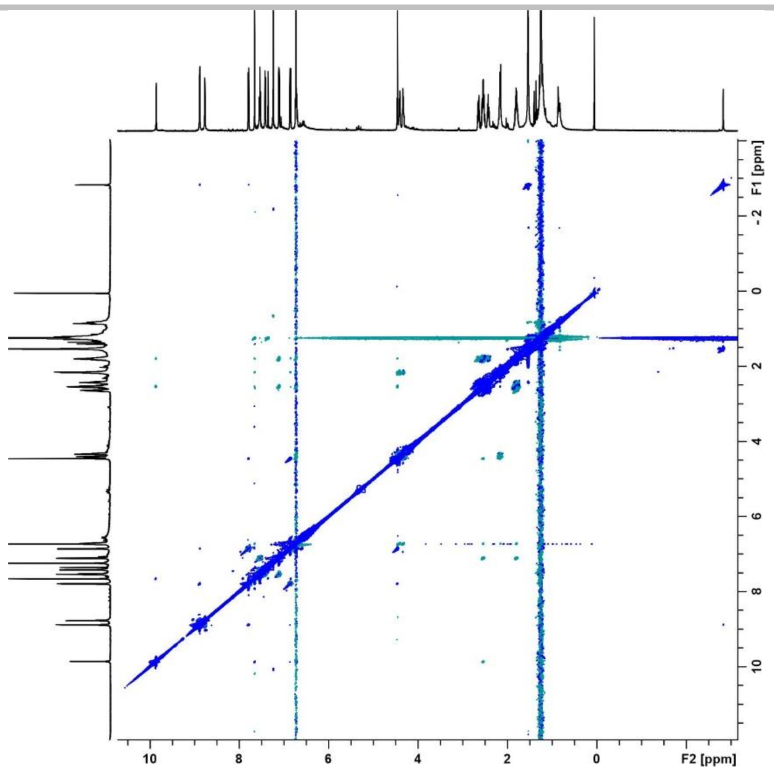

Figure S 45. The ^{1}H-^{1}H NOESY NMR spectrum of **9** (500 MHz, [D]chloroform, 300 K).

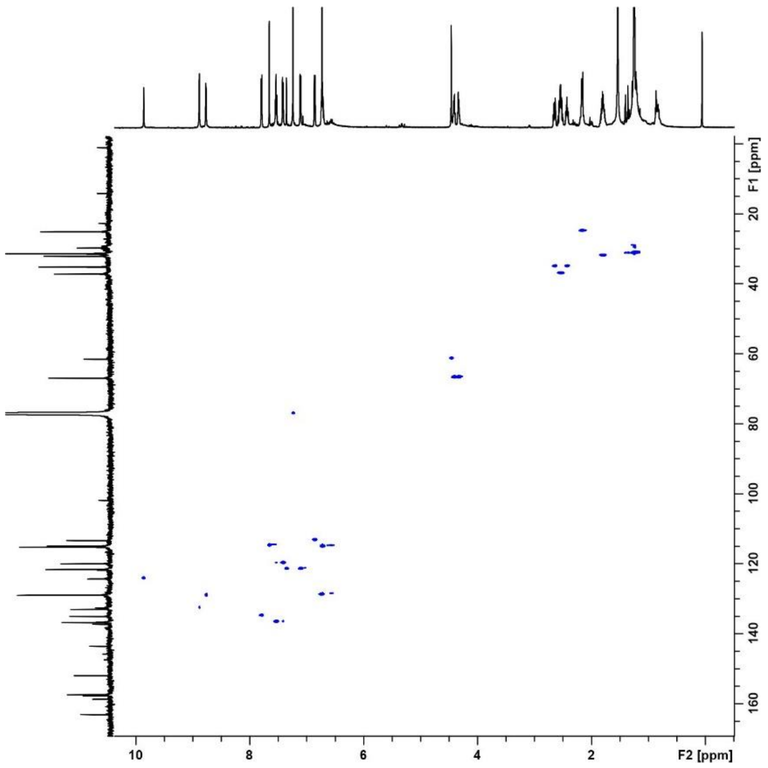

Figure S 46. The ^{1}H-^{13}C HSQC NMR spectrum of **9** (500 MHz, [D]chloroform, 300 K).

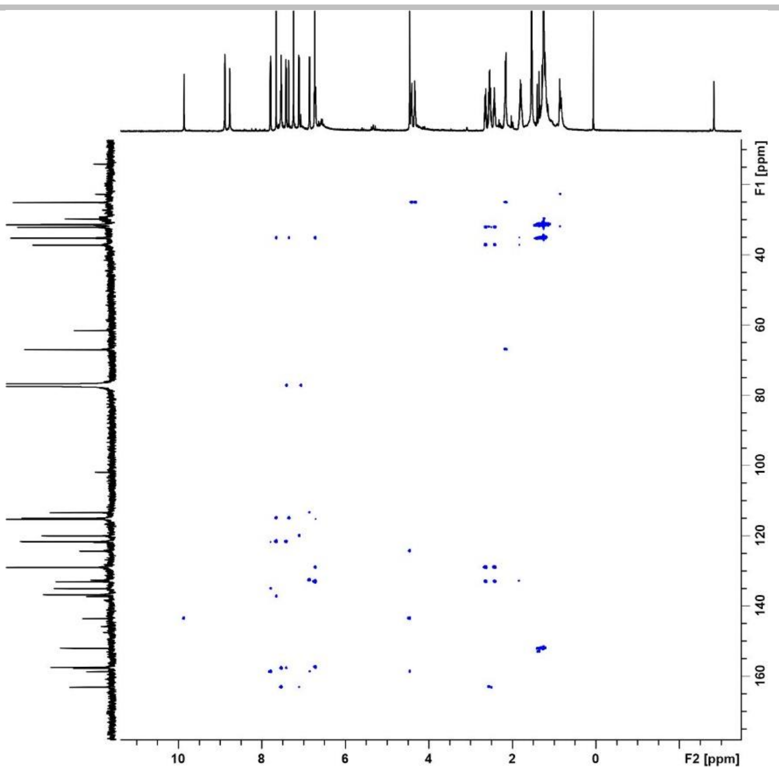

Figure S 47. The ^{1}H-^{13}C HMBC spectrum of **9** (500 MHz, [D]chloroform, 300 K).

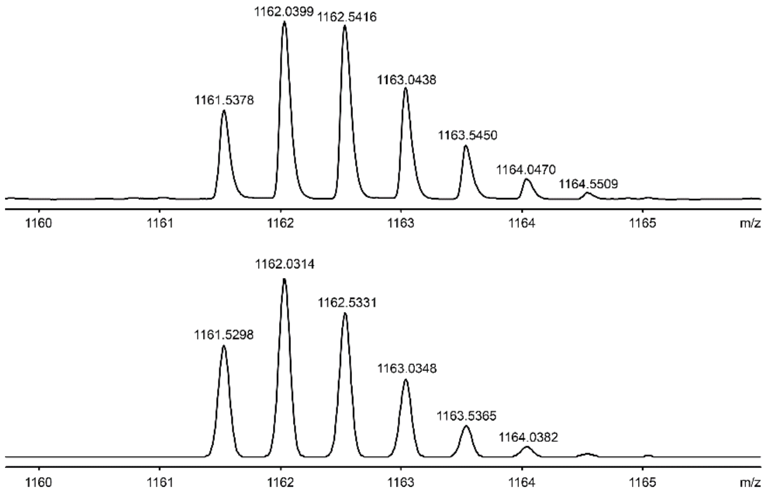

Figure S 48. The high-resolution ESI mass spectrum of **9**. Top: experimental spectrum, bottom: simulated isotopic pattern.

Compounds 10 and 11

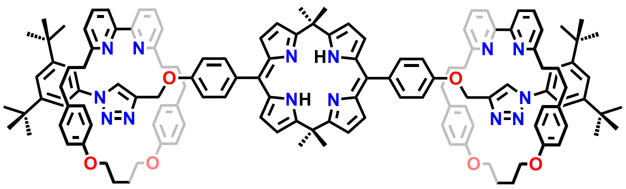

10; 31%

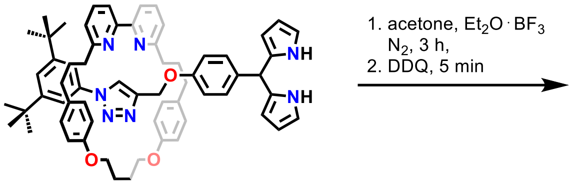

1

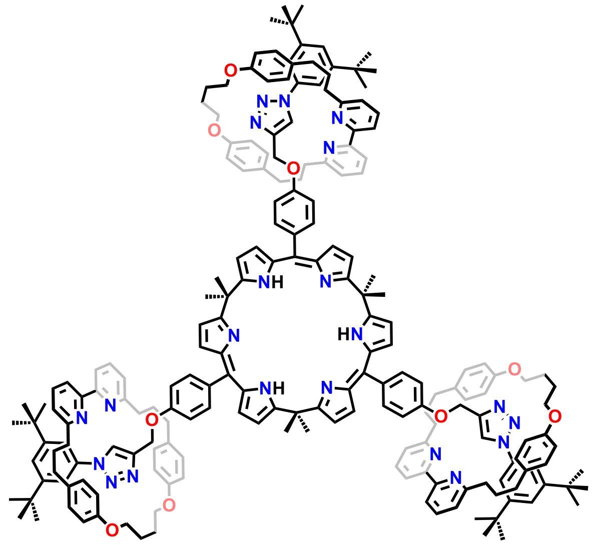

11; 7%

In a 50 ml round bottom flask, **1** (100 mg, 101 μmol) and acetone (25 ml) were introduced. The mixture was flushed by nitrogen bubbling under a sealed flask for 10 minutes. Then $Et_2O·BF_3$ (35 μl, 284 μmol) was added via syringe. The mixture was then stirred for 3 h under a nitrogen atmosphere. After this time septum was opened, DDQ (46 mg, 202 μmol) was introduced, and the reaction was carried out for an additional 5 min. Then, the acid was quenched by TEA (0.5 ml), and the mixture was passed through a short column with deactivated aluminum oxide. Residues on the column were washed out with ethyl acetate. The solvent was removed under reduced pressure. The reddish oil was purified via column chromatography (hexane with 15-40% ethyl acetate gradient) to provide **10** (33 mg, 16.1 μmol, 31%) as an orange-red solid and **11** (7 mg, 2.3 μmol, 7%) as an orange-red solid.

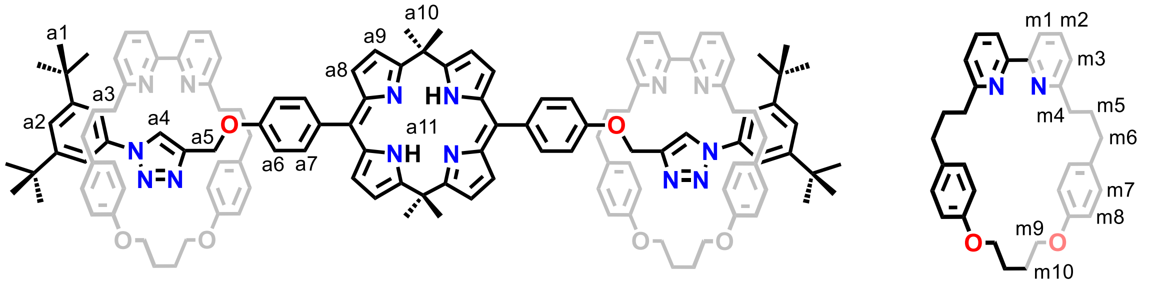

^{1}H NMR (600 MHz, [D]chloroform, 300 K, ppm): δ 14.15 (s, 2H, H_{a11}), 9.71 (s, 2H, H_{a4}), 7.57 (d, ^3J = 1.6 Hz, 4H, H_{a3}), 7.53 (t, ^3J = 7.7 Hz, 4H, H_{m2}), 7.38 (d, ^3J = 7.7 Hz, 4H, H_{m1}), 7.30 (t, ^3J = 1.6 Hz, 2H, H_{a2}), 7.06 (d, ^3J = 8.7 Hz, 4H, H_{a7}), 7.02 (d, ^3J = 7.7 Hz, 4H, H_{m3}), 6.57–6.53 (m, 16H, H_{m7}, H_{m8}), 6.41 (d, ^3J = 8.7 Hz, 4H, H_{a6}), 6.25 (d, ^3J = 4.1 Hz, 4H, H_{a8}), 6.21 (d, ^3J = 4.1 Hz, 4H, H_{a9}), 4.32–4.20 (m, 8H, H_{m9}), 4.21 (s, 4H, H_{a5}), 2.53–2.47 (m, 4H, H_{m6}), 2.47–2.27 (m, 12H, H_{m6}, H_{m4}), 2.10–2.06 (m, 8H, H_{m10}), 1.93 (s, 12H, H_{a10}), 1.70–1.51 (m, 8H, H_{m5}), 1.21 (s, 36H, H_{a1}); **^{13}C NMR** (151 MHz, [D]chloroform, 300 K, ppm): δ 164.7, 162.9, 159.2, 157.5, 157.2, 151.8, 143.3, 140.9, 140.4, 137.1, 136.6, 132.8, 131.6, 128.7, 128.6, 128.3, 124.1, 121.5, 119.8, 115.0, 114.9, 114.8, 113.8, 113.7, 66.8, 61.3, 38.2, 37.0, 35.04, 35.00, 31.9, 31.3, 24.9; **HR–ESI–MS** (*m/z*): $[M+H]^+$ calcd. for $C_{134}H_{147}N_{14}O_6^+$, 2049.1654; found, 2049.1736.

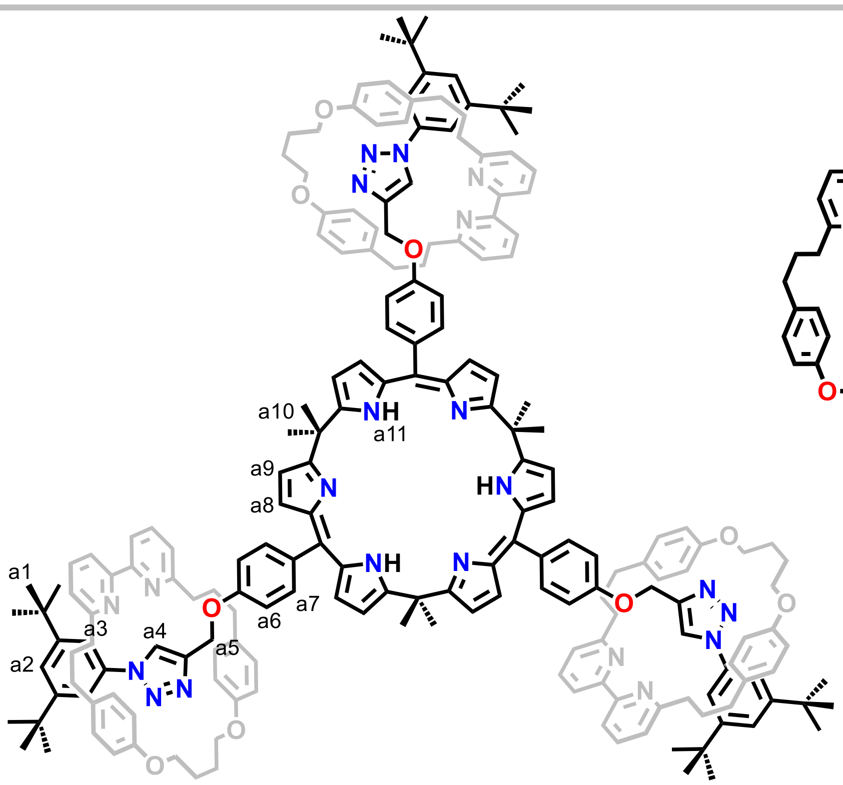

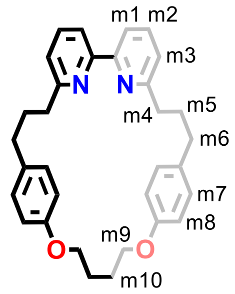

^{1}H NMR (500 MHz, [D]chloroform, 300 K, ppm): δ 13.12 (s, 3H, H_{a11}), 9.66 (s, 3H, H_{a4}), 7.58 (d, ^3J = 1.6 Hz, 6H, H_{a3}), 7.53 (t, ^3J = 7.7 Hz, 6H, H_{m2}), 7.38 (d, ^3J = 7.7 Hz, 6H, H_{m1}), 7.31 (t, ^3J = 1.6 Hz, 3H, H_{a2}), 7.09 (d, ^3J = 8.7 Hz, 6H, H_{a7}), 7.02 (d, ^3J = 7.7 Hz, 6H, H_{m3}), 6.58–6.54 (m, 24H, H_{m7}, H_{m8}), 6.46–6.42 (m, 12H, H_{a6}, H_{a8}), 6.34 (d, ^3J = 4.2 Hz, 6H, H_{a9}), 4.33–4.20 (m, 12H, H_{m9}), 4.19 (s, 6H, H_{a5}), 2.56–2.46 (m, 6H, H_{m6}), 2.44–2.27 (m, 18H, H_{m6}, H_{m4}), 2.12–2.03 (m, 12H, H_{m10}), 1.76 (s, 18H, H_{a10}), 1.71–1.53 (m, 12H, H_{m5}), 1.22 (s, 54H, H_{a1}); **^{13}C NMR** (125 MHz, [D]chloroform, 300 K, ppm): δ 162.9, 162.1, 159.1, 157.6, 157.2, 151.9, 143.4, 140.3, 139.7, 137.2, 136.6, 132.9, 131.3, 128.9, 128.8, 124.1, 121.5, 119.9, 115.1, 115.0, 114.9, 113.9, 66.9, 61.3, 39.1, 37.0, 35.07, 35.03, 31.9, 31.3, 29.7, 25.0; **HR–ESI–MS** (*m/z*): $[M+H]^+$ calcd. for $C_{201}H_{220}N_{21}O_9^+$, 3073.7461; found, 3073.7550.

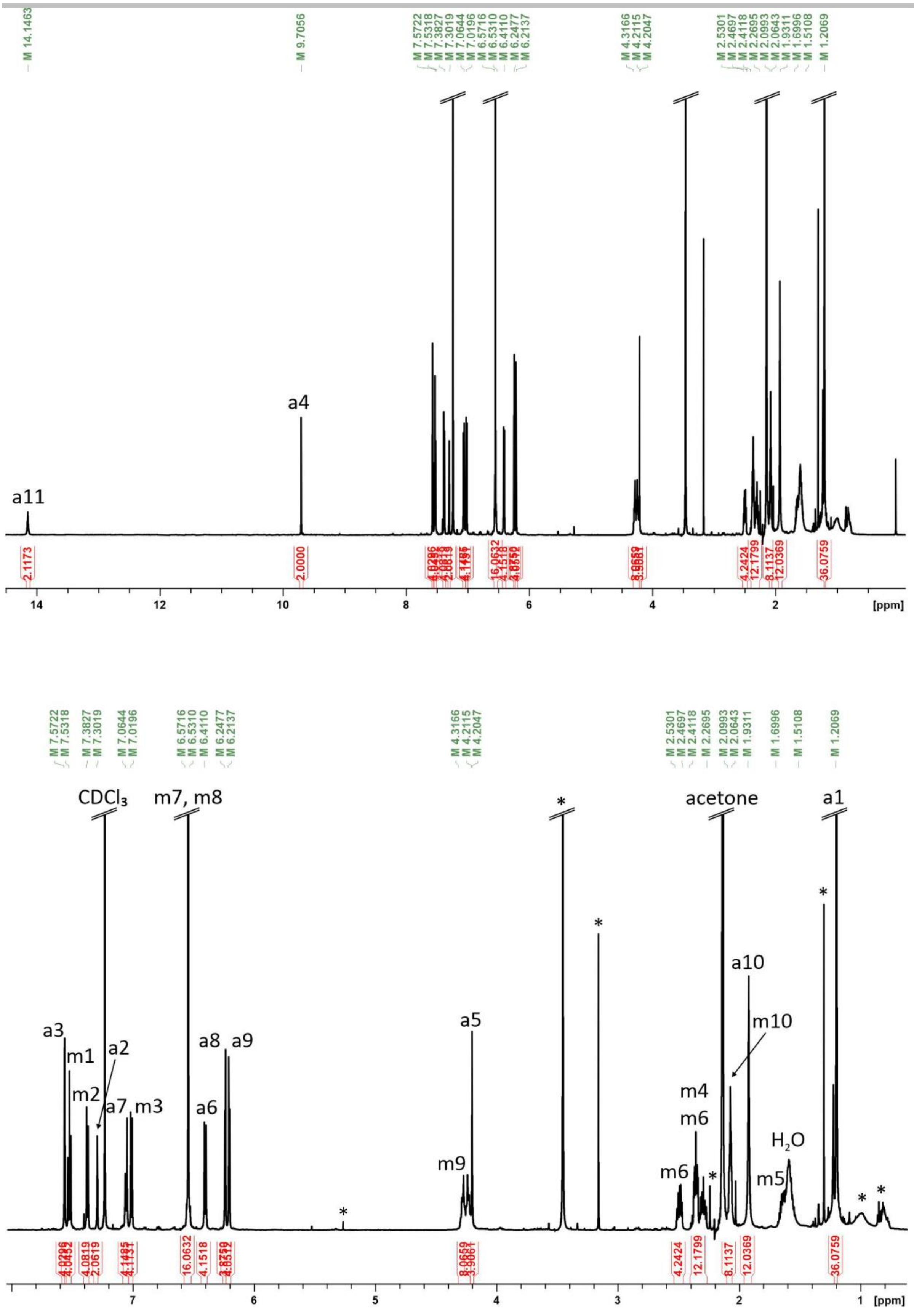

Figure S 49. The ^{1}H NMR spectrum of **10** (600 MHz, [D]chloroform, 300 K).

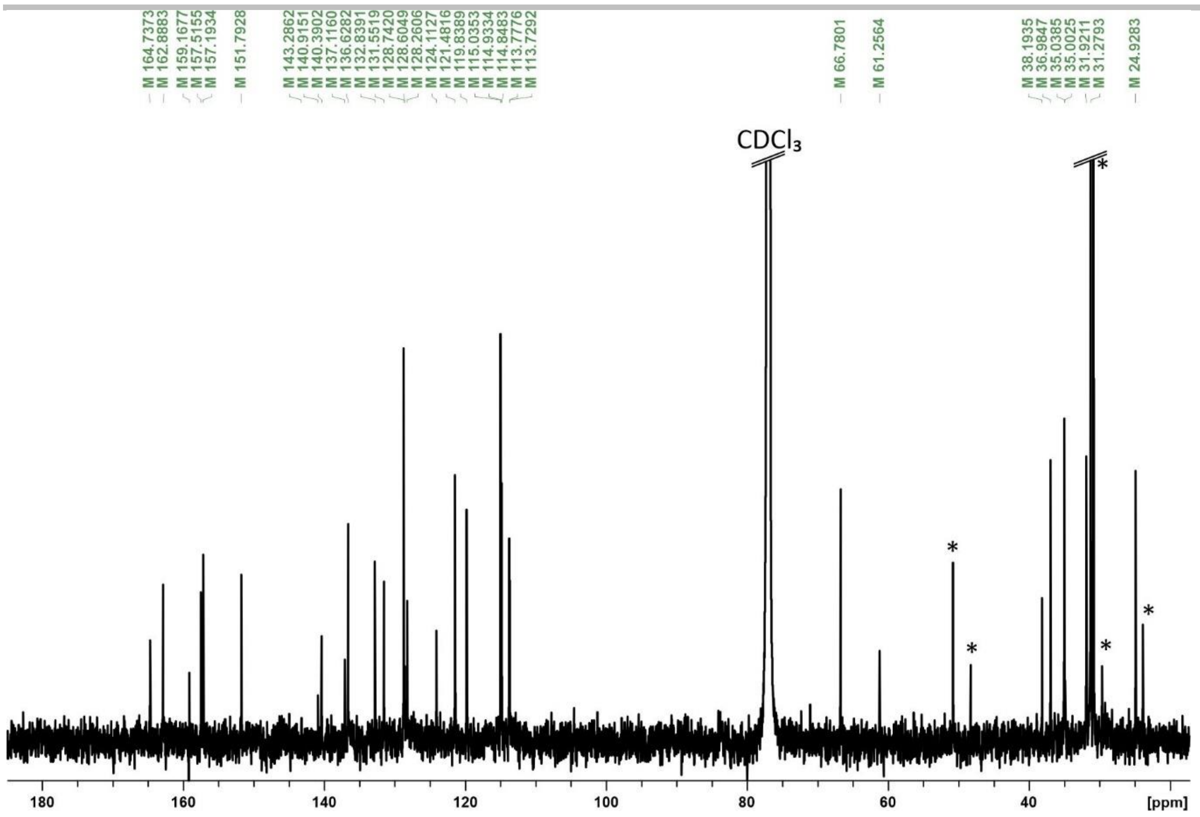

Figure S 50. The ^{13}C NMR spectrum of **10** (151 MHz, [D]chloroform, 300 K).

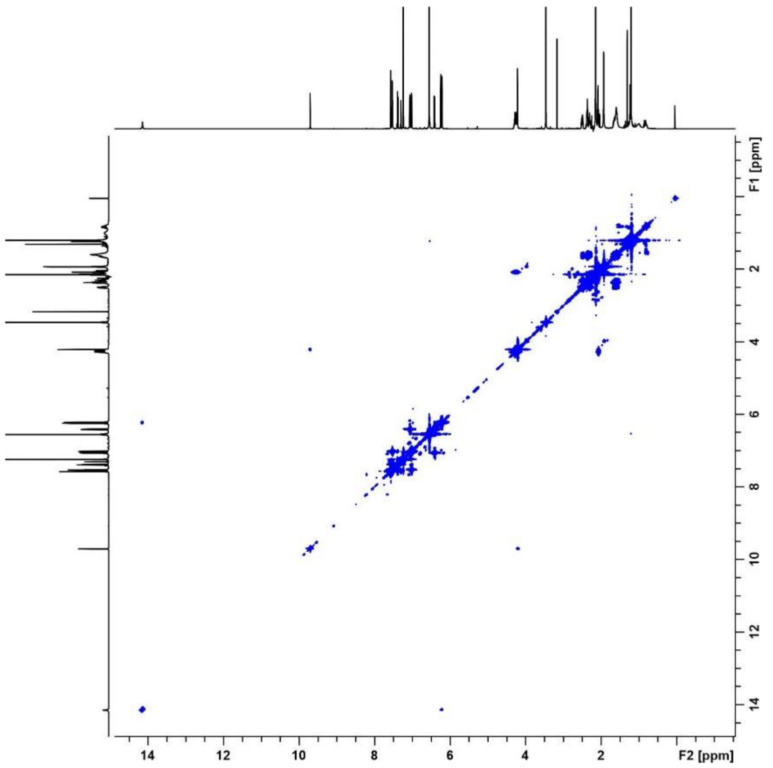

Figure S 51. The ^{1}H-^{1}H COSY NMR spectrum of **10** (600 MHz, [D]chloroform, 300 K).

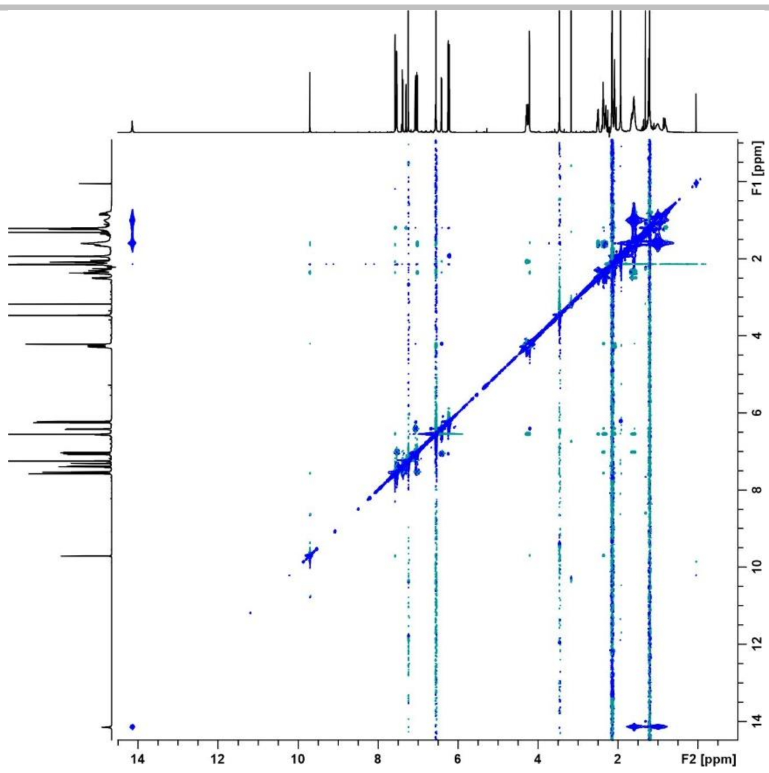

Figure S 52. The ^{1}H-^{1}H NOESY NMR spectrum of **10** (600 MHz, [D]chloroform, 300 K).

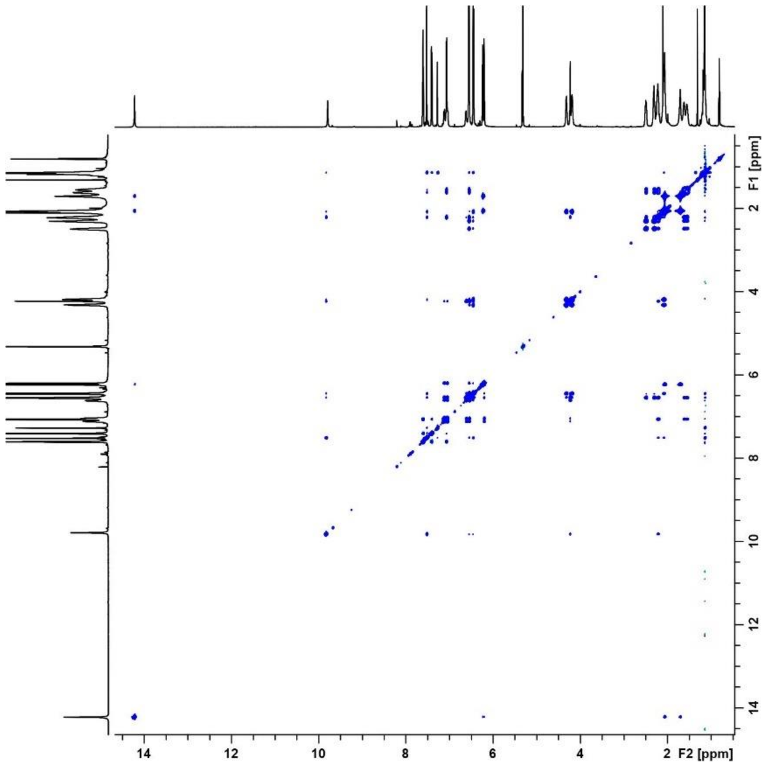

Figure S 53. The ^{1}H-^{1}H NOESY NMR spectrum of **10** (600 MHz, [D_2]dichloromethane, 210 K).

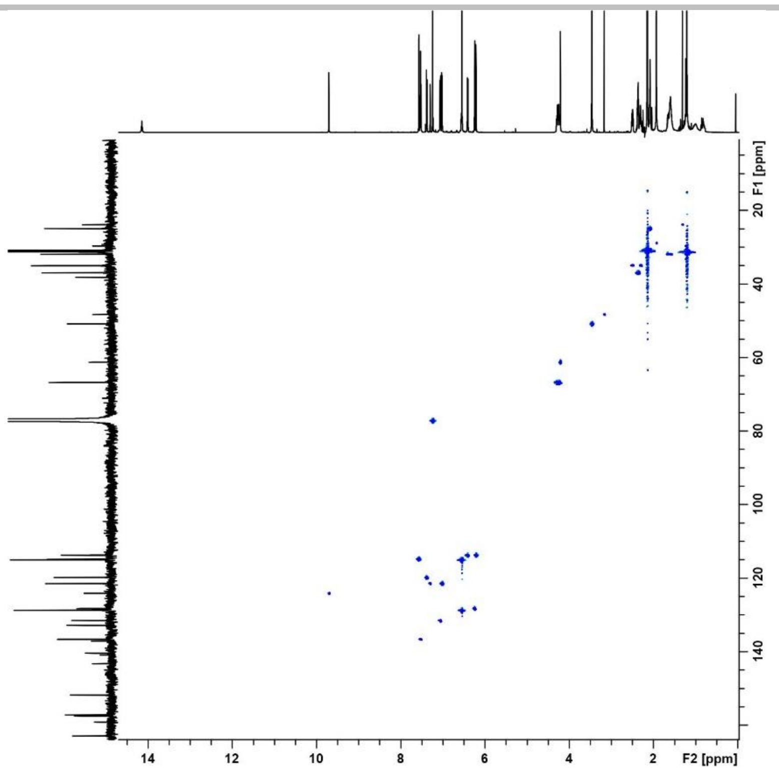

Figure S 54. The ^{1}H-^{13}C HSQC NMR spectrum of **10** (600 MHz, [D]chloroform, 300 K).

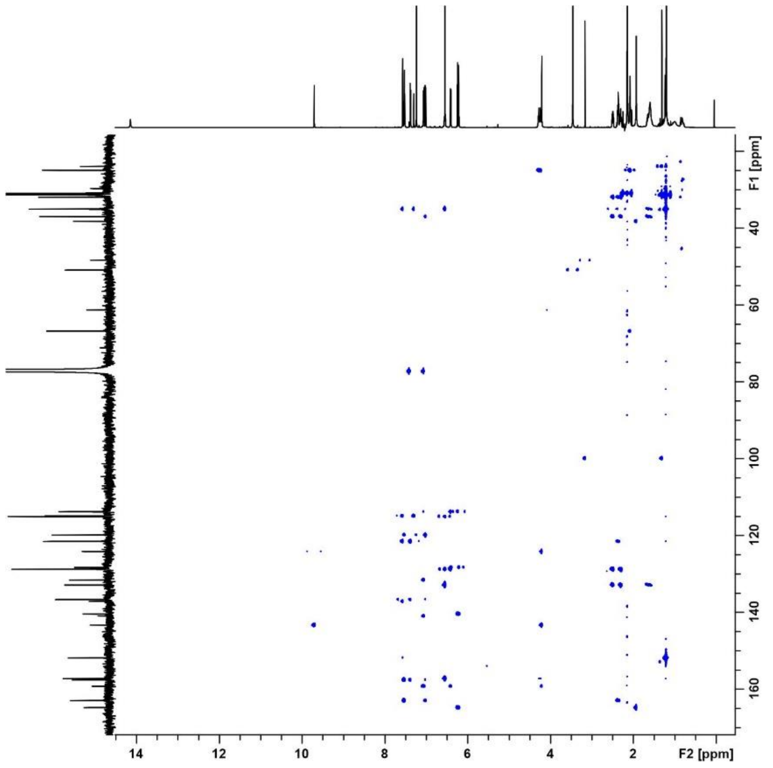

Figure S 55. The ^{1}H-^{13}C HMBC NMR spectrum of **10** (600 MHz, [D]chloroform, 300 K).

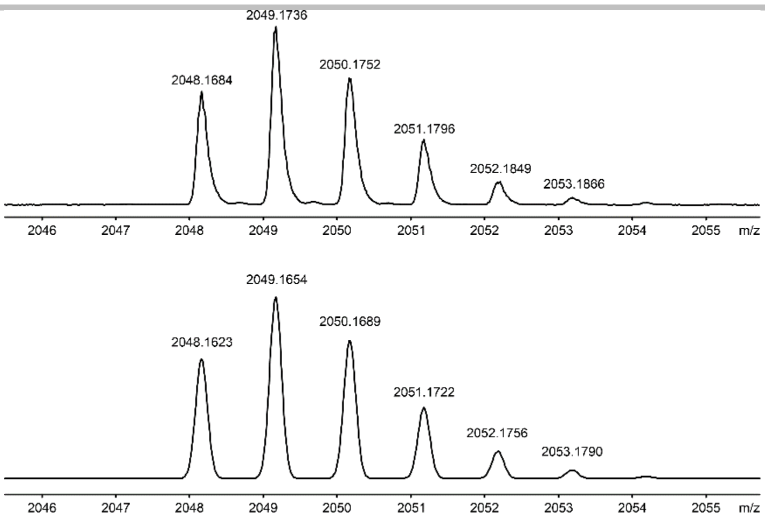

Figure S 56. The high-resolution mass spectrum of **10** (ESI MS). Top: experimental spectrum, bottom: simulated isotopic pattern.

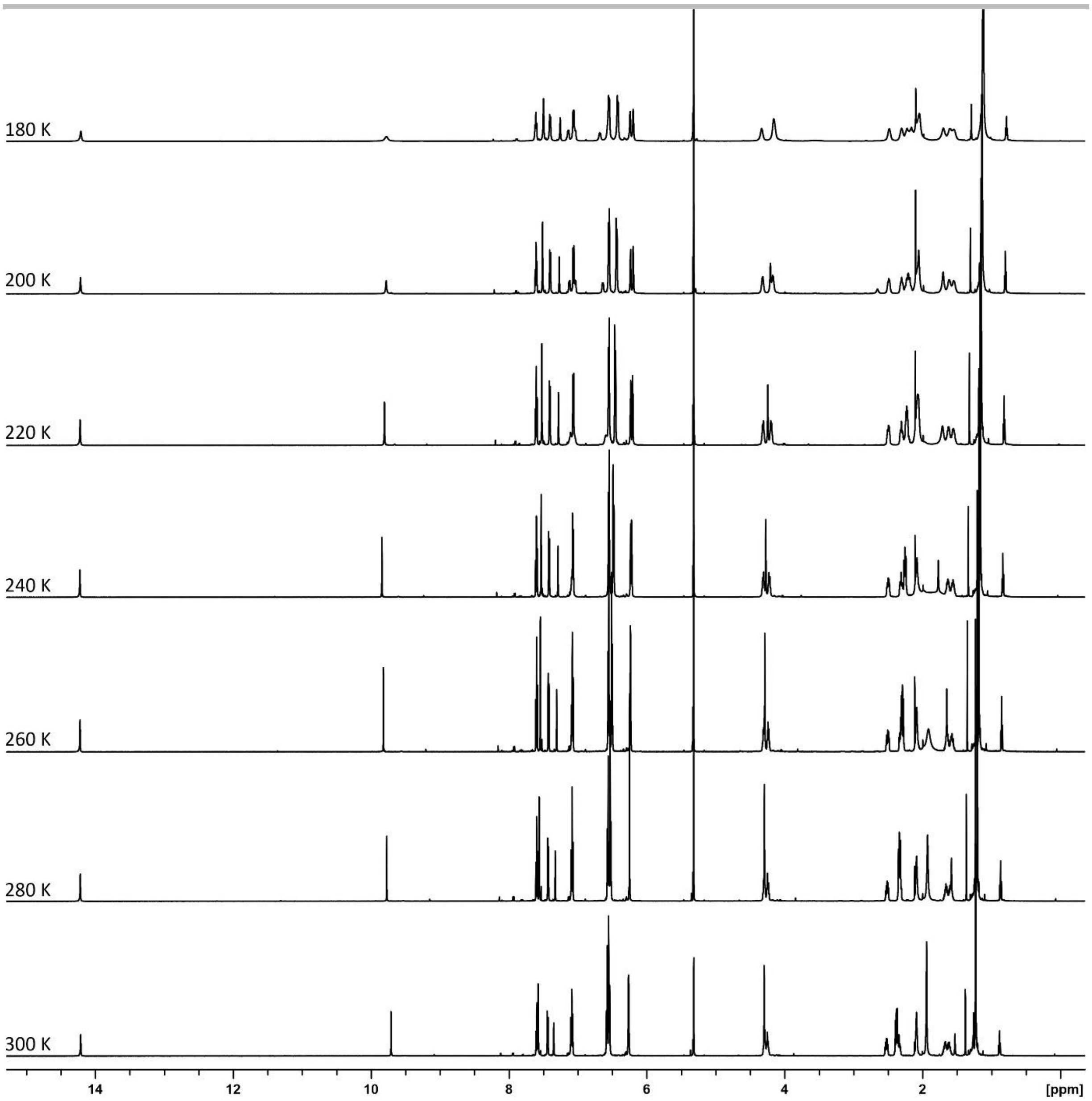

Figure S 57. The ^{1}H NMR spectra of **10** recorded in the 300–180 K temperature range (600 MHz, [D$_2$]dichloromethane).

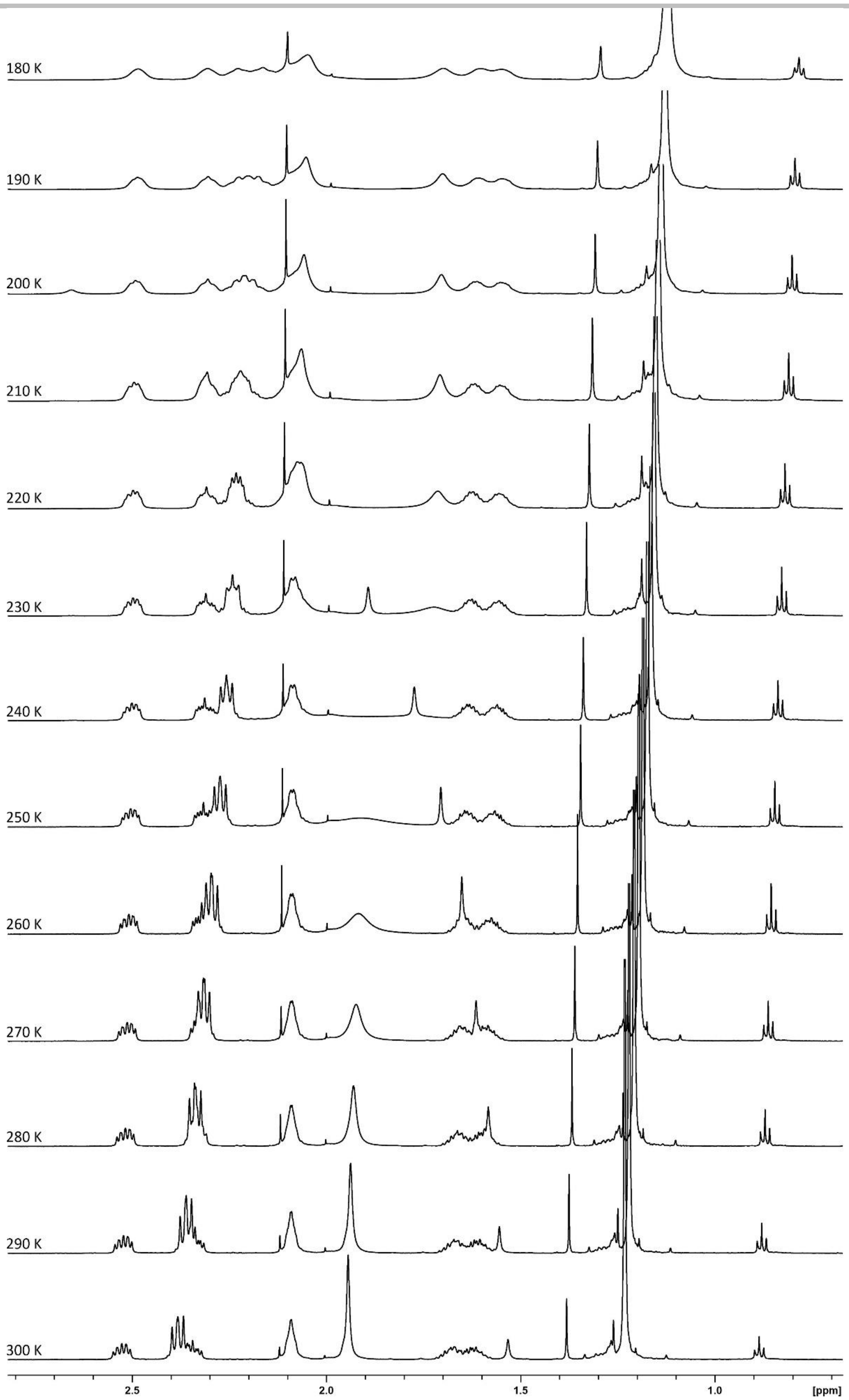

Figure S 58. The aliphatic region of the ^{1}H NMR spectra of **10** recorded at the 300–180 K temperature range (600 MHz, [D$_2$]dichloromethane).

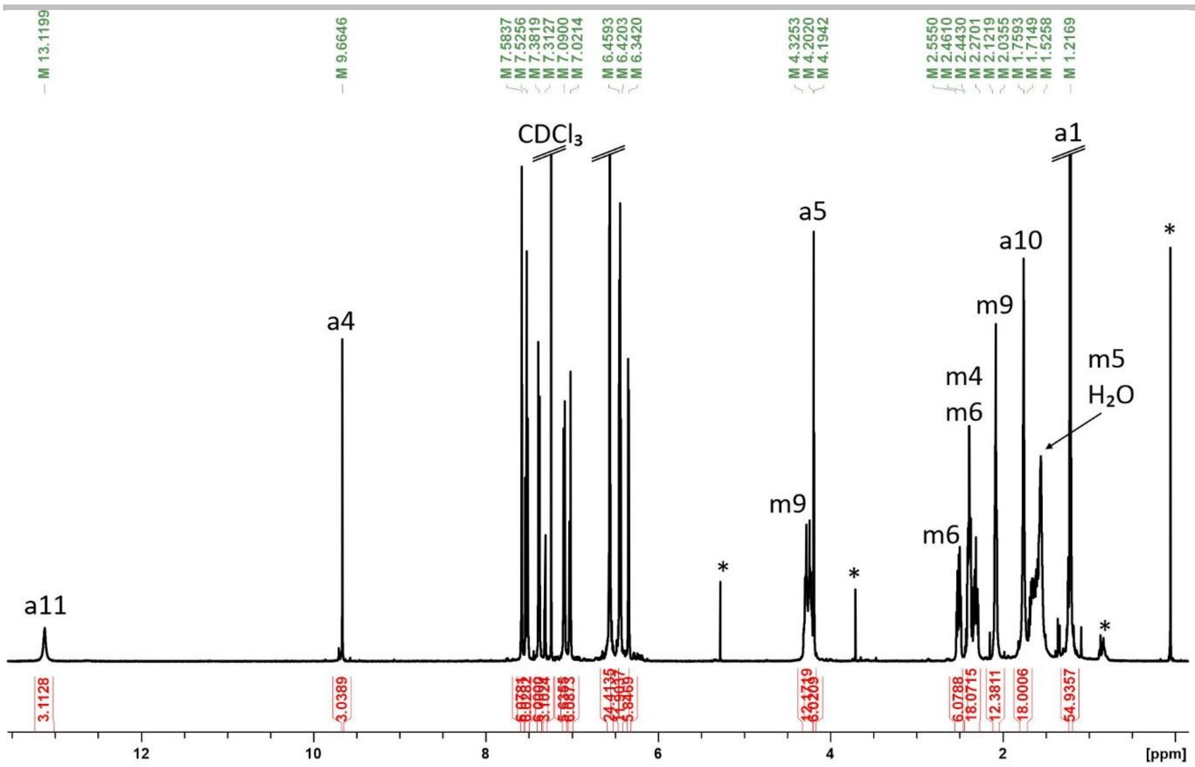

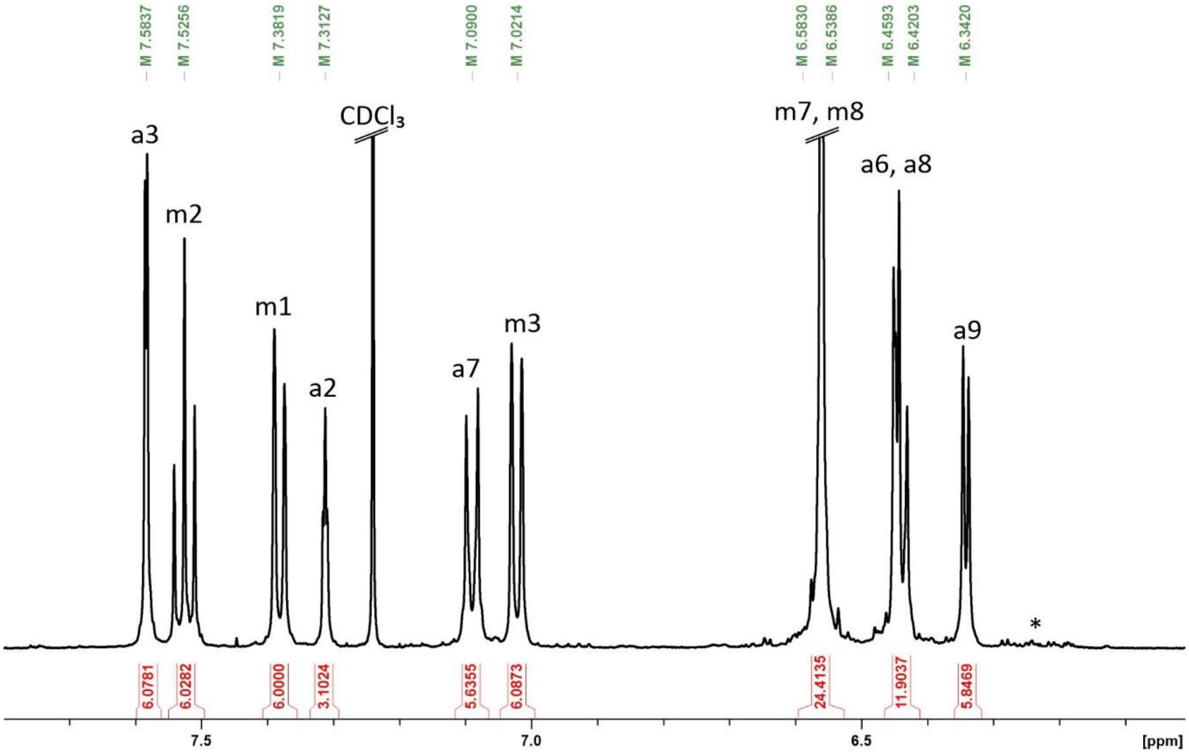

Figure S 59. The ^{1}H NMR spectrum of **11** (500 MHz, [D]chloroform, 300 K).

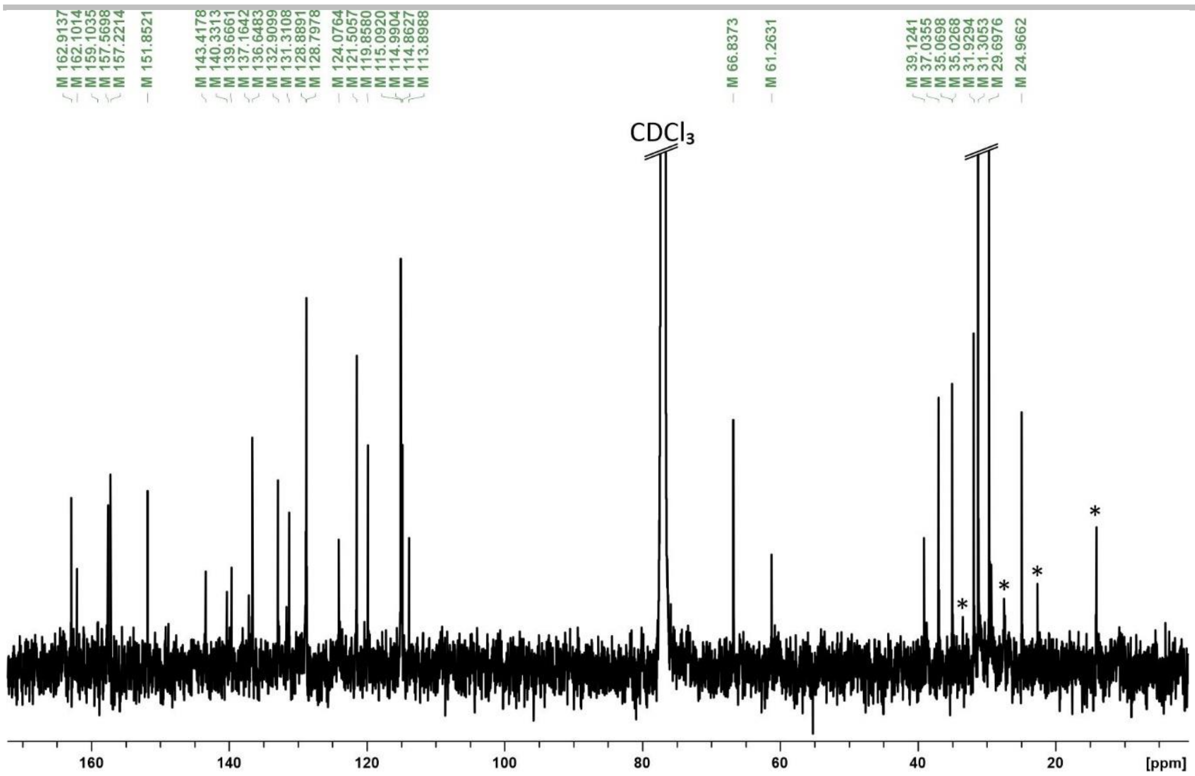

Figure S 60. The ^{13}C NMR spectrum of **11** (125 MHz, [D]chloroform, 300 K).

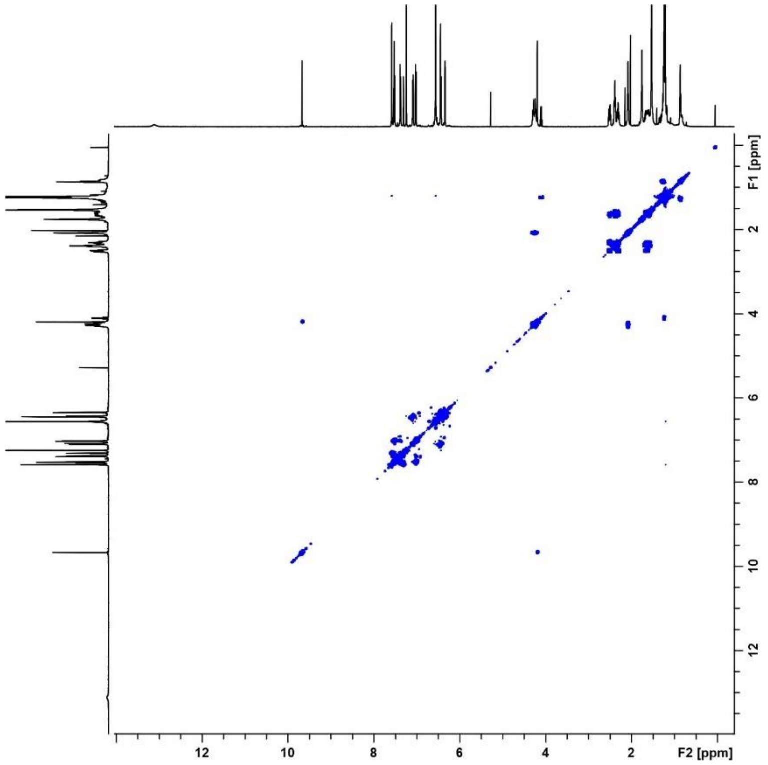

Figure S 61. The ^{1}H-^{1}H COSY NMR spectrum of **11** (500 MHz, [D]chloroform, 300 K).

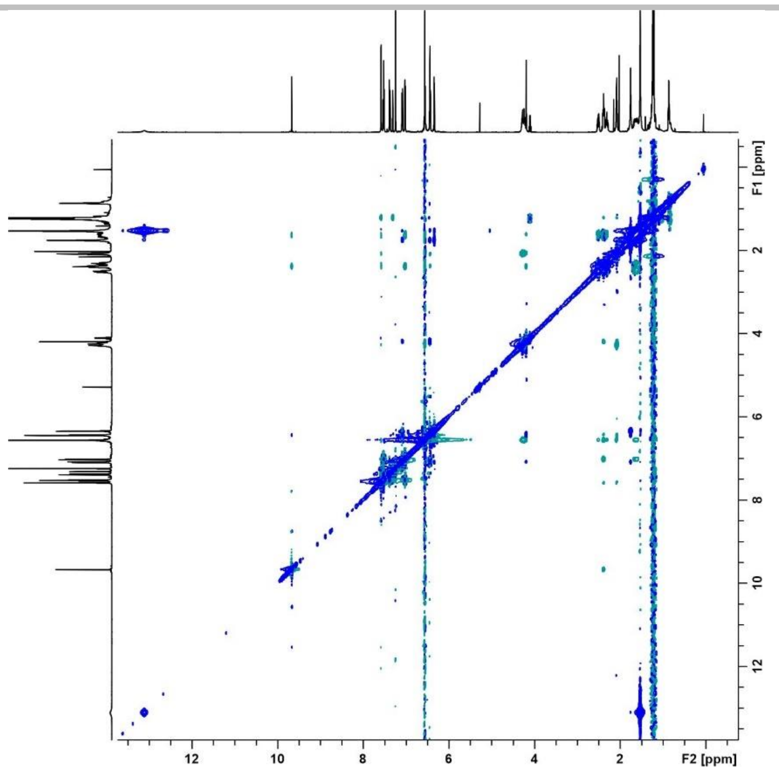

Figure S 62. The ^{1}H-^{1}H NOESY NMR spectrum of **11** (500 MHz, [D]chloroform, 300 K).

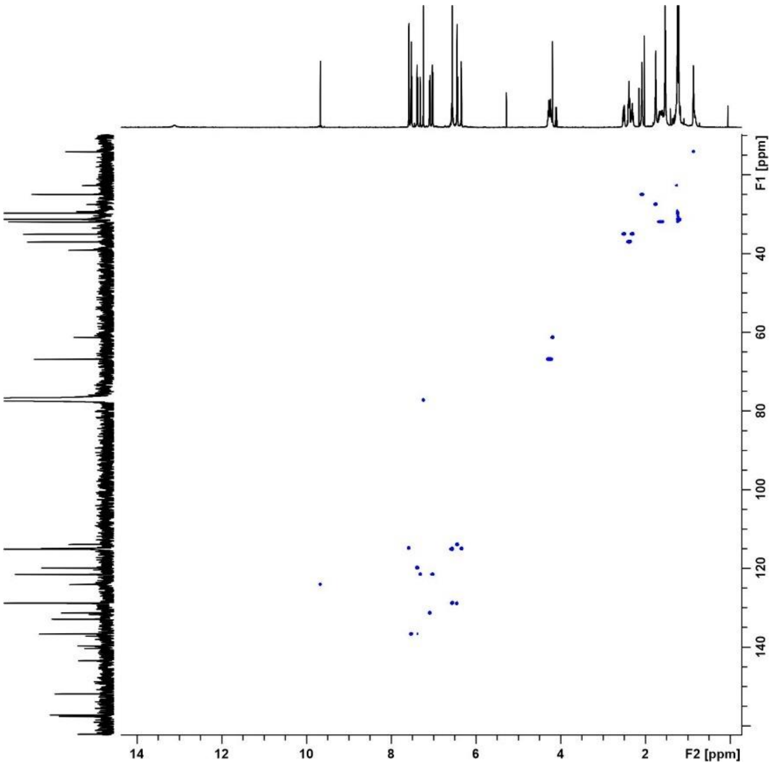

Figure S 63. The ^{1}H-^{13}C HSQC NMR spectrum of **11** (500 MHz, [D]chloroform, 300 K).

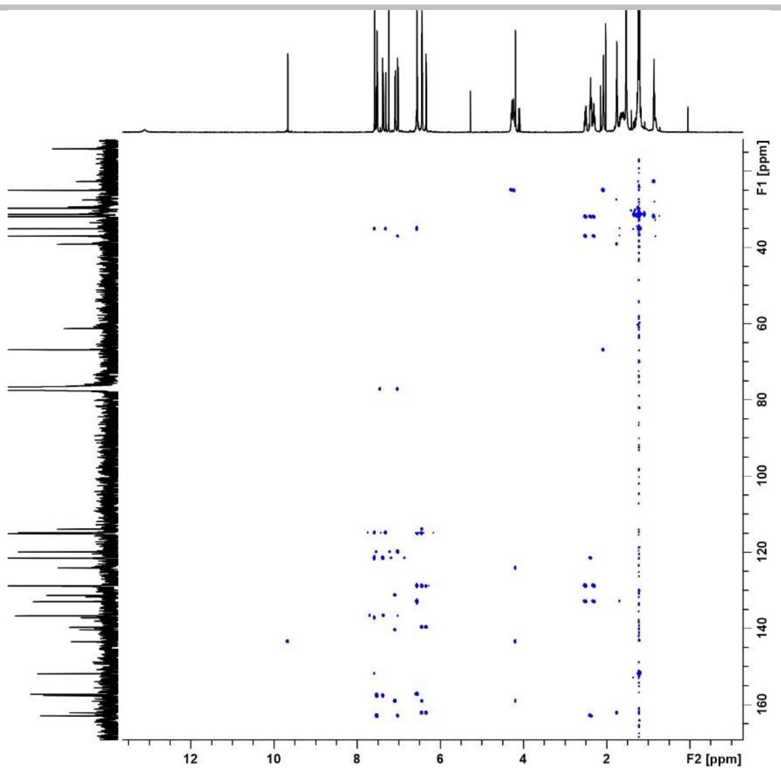

Figure S 64. The ¹H-¹³C HMBC spectrum of **11** (500 MHz, [D]chloroform, 300 K).

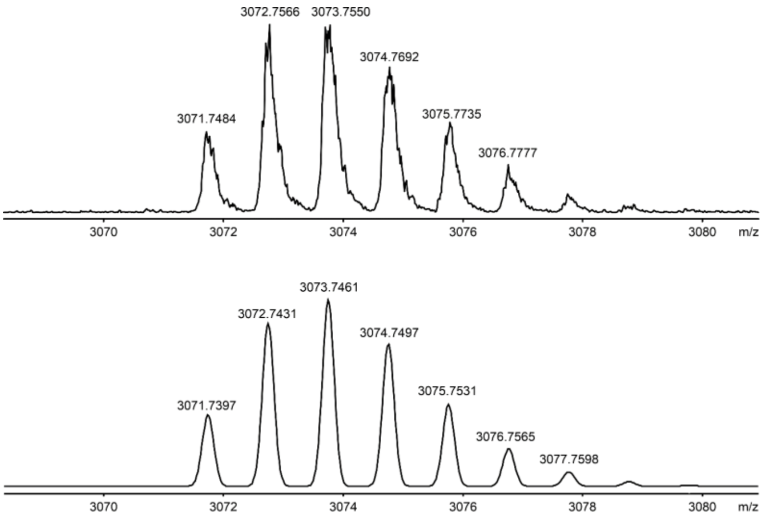

Figure S 65. The high-resolution mass spectrum of **11** (ESI MS). Top: experimental spectrum, bottom: simulated isotopic pattern.

Compound [10-H$_2$]$^{2+}$

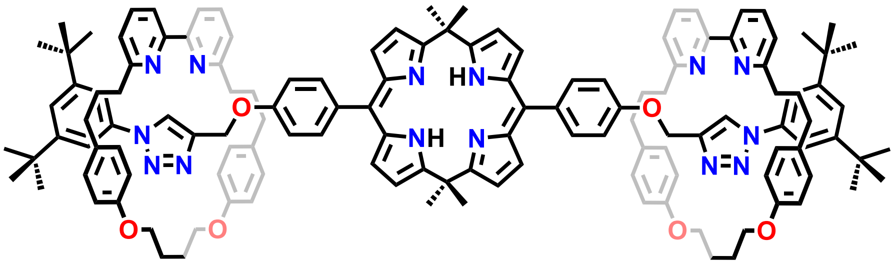

10

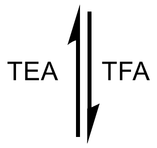

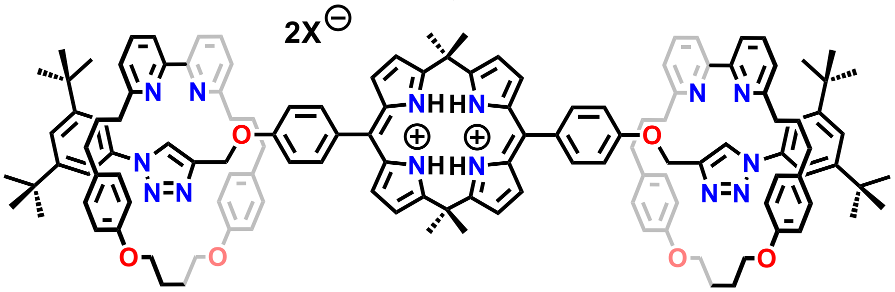

[10-H$_2$]$^{2+}$

The dicationic species **[10-H$_2$]$^{2+}$** was obtained by acidification of **10** with ca. two equiv. of trifluoroacetic acid in [D$_2$]dichloromethane.

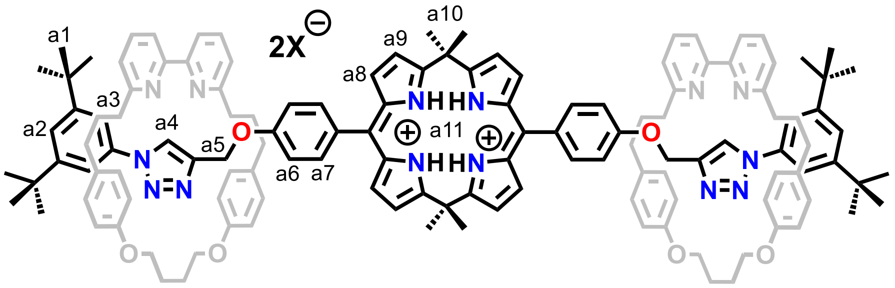

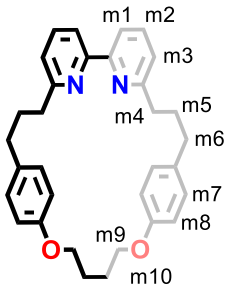

^{1}H NMR (600 MHz, [D$_2$]dichloromethane, 200 K, ppm): δ 13.80 (s, 4H, H_{a11}), 9.44 (s, 2H, H_{a4}), 7.68–7.61 (m, 4H, H_{m2}), 7.57 (s, 2H, H_{a3}), 7.45–7.39 (m, 4H, H_{m1}), 7.34 (s, 2H, H_{a2}), 7.13 (d, ^3J = 8.2 Hz, 4H, H_{a7}), 7.08 (d, ^3J = 7.7 Hz, 4H, H_{m3}), 6.82 (d, ^3J = 8.2 Hz, 4H, H_{a6}), 6.68–6.62 (m, 12H, H_{a8}, H_{a9}, H_{m7}), 6.59 (d, ^3J = 8.1 Hz, 4H, H_{m7}), 6.50–6.44 (overlapped d, 8H, H_{m8}), 4.55 (d, ^3J = 11.2 Hz, 2H, H_{a5}), 4.36–4.21 (m, 4H, H_{m9}) 4.11–4.02 (m, 4H, H_{m9}), 3.98 (d, ^3J = 11.2 Hz, 2H, H_{a5}), 2.64–2.53 (m, 4H, H_{m6}), 2.38–2.16 (m, 12H, H_{m4}, H_{m6}), 2.15–2.03 (m, 8H, H_{m10}), 1.76 (s, 12H, H_{a10}), 1.74–1.64 (m, 4H, H_{m5}), 1.60–1.46 (m, 4H, H_{m5}), 1.18 (s, 36H, H_{a1}).

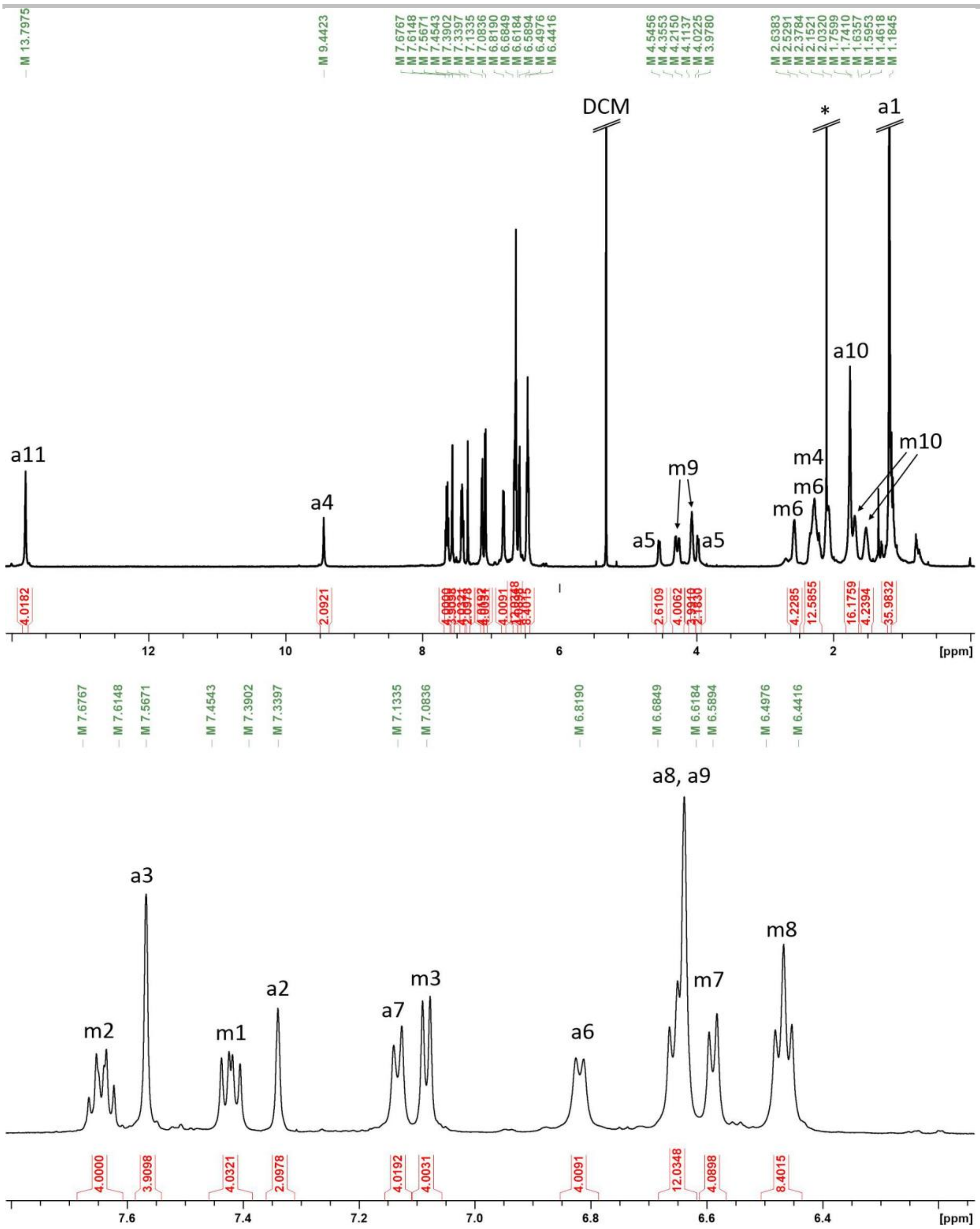

Figure S 66. The ^{1}H NMR spectrum of **$[10\text{-}H_2]^{2+}$** (600 MHz, [D_2]dichloromethane, 200 K).

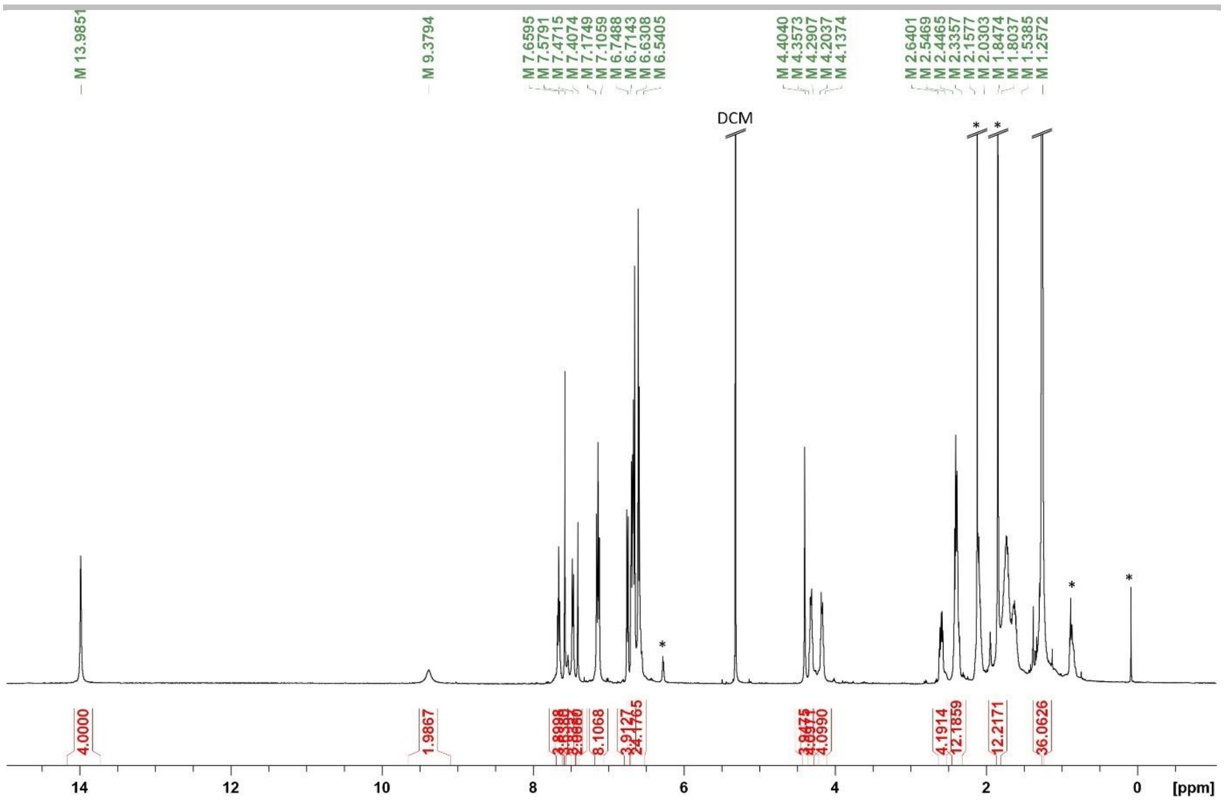

Figure S 67. The ^{1}H NMR spectrum of **[10-H$_2$]$^{2+}$** (500 MHz, [D$_2$]dichloromethane, 300 K).

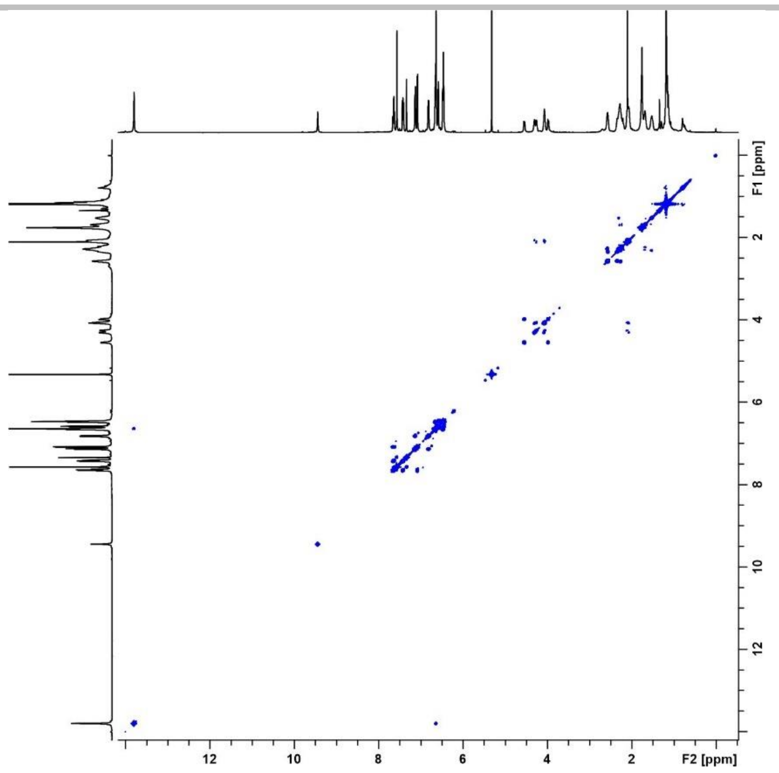

Figure S 68. The ^{1}H-^{1}H COSY NMR spectrum of **[10-H$_2$]$^{2+}$** (600 MHz, [D$_2$]dichloromethane, 200 K).

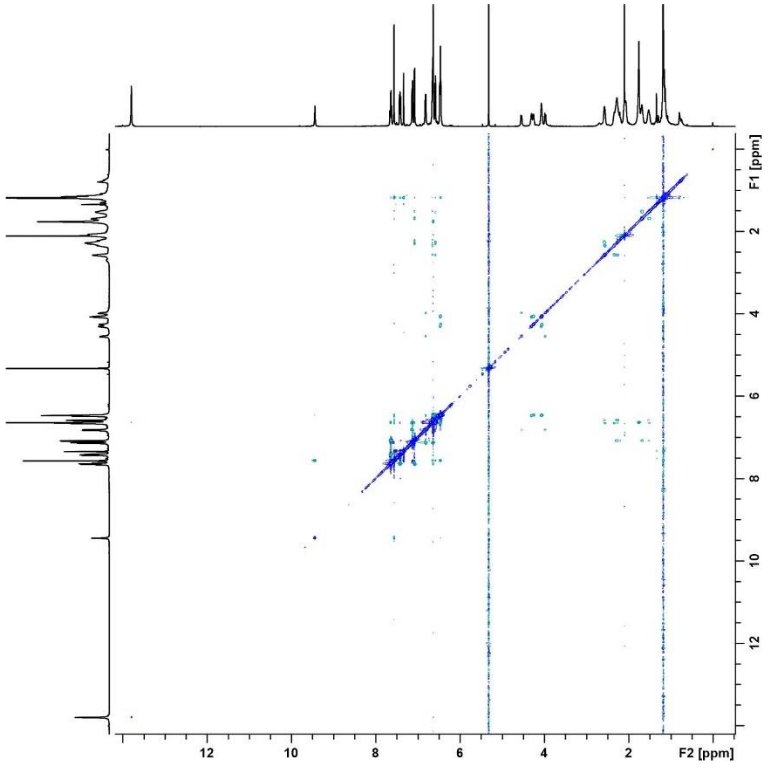

Figure S 69. The ^{1}H-^{1}H ROESY NMR spectrum of **[10-H$_2$]$^{2+}$** (600 MHz, [D$_2$]dichloromethane, 200 K).

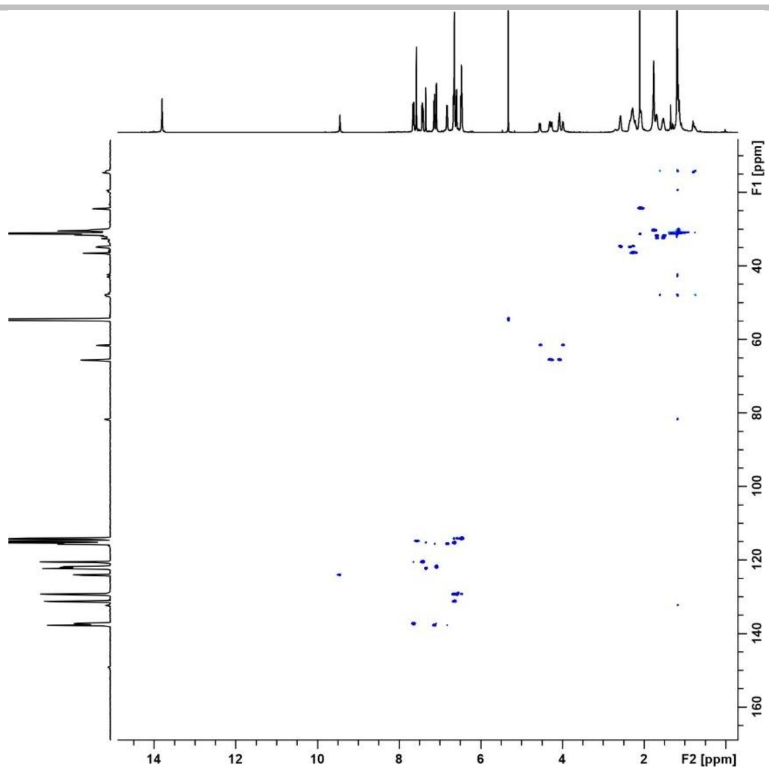

Figure S 70. The ^{1}H-^{13}C HSQC NMR spectrum of **$[10\text{-}H_2]^{2+}$** (600 MHz, $[D_2]$dichloromethane, 200 K).

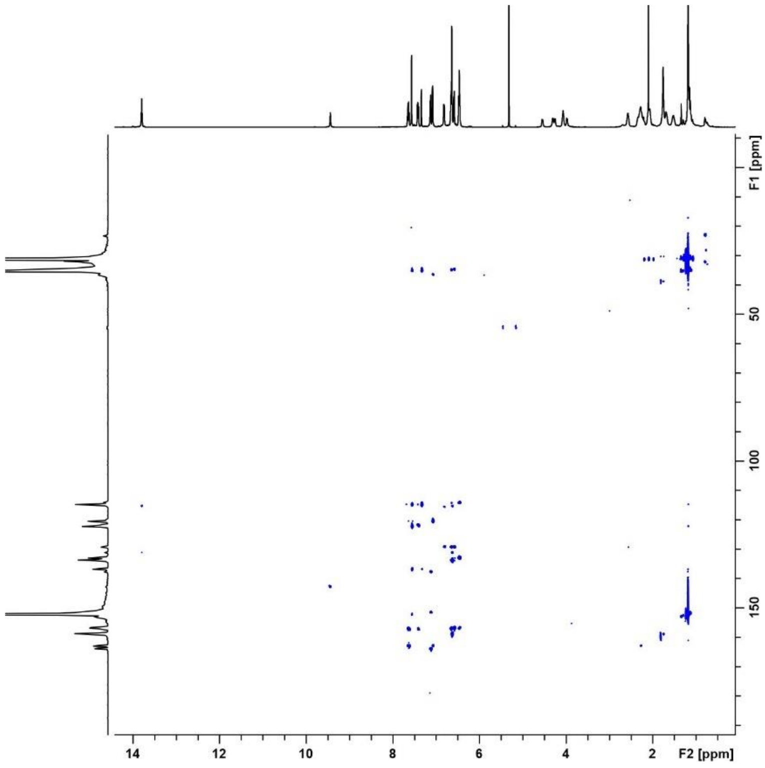

Figure S 71. The ^{1}H-^{13}H HMBC spectrum of **$[10\text{-}H_2]^{2+}$** (600 MHz, $[D_2]$dichloromethane, 200 K).

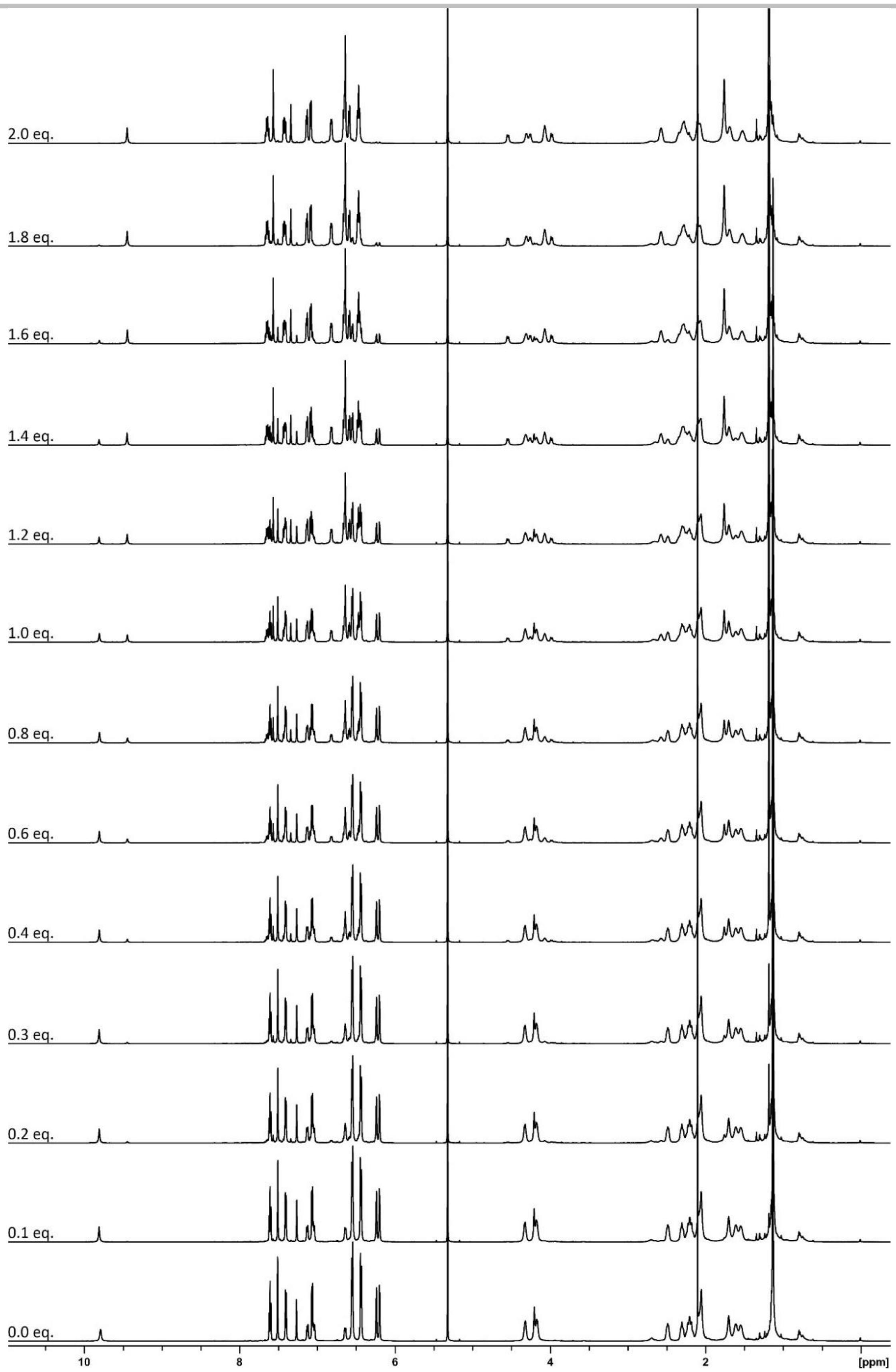

Figure S 72. The ^{1}H NMR spectra recorded during titration of **10** with trifluoroacetic acid (600 MHz, [D_2]dichloromethane, 200 K).

Compound [10-H$_4$]$^{4+}$

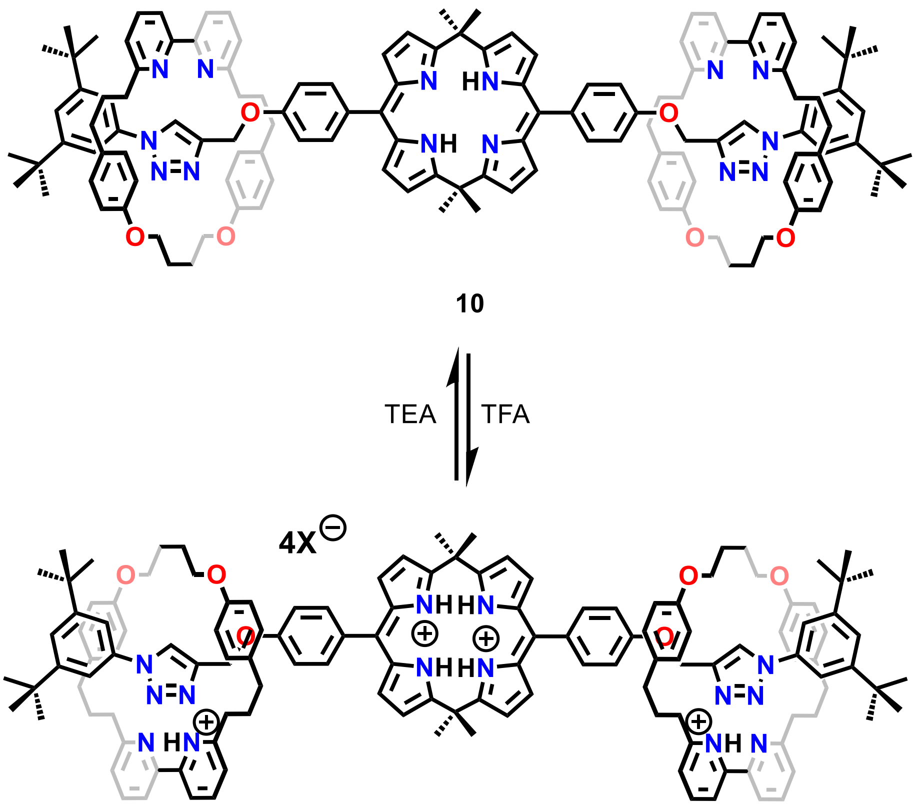

[10-H$_4$]$^{4+}$

Figure S 73. The cationic species **[10-H$_4$]$^{4+}$** was obtained by acidification of **10** with excess (ca. 5 equiv.) of trifluoroacetic acid in [D$_2$]dichloromethane.

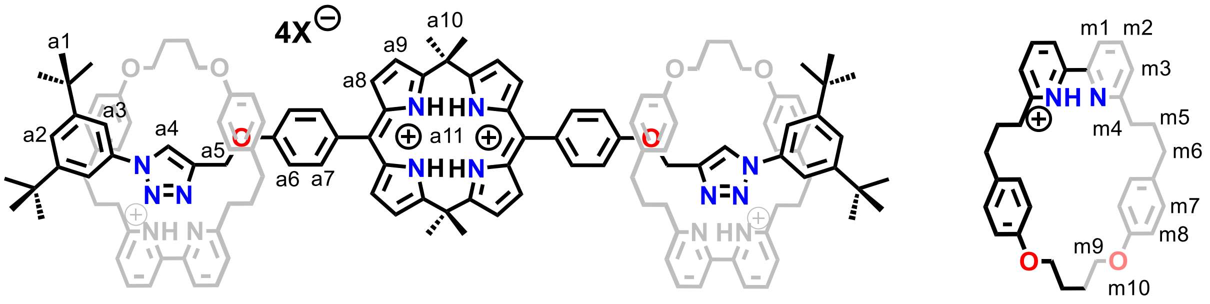

^{1}H NMR (600 MHz, [D$_2$]dichloromethane, 300 K, ppm): δ 13.25 (s, 4H, H$_{a11}$), 8.21 (t, ^{3}J = 8.0 Hz, 4H, H$_{m2}$), 8.06 (d, ^{3}J = 8.0 Hz, 4H, H$_{m1}$), 7.64 (t, ^{3}J = 1.7 Hz, 2H, H$_{a2}$), 7.61 (d, ^{3}J = 8.0 Hz, H4, H$_{m3}$), 7.52 (d, ^{3}J = 8.8 Hz, 4H, H$_{a7}$), 7.37 (d, ^{3}J = 1.7 Hz, 4H, H$_{a3}$), 6.92 (d, ^{3}J = 8.8 Hz, 4H, H$_{a6}$), 6.88 (dd, ^{3}J = 4.3 Hz, ^{4}J = 1.7 Hz, 4H, H$_{a8}$), 6.82–6.79 (m 10H, H$_{a4}$, H$_{m7}$), 6.78 (dd, ^{3}J = 4.3 Hz, ^{4}J = 1.7 Hz, 4H, H$_{a9}$), 6.65 (d, ^{3}J = 8.6 Hz, 8H, H$_{m8}$), 4.51 (s, 4H, H$_{a5}$), 4.19–4.08 (m, 8H, H$_{m9}$), 2.71–2.58 (m, 12H, H$_{m4}$, H$_{m6}$), 2.51–2.43 (m, 4H, H$_{m4}$), 2.26–2.17 (m, 4H, H$_{m10}$), 2.14–2.06 (m, 4H, H$_{m10}$), 1.89–1.82 (m, 8H, H$_{m5}$), 1.85 (s, 12H, H$_{a10}$), 1.43 (s, H36, H$_{a1}$).

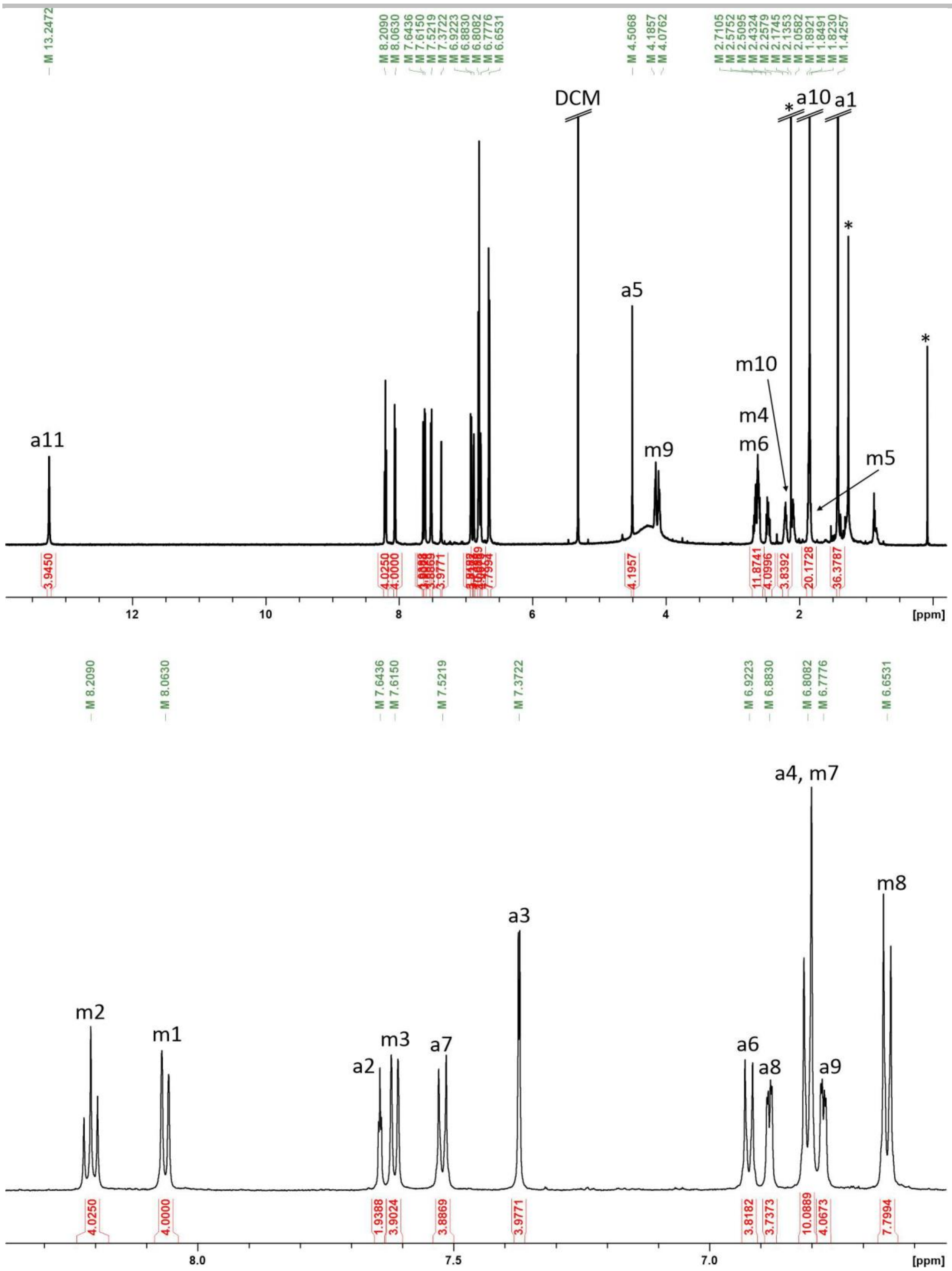

Figure S 74. The ^{1}H NMR spectrum of **$[10\text{-}H_4]^{4+}$** (600 MHz, $[D_2]$dichloromethane, 300 K).

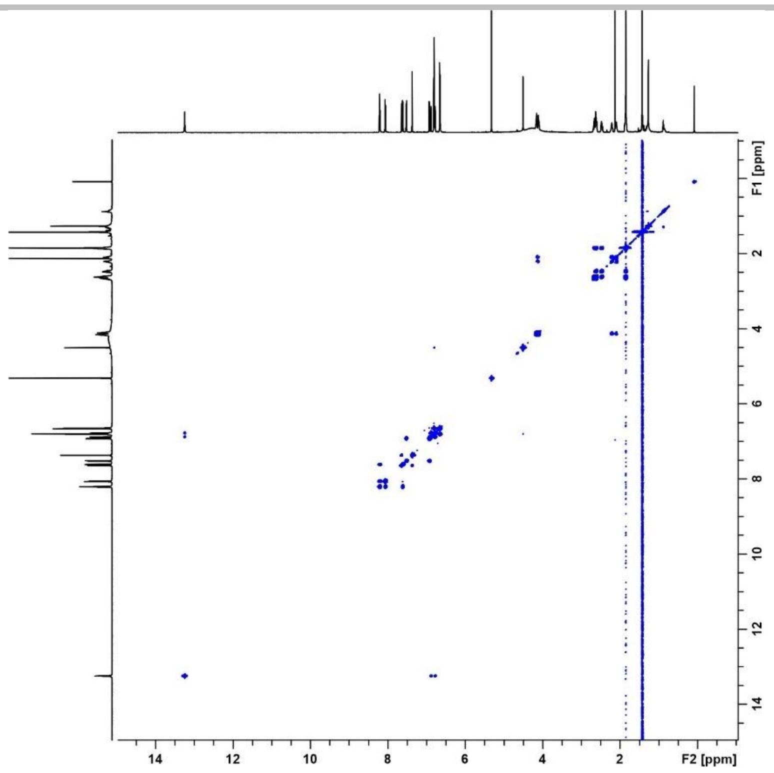

Figure S 75. The ^{1}H-^{1}H COSY NMR spectrum of **[10-H$_4$]$^{4+}$** (600 MHz, [D$_2$]dichloromethane, 300 K).

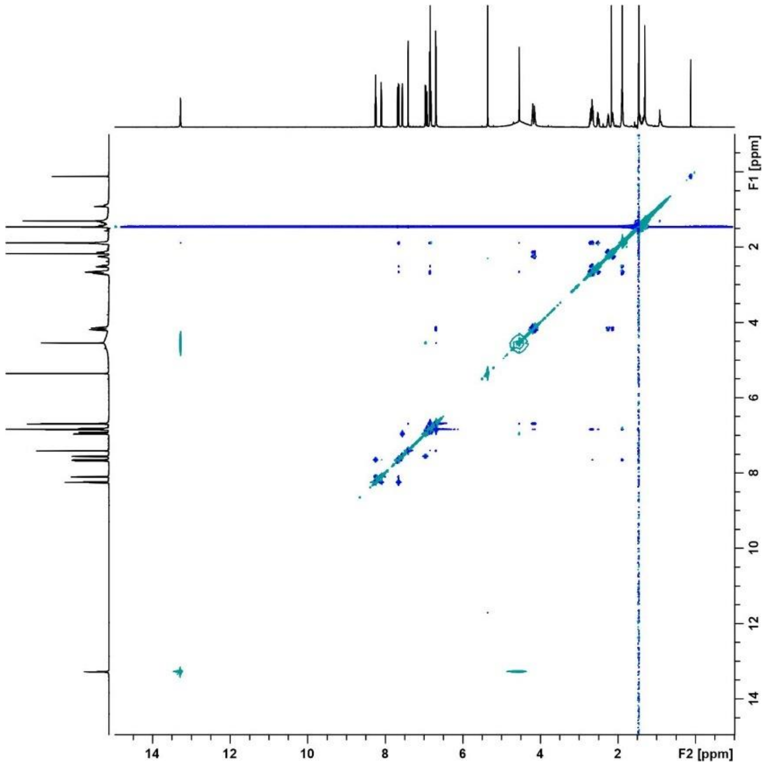

Figure S 76. The ^{1}H-^{1}H NOESY NMR spectrum of **[10-H$_4$]$^{4+}$** (600 MHz, [D$_2$]dichloromethane, 300 K).

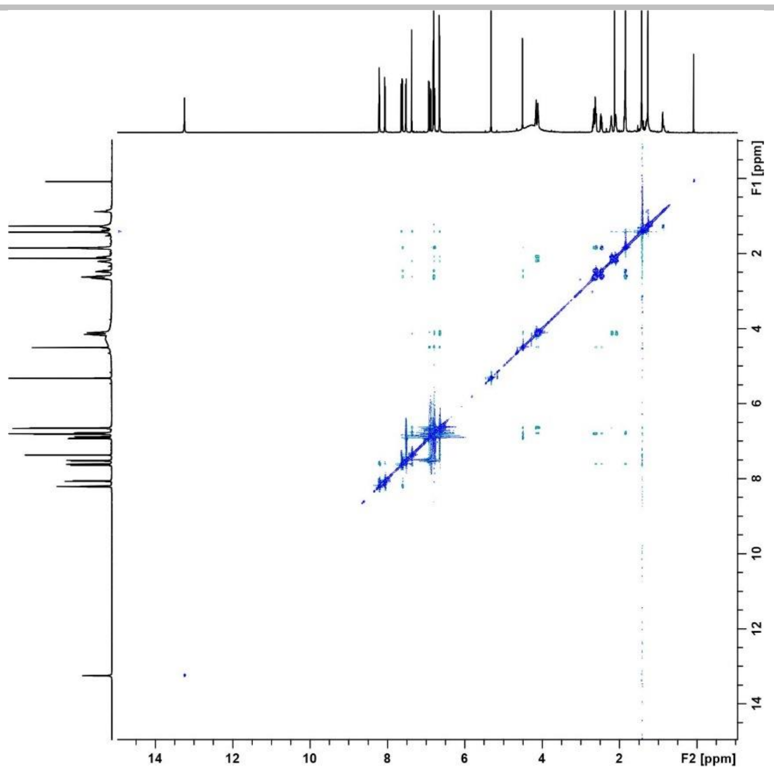

Figure S 77. The ^{1}H-^{1}H ROESY NMR spectrum of **[10-H$_4$]$^{4+}$** (600 MHz, [D$_2$]dichloromethane, 300 K).

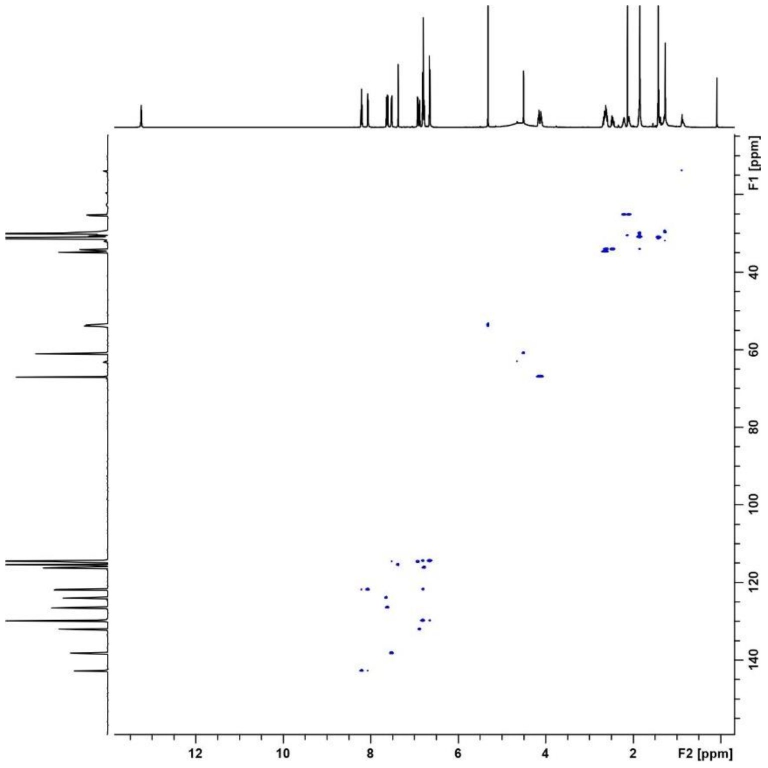

Figure S 78. The ^{1}H-^{13}C HSQC NMR spectrum of **[10-H$_4$]$^{4+}$** (500 MHz, [D$_2$]dichloromethane, 300 K).

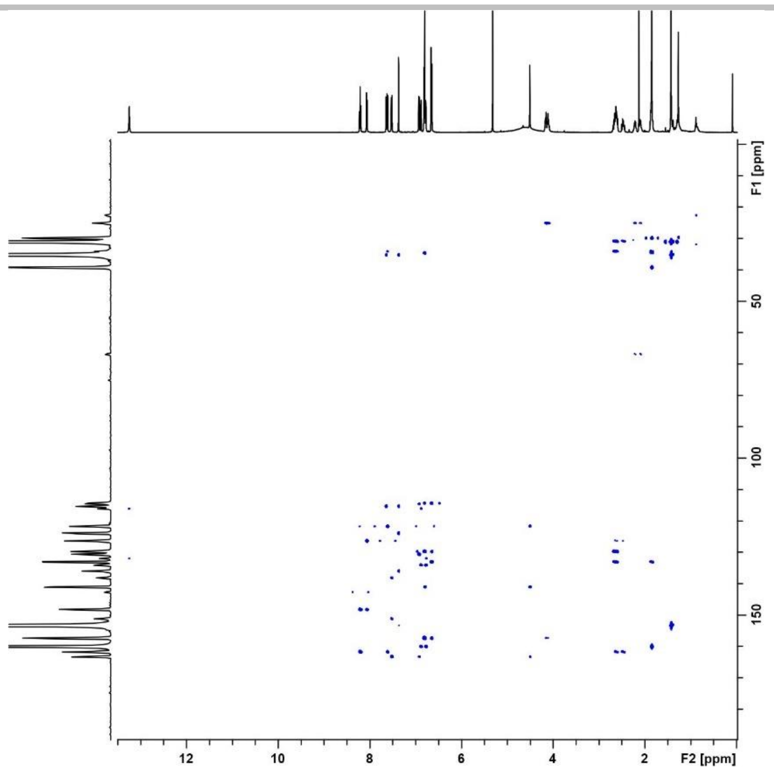

Figure S 79. The ^{1}H-^{13}C HMBC spectrum of **[10-H$_4$]$^{4+}$** (500 MHz, [D$_2$]dichloromethane, 300 K).

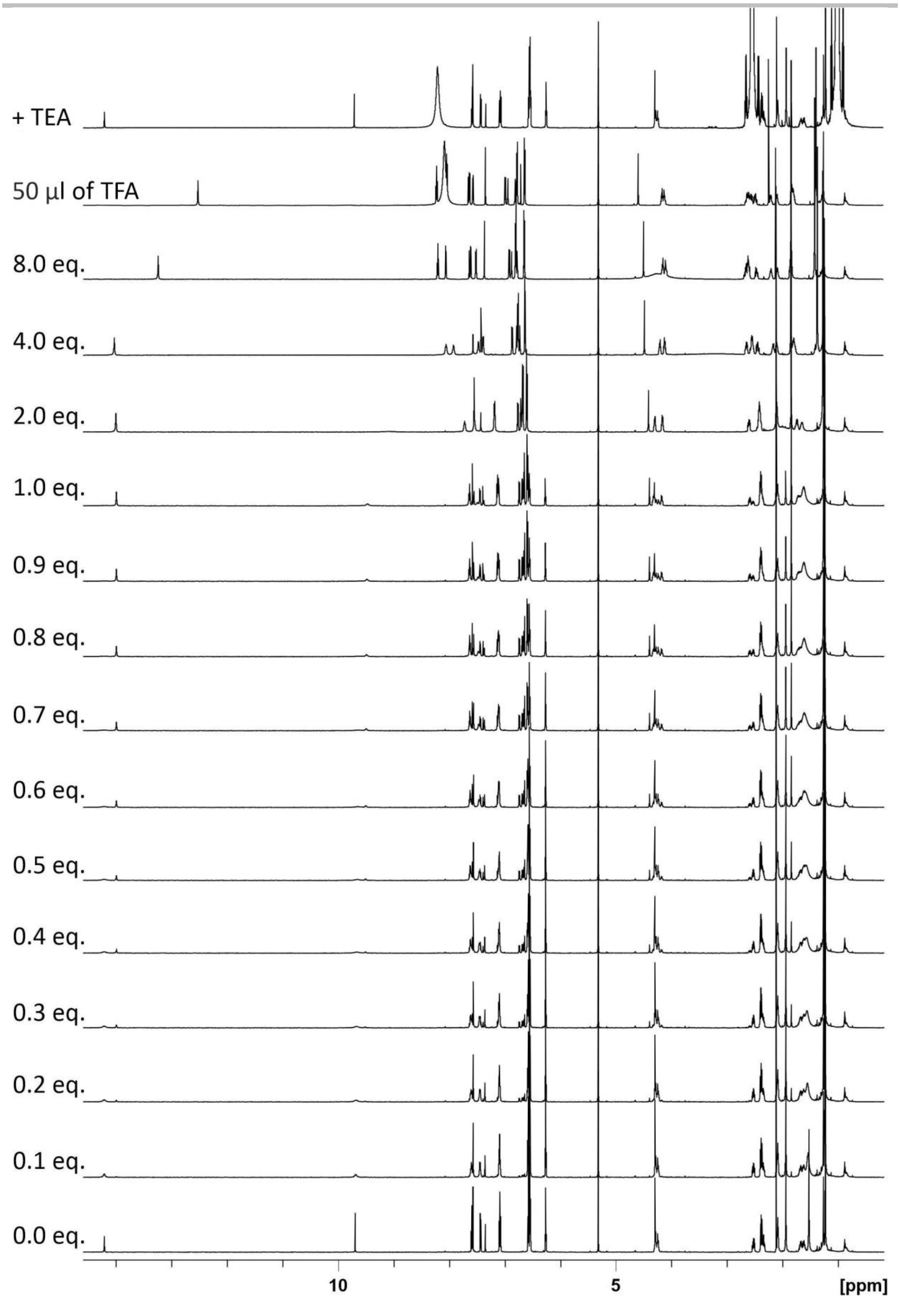

Figure S 80. The ^{1}H NMR spectra recorded during titration of **10** with trifluoroacetic acid (600 MHz, [D$_2$]dichloromethane, 300 K). The last spectrum was recorded upon the addition of TEA to **[10-H$_4$]$^{4+}$**.

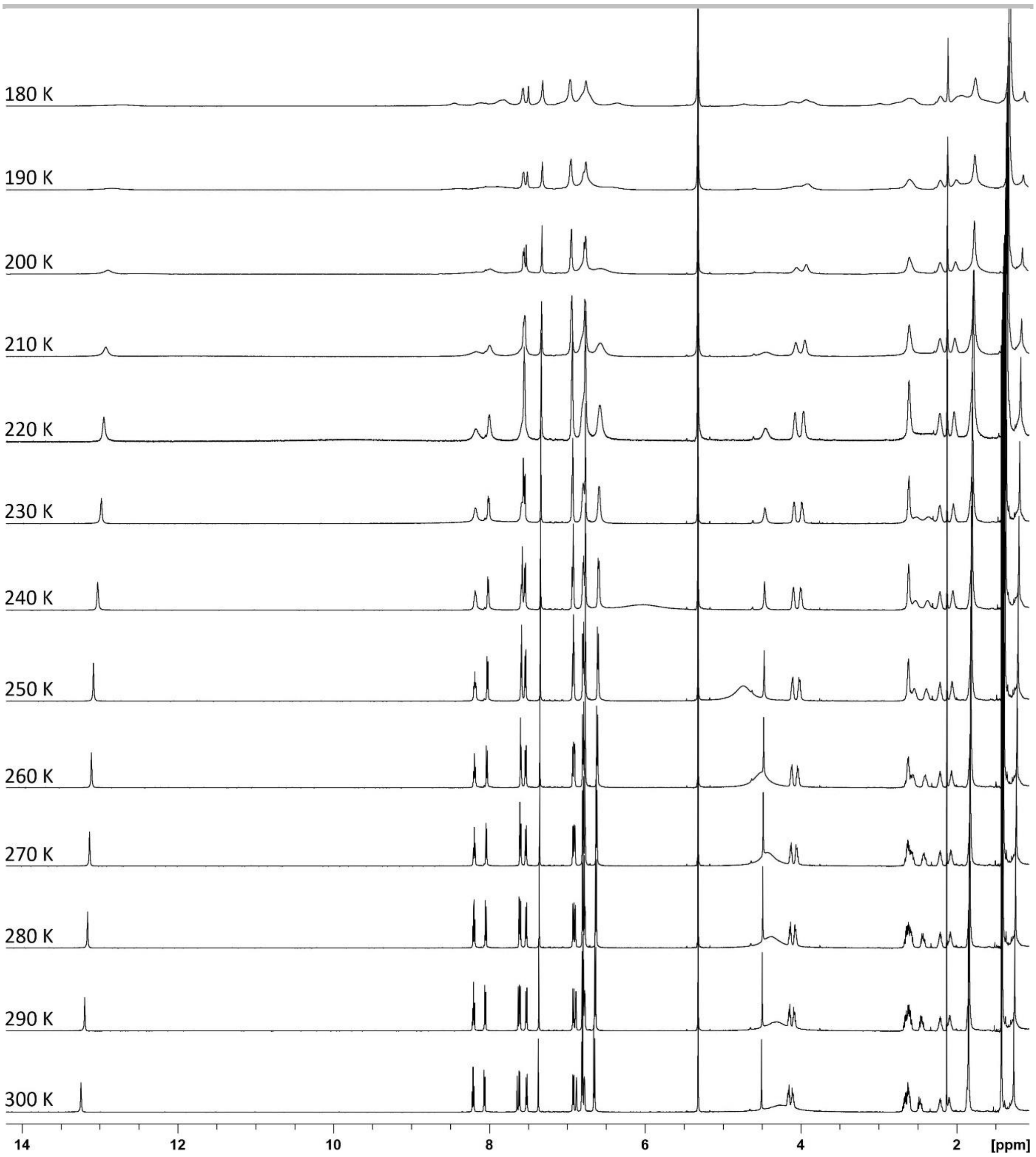

Figure S 81. The ^{1}H NMR spectra of **[10-H$_4$]$^{4+}$** recorded at the 300–180 K temperature range (600 MHz, [D$_2$]dichloromethane).

Compound 12

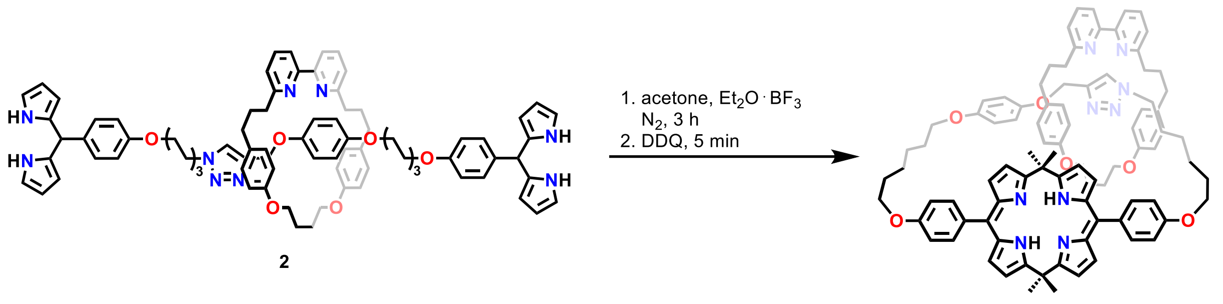

12; 31%

In a 50 ml round bottom flask, **2** (41 mg, 31.3 μmol) and acetone (25 ml) were introduced. The mixture was flushed via nitrogen bubbling under a sealed flask for 10 minutes. Then $Et_2O{\cdot}BF_3$ (10 μl, 81 μmol) was added via the syringe. The mixture was then stirred for 3 h under a nitrogen atmosphere. After this time septum was opened, DDQ (24.6 mg, 108 μmol) was introduced, and the reaction was carried out for an additional 5 min. Then, the acid was quenched by TEA (0.5 ml), and the mixture was passed through a short column with deactivated aluminum oxide. Residues on the column were washed out with ethyl acetate. The solvent was removed under reduced pressure. The reddish oil was purified via flash chromatography (DCM with 0-2% ethyl acetate gradient) and then recrystallised (DCM and methanol mixture) to provide **12** (13.5 mg, 9.7 μmol, 31%) as a red crystalline solid.

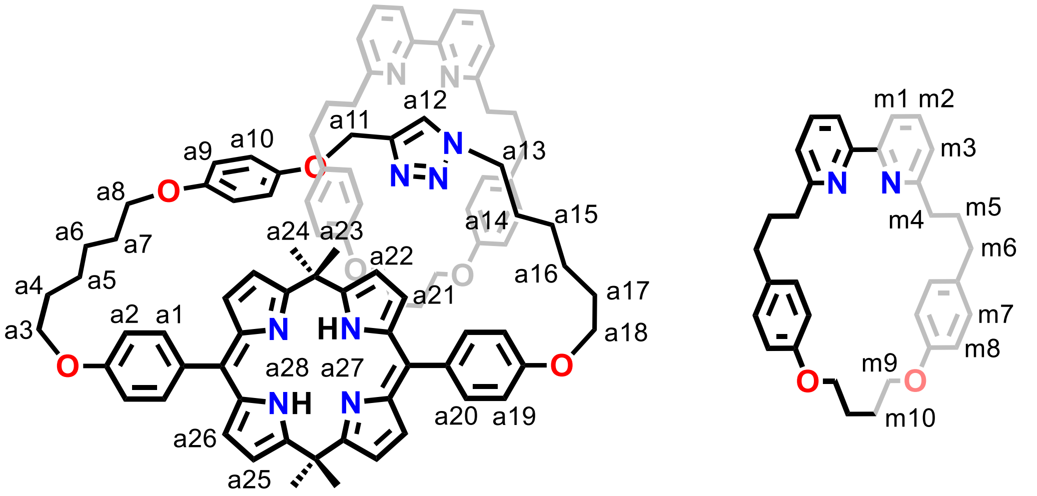

^{1}**H NMR** (600 MHz, [D_2]dichloromethane, 260 K, ppm): δ 14.55 (s, 1H, H_{a28}), 14.48 (s, 1H, H_{a27}), 8.13 (s, 1H, H_{a12}), 7.41 (t, ^3J= 7.7 Hz, 2H, H_{m2}), 7.35 (d, ^3J = 8.5 Hz, 1H, H_{a2}), 7.32 (d, ^3J = 8.5 Hz, 1H, H_{a2}), 7.27 (d, ^3J = 8.2 Hz, 1H, H_{a20}), 7.18 (d, ^3J = 7.7 Hz, 2H, H_{m1}), 7.00–6.97 (overlapped, 1H, H_{a20}), 6.97 (d, ^3J = 7.7 Hz, 2d, H_{m3}), 6.91 (d, ^3J = 8.5 Hz, 1H, H_{a1}), 6.87 (d, ^3J = 8.5 Hz, 1H, H_{a1}), 6.83 (d, ^3J = 8.5 Hz, 1H, H_{a19}), 6.73 (d, ^3J = 8.5 Hz, 4H, H_{m7}), 6.67 (d, ^3J = 9.1 Hz, 2H, H_{a9}), 6.62 (d, ^3J = 9.1 Hz, 2H, H_{a10}), 6.61 (d, ^3J = 8.5 Hz, 4H, H_{m8}), 6.42 (d, ^3J = 8.5 Hz, 1H, H_{a19}), 6.38 (d, ^3J = 4.2 Hz, 2H, H_{a26}), 6.29 (d, ^3J = 4.2 Hz, 2H, H_{a21}), 6.25 (d, ^3J = 4.2 Hz, 2H, H_{a25}), 6.20 (d, ^3J = 4.2 Hz, 2H, H_{a22}), 4.53(s, 2H, H_{a11}), 4.15–4.09 (m, 2H, H_{m9}), 4.07 (t, ^3J = 6.8 Hz, 2H, H_{a3}), 4.00–3.94 (m, 2H, H_{m9}), 3.81 (t, ^3J = 6.1 Hz, 2H, H_{a8}), 3.74 (t, ^3J = 6.9 Hz, 2H, H_{a18}), 3.39 (t, ^3J = 7.6 Hz, 2H, H_{a13}), 2.47 (t, ^3J = 6.1 Hz, 4H, H_{m6}), 2.42–2.30 (m, 4H, H_{m4}), 2.23 (s, 6H, H_{a23}), 2.05–1.95 (m, 2H, H_{m10}), 1.95–1.86 (m, 2H, H_{m10}) 1.79–1.74 (m, 2H, H_{a4}), 1.72 (s, 6H, H_{a24}), 1.74–1.54 (m, H6, H_{a7}, H_{m5}), 1.54–1.41 (m, 4H, H_{a5}, H_{a6}), 1.26–1.15 (m, 2H, H_{a17}), 0.88–0.70 (m, 4H, H_{a14}, H_{a16}), 0.67–0.59 (m, 2H, H_{a15}); ^{13}**C NMR** (151 MHz, [D_2]dichloromethane, 260 K, ppm): δ 165.4, 165.0, 162.2, 159.51, 159.46, 157.0, 156.9, 152.9, 152.1, 142.1, 140.13, 140.09, 139.8, 139.7, 136.5, 133.2, 132.5, 132.3, 132.2, 131.9, 129.2, 128.4, 128.1, 127.8, 127.7, 124.5, 121.2, 119.9, 115.5, 114.7, 114.6, 114.5, 114.3, 114.0, 113.8, 112.6, 111.7, 67.6, 67.5, 67.3, 65.9, 61.5, 49.4, 38.1, 36.5, 34.4, 32.0, 31.6, 29.6, 28.8, 27.9, 27.5, 25.8, 25.0, 24.8, 24.6, 24.3, 23.2; **HR–ESI–MS** (*m/z*): $[M+H]^+$ calcd. for $C_{89}H_{96}N_9O_6^+$, 1386.7478; found, 1386.7428.

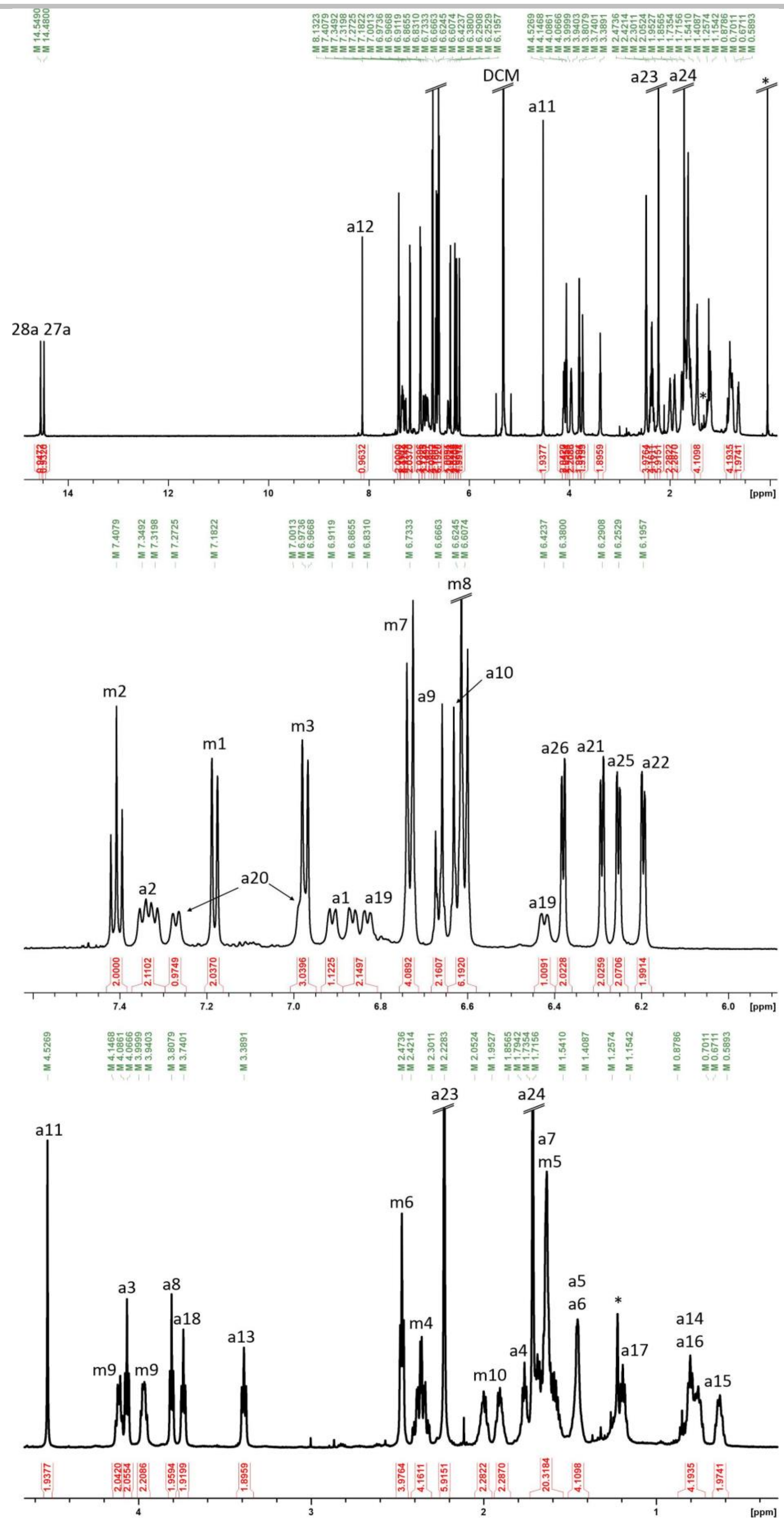

Figure S 82. The ^{1}H NMR spectrum of **12** (600 MHz, [D$_2$]dichloromethane, 260 K).

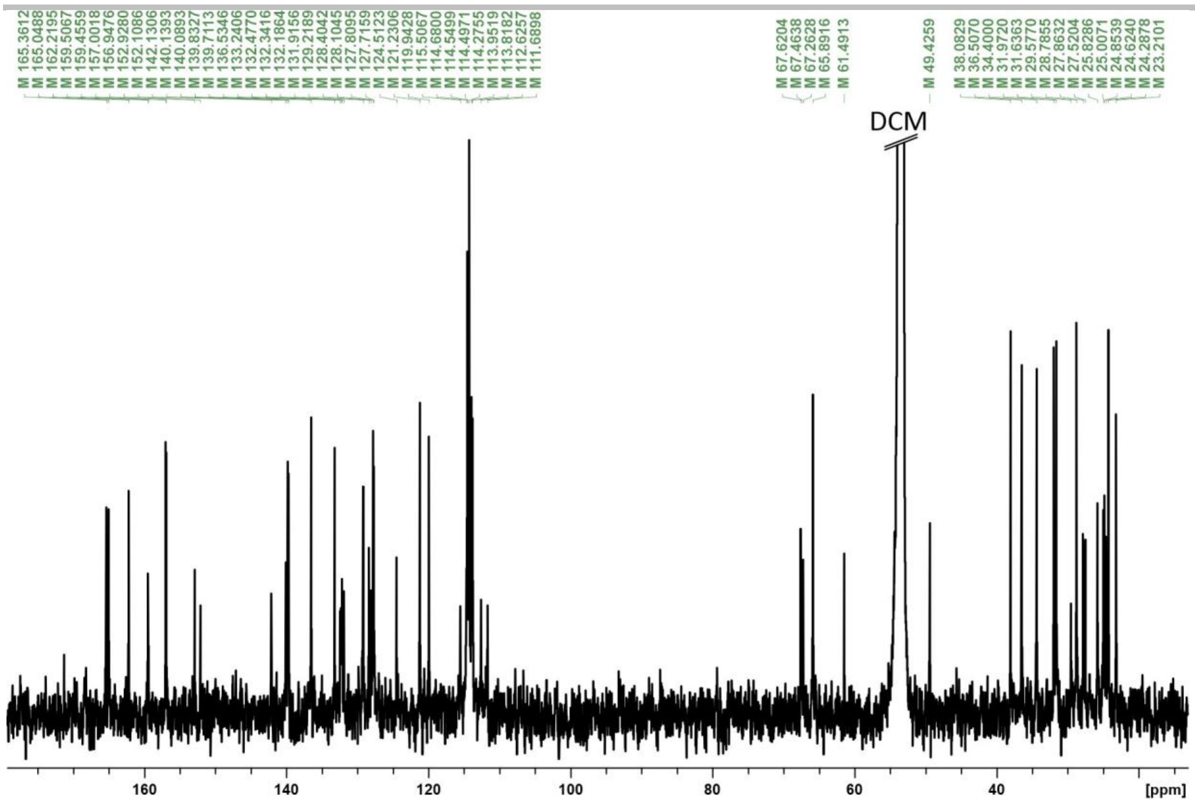

Figure S 83. The ^{13}C NMR spectrum of **12** (151 MHz, [D$_2$]dichloromethane, 260 K).

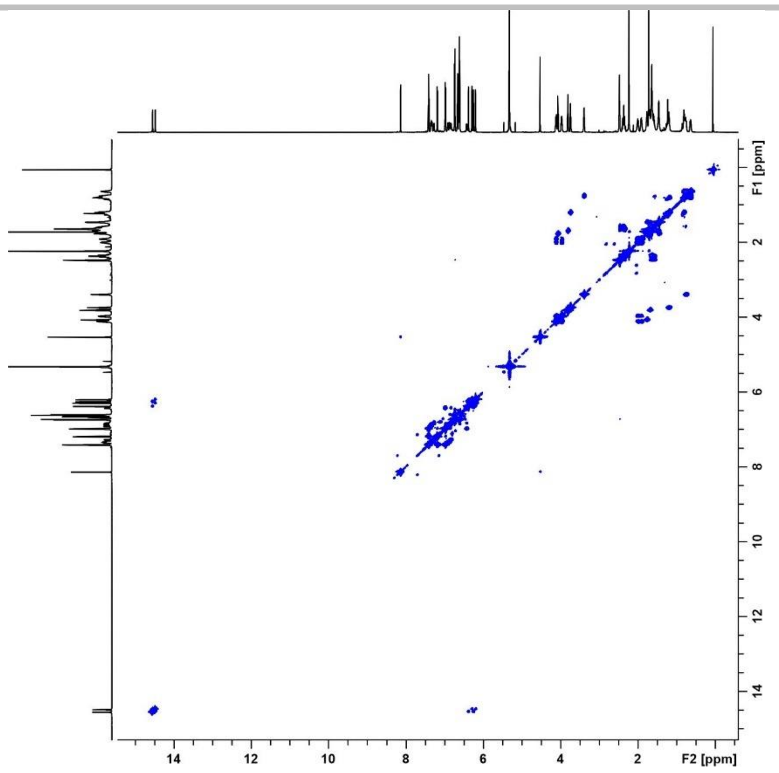

Figure S 84. The ^{1}H-^{1}H COSY NMR spectrum of **12** (600 MHz, [D_2]dichloromethane, 260 K).

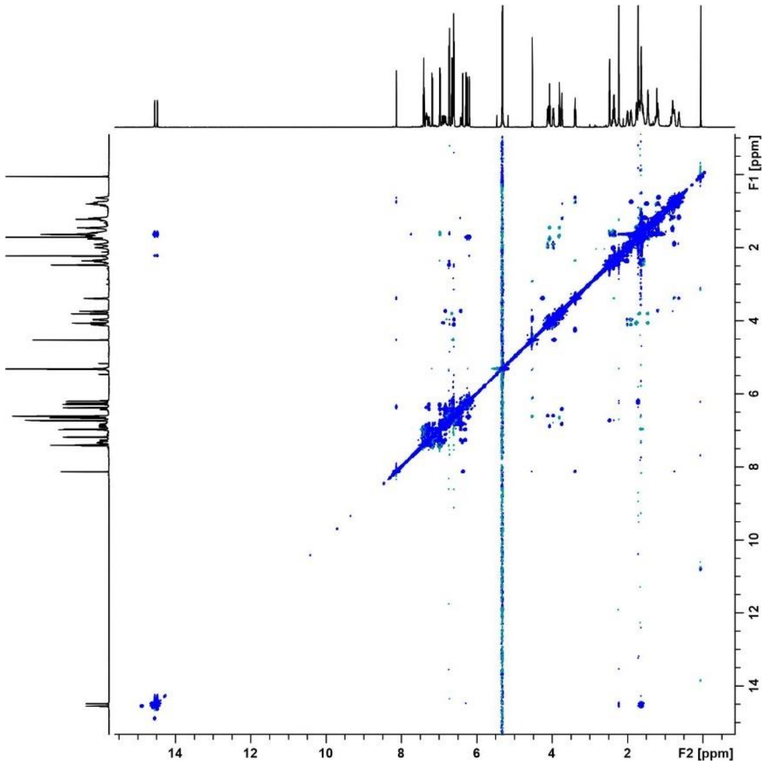

Figure S 85. The ^{1}H-^{1}H NOESY NMR spectrum of **12** (600 MHz, [D_2]dichloromethane, 260 K).

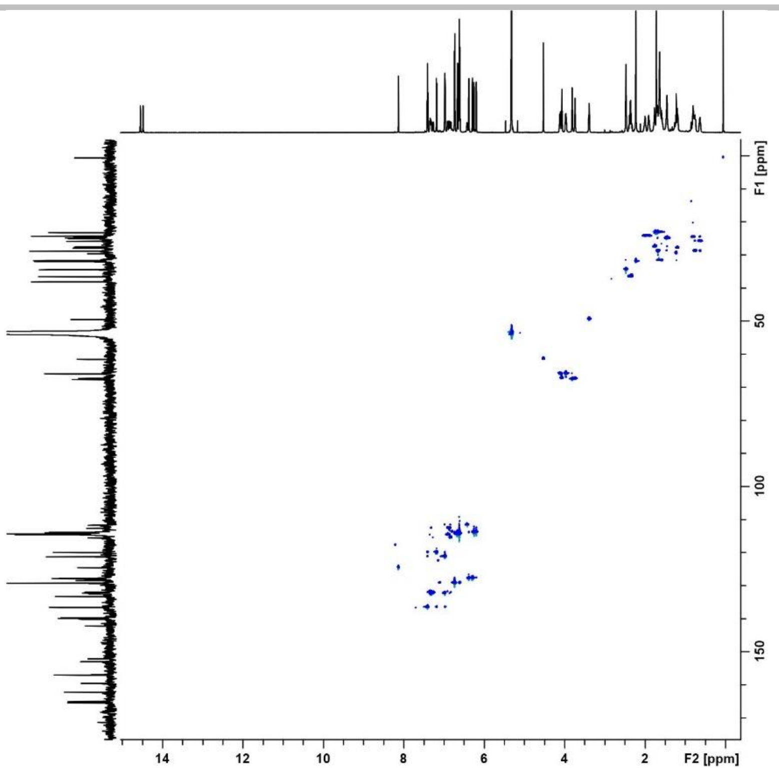

Figure S 86. The ^{1}H-^{13}C HSQC NMR spectrum of **12** (600 MHz, [D_2]dichloromethane, 260 K).

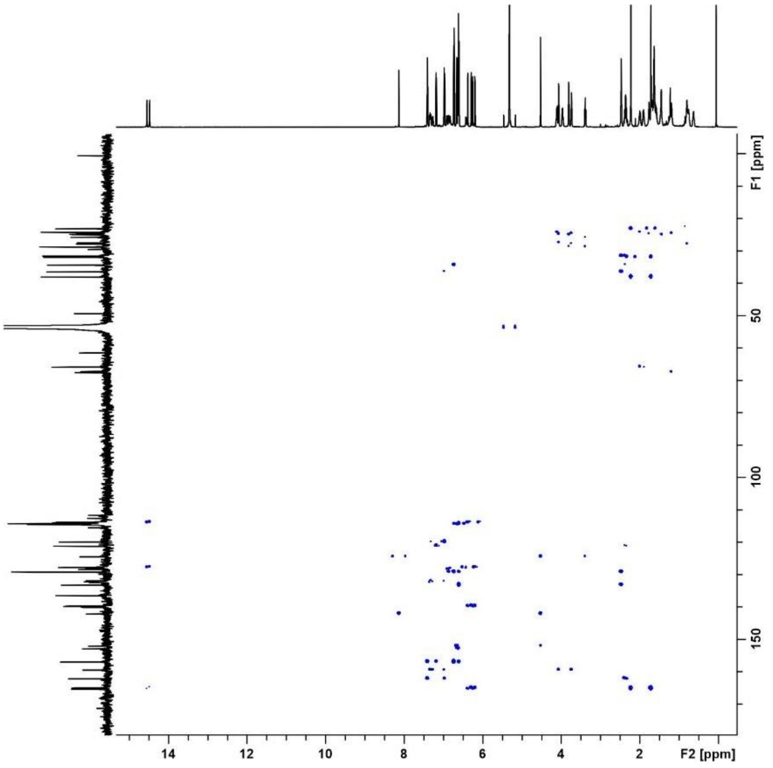

Figure S 87. The ^{1}H-^{13}C HMBC spectrum of **12** (600 MHz, [D_2]dichloromethane, 260 K).

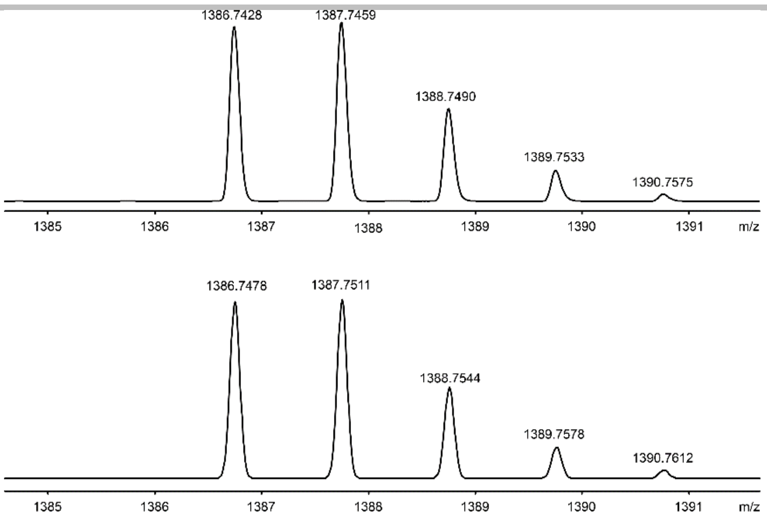

Figure S 88. The high-resolution ESI mass spectrum of **12**. Top: experimental spectrum, bottom: simulated isotopic pattern.

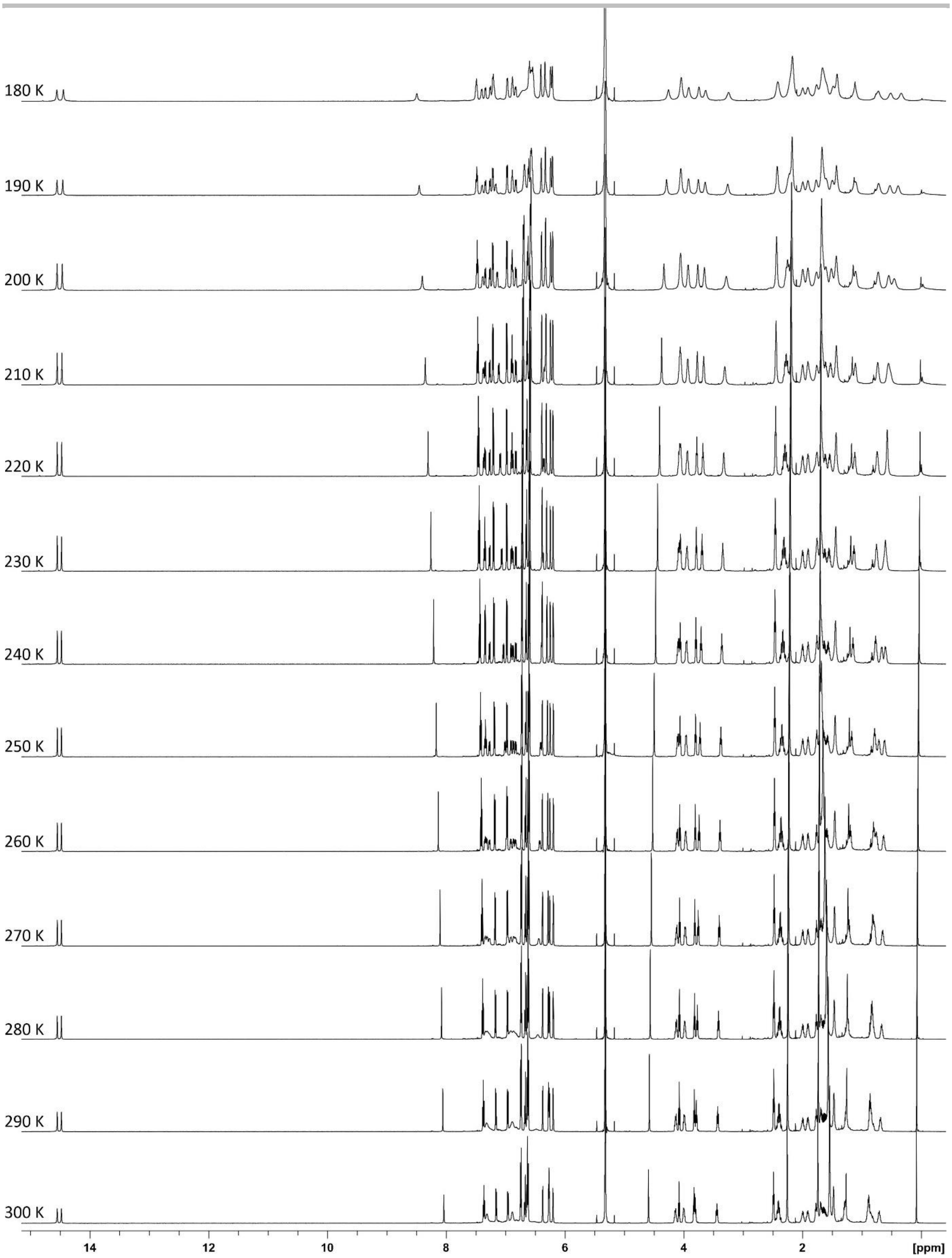

Figure S 89. The ^{1}H NMR spectra of **12** recorded at the 300–180 K temperature range (600 MHz, [D_2]dichloromethane).

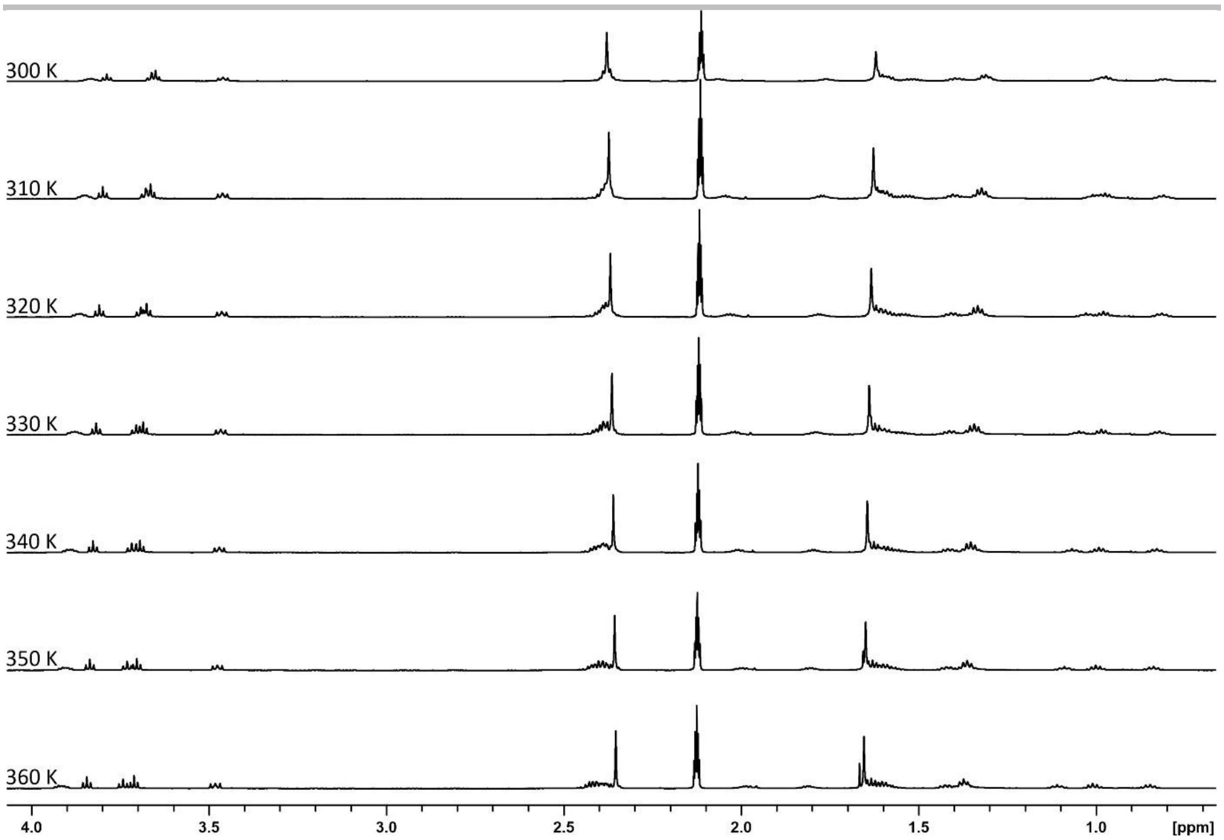

Figure S 90. The aliphatic region of the ^{1}H NMR spectra of **12** recorded at the 360–300 K temperature range (600 MHz, [D$_8$]toluene).

Compound [12-H]+

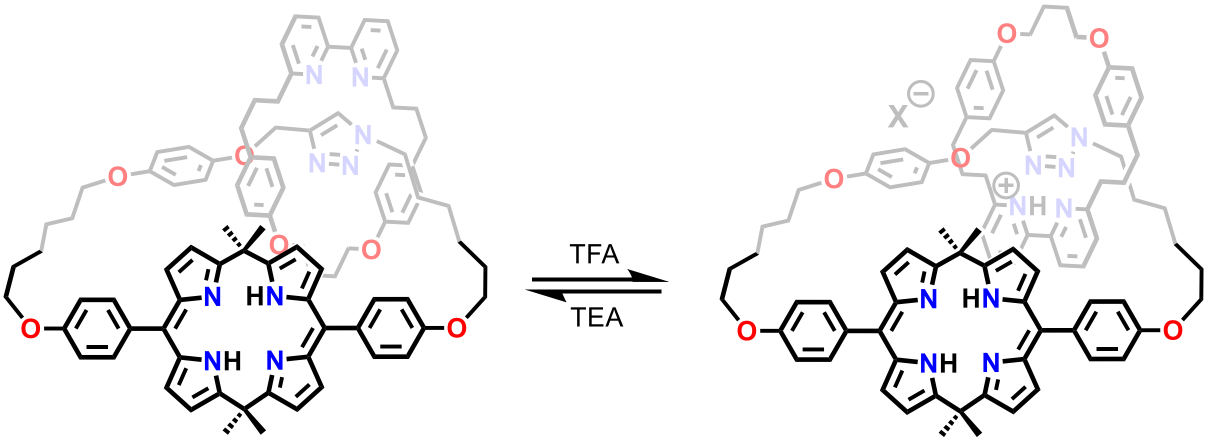

12 **[12-H]+**

The cationic species **[12-H]+** was obtained by the acidification of **12** with ca. one equiv. of trifluoroacetic acid in [D2]dichloromethane.

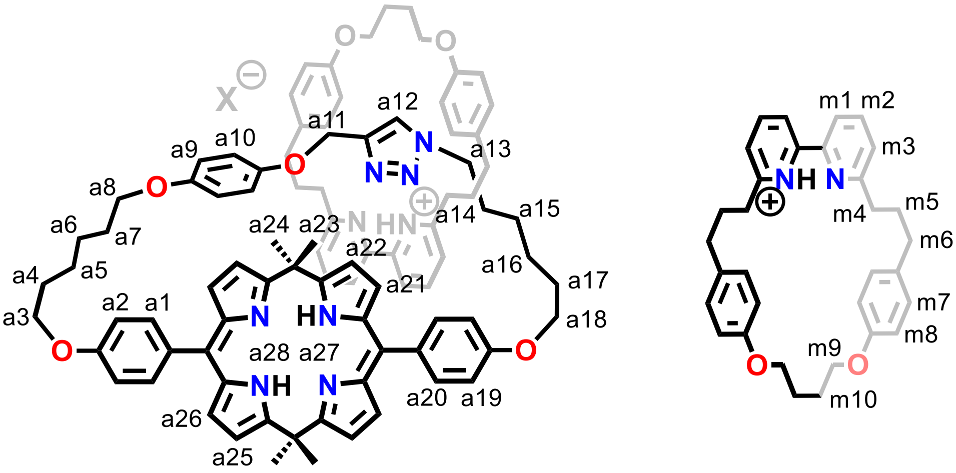

^{1}H NMR (600 MHz, [D2]dichloromethane, 300 K, ppm; *not all signals were identified*): δ 14.72 (broad s, 2H, H_{a27}), 7.32 (t, ^3J = 7.8 Hz, 2H, H_{m2}), 7.13 (d, ^3J = 7.8 Hz, 2H, H_{m1}), 7.09 (d, ^3J = 7.8 Hz, 2H, H_{m3}), 6.76 (d, ^3J = 8.5 Hz, 4H, H_{m7}), 6.66 (d, ^3J = 8.5 Hz, 4H, H_{m8}), 6.63 (d, ^3J = 9.0 Hz, 2H, H_{a9}), 6.44 (d, ^3J = 4.2 Hz, 2H, H_{a21}/H_{a26}), 6.39 (d, ^3J = 4.2 Hz, 2H, H_{a22}/H_{a25}), 6.28 (d, ^3J = 4.2 Hz, 2H, H_{a22}/H_{a25}), 6.23 (broad signal, 2H, H_{a10}), 6.22 (d, ^3J = 4.2 Hz, 2H, H_{a21}/H_{a26}), 4.12 (broad signal, 2H, H_{a13}) 4.08 (t, ^3J = 6.1 Hz, 2H, H_{a3}/ H_{a18}), 4.06–3.97 (m, 6H, H_{m9}, H_{a11}), 3.92 (t, 2H, ^3J = 7.0, 2H, H_{a3}), 3.88 (t, ^3J = 5.9 Hz, 2H, H_{a8}), 2.56–2.46 (m, 2H, H_{m6}), 2.46–2.32 (m, 6H, H_{m4}, H_{m6}), 2.30 (s, 6H, H_{a23}), 2.25–2.15 (m, 2H, H_{m4}), 2.11–1.93 (m, 4H, H_{m10}), 1.81 (s, 6H, H_{a24}), 1.86–1.35 (aliphatic region – signals overlapped).

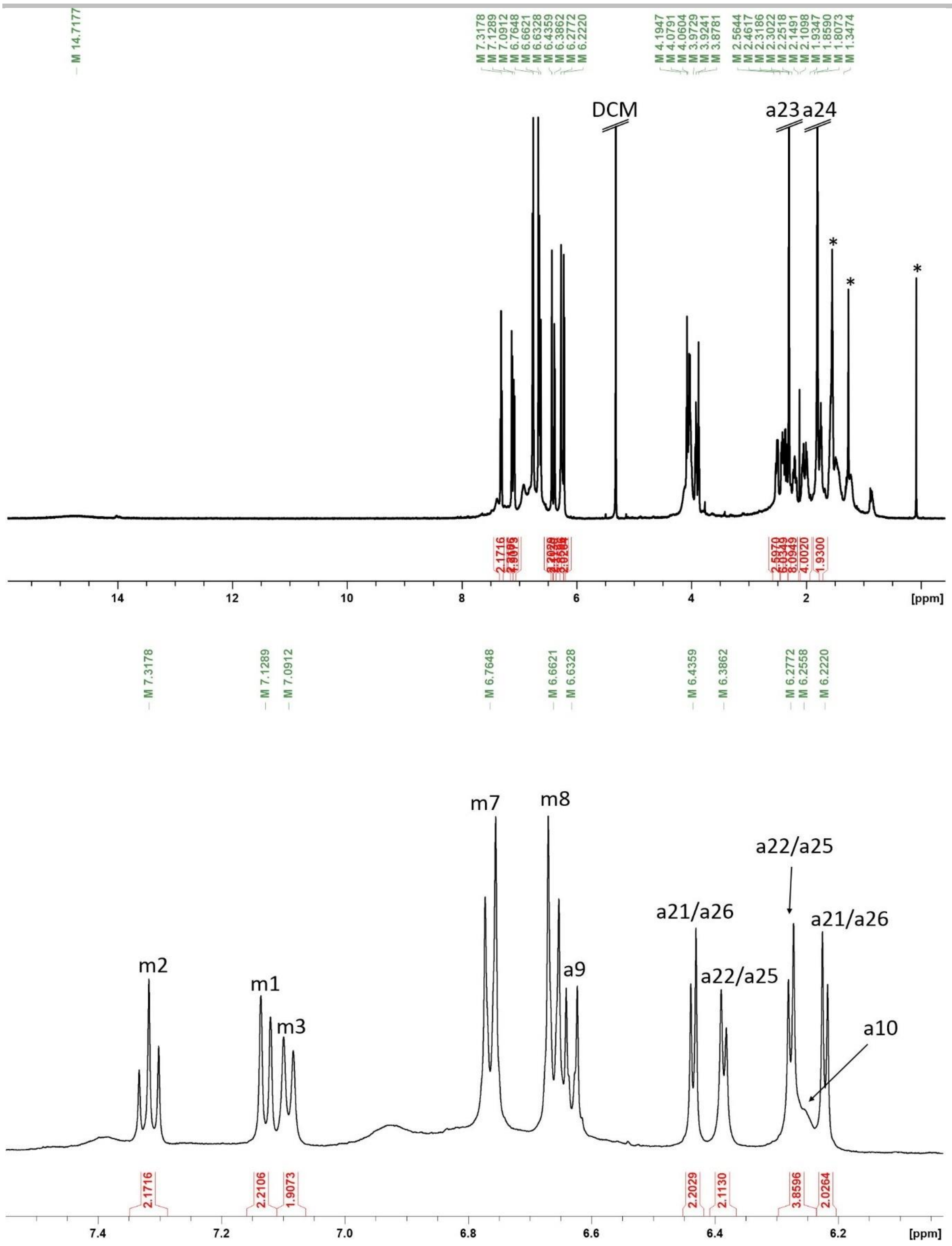

Figure S 91. The ^{1}H NMR spectrum of **[12-H]**$^{+}$ (600 MHz, [D_2]dichloromethane, 300 K).

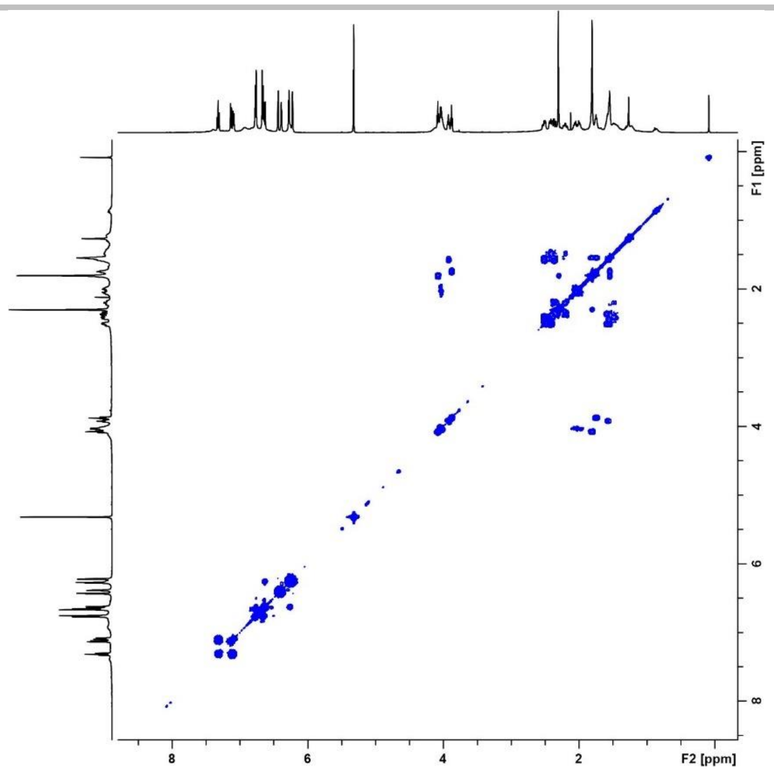

Figure S 92. The ^{1}H-^{1}H COSY NMR spectrum of **[12-H]**$^{+}$ (600 MHz, [D_2]dichloromethane, 300 K).

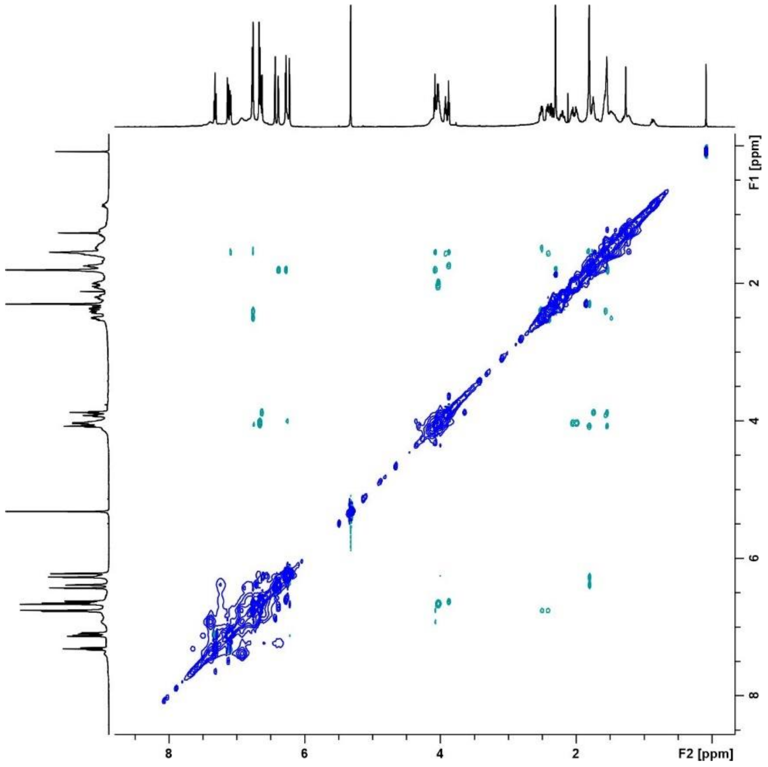

Figure S 93. The ^{1}H-^{1}H NOESY NMR spectrum of **[12-H]**$^{+}$ (600 MHz, [D_2]dichloromethane, 300 K).

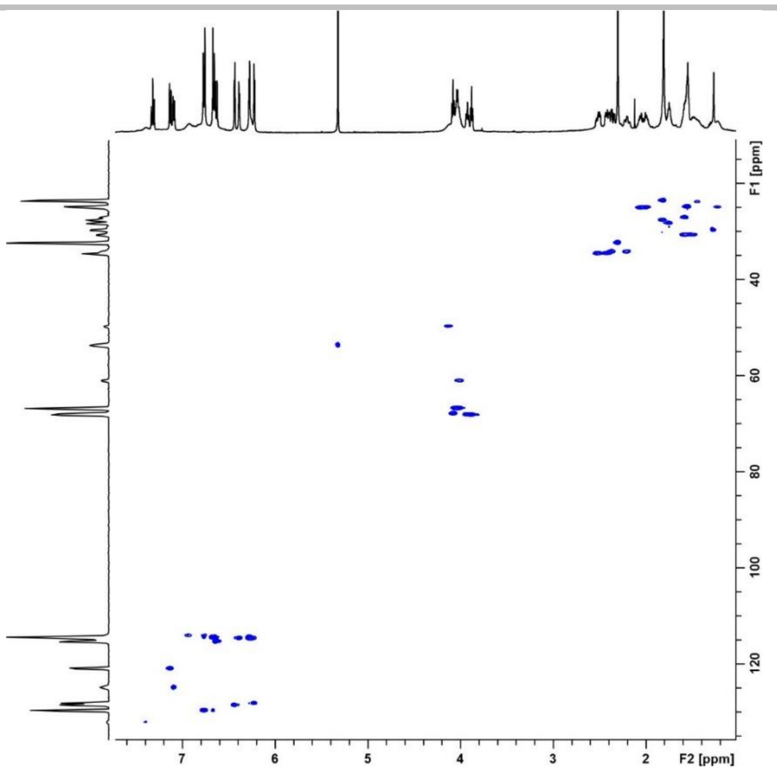

Figure S 94. The ^{1}H-^{13}C HSQC NMR spectrum of **[12-H]$^{+}$** (600 MHz, [D_2]dichloromethane, 300 K).

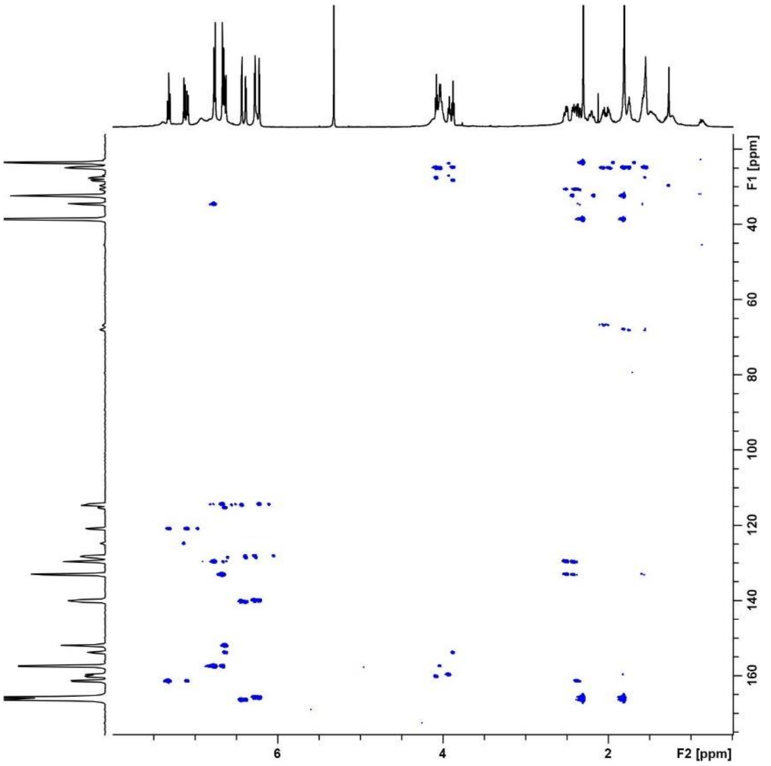

Figure S 95. The ^{1}H-^{13}C HMBC NMR spectrum of **[12-H]$^{+}$** (600 MHz, [D_2]dichloromethane, 300 K).

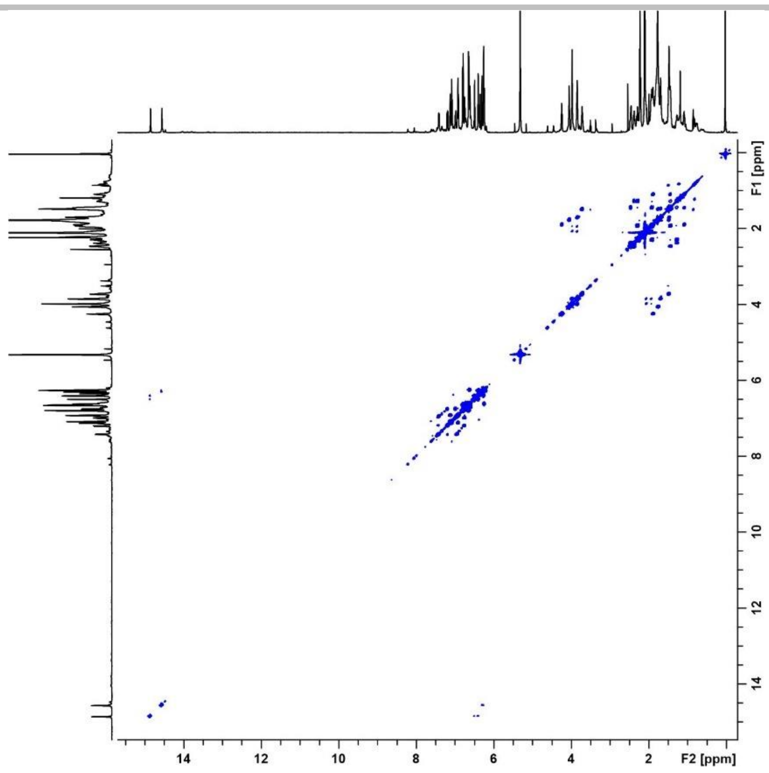

Figure S 96. The ^{1}H-^{1}H COSY NMR spectrum of **[12-H]$^+$** (600 MHz, [D$_2$]dichloromethane, 235 K).

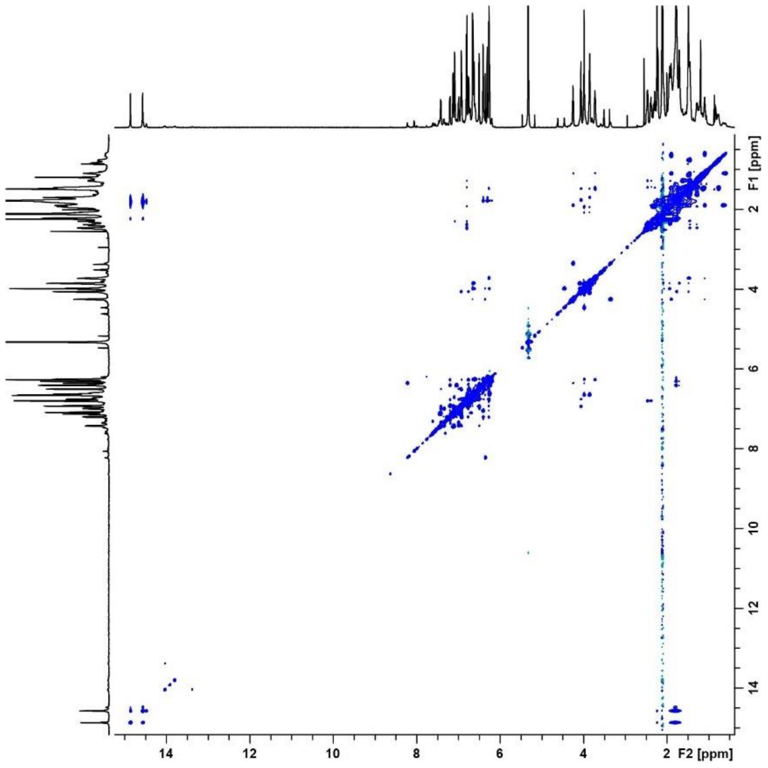

Figure S 97. The ^{1}H-^{1}H NOESY NMR spectrum of **[12-H]$^+$** (600 MHz, [D$_2$]dichloromethane, 235 K).

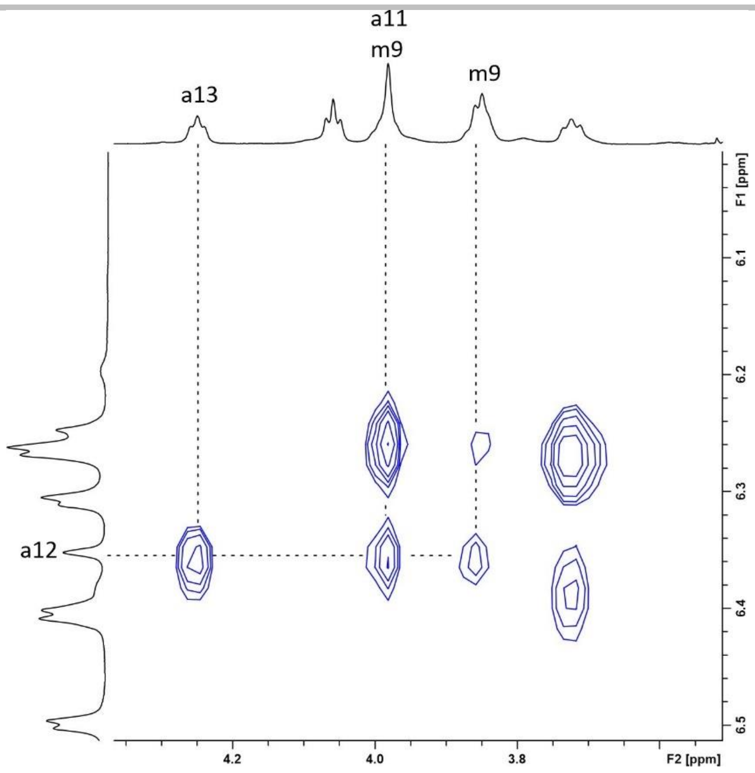

Figure S 98. The ^{1}H-^{1}H NOESY NMR spectrum of **[12-H]⁺** (600 MHz, [D$_2$]dichloromethane, 235 K). Highlighted NOE interactions between a12 and a11, a13 and m9.

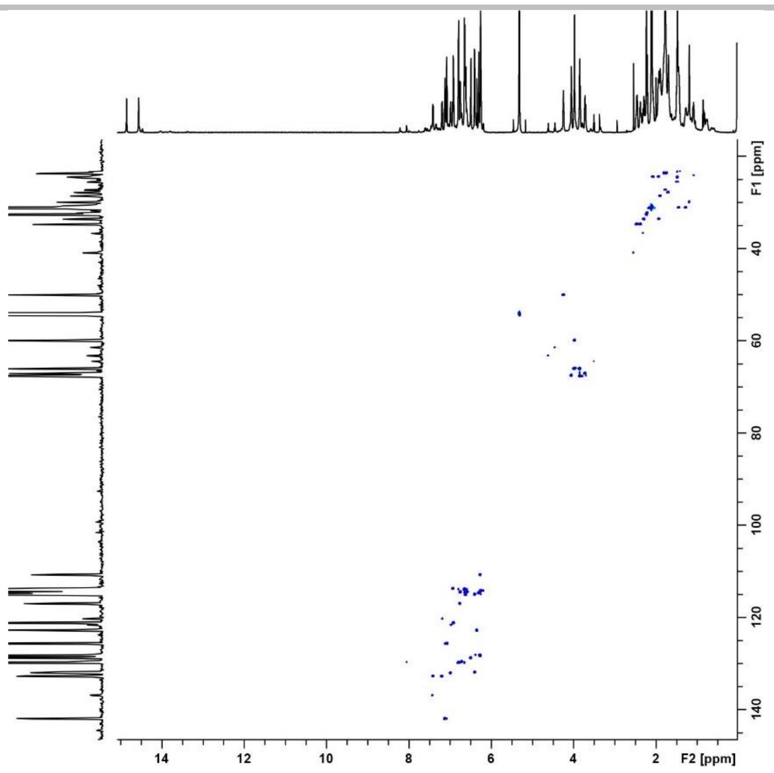

Figure S 99. The ^{1}H-^{13}C HSQC NMR spectrum of **[12-H]$^{+}$** (600 MHz, [D_2]dichloromethane, 235 K).

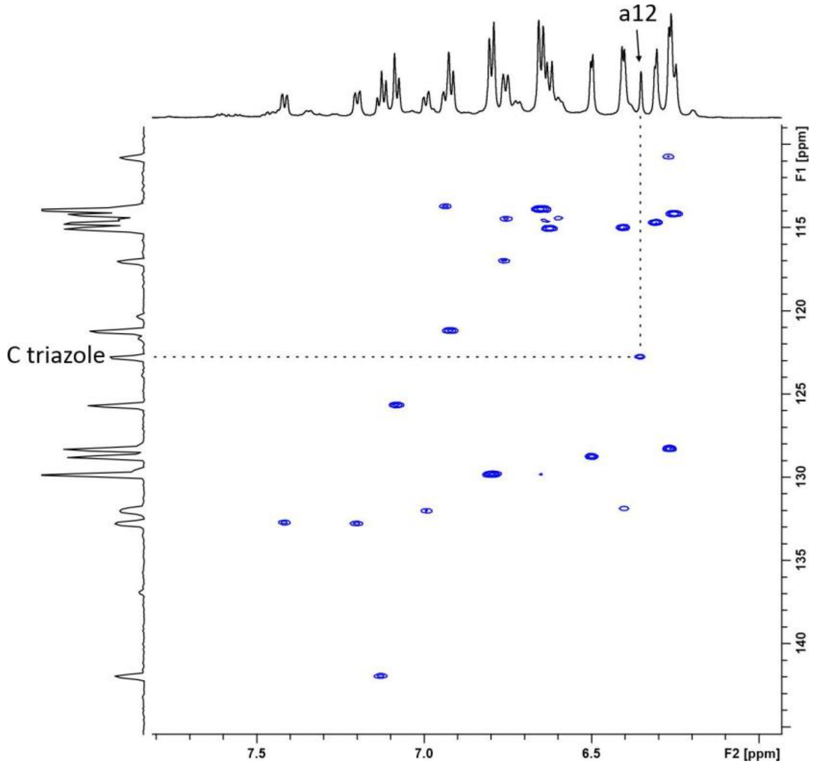

Figure S 100. The ^{1}H-^{13}C HSQC NMR spectrum of **[12-H]$^{+}$** (600 MHz, [D_2]dichloromethane, 235 K). The correlation marked with a dashed line demonstrates the a12(H)···C(triazole) coupling.

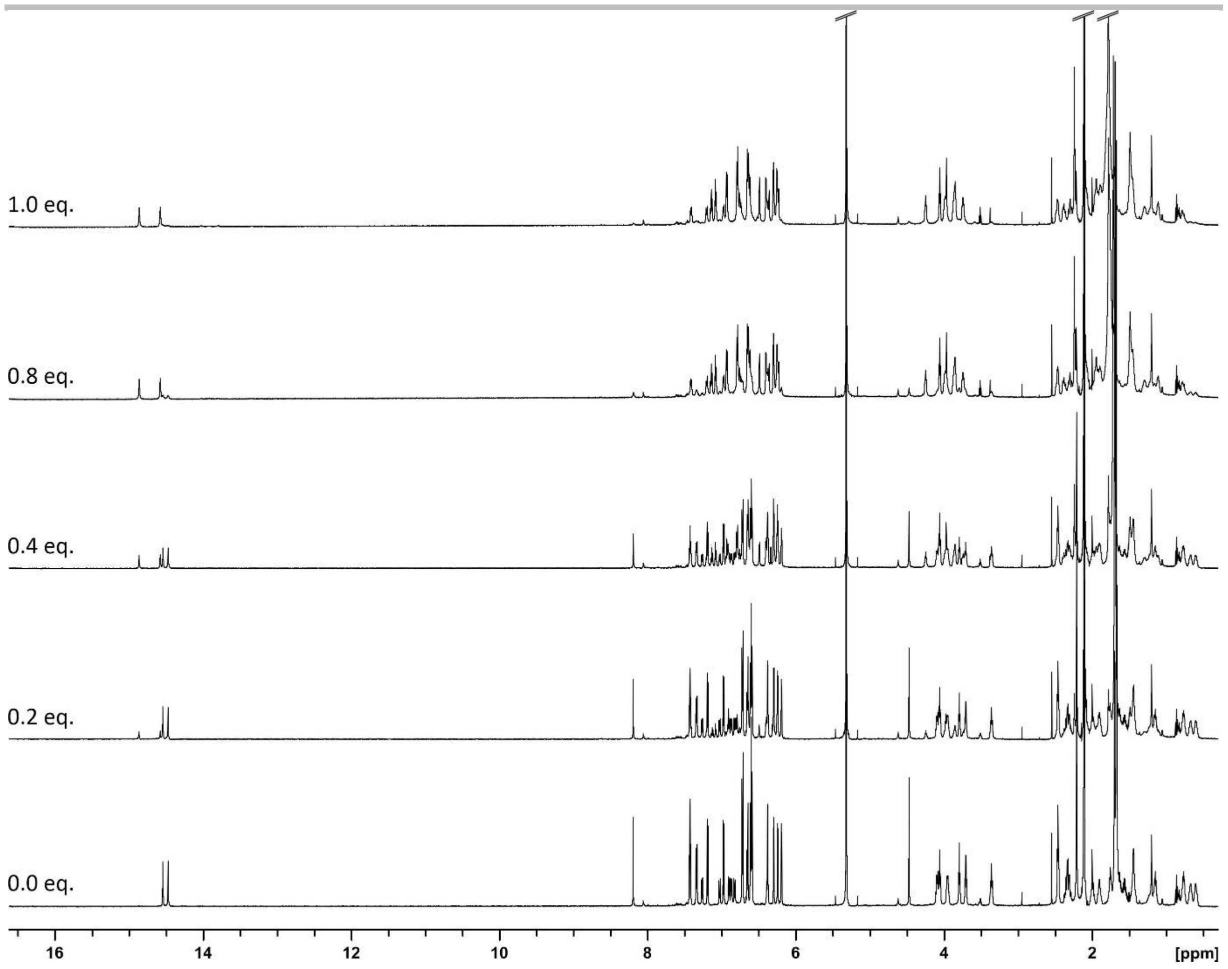

Figure S 101. The ^{1}H NMR spectra recorded during titration of **12** with trifluoroacetic acid (600 MHz, [D$_2$]dichloromethane, 240 K).

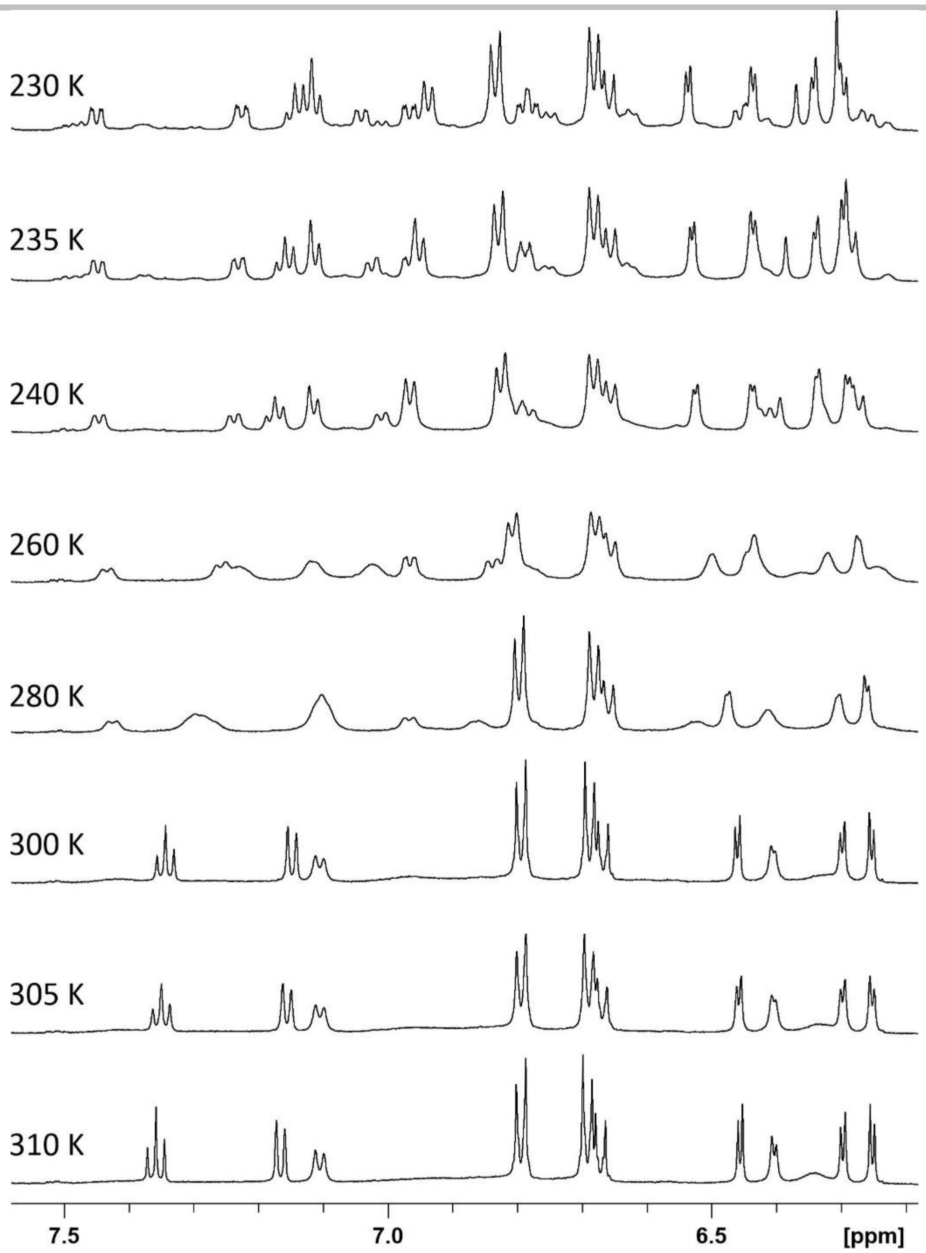

Figure S 102. The aromatic region of the ^{1}H NMR spectra of **[12-H]⁺** recorded at the 310–230 K temperature range (600 MHz, [D_2]dichloromethane).

Compound [12-H_3]$^{3+}$

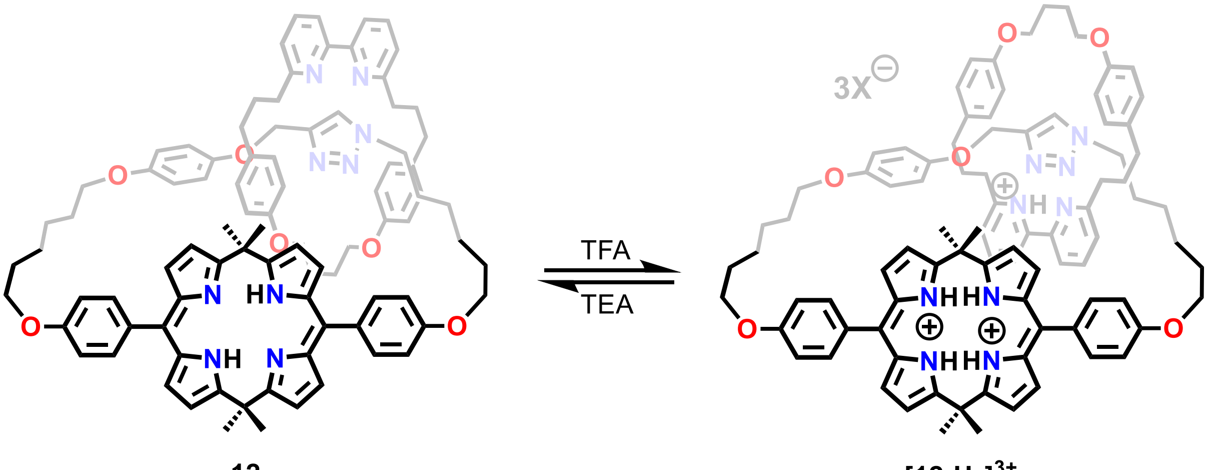

12 **[12-H_3]$^{3+}$**

The cationic species **[12-H_3]$^{3+}$** was obtained by acidification of **12** with ca. five equiv. of trifluoroacetic acid in [D_2]dichloromethane.

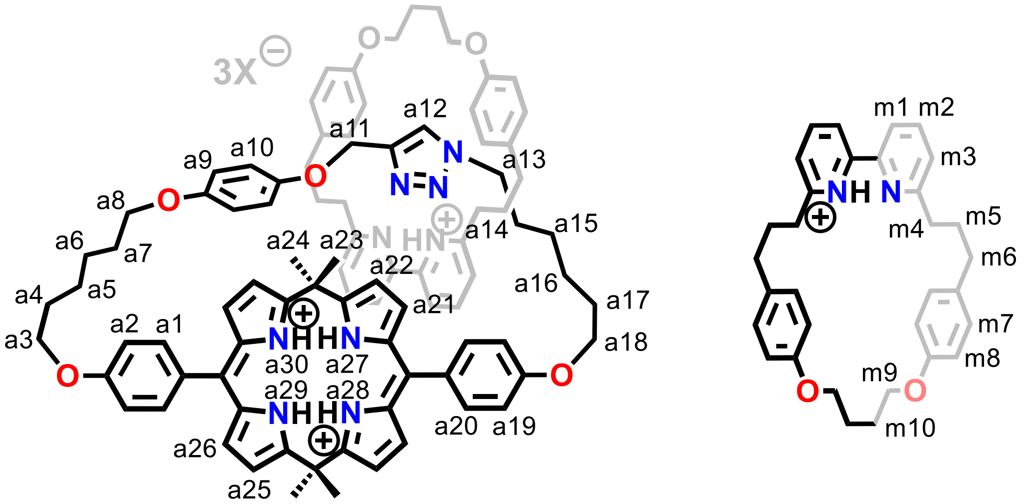

^{1}H NMR (600 MHz, [D_2]dichloromethane, 300 K, ppm): δ 13.00 (broad s, 4H, H_{a27} H_{a28}, H_{a29} H_{a30}), 8.06 (t, ^3J = 8.0 Hz, 2H, H_{m2}), 7.92 (d, ^3J =8.0 Hz, 2H, H_{m1}), 7.54–7.44 (m, 6H, H_{a1}, H_{a20}, H_{m3}), 7.09 (overlapped d, 4H, H_{a2}, H_{a19}), 6.94 (b, 2H, H_{a21}/H_{a26}), 6.70 (d, ^3J = 4.3 Hz, 2H, H_{a22}/H_{a25}), 6.67–6.61 (m, 6H, H_{a9}, H_{m7}), 6.54 (d, ^3J = 8.5 Hz, 4H, H_{m8}), 6.40 (d, ^3J = 9.1 Hz, 4H, Ha_{10}), 6.21 (s, 1H, H_{a12}), 4.40 (t, ^3J = 5.8 Hz, 2H, H_{a3}/H_{a18}), 4.35 (t, ^3J = 6.20 Hz, 2H, H_{a3}/H_{a18}), 4.01 (t, ^3J = 7.1 Hz, 2H, H_{a13}), 3.98 (s, 2H, H_{a11}), 3.93–3.84 (m, 4H, H_{m9}), 3.65 (t, ^3J = 7.0 Hz, 2H, H_{a8}), 2.55–2.22 (m, 8H, H_{m4}, H_{m6}), 2.00–1.89 (m, 2H, H_{m10}), 1.89–1.72 (m, 6H, H_{m10}, H_{a4}, H_{a17}), 1.81 (s, 12H, H_{a23}, H_{a24}), 1.72–1.18 (aliphatic region, H_{a5}, H_{a6}, H_{a7}, H_{a14}, H_{a15}, H_{a16}).

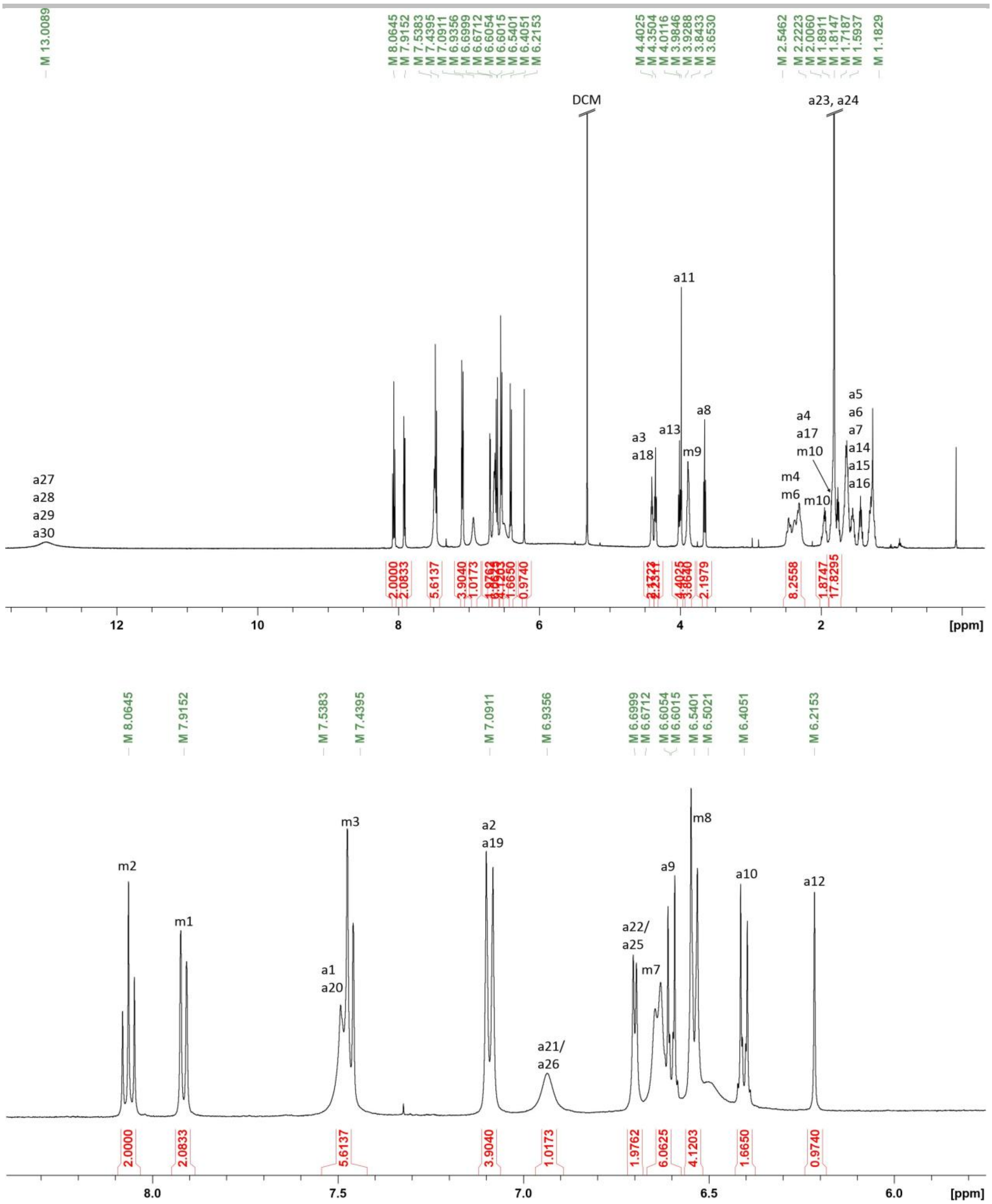

Figure S 103. The ^{1}H NMR spectrum of **[12-H$_3$]$^{3+}$** (600 MHz, [D$_2$]dichloromethane, 300 K).

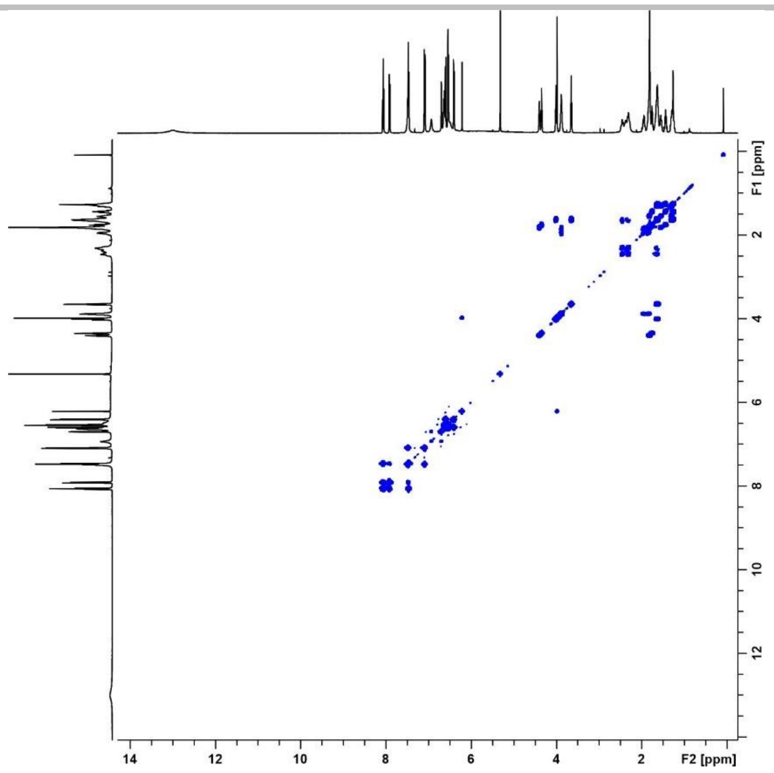

Figure S 104. The ^{1}H-^{1}H COSY NMR spectrum of **[12-H$_3$]$^{3+}$** (600 MHz, [D$_2$]dichloromethane, 300 K).

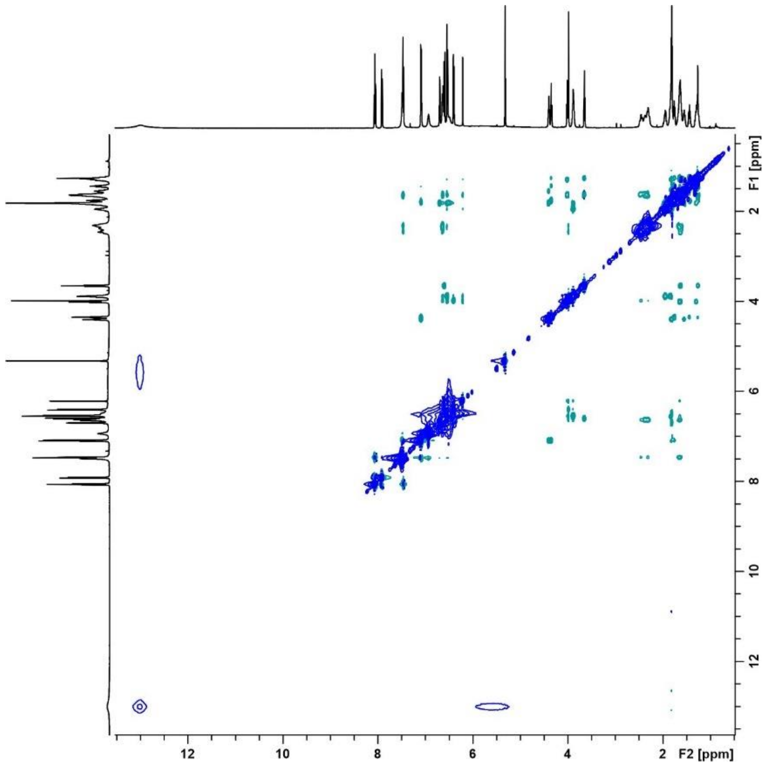

Figure S 105. The ^{1}H-^{1}H NOESY NMR spectrum of **[12-H$_3$]$^{3+}$** (600 MHz, [D$_2$]dichloromethane, 300 K).

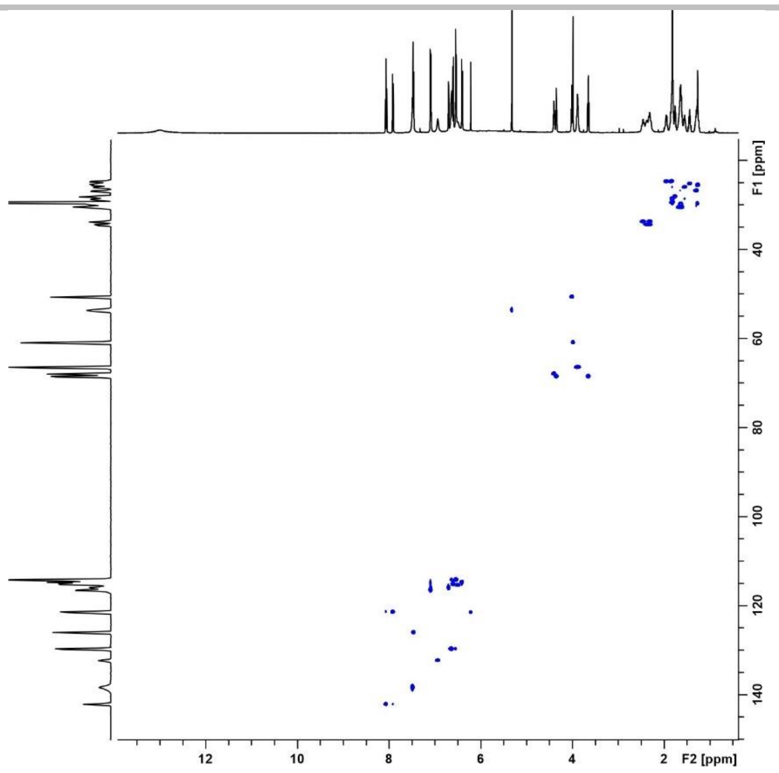

Figure S 106. The ^{1}H-^{13}C HSQC NMR spectrum of **[12-H$_3$]$^{3+}$** (600 MHz, [D$_2$]dichloromethane, 300 K).

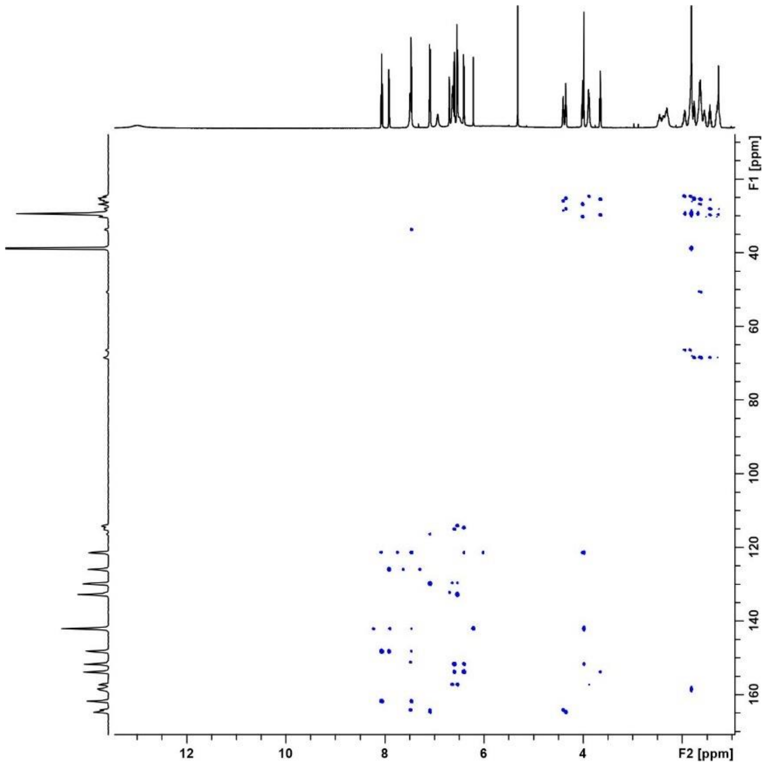

Figure S 107. The ^{1}H-^{13}C HMBC NMR spectrum of **[12-H$_3$]$^{3+}$** (600 MHz, [D$_2$]dichloromethane, 300 K).

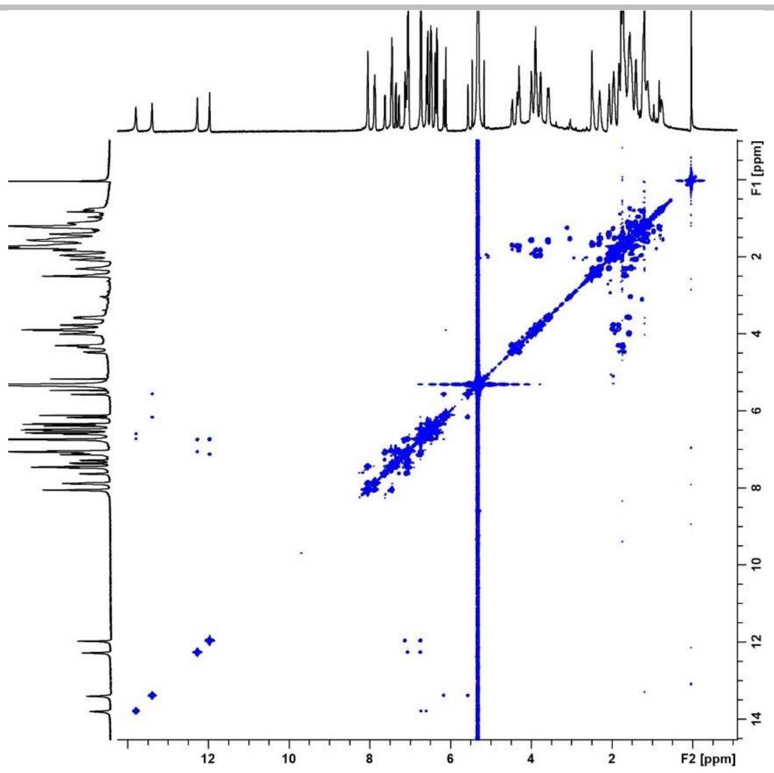

Figure S 108. The ^{1}H-^{1}H COSY NMR spectrum of **[12-H$_3$]$^{3+}$** (600 MHz, [D$_2$]dichloromethane, 240 K).

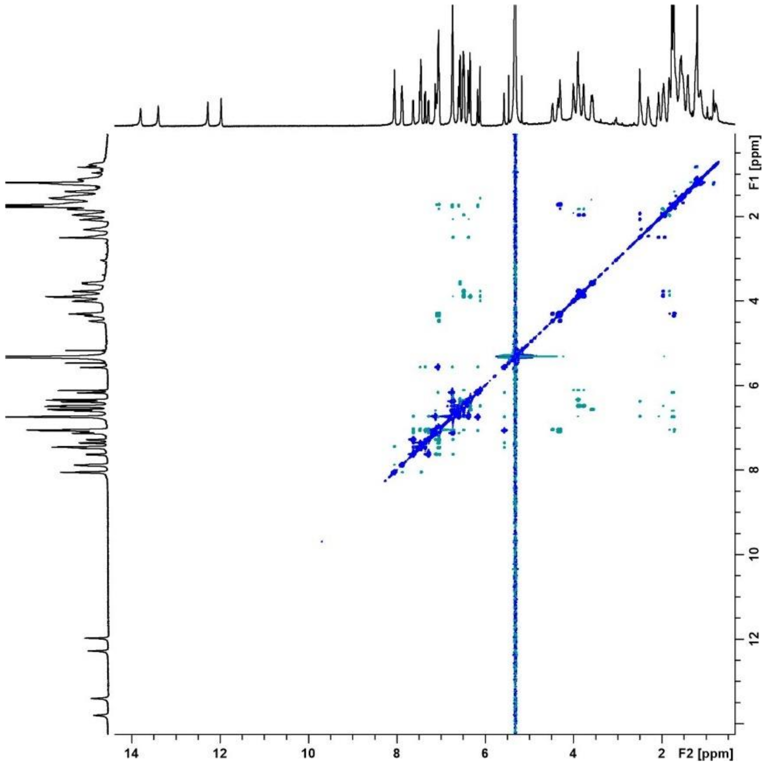

Figure S 109. The ^{1}H-^{1}H ROESY NMR spectrum of **[12-H$_3$]$^{3+}$** (600 MHz, [D$_2$]dichloromethane, 240 K).

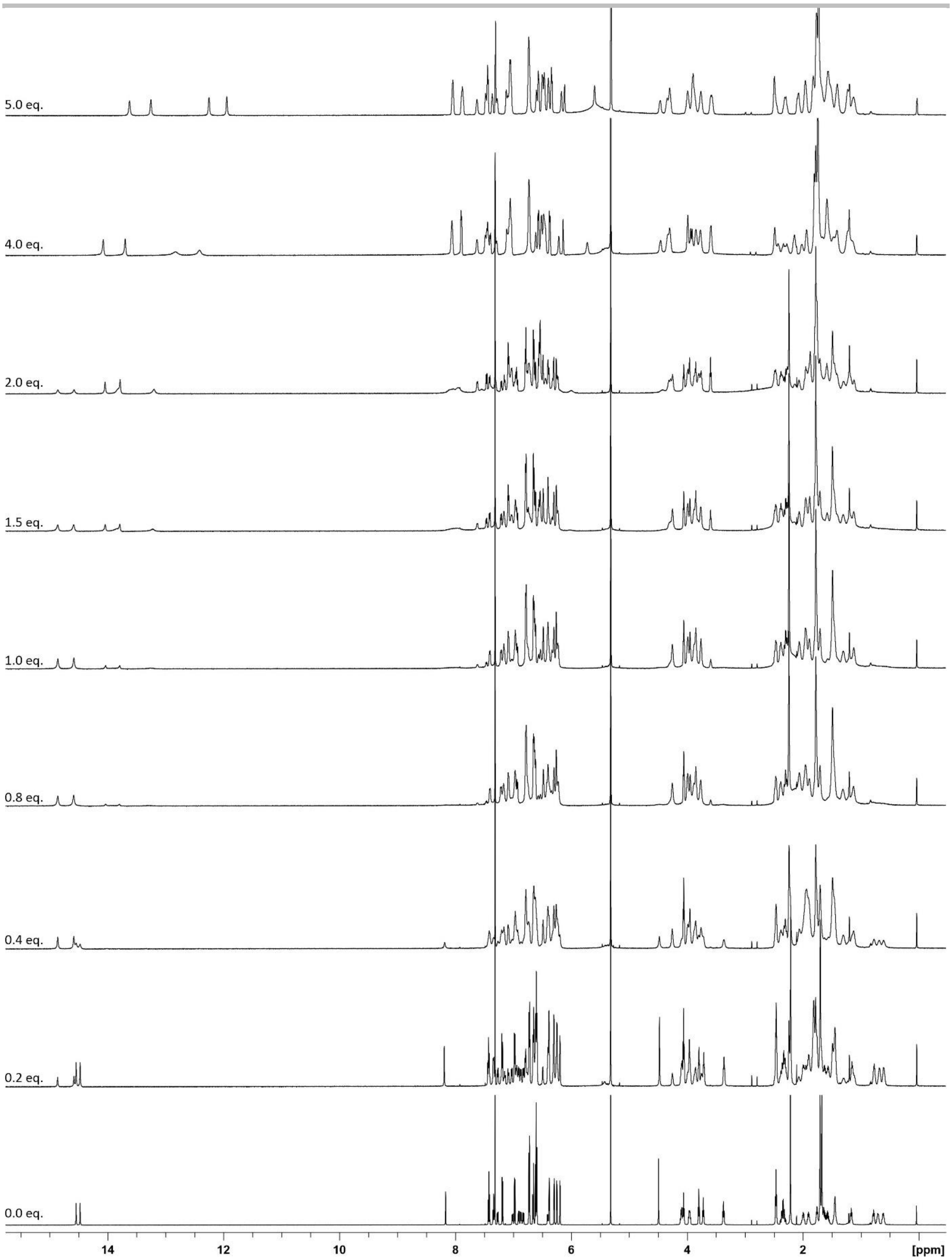

Figure S 110. The ^{1}H NMR spectra recorded during titration of **12** with trifluoroacetic acid (600 MHz, [D$_2$]dichloromethane, 240 K).

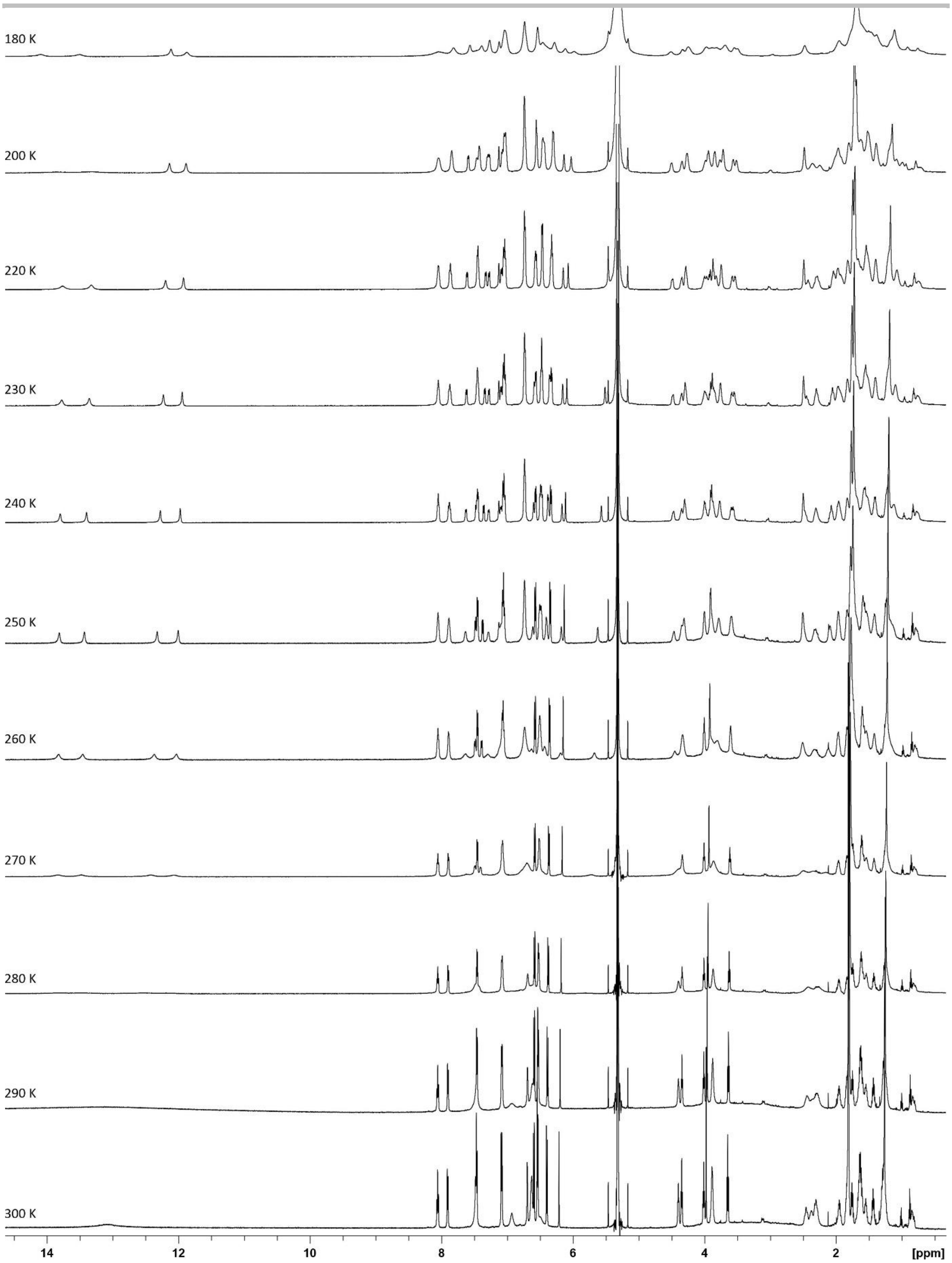

Figure S 111. The ^{1}H NMR spectra of **[12-H$_3$]$^{3+}$** recorded at the 300–180 K temperature range (600 MHz, [D$_2$]dichloromethane).

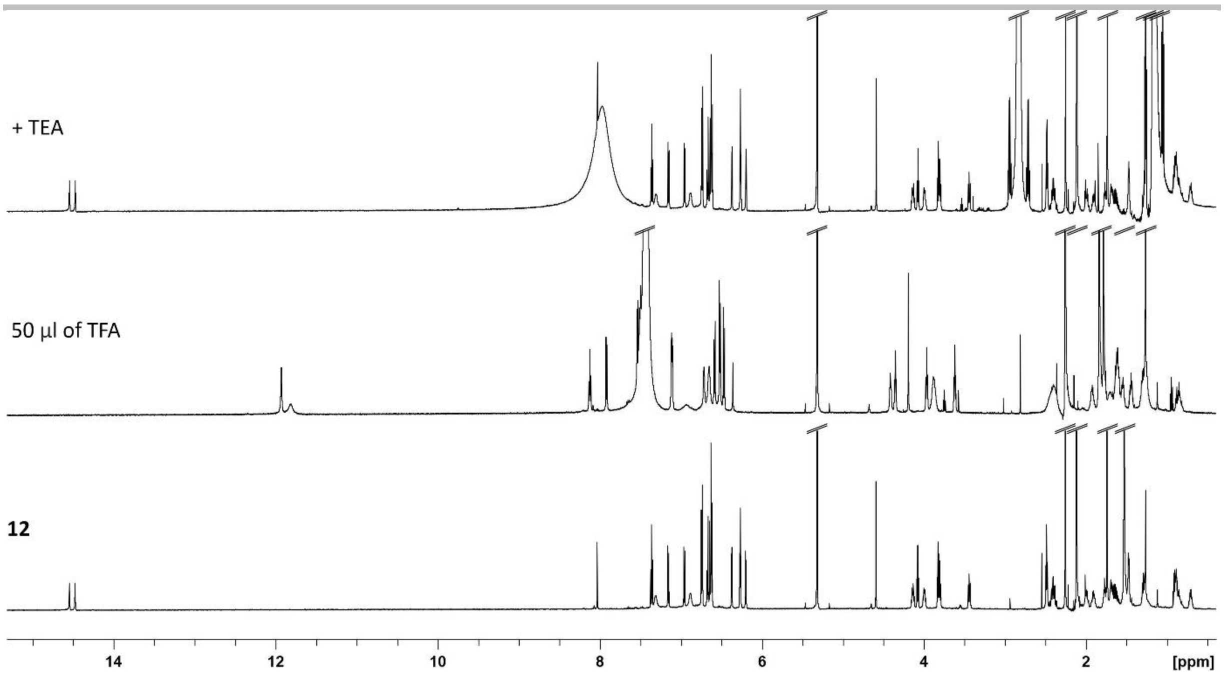

Figure S 112. The ^{1}H NMR spectra recorded after the addition of an excess ca. 50 µl of trifluoroacetic acid to **12**, followed by the addition of TEA (600 MHz, [D$_2$]dichloromethane, 300 K).

Synthesis of methylated compounds

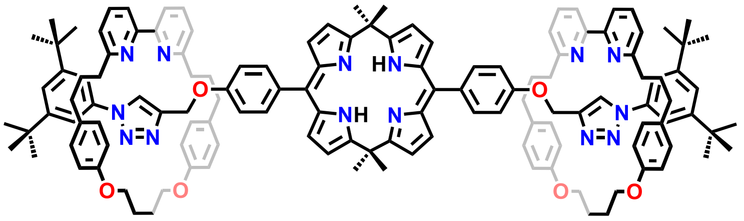

10

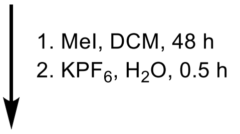

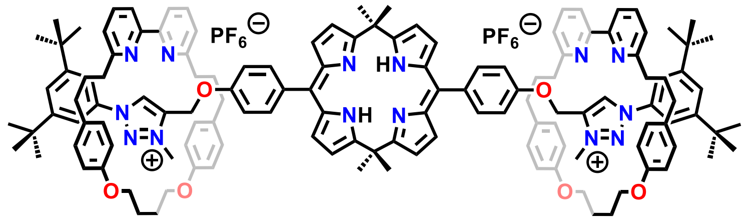

[10-Me$_2$]$^{2+}$; 48%

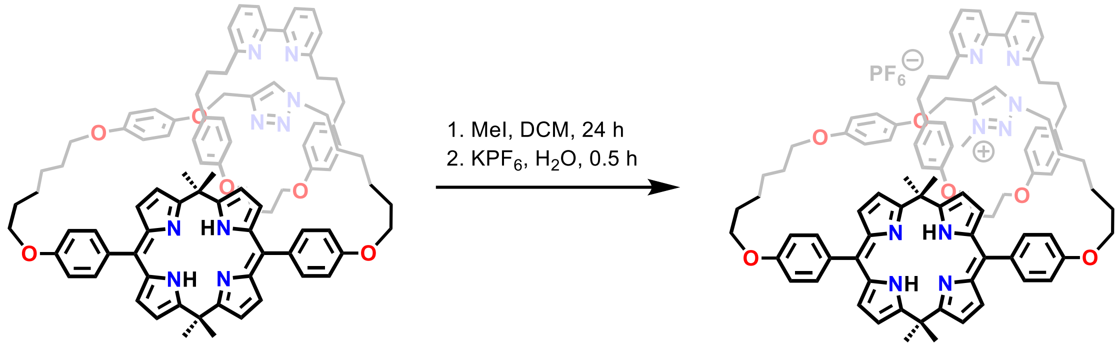

12 **[12-Me]$^{+}$**; 83%

Scheme S 5. Synthesis of **[10-Me$_2$]$^{2+}$** and **[12-Me]$^{+}$**.

Compound [10-Me$_2$]$^{2+}$

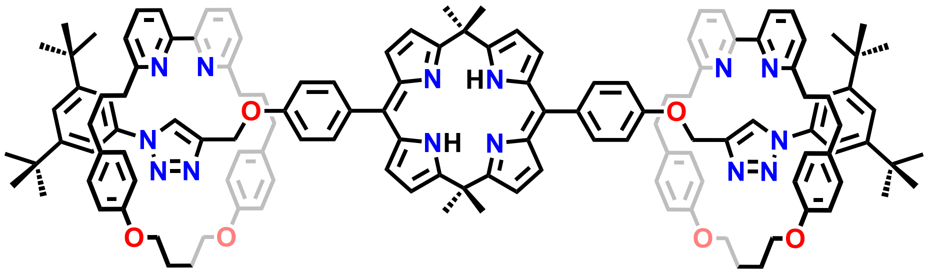

10

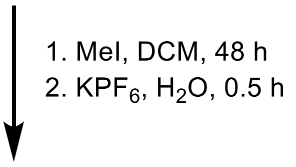

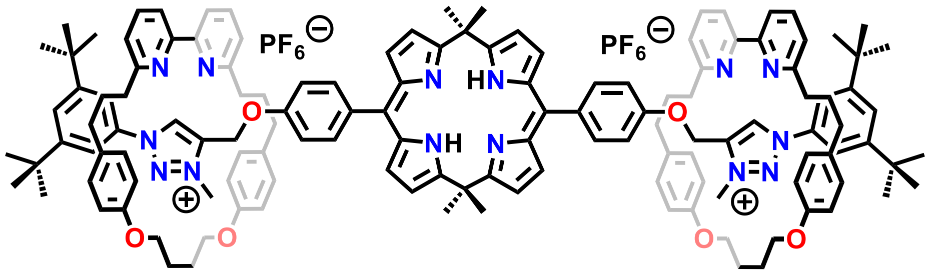

[10-Me$_2$]$^{2+}$; 48%

In a 10 ml vial with a screw cap, rotaxane **10** (12.5 mg, 6.1 µmol), MeI (1 ml) and DCM (1 ml) were introduced. The mixture was stirred for 48 hours at room temperature. After this time, KPF_6 (46 mg, 250 µmol) and H_2O (1 ml) were added, and the heterogenous mixture was stirred vigorously for another 30 minutes. After this time, the mixture was portioned between DCM (20 ml) and H_2O (20 ml) and then transferred into a separatory funnel. The aqueous phase was extracted with DCM (20 ml). The collected organic extracts were washed with water and brine. The collected organic layers were combined and dried over anhydrous Na_2SO_4. The filtrate was collected *via* gravity filtration, and the solvent was removed under reduced pressure. The obtained orange solid was purified *via* flash chromatography (DCM with 0-10% ethyl acetate gradient) and recrystallised from DCM/hexane mixture to provide **[10-Me$_2$]$^{2+}$** (6.76 mg, 2.9 µmol, 48%) as an orange solid.

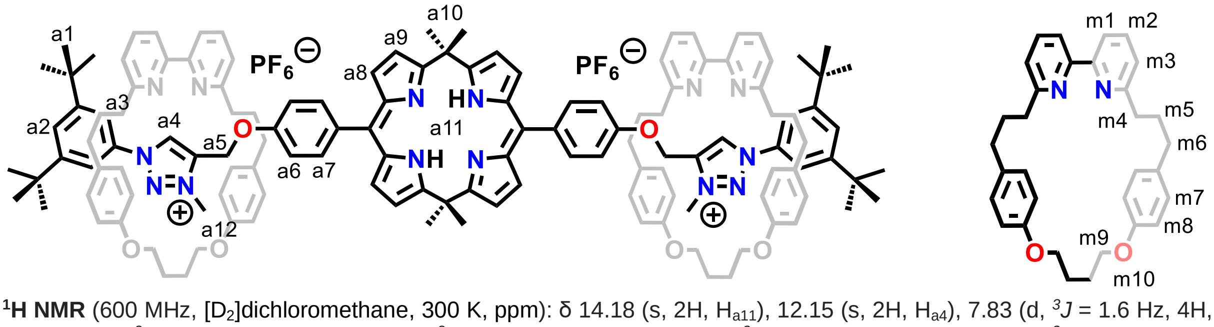

^{1}H NMR (600 MHz, [D$_2$]dichloromethane, 300 K, ppm): δ 14.18 (s, 2H, H_{a11}), 12.15 (s, 2H, H_{a4}), 7.83 (d, ^3J = 1.6 Hz, 4H, H_{a3}), 7.73 (t, ^3J = 7.8 Hz, 4H, H_{m2}), 7.68 (t, ^3J = 1.6 Hz, 2H, H_{a2}), 7.58 (d, ^3J = 7.8 Hz, 4H, H_{m1}), 7.26 (d, ^3J = 8.6 Hz, 4H, H_{a7}), 7.18 (d, ^3J = 7.7, 4H, H_{m3}), 6.70 (d, ^3J = 8.6 Hz, 4H, H_{a6}), 6.65 (d, ^3J = 8.5 Hz, 8H, H_{m8}), 6.59 (d, ^3J = 8.5 Hz, 8H, H_{m7}), 6.27 (d, ^3J = 4.1 Hz, 4H, H_{a9}), 6.24 (d, ^3J = 4.1 Hz, 4H, H_{a8}), 4.80 (s, 2H, H_{a5}), 4.35–4.20 (m, 8H, H_{m9}), 4.28 (s, 6H, H_{a12}), 2.57–2.49 (m, 4H, H_{m6}), 2.35–2.24 (m, 8H, H_{m4}, H_{m6}), 2.24–2.11 (m, 12H, H_{m4}, H_{m10}), 1.93 (s, 12H, H_{a10}), 1.66–1.56 (m, 4H, H_{m5}), 1.56–1.45 (m, 4H, H_{m5}), 1.25 (s, 36H, H_{a1}); **^{13}C NMR** (125 MHz, [D$_2$]dichloromethane, 300 K, ppm): δ 165.5, 162.6, 157.5, 157.2, 156.7, 153.5, 140.4, 139.5, 138.0, 134.4, 133.9, 132.3, 132.1, 131.2, 129.5, 128.0, 126.1, 123.0, 120.6, 116.0, 114.4, 114.1, 113.6, 66.4, 57.4, 38.7, 38.3, 37.0, 35.5, 34.9, 32.3, 31.0, 25.1; **HR–ESI–MS** (*m/z*): [M]$^{2+}$calcd. for $C_{136}H_{152}N_{14}O_6^{2+}$, 1039.1020; found, 1039.1025.

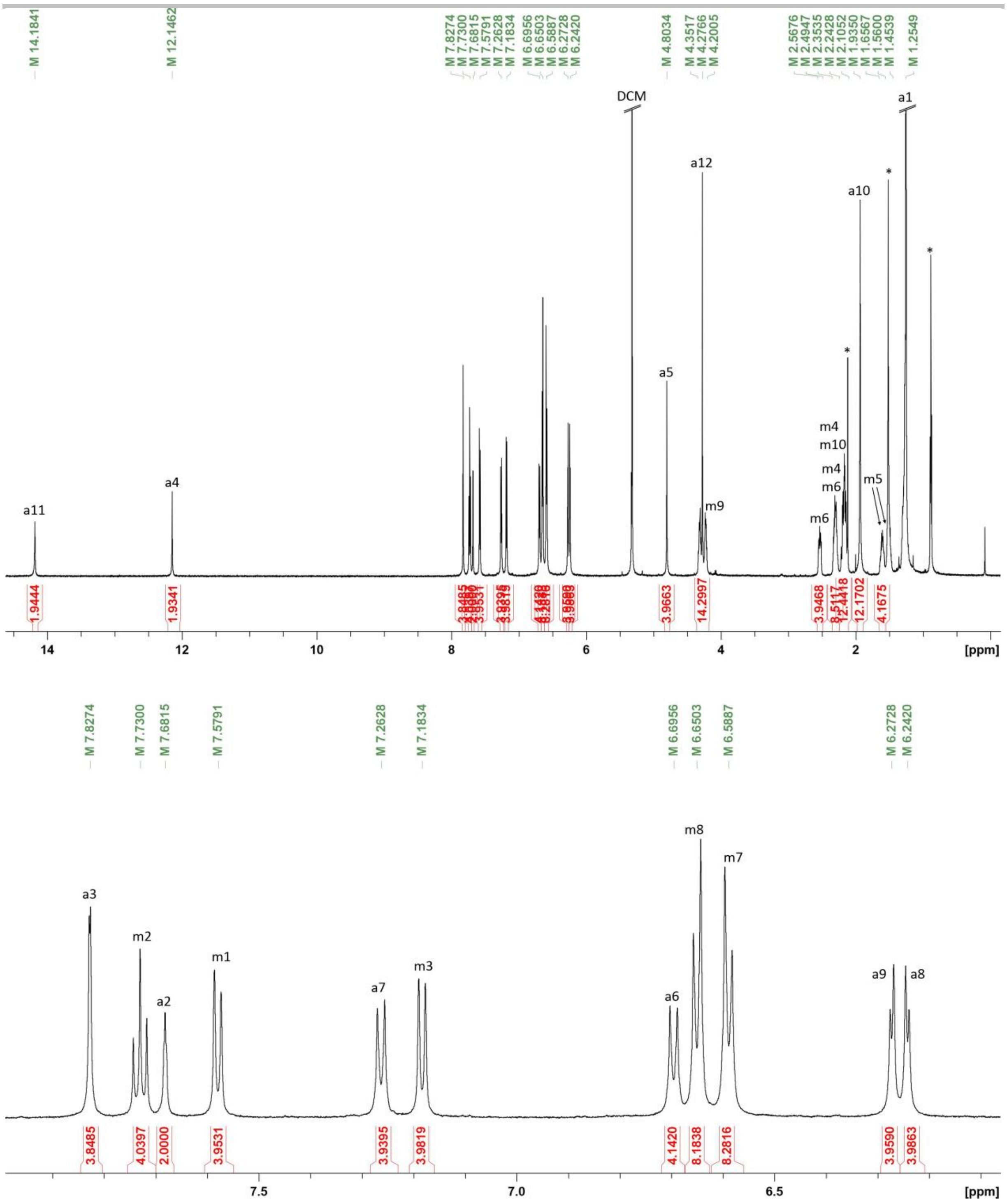

Figure S 113. The ^1H NMR spectrum of **[10-Me$_2$]$^{2+}$** (600 MHz, [D$_2$]dichloromethane, 300 K).

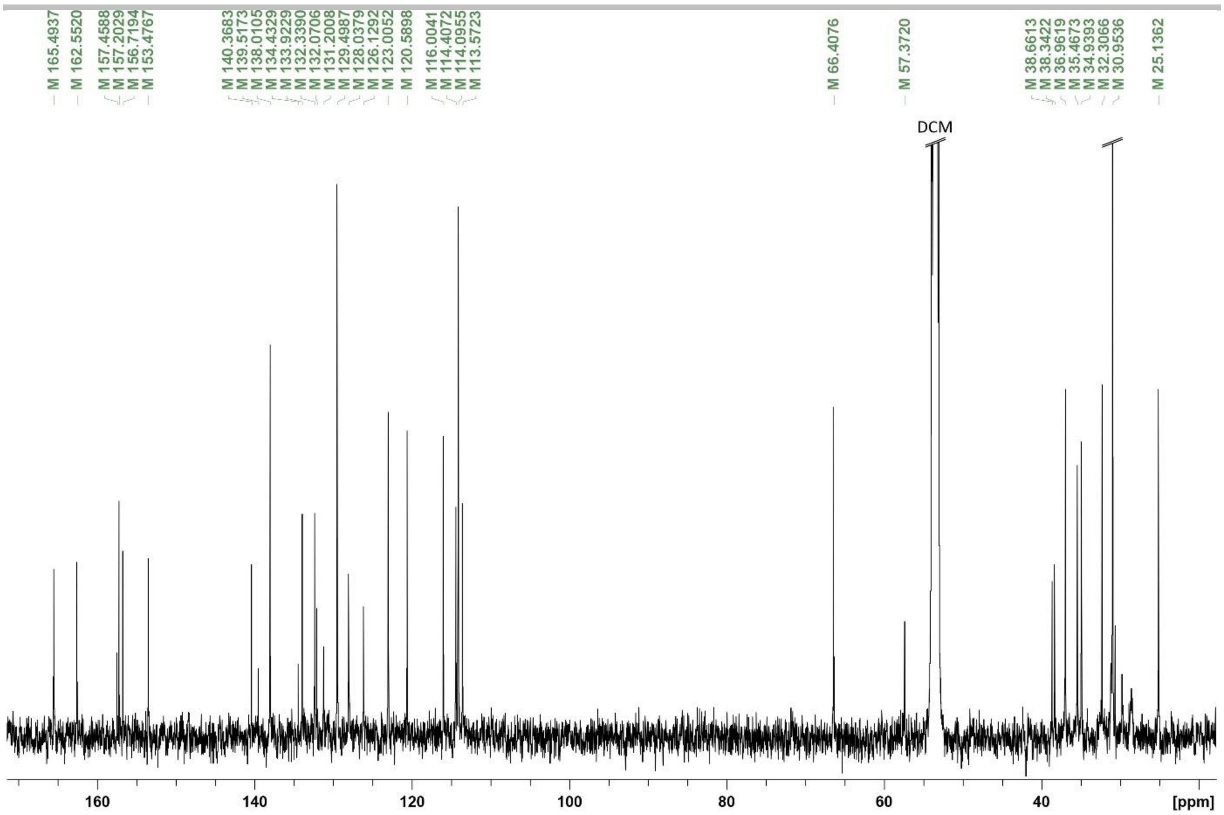

Figure S 114. The ^{13}C NMR spectrum of **[10-Me$_2$]$^{2+}$** (125 MHz, [D$_2$]dichloromethane, 300 K).

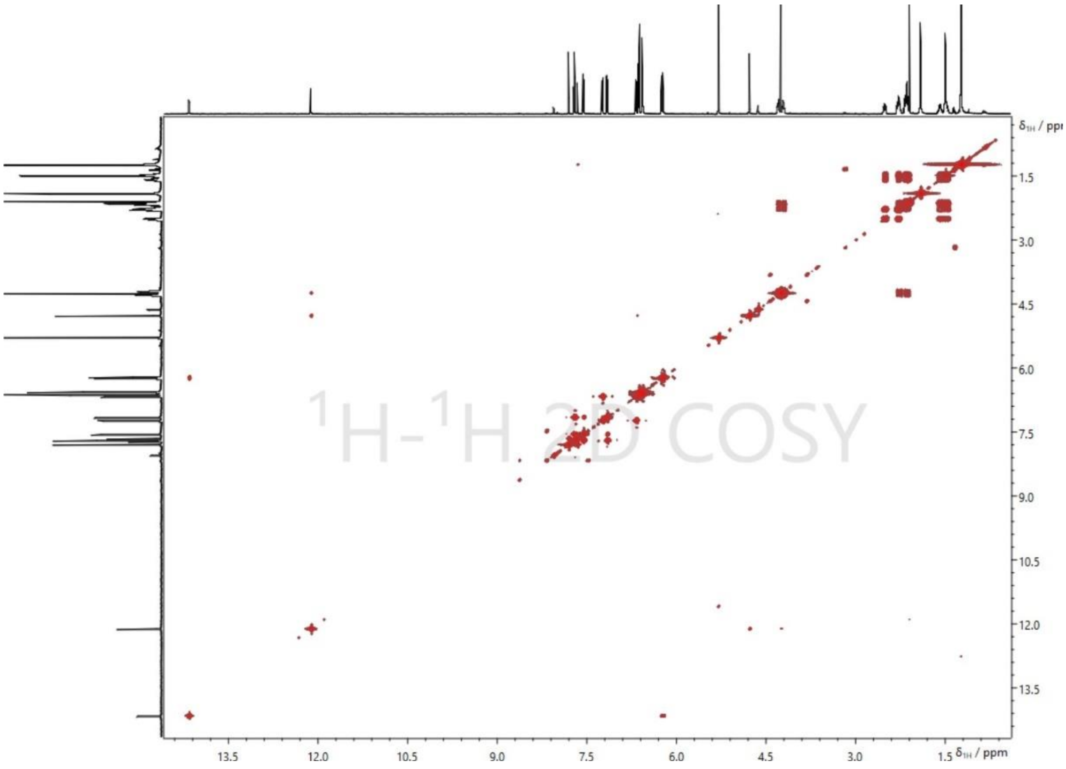

Figure S 115. The ^{1}H-^{1}H COSY NMR spectrum of **[10-Me$_2$]$^{2+}$** (500 MHz, [D$_2$]dichloromethane, 300 K).

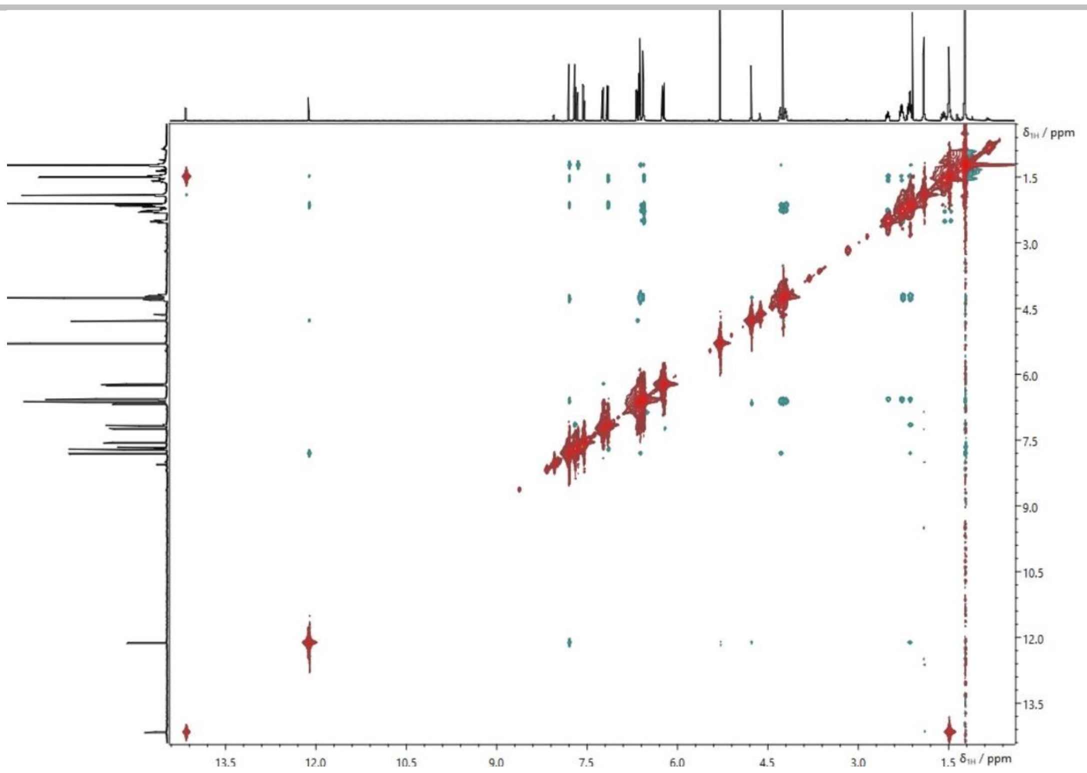

Figure S 116. The ^{1}H-^{1}H NOESY NMR spectrum of **[10-Me2]$^{2+}$** (500 MHz, [D2]dichloromethane, 300 K).

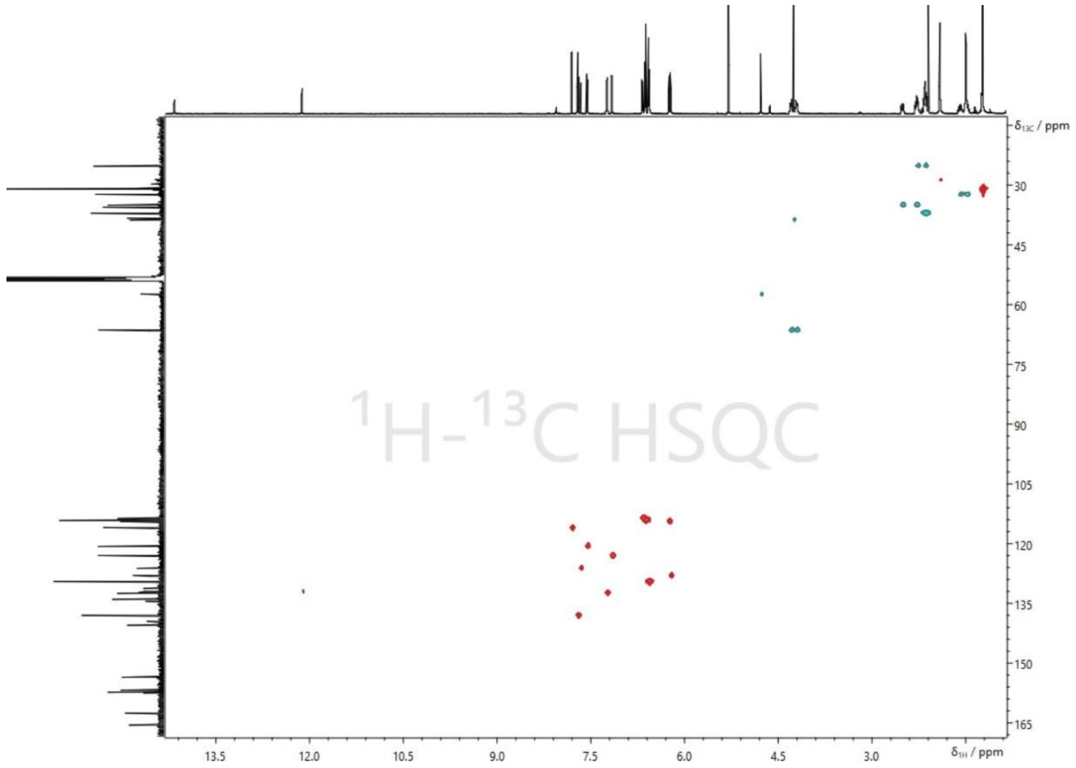

Figure S 117. The ^{1}H-^{13}C HSQC NMR spectrum of **[10-Me2]$^{2+}$** (500 MHz, [D2]dichloromethane, 300 K).

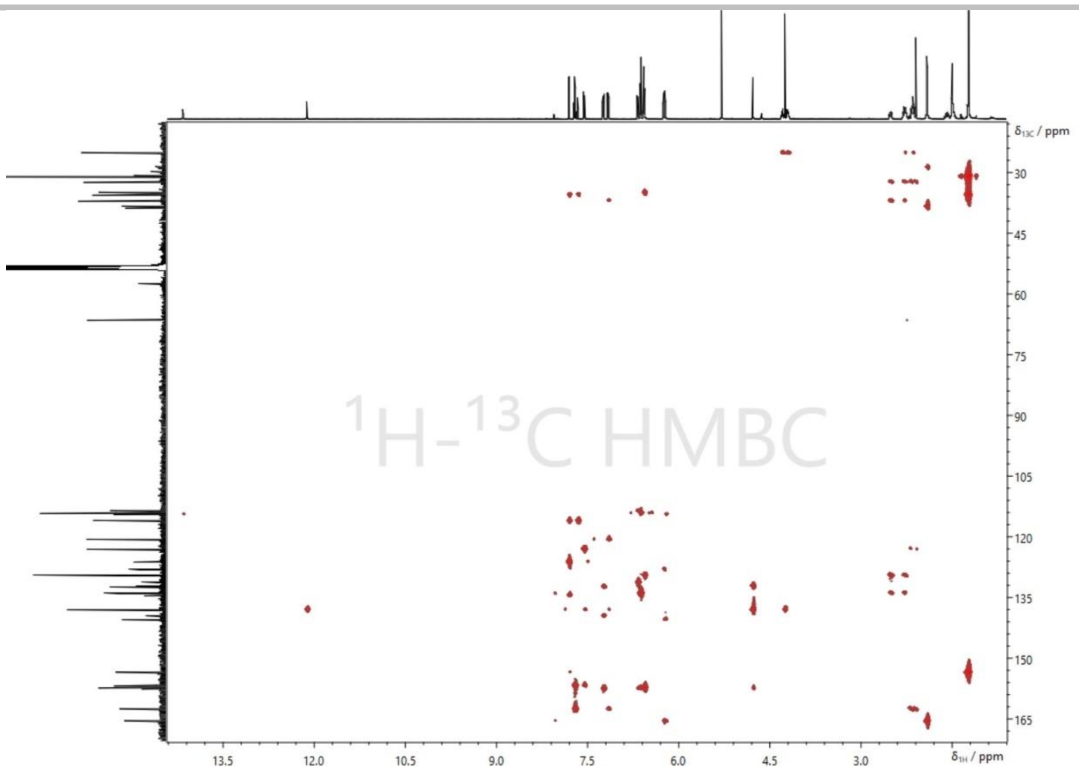

Figure S 118. The ^{1}H-^{13}C HMBC spectrum of **[10-Me$_2$]$^{2+}$** (500 MHz, [D$_2$]dichloromethane, 300 K).

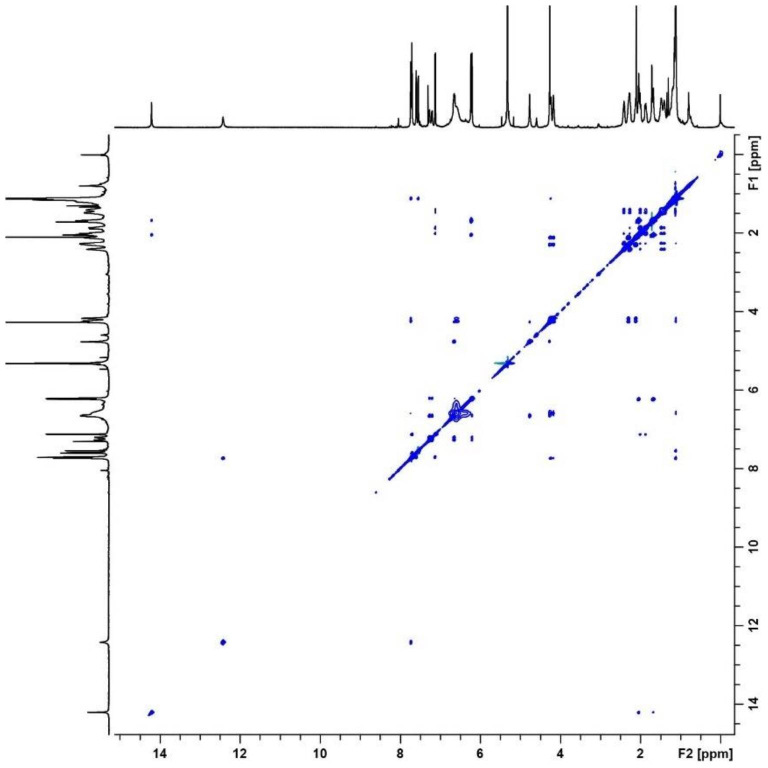

Figure S 119. The ^{1}H-^{1}H NOESY NMR spectrum of **[10-Me$_2$]$^{2+}$** (600 MHz, [D$_2$]dichloromethane, 200 K).

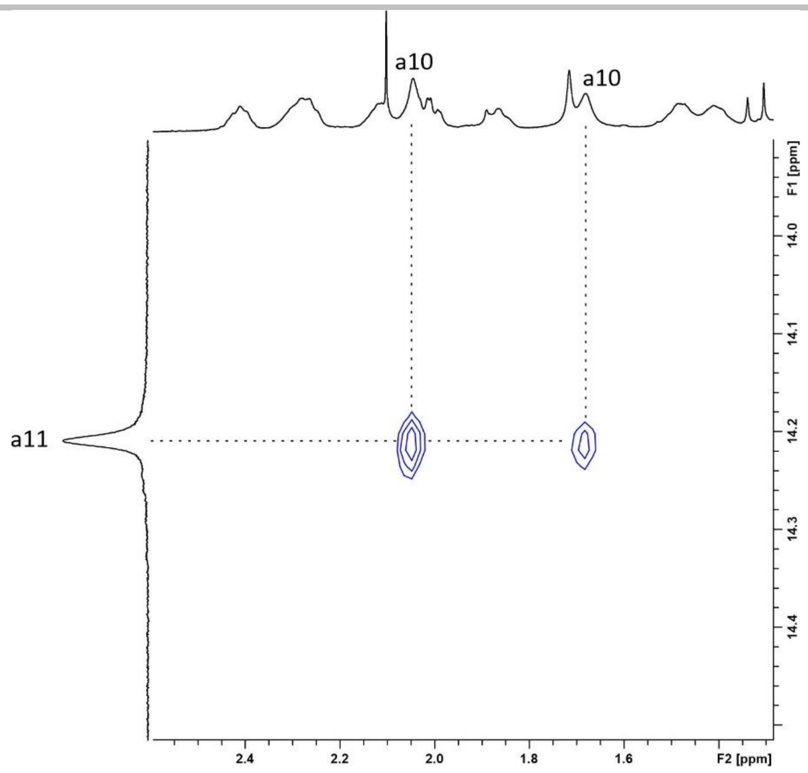

Figure S 120. The ^{1}H-^{1}H NOESY NMR spectrum of **[10-Me$_2$]$^{2+}$** (600 MHz, [D$_2$]dichloromethane, 200 K). The NOE signals showing the correlation between a11 (NH) and a10 (meso-Me) were marked with a dashed line.

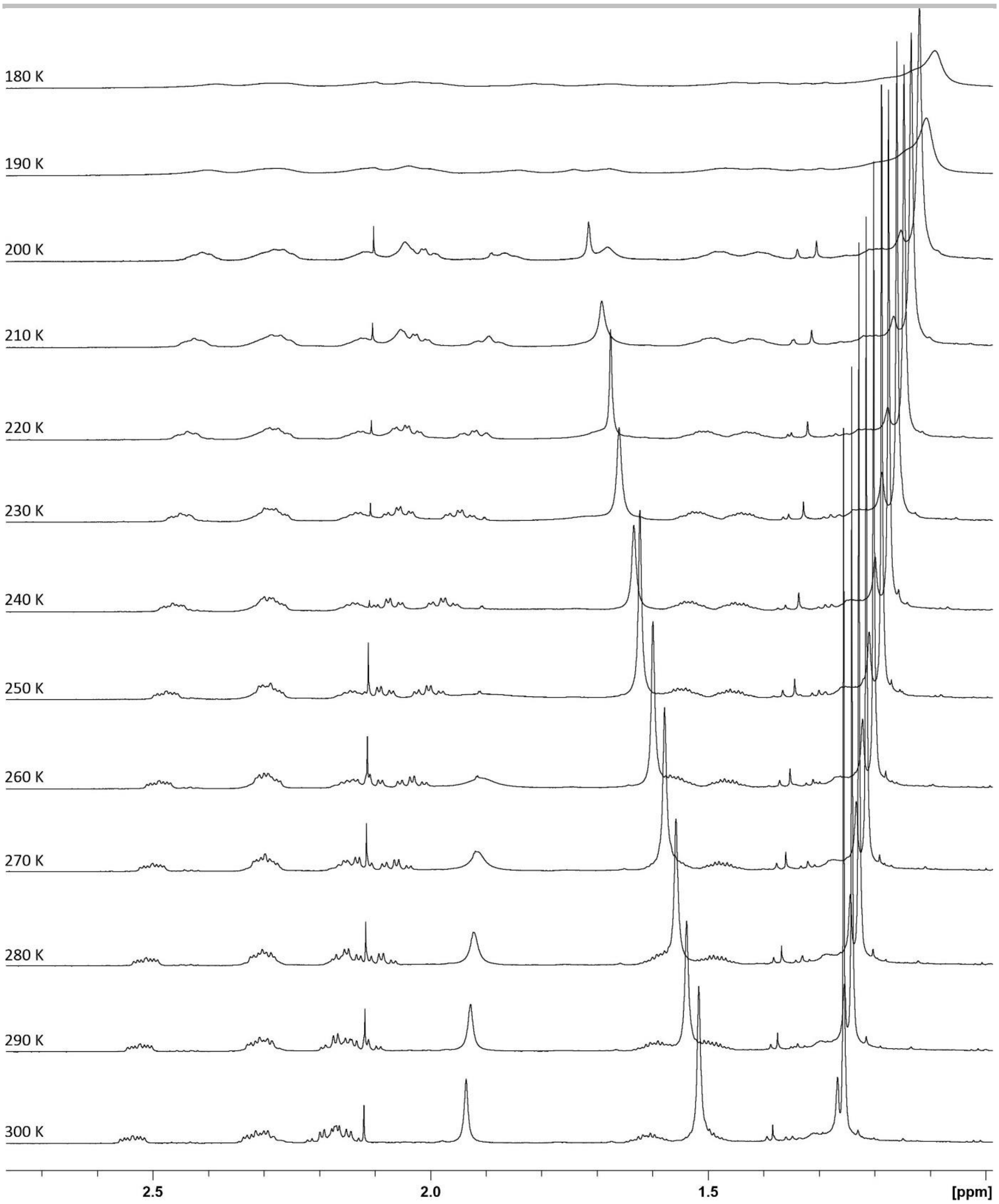

Figure S 121. The aliphatic region of the ^{1}H NMR spectra of **$[10\text{-}Me_2]^{2+}$** recorded at the 300–180 K temperature range (600 MHz, $[D_2]$dichloromethane).

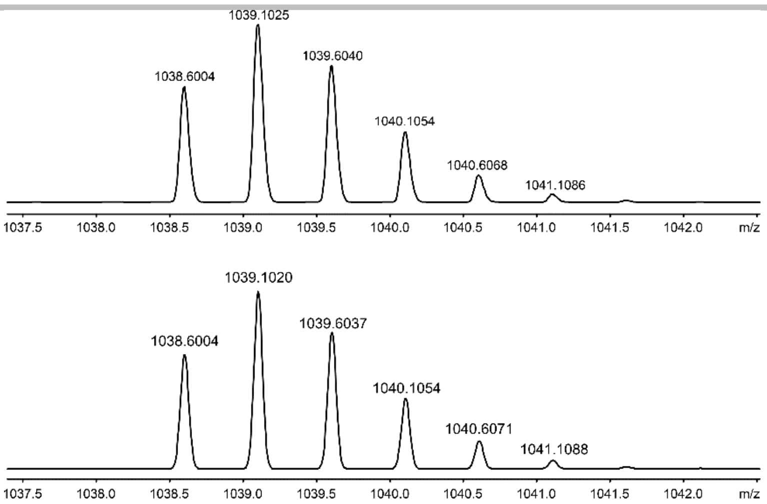

Figure S 122. The high-resolution ESI mass spectrum of **[10-Me$_2$]$^{2+}$**. Top: experimental spectrum, bottom: simulated isotopic pattern.

Compound [10-Me₂H₂]⁴⁺

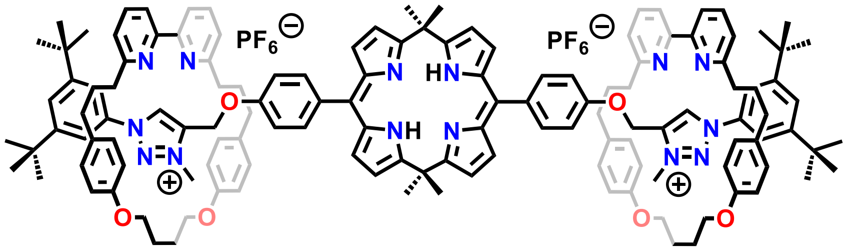

$[10\text{-}Me_2]^{2+}$

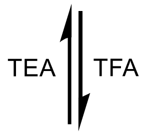

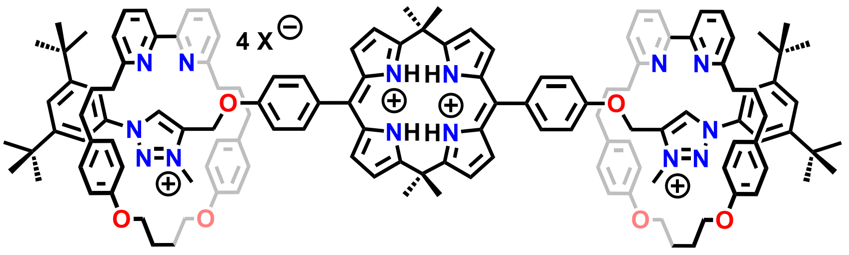

$[10\text{-}Me_2H_2]^{4+}$

The cationic species **$[10\text{-}Me_2H_2]^{4+}$** was obtained by acidification of **$[10\text{-}Me_2]^{2+}$** with ca. two equiv. of trifluoroacetic acid in [D₂]dichloromethane.

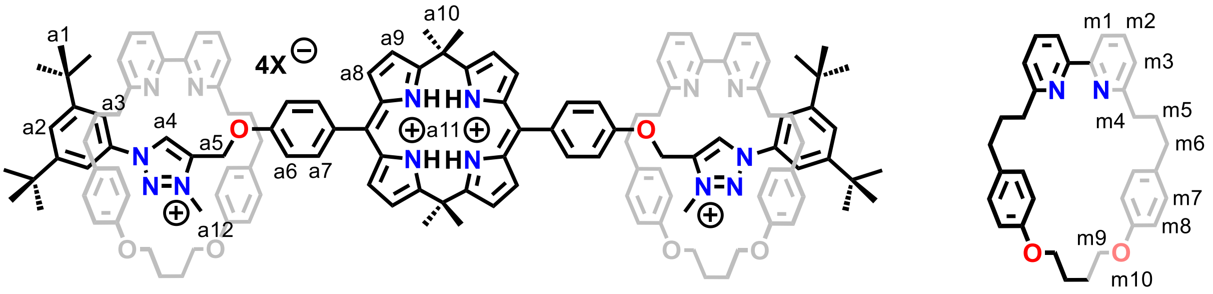

^{1}H NMR (500 MHz, [D₂]dichloromethane, 300 K, ppm): δ 14.06 (s, 4H, H_{a11}), 12.04 (s, 2H, H_{a4}), 7.85 (d, ^3J = 1.5 Hz, 4H, H_{a3}), 7.73 (t, ^3J = 7.8 Hz, 4H, H_{m2}), 7.69 (t, ^3J = 1.5 Hz, 2H, H_{a2}), 7.58 (d, ^3J = 7.8 Hz, 4H, H_{m1}), 7.39 (d, ^3J = 8.3 Hz, 4H, H_{a7}), 7.19 (d, ^3J = 7.8 Hz, 4H, H_{m3}), 6.84–6.77 (m, 8H, H_{a6}, H_{a8}), 6.71 (d, ^3J = 3.9 Hz, 4H, H_{a9}), 6.67 (d, ^3J = 8.7 Hz, 8H, H_{m8}), 6.62 (d, ^3J = 8.7 Hz, 8H, H_{m7}), 4.93 (s, 4H, H_{a5}), 4.36–4.18 (m, 8H, H_{m9}), 4.28 (s, 6H, H_{a12}), 2.61–2.51 (m, 4H, H_{m6}), 2.37–2.25 (m, 8H, H_{m4}, H_{m6}), 2.25–2.11 (m, 12H, H_{m4}, H_{m10}), 1.85 (s, 12H, H_{a10}), 1.68–1.47 (m, 8H, H_{m5}), 1.26 (s, 36H, H_{a1}).

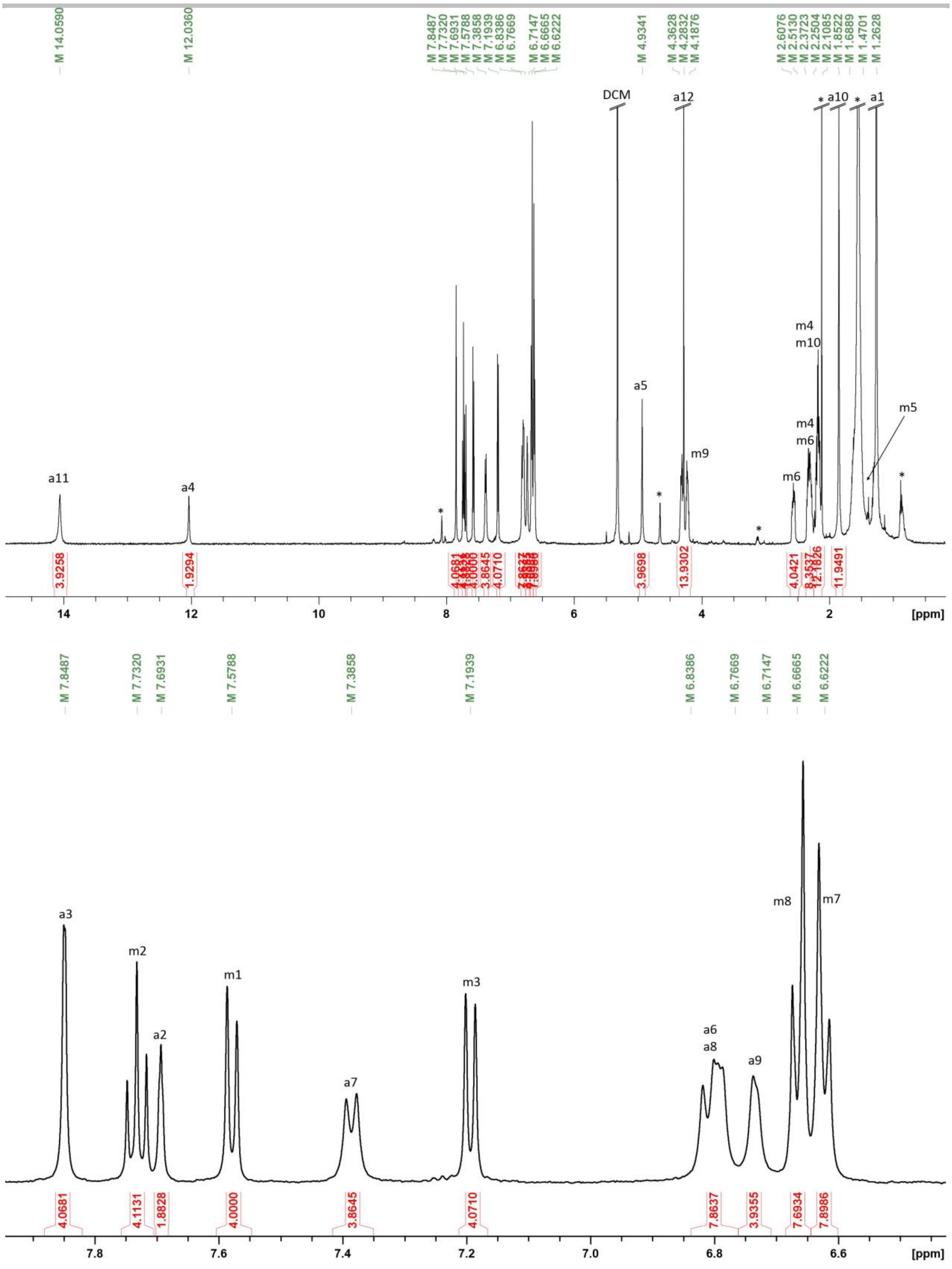

Figure S 123. The ^{1}H NMR spectrum of **[10-Me$_2$H$_2$]$^{4+}$** (500 MHz, [D$_2$]dichloromethane, 300 K).

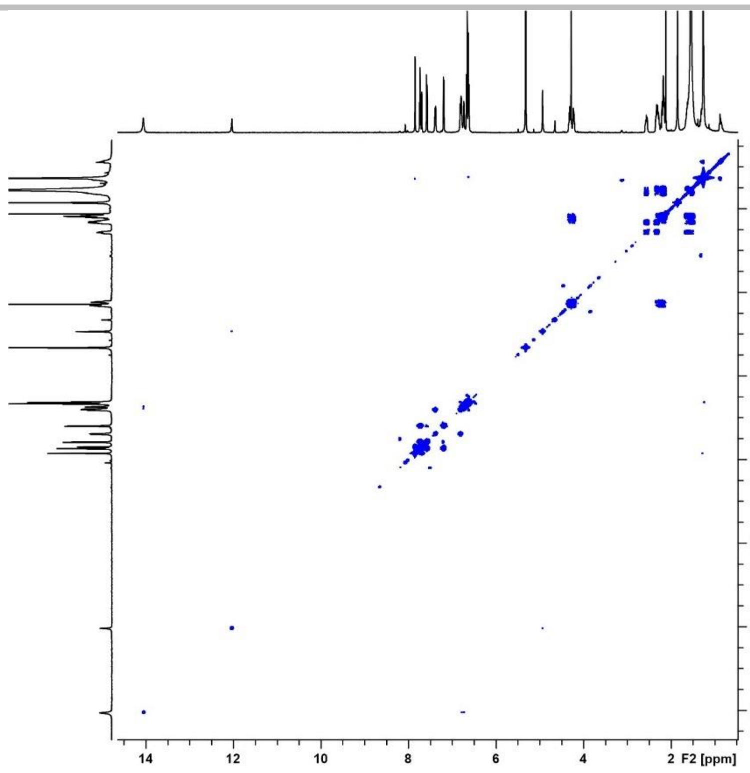

Figure S 124. The ^{1}H-^{1}H COSY NMR spectrum of **[10-Me$_2$H$_2$]$^{4+}$** (500 MHz, [D$_2$]dichloromethane, 300 K).

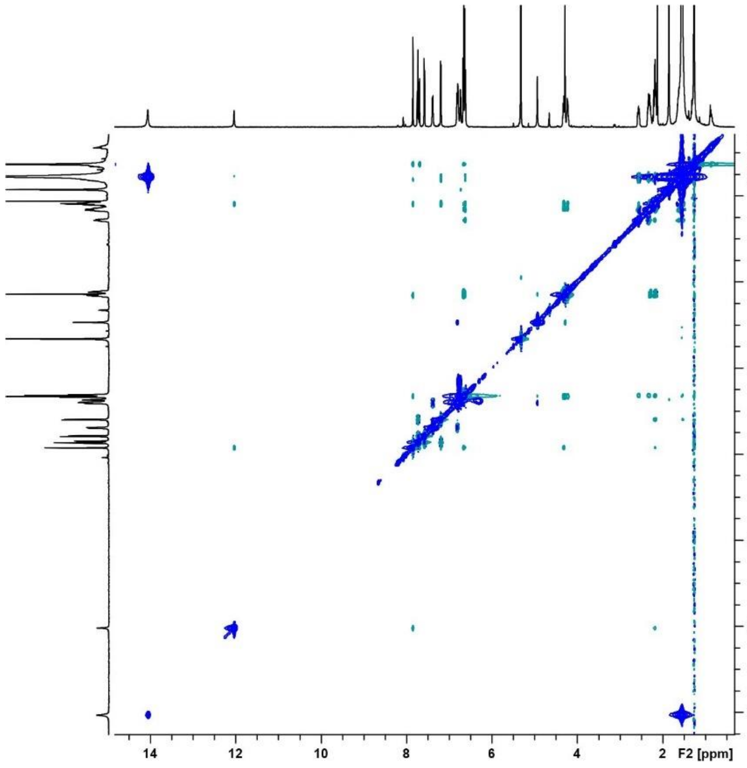

Figure S 125. The ^{1}H-^{1}H NOESY NMR spectrum of **[10-Me$_2$H$_2$]$^{4+}$** (500 MHz, [D$_2$]dichloromethane, 300 K).

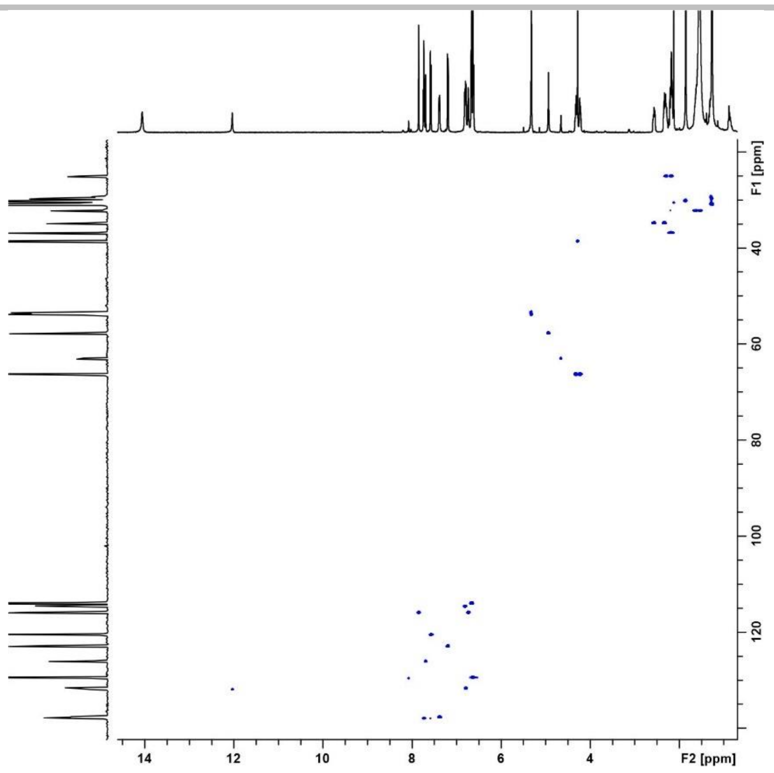

Figure S 126. The ^{1}H-^{13}C HSQC NMR spectrum of **$[10\text{-}Me_2H_2]^{4+}$** (500 MHz, [D_2]dichloromethane, 300 K).

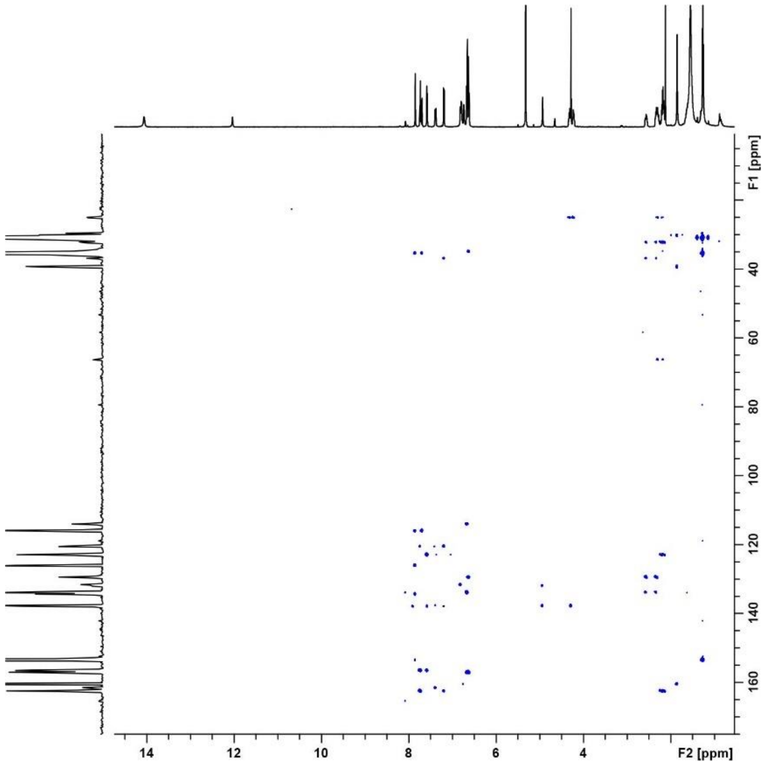

Figure S 127. The ^{1}H-^{13}C HMBC spectrum of **$[10\text{-}Me_2H_2]^{4+}$** (500 MHz, [D_2]dichloromethane, 300 K).

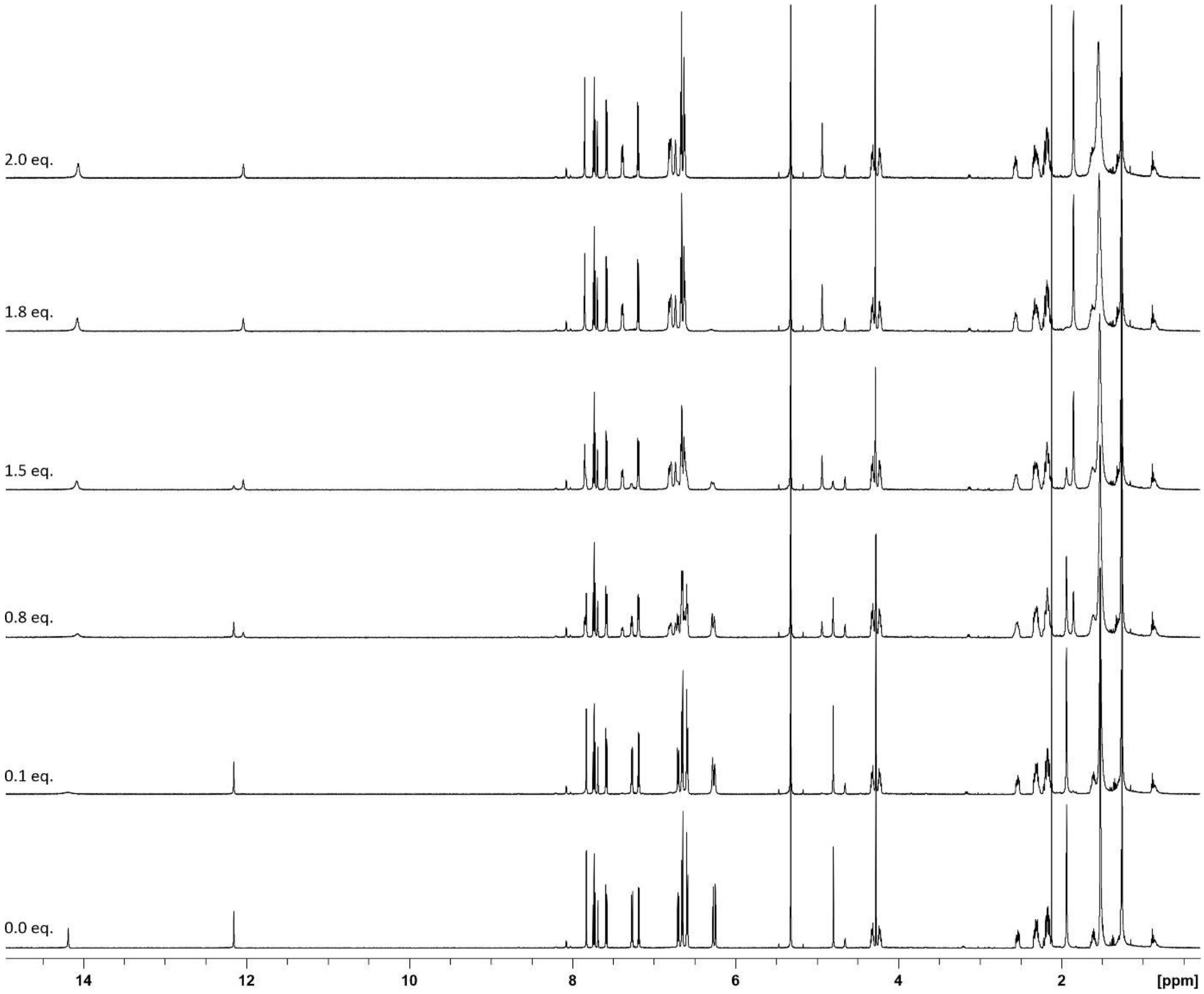

Figure S 128. The ^{1}H NMR spectra recorded during titration of **[10-Me$_2$]$^{2+}$** with trifluoroacetic acid (600 MHz, [D$_2$]dichloromethane, 240 K).

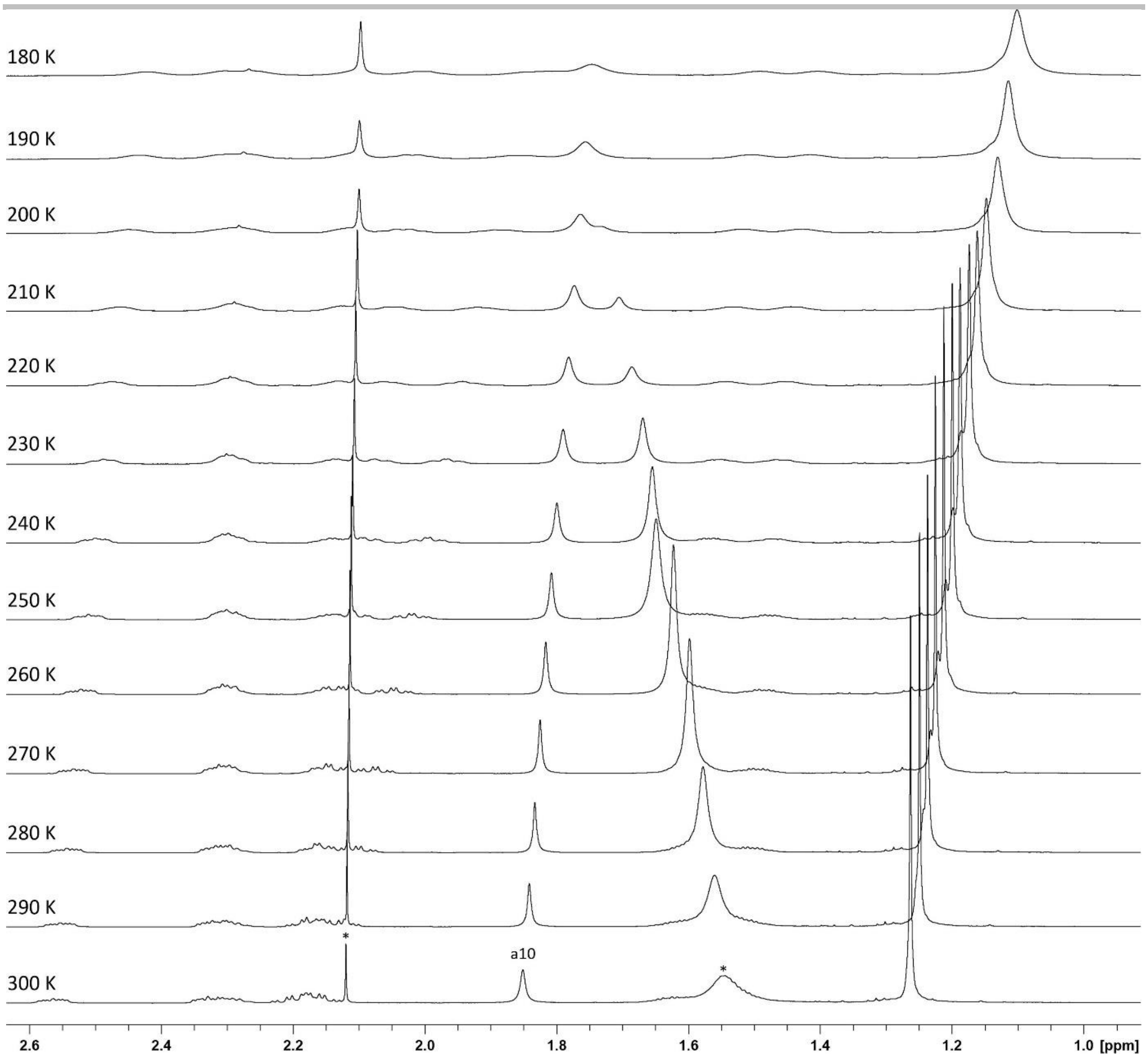

Figure S 129. The aliphatic region of the ^{1}H NMR spectra of **[10-Me$_2$H$_2$]$^{4+}$** recorded in the 300–180 K temperature range (600 MHz, [D$_2$]dichloromethane).

Compound [10-Me$_2$H$_4$]$^{6+}$

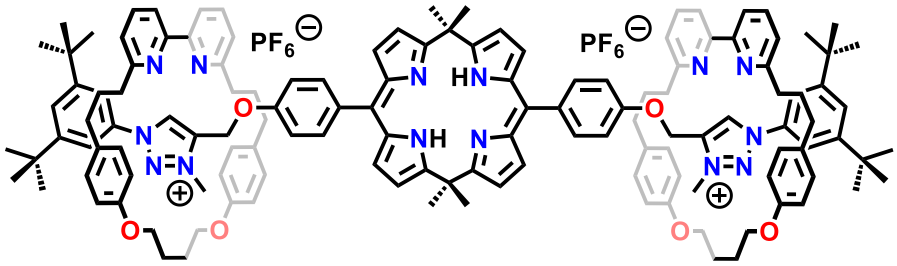

[10-Me$_2$]$^{2+}$

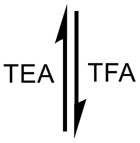

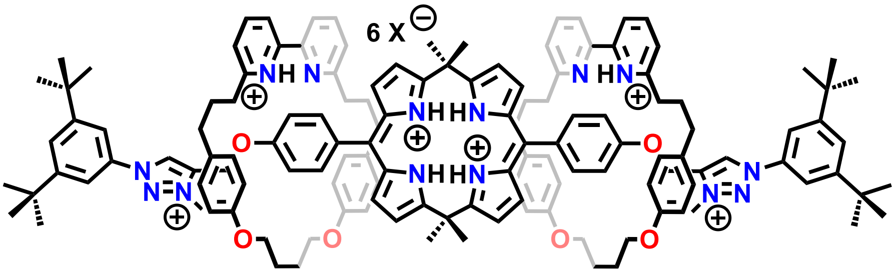

[10-Me$_2$H$_4$]$^{6+}$

The cationic species **[10-Me$_2$H$_4$]$^{6+}$** was obtained by acidification of **[10-Me$_2$]$^{2+}$** with an excess (17 equiv.) of trifluoroacetic acid in [D$_2$]dichloromethane.

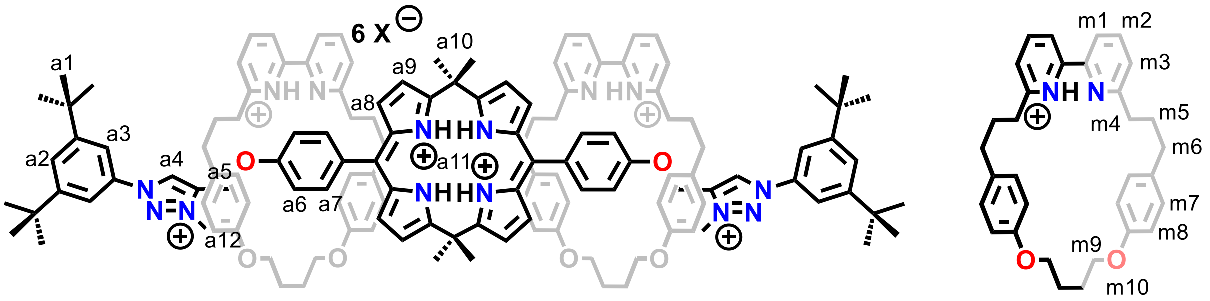

^{1}H NMR (600 MHz, [D$_2$]dichloromethane, 300 K, ppm): δ 12.77 (s, 4H, H_{a11}), 8.57 (s, 2H, H_{a4}), 8.22 (t, ^3J = 8.0 Hz, 4H, H_{m2}), 7.97 (d, ^3J = 7.9 Hz, 4H, H_{m1}), 7.79 (t, ^3J = 1.6 Hz, 2H, H_{a2}), 7.66 (d, ^3J = 8.0 Hz, 4H, H_{m3}), 7.52 (d, ^3J = 1.6 Hz, 4H, H_{a3}), 7.38 (d, ^3J = 8.8 Hz, 4H, H_{a7}), 6.90 (d, ^3J = 8.3 Hz, 8H, H_{m7}), 6.86 (dd, ^3J = 4.4 Hz, ^4J = 2.0 Hz, H_{a9}), 6.84 (d, ^3J = 8.8 Hz, 4H, H_{a6}), 6.82 (dd, ^3J = 4.4 Hz, ^4J = 2.0 Hz, H_{a8}), 6.52 (d, ^3J = 8.3 Hz, 8H, H_{m8}), 4.52 (s, 4H, H_{a5}), 4.36 (s, 6H, H_{a12}), 4.23–4.16 (m, 4H, H_{m9}), 4.13–4.05 (m, 4H, H_{m9}), 2.81–2.72 (m, 8H, H_{m4}, H_{m6}), 6.67–2.56 (m, 8H, H_{m4}, H_{m6}), 2.18–2.04 (m, 8H, H_{m10}), 2.00–1.91 (m, 4H, H_{m5}), 1.91–1.78 (m, 4H, H_{m5}), 1.87 (s, 12H, H_{a10}), 1.36 (s, 36H, H_{a1}).

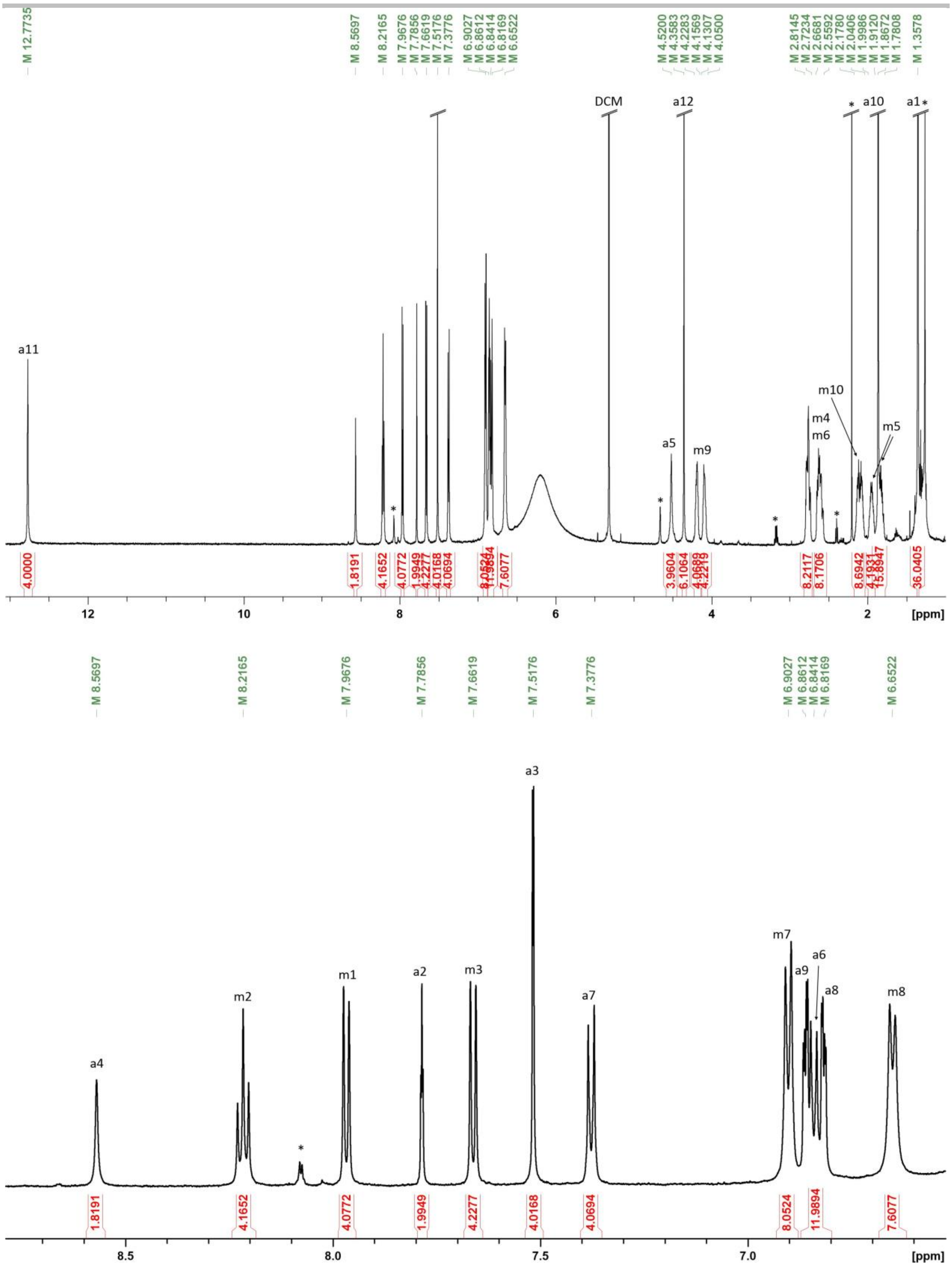

Figure S 130. The ^{1}H NMR spectrum of **[10-Me$_2$H$_4$]$^{6+}$** (600 MHz, [D$_2$]dichloromethane, 300 K).

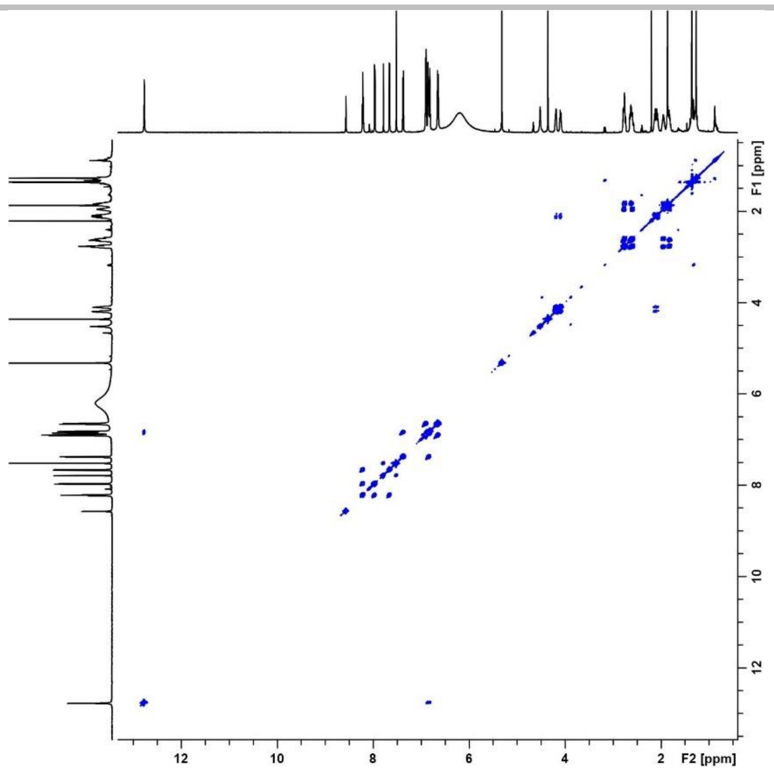

Figure S 131. The ^{1}H-^{1}H COSY NMR spectrum of **[10-Me$_2$H$_4$]$^{6+}$** (600 MHz, [D$_2$]dichloromethane, 300 K).

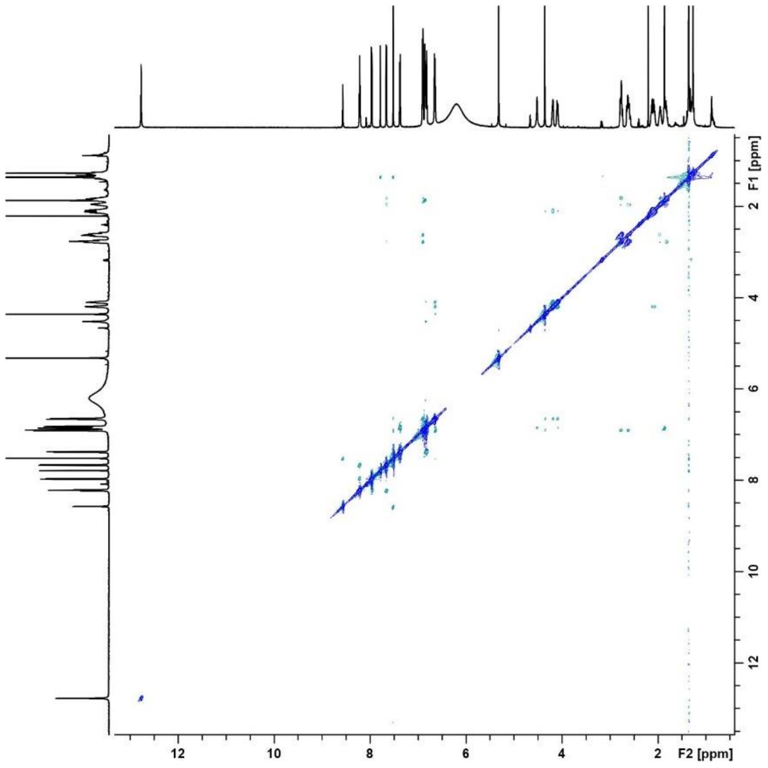

Figure S 132. The ^{1}H-^{1}H ROESY NMR spectrum of **[10-Me$_2$H$_4$]$^{6+}$** (600 MHz, [D$_2$]dichloromethane, 300 K).

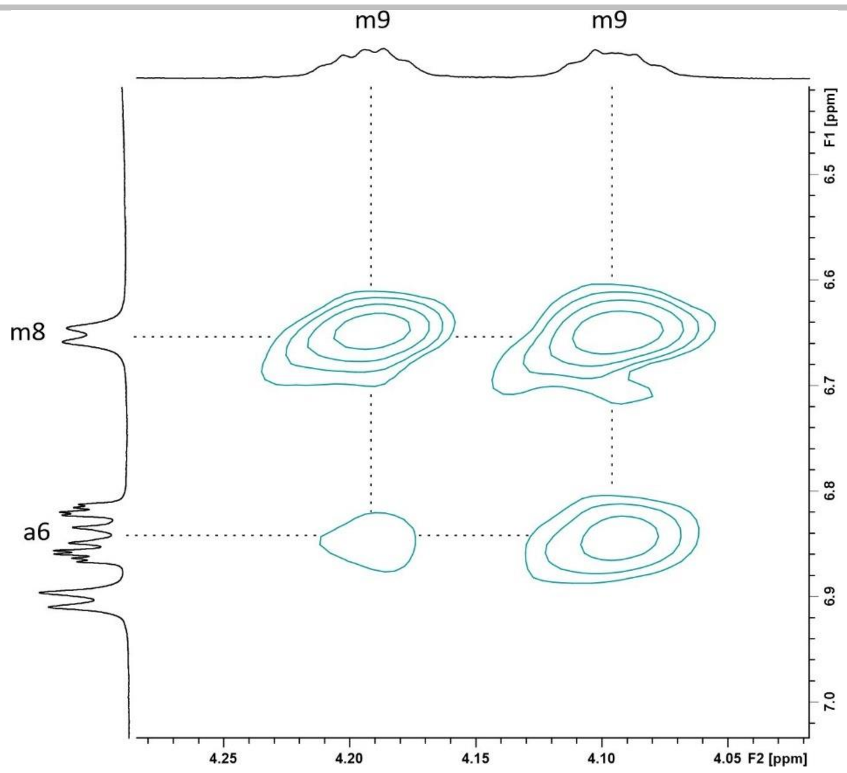

Figure S 133. The ^{1}H-^{1}H ROESY NMR spectrum of **[10-Me$_2$H$_4$]$^{6+}$** (600 MHz, [D$_2$]dichloromethane, 300 K). The NOE signal showing the correlations between protons m9 and m8 and m9 and a6 were marked with dashed lines.

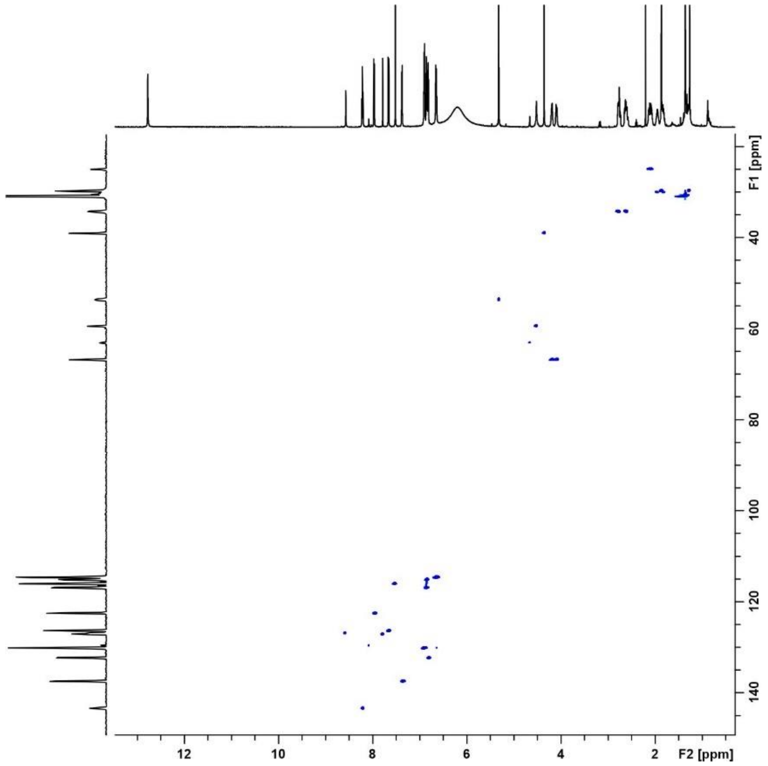

Figure S 134. The ^{1}H-^{13}C HSQC NMR spectrum of **[10-Me$_2$H$_4$]$^{6+}$** (600 MHz, [D$_2$]dichloromethane, 300 K).

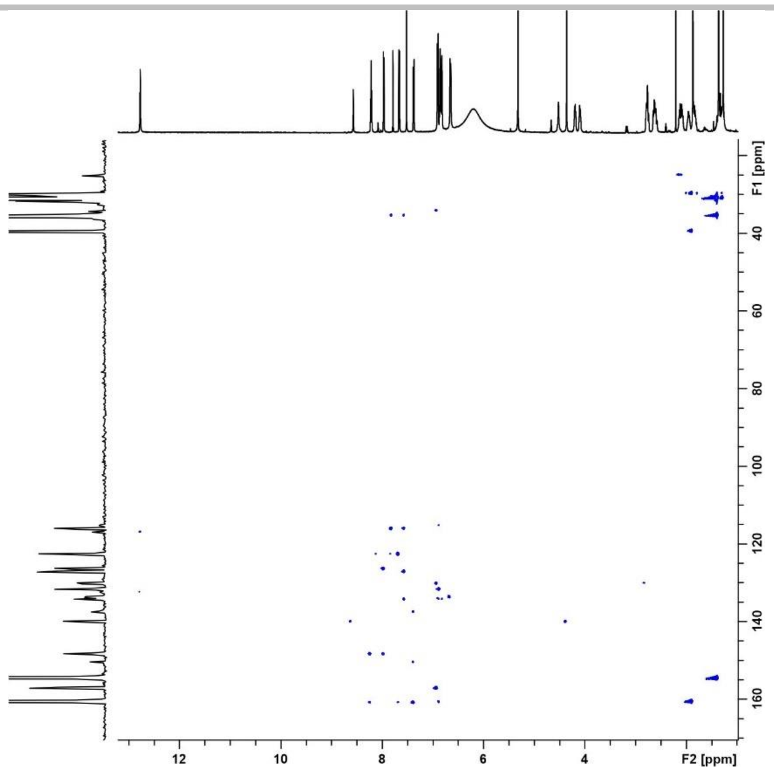

Figure S 135. The ^{1}H-^{13}C HMBC spectrum of **[10-Me$_2$H$_4$]$^{6+}$** (600 MHz, [D$_2$]dichloromethane, 300 K).

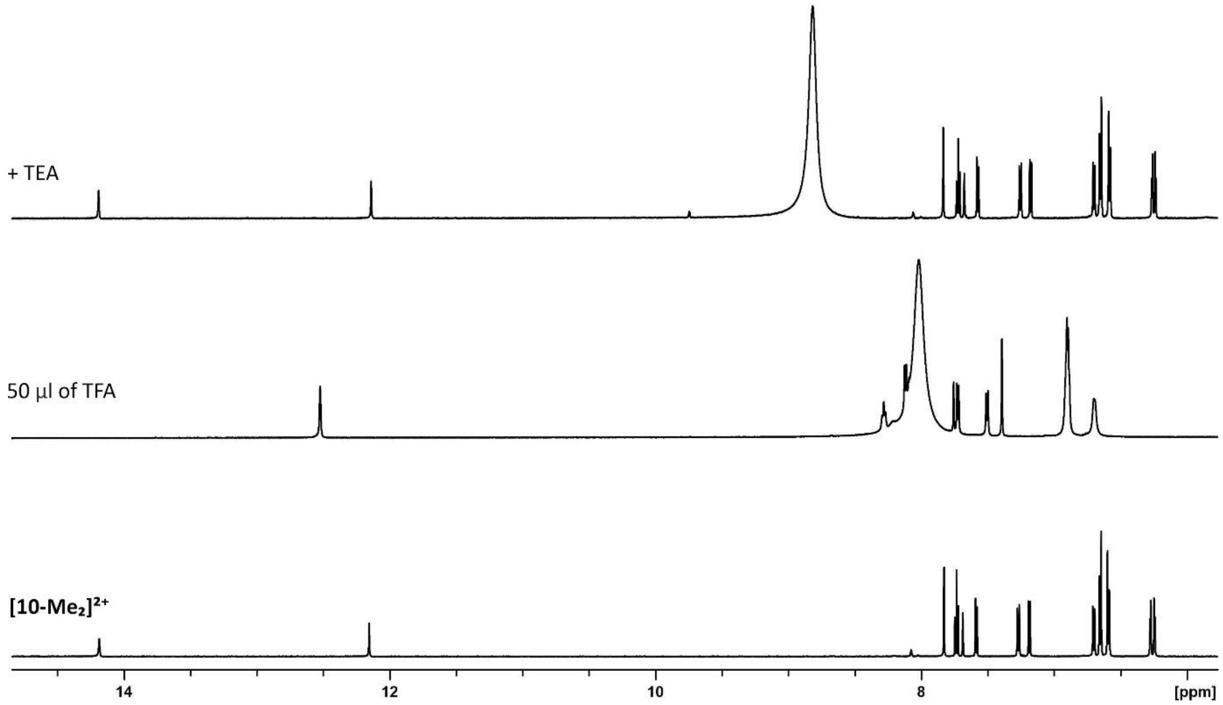

Figure S 136. The ^{1}H NMR spectra recorded upon the addition of 50 µl of trifluoroacetic acid to **[10-Me$_2$]$^{2+}$**, followed by the addition of TEA (600 MHz, [D$_2$]dichloromethane, 300 K).

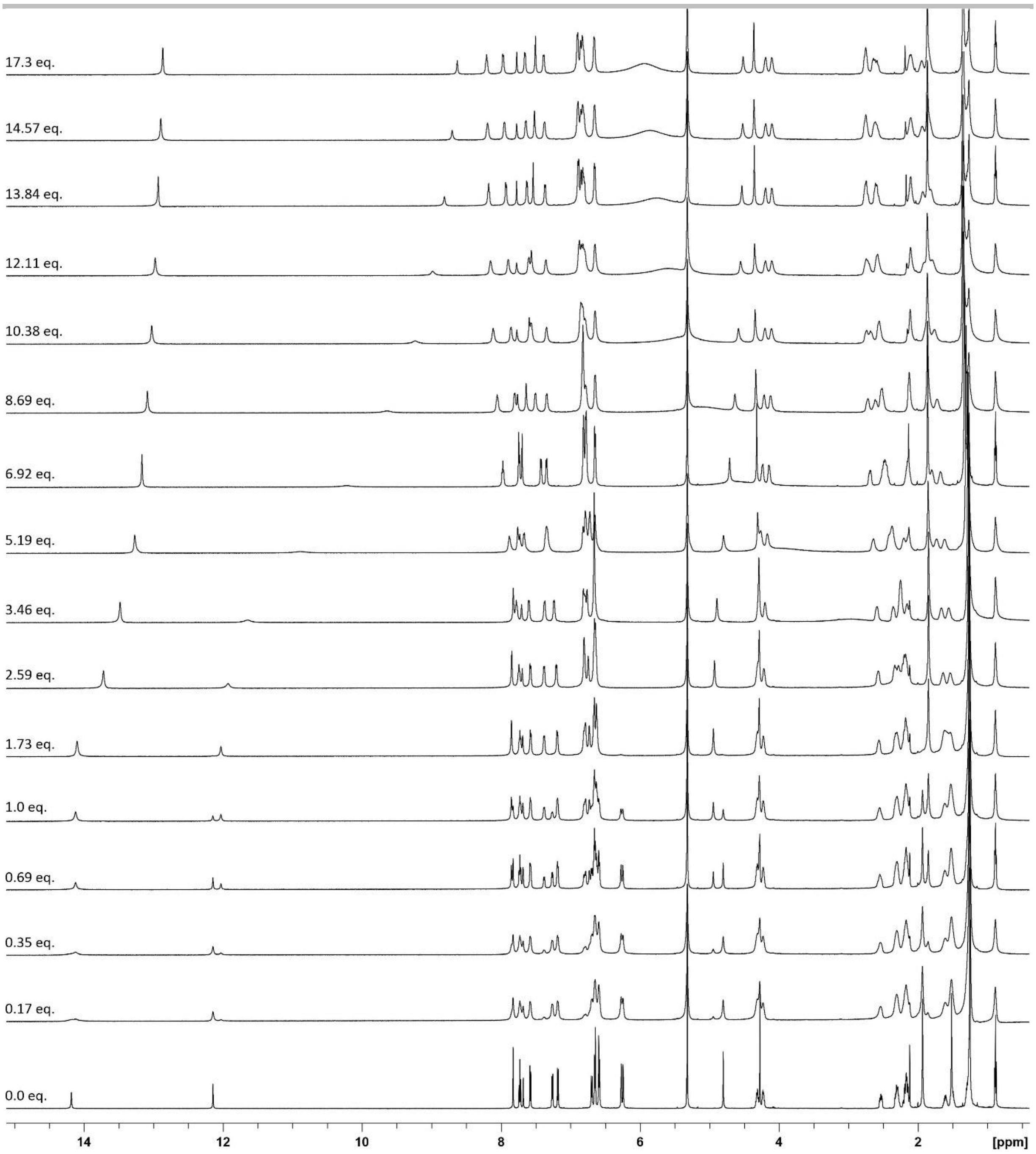

Figure S 137. The ^{1}H NMR spectra recorded during titration of **[10-Me$_2$]$^{2+}$** with trifluoroacetic acid (600 MHz, [D$_2$]dichloromethane, 300 K).

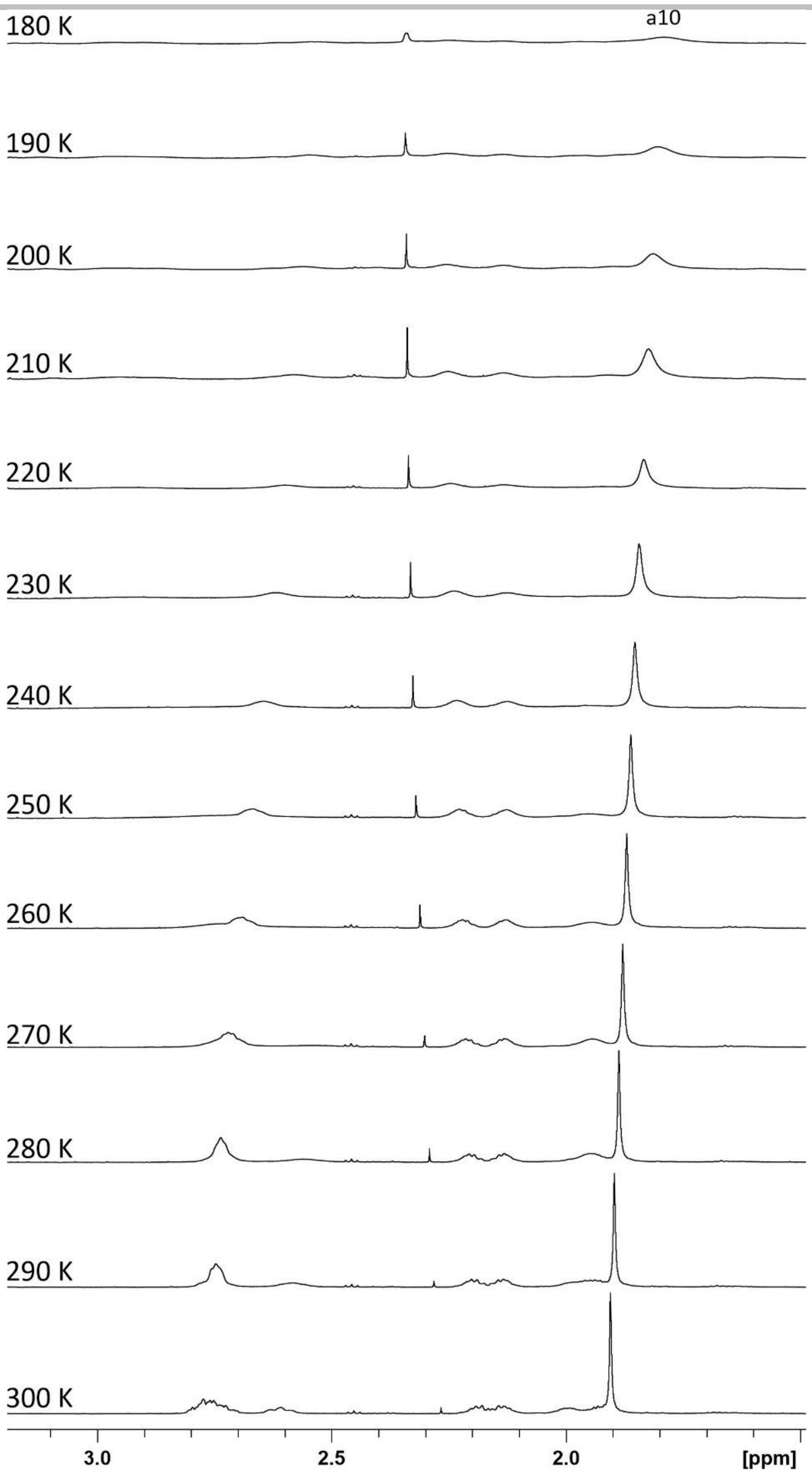

Figure S 138. The aliphatic region of the ^{1}H NMR spectra of **[10-Me$_2$H$_4$]$^{6+}$** recorded in the 300–180 K temperature range (600 MHz, [D$_2$]dichloromethane).

Compound [12-Me]+

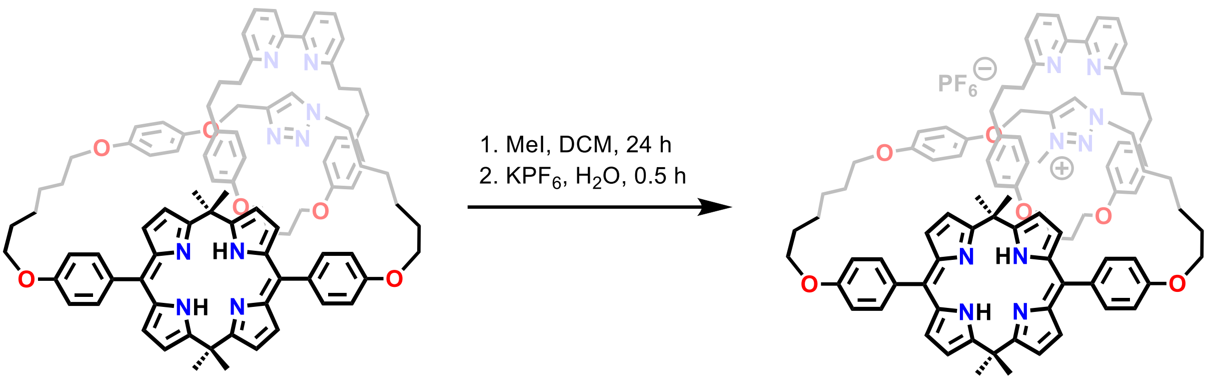

12 **[12-Me]+**; 83%

In a 10 ml vial with a screw cap, catenane **12** (15.2 mg, 11 μmol), MeI (1 ml) and DCM (1 ml) were introduced. The mixture was stirred for 24 hours at room temperature. After this time, KPF_6 (133 mg, 723 μmol) and H_2O (2 ml) were added, and the heterogenous mixture was stirred vigorously for another 30 minutes. After this time, the mixture was portioned between DCM (30 ml) and H_2O (30 ml) and then transferred into a separatory funnel. The aqueous phase was extracted with DCM (30 ml). The organic extracts were washed with water and brine. The collected organic layers were combined and dried over anhydrous Na_2SO_4. The filtrate was collected *via* gravity filtration, and the solvent was removed under reduced pressure to provide **[12-Me]+** (14.12 mg, 9.1 μmol, 83 %) as an orange solid.

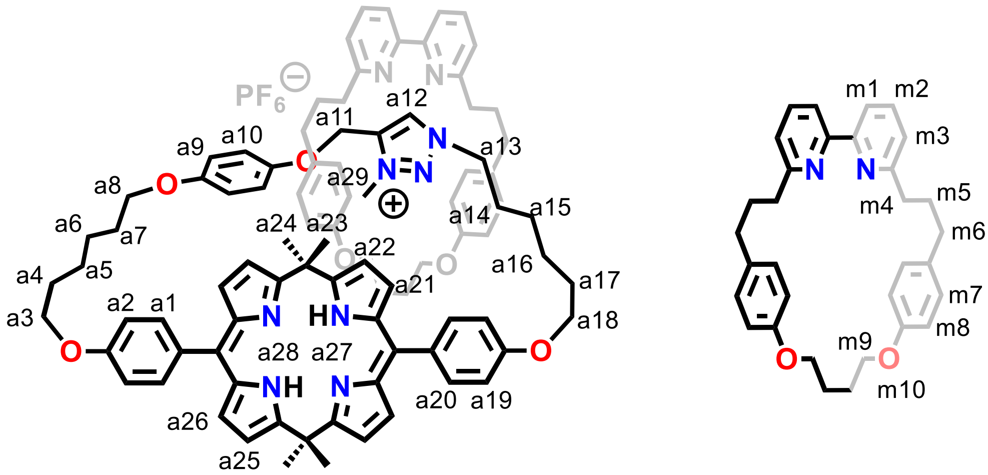

^{1}H NMR (600 MHz, [D]chloroform, 250 K, ppm): δ 14.50 (s, 1H, H_{a28}), 14.37 (s, 1H, H_{a27}), 10.17 (s, 1H, H_{a12}), 7.36 (dd, ^3J = 8.5 Hz, ^4J = 2.2 Hz, 1H, H_{a1}), 7.30 (t, ^3J = 7.8 Hz, 2H, H_{m2}), 7.26–7.23 (m, 2H, H_{a1}, H_{a20}), 7.10 (d, ^3J = 7.8 Hz, 2H, H_{m1}), 6.95 (d, ^3J = 7.8 Hz, 2H, H_{m3}), 6.91 (dd, J^3 = 8.5 Hz, J^4 = 2.6 Hz, 1H, H_{a2}), 6.83 (d, ^3J = 8.6 Hz, 4H, H_{m7}), 6.83–6.80 (overlapped, 1H, H_{a19}), 6.78 (dd, ^3J = 8.6 Hz, ^4J = 2.6 Hz, 1H, H_{a2}), 6.73 (dd, ^3J = 8.5 Hz, ^4J = 1.9 Hz, 1H, H_{a20}), 6.68 (d, ^3J = 8.6 Hz, 4H, H_{m8}), 6.62 (d, ^3J = 9.1 Hz, 2H, H_{a9}), 6.51 (d, ^3J = 9.1 Hz, 2H, H_{a10}), 6.42 (dd, ^3J = 4.1 Hz, ^4J = 0.8 Hz, 2H, H_{a26}), 6.27 (dd, ^3J = 4.1 Hz, ^4J = 0.8 Hz, 2H, H_{a25}), 6.21–6.17 (overlapped, 1H, H_{a19}), 6.19 (dd, ^3J = 4.1 Hz, ^4J = 0.8 Hz, 2H, H_{a21}), 6.14 (dd, ^3J = 4.1 Hz, ^4J = 0.8 Hz, 2H, H_{a22}), 4.53 (s, 2H, H_{a11}), 4.28 (s, 3H, H_{a29}), 4.09–4.01 (m, 6H, H_{a3}, H_{m9}), 3.86–3.78 (m, 4H, H_{a8}, H_{a18}), 3.24 (t, ^3J = 8.4 Hz, H_{a13}), 2.54–2.41 (m, 4H, H_{m4}), 2.27–2.19 (m, 2H, H_{m6}), 2.23 (s, 6H, H_{a23}), 2.15–2.99 (m, 6H, H_{m6}, H_{m10}) 1.84–1.76 (m, 2H, H_4), 1.76–1.68 (m, 4H, H_{a4}, H_{a7}), 1.73 (s, 6H, H_{a24}), 1.68–1.44 (m, 8H, H_{a5}, H_{a6}, H_{m5}), 1.44–1.36 (m, 2H, H_{a17}), 1.21–1.14 (m, 2H, H_{a16}), 1.01–0.98 (m, 2H, H_{a14}), 0.98–0.91 (m, 2H, H_{a15}); **^{13}C NMR** (125 MHz, [D]chloroform, 300 K): δ 165.5, 162.0, 159.9, 159.8, 157.6, 156.4, 154.7, 150.9, 140.5, 140.4, 140.2, 140.1, 138.6, 137.5, 133.9, 132.5, 132.24, 132.16, 129.8, 128.99, 128.97, 128.2, 127.9, 122.2, 120.5, 115.6, 115.4, 114.3, 114.1, 68.2, 67.83, 67.81, 66.3, 60.3, 58.2, 38.5, 38.4, 36.9, 34.8, 32.4, 31.6, 29.8, 28.7, 28.1, 27.9, 25.7, 25.1, 25.0, 24.9, 24.8, 23.6; **HR–ESI–MS** (*m/z*): $[M]^+$ calcd. for $C_{90}H_{98}N_9O_6^+$ 1401.7668; found 1401.7719.

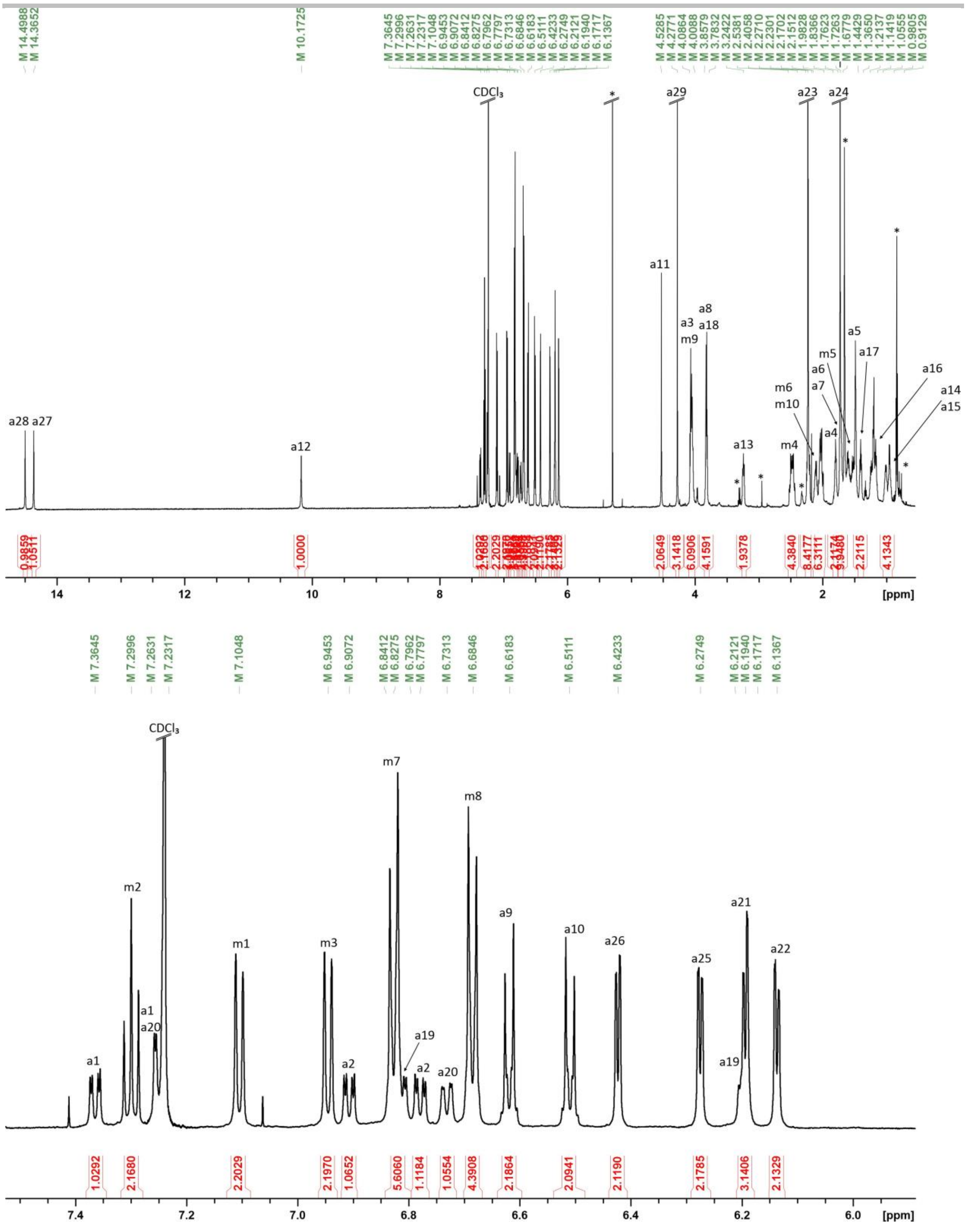

Figure S 139. The ^{1}H NMR spectrum of **[12-Me]⁺** (600 MHz, [D]chloroform, 250 K).

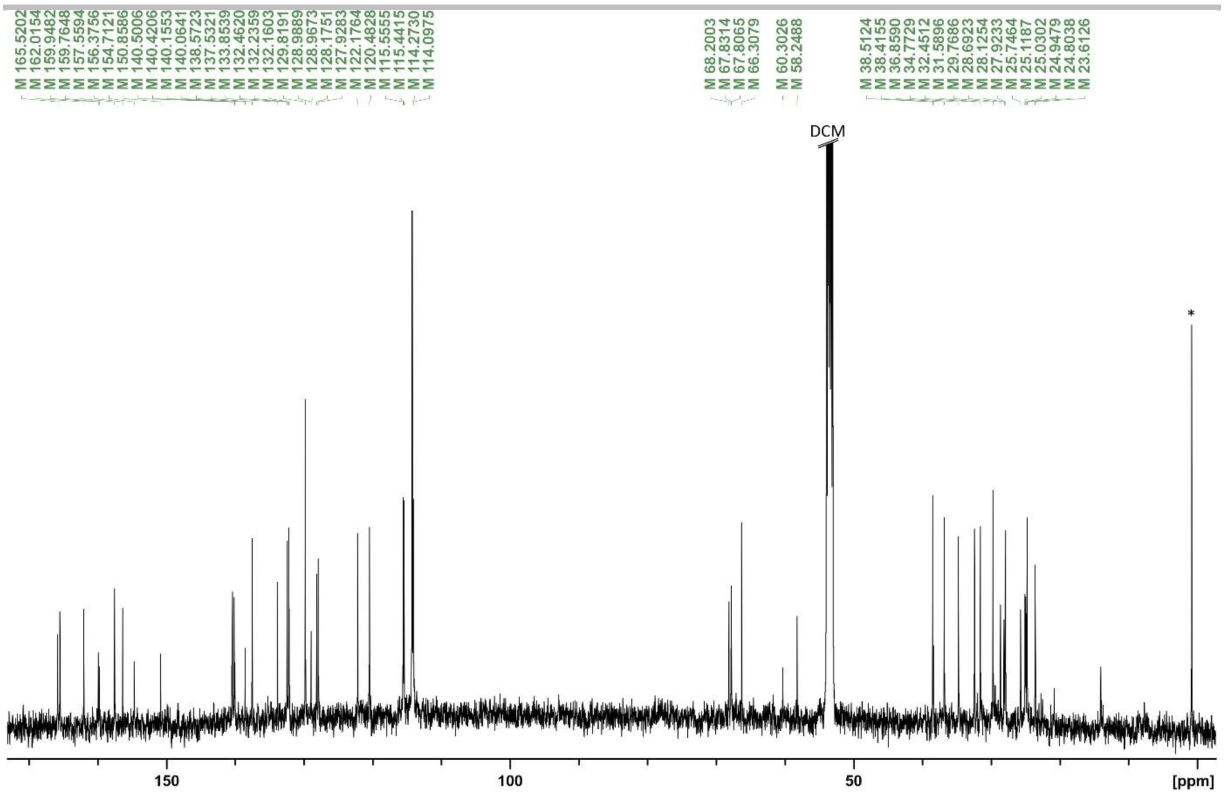

Figure S 140. The ^{13}C NMR spectrum of **[12-Me]⁺** (125 MHz, [D]chloroform, 250 K).

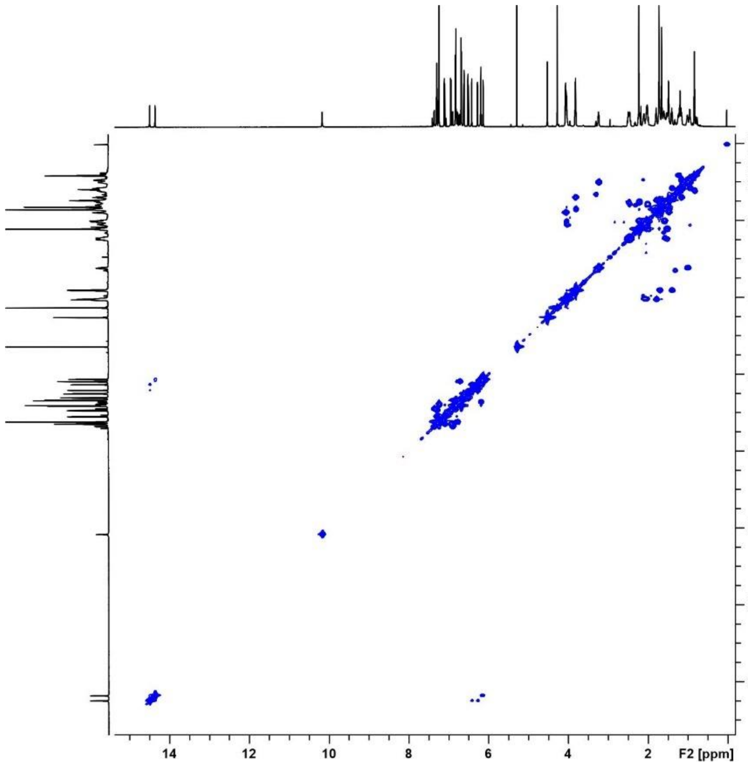

Figure S 141. The ^{1}H-^{1}H COSY NMR spectrum of **[12-Me]⁺** (600 MHz, [D]chloroform, 250 K).

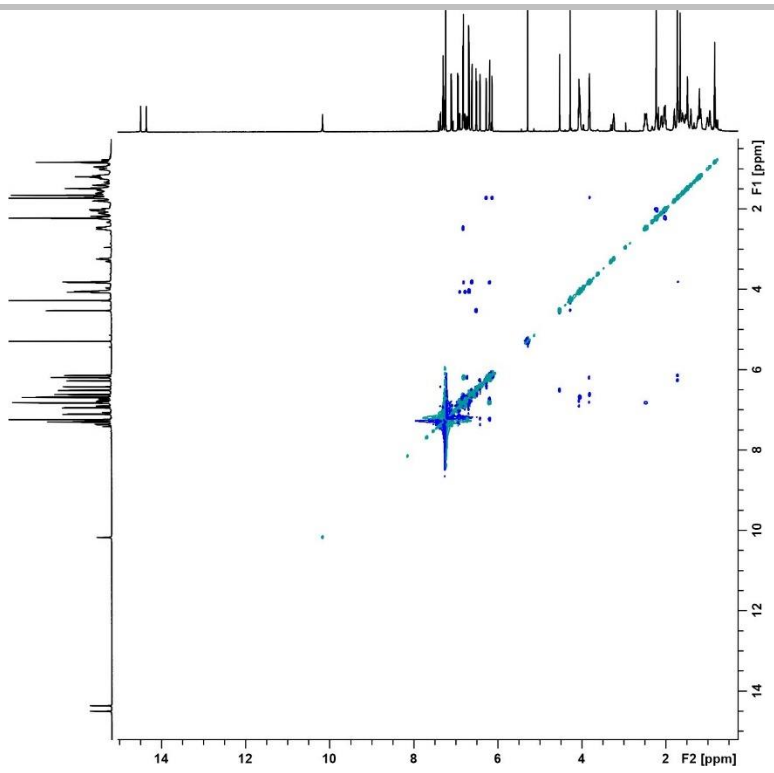

Figure S 142. The ^{1}H-^{1}H ROESY NMR spectrum of **[12-Me]⁺** (600 MHz, [D]chloroform, 250 K).

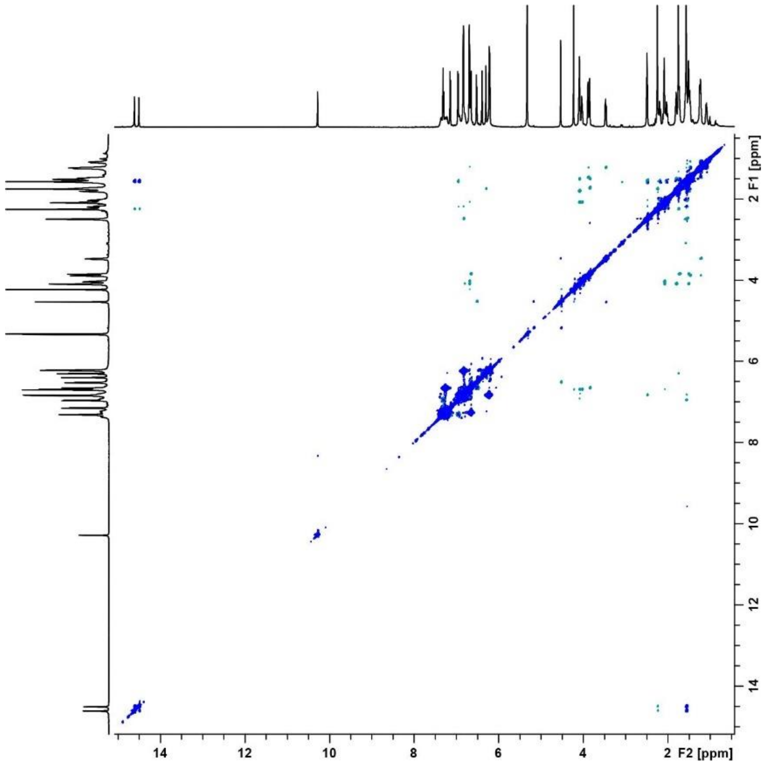

Figure S 143. The ^{1}H-^{1}H NOESY NMR spectrum of **[12-Me]⁺** (600 MHz, [D]chloroform, 280 K).

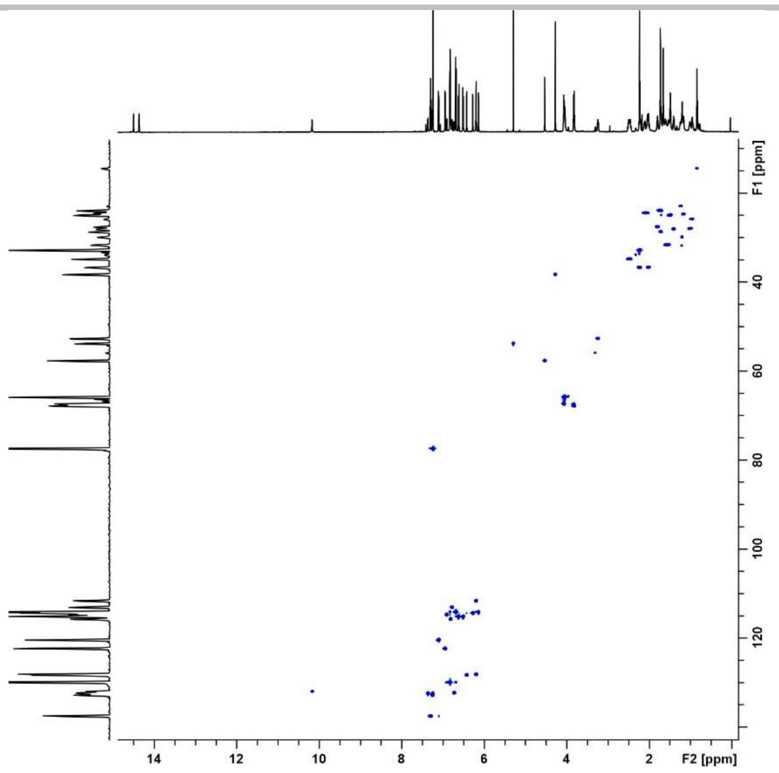

Figure S 144. The ^{1}H-^{13}C HSQC NMR spectrum of **[12-Me]⁺** (600 MHz, [D]chloroform, 250 K).

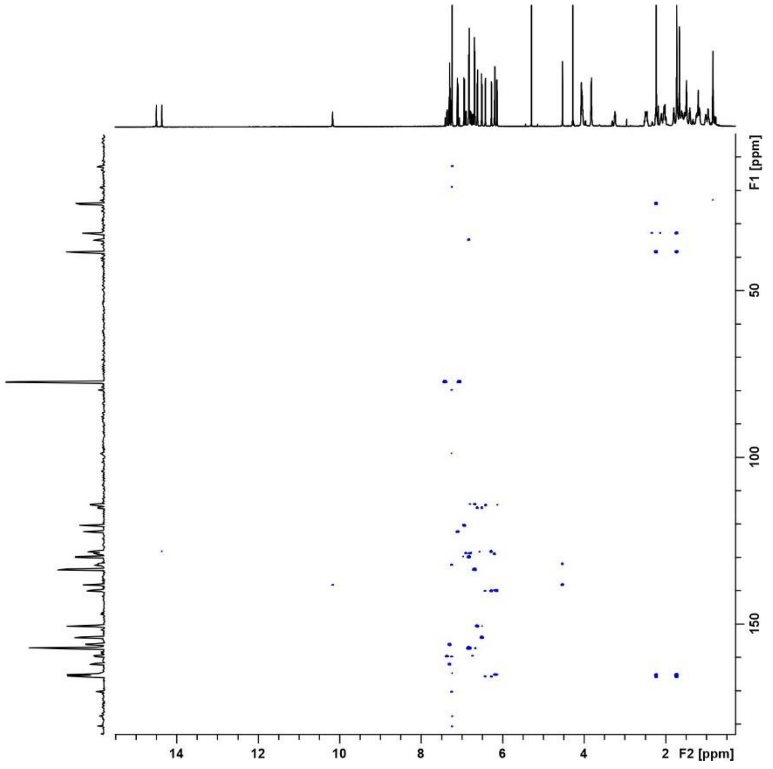

Figure S 145. The ^{1}H-^{13}C HMBC NMR spectrum of **[12-Me]⁺** (600 MHz, [D]chloroform, 250 K).

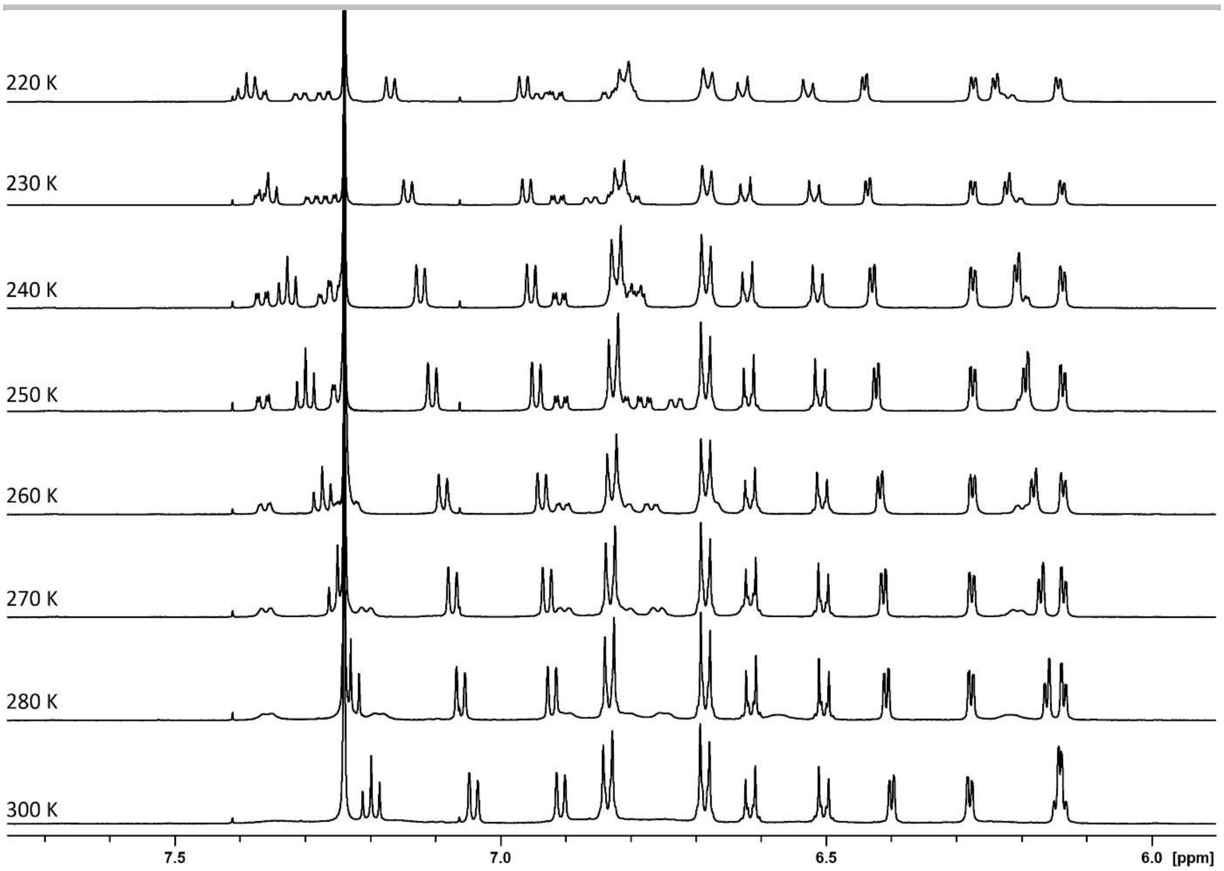

Figure S 146. The aromatic region of the ^{1}H NMR spectra of **[12-Me]⁺** recorded in the 300–220 K temperature range (600 MHz, [D]chloroform).

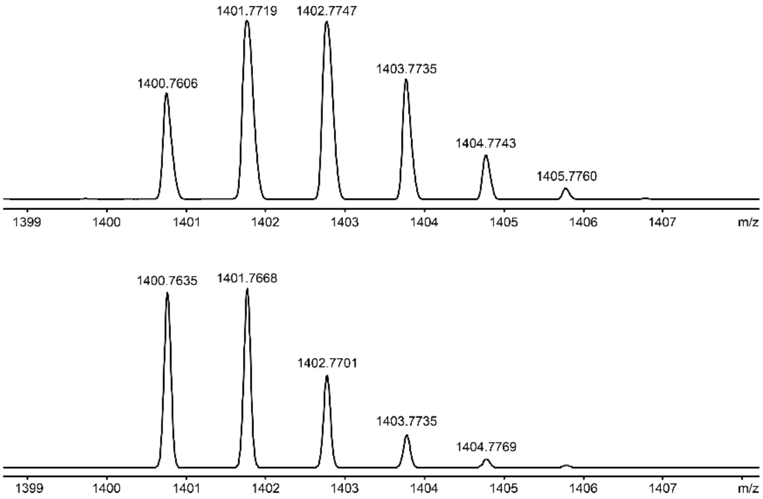

Figure S 147. The high-resolution ESI mass spectrum of **[12-Me]⁺**. Top: experimental spectrum, bottom: simulated isotopic pattern.

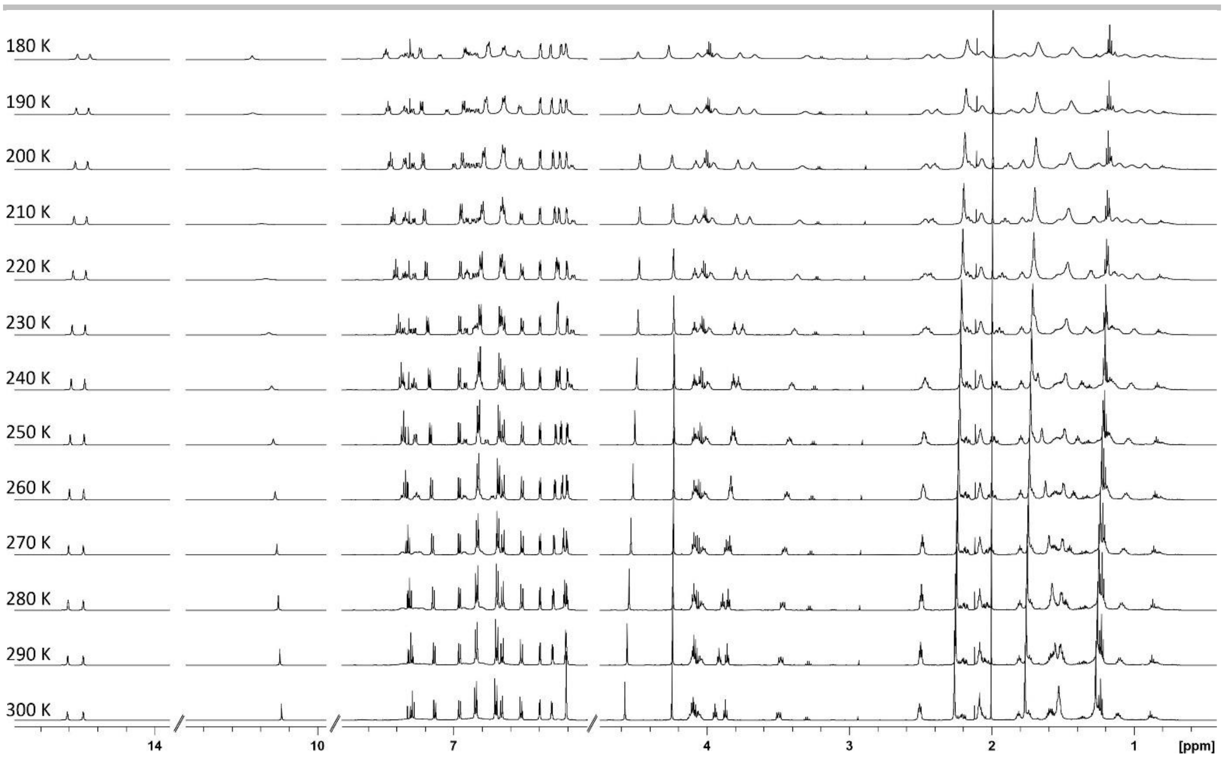

Figure S 148. The ^{1}H NMR spectra of **[12-Me]+** recorded in the 300–180 K temperature range (600 MHz, [D2]dichloromethane).

Compound [12-MeH₂]$^{3+}$

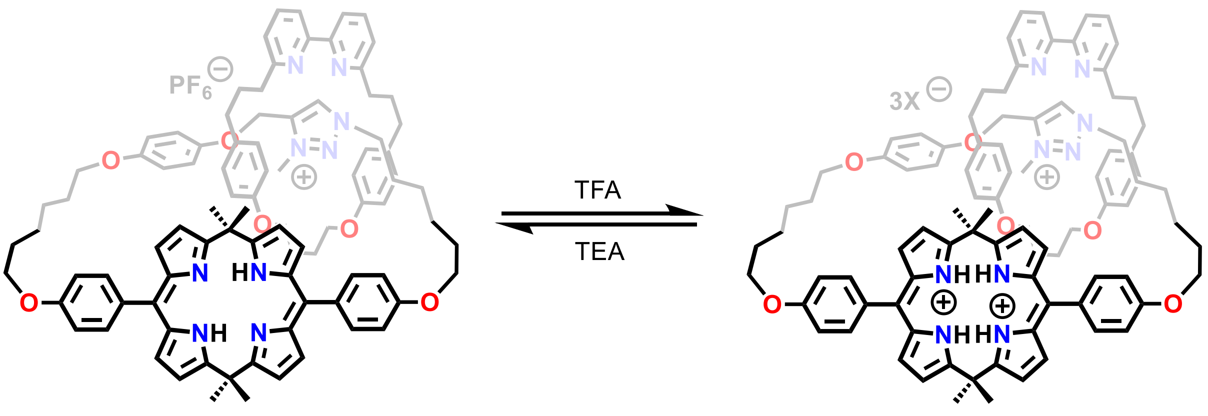

[12-Me]$^+$ **[12-MeH$_2$]$^{3+}$**

The cationic species **[12-MeH$_2$]$^{3+}$** was obtained by acidification of **[12-Me]$^+$** with a ca. two equiv. of trifluoroacetic acid in [D$_2$]dichloromethane.

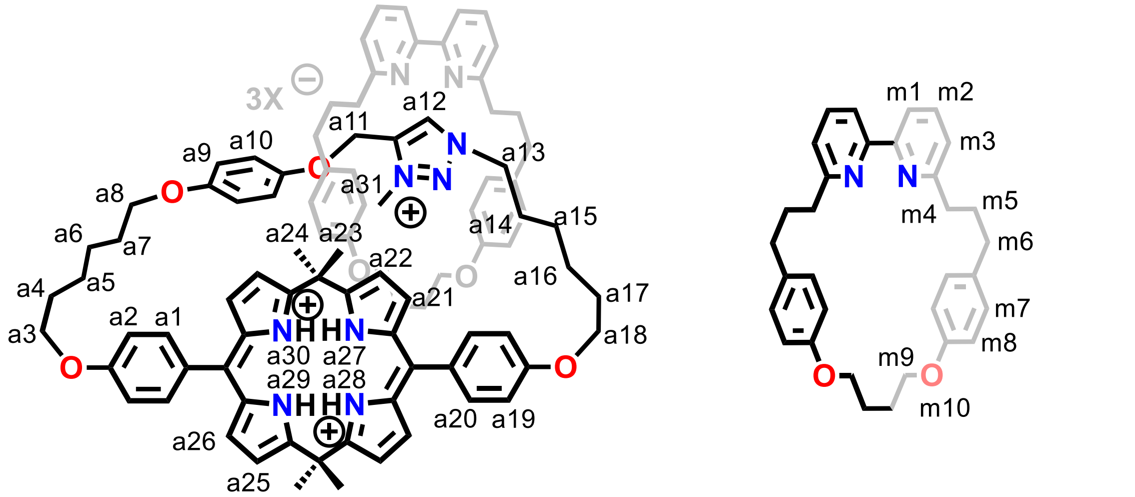

^{1}**H NMR** (600 MHz, [D$_2$]dichloromethane, 180 K, ppm; only selected signals could be unambiguously assigned and only those are listed): δ 13.92, 13.90, 13.66, 13.13 (calix[4]phyrin NH signals), 10.24 (s, 1H, H$_{a12}$), 7.13, 6.95, 6.76, 6.73, 6.71, 6.64, 6.47, 6.37 (β–pyrrolic signals, 8H), 4.24 (s, 3H, H$_{a31}$).

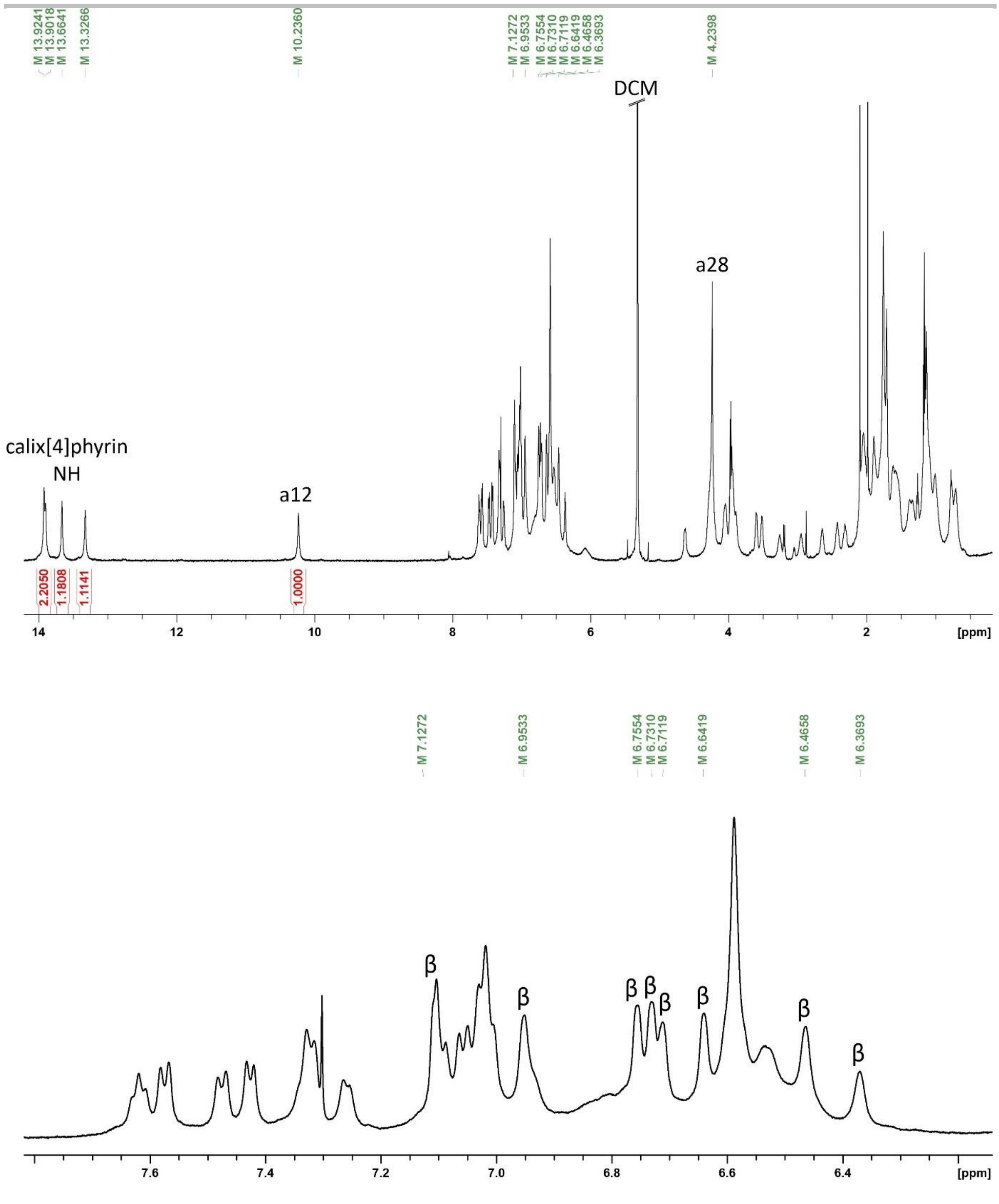

Figure S 149. The ^{1}H NMR spectrum of **$[12\text{-}MeH_2]^{3+}$** (600 MHz, $[D_2]$dichloromethane, 180 K).

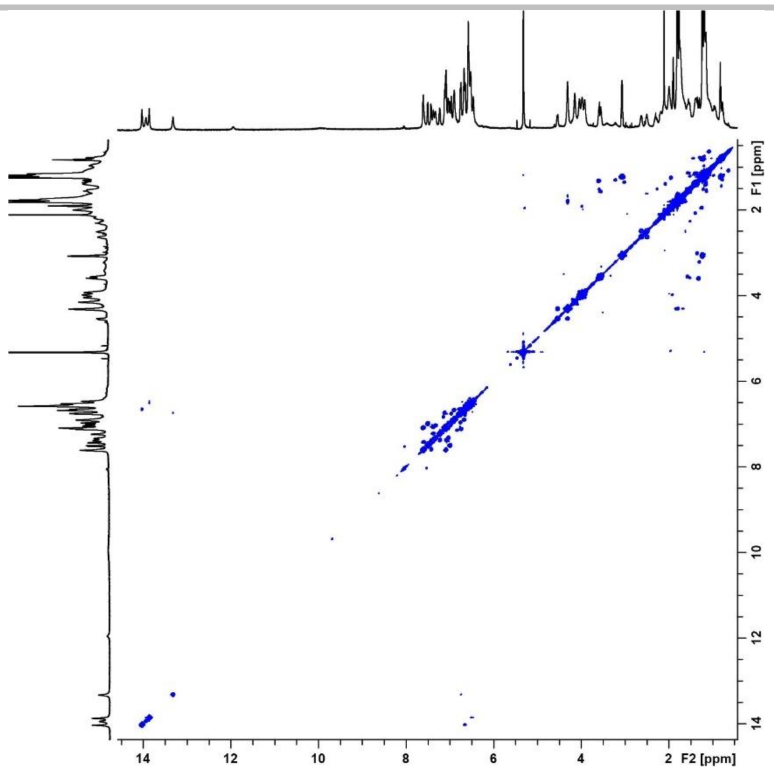

Figure S 150. The ^{1}H-^{1}H COSY NMR spectrum of **[12-MeH$_2$]$^{3+}$** (600 MHz, [D$_2$]dichloromethane, 230 K).

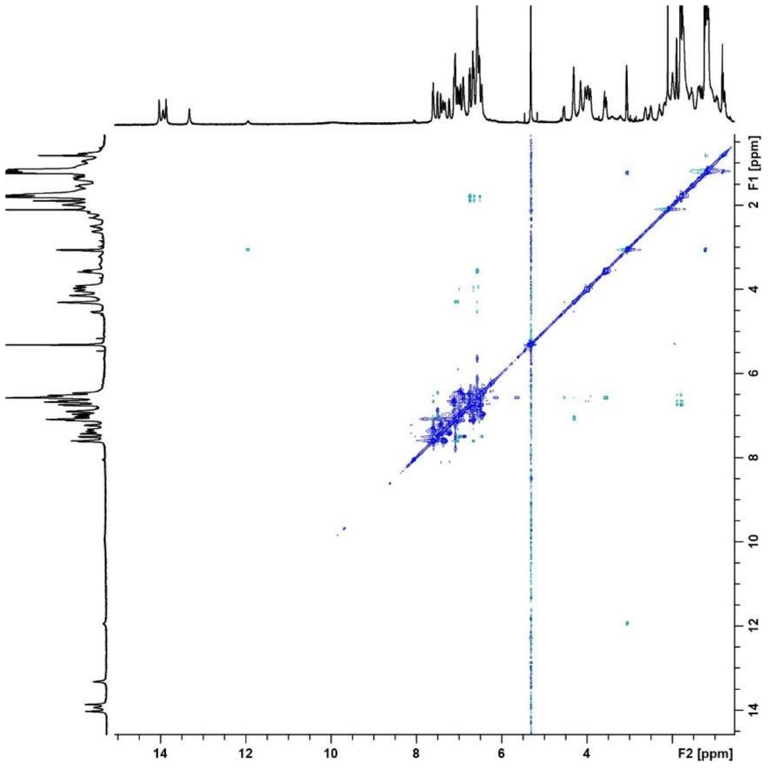

Figure S 151. The ^{1}H-^{1}H NOESY NMR spectrum of **[12-MeH$_2$]$^{3+}$** (600 MHz, [D$_2$]dichloromethane, 230 K).

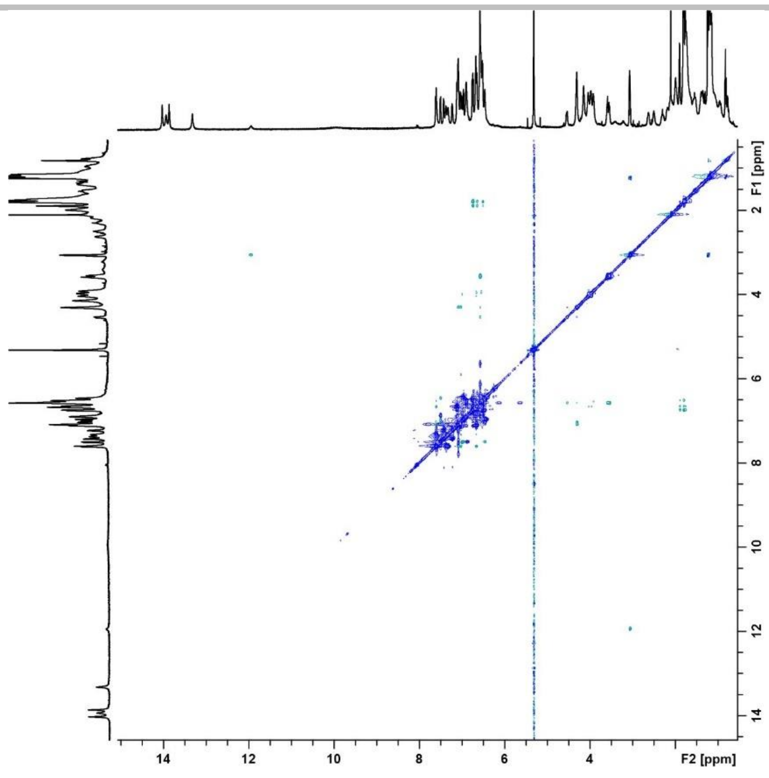

Figure S 152. The ^{1}H-^{1}H ROESY NMR spectrum of **[12-MeH$_2$]$^{3+}$** (600 MHz, [D$_2$]dichloromethane, 230 K).

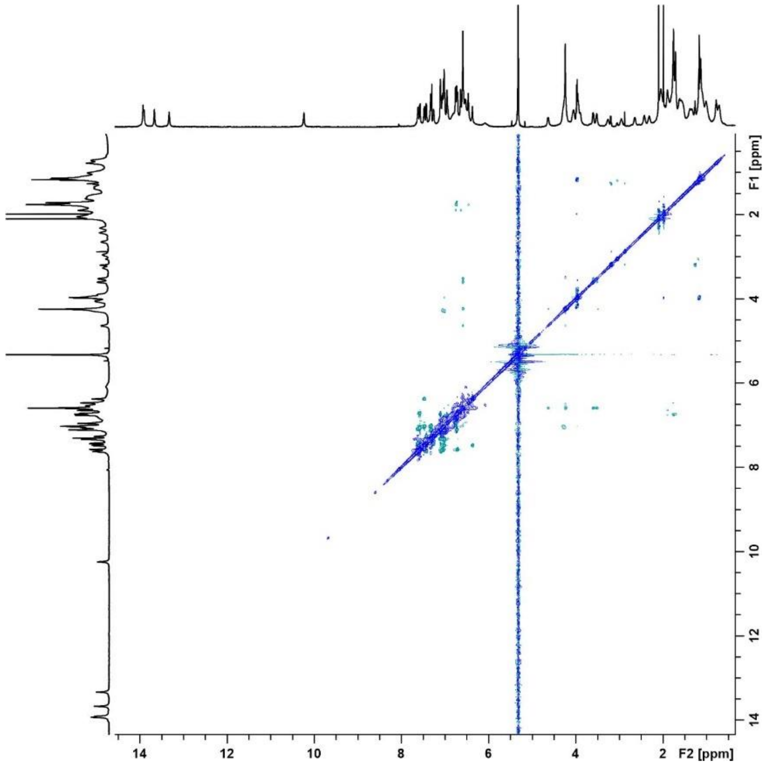

Figure S 153. The ^{1}H-^{1}H ROESY NMR spectrum of **[12-MeH$_2$]$^{3+}$** (600 MHz, [D$_2$]dichloromethane, 180 K).

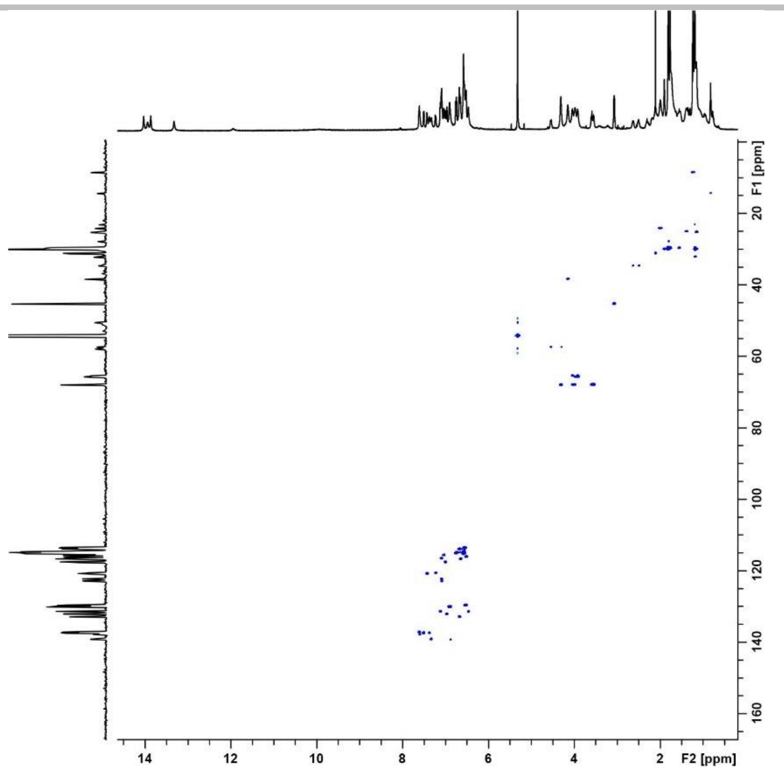

Figure S 154. The ^{1}H-^{13}C HSQC NMR spectrum of **[12-MeH$_2$]$^{3+}$** (600 MHz, [D$_2$]dichloromethane, 230 K).

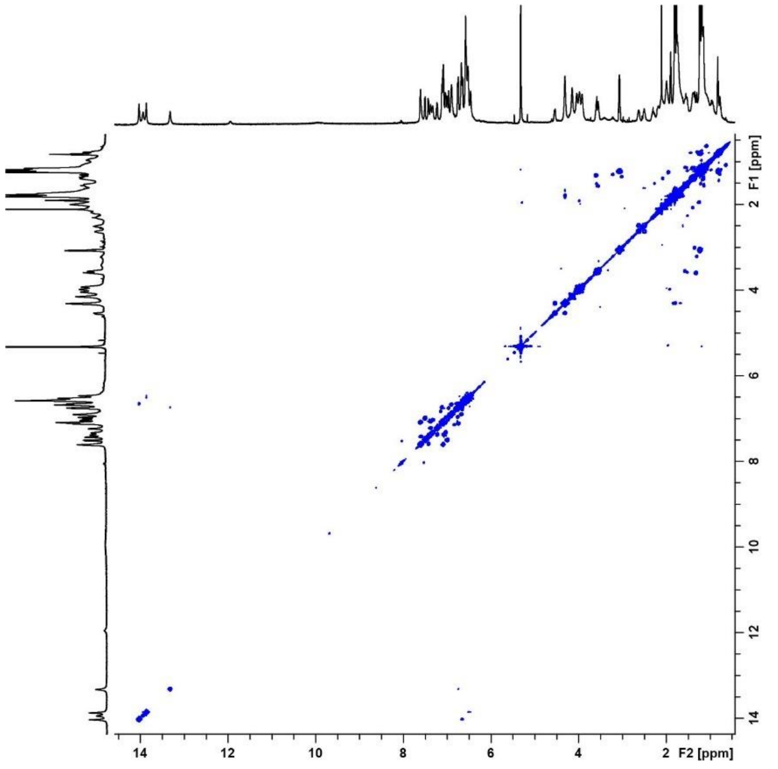

Figure S 155. The ^{1}H-^{1}H COSY NMR spectrum of **[12-MeH$_2$]$^{3+}$** (600 MHz, [D$_2$]dichloromethane, 180 K).

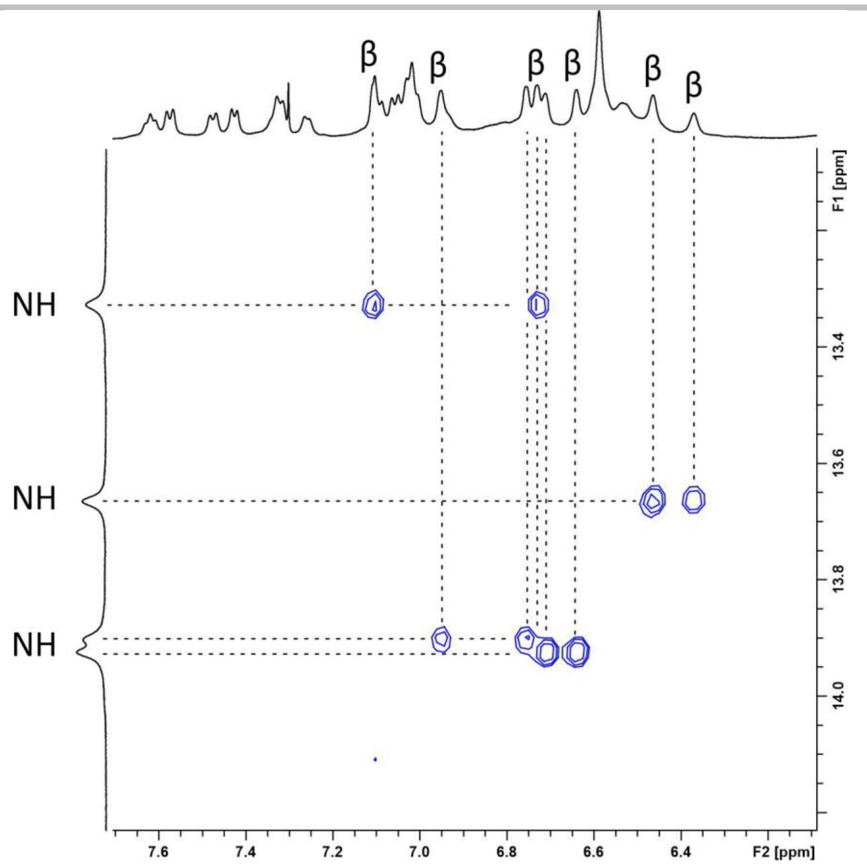

Figure S 156. The ^{1}H-^{1}H COSY NMR spectrum of **[12-MeH₂]$^{3+}$** (600 MHz, [D$_2$]dichloromethane, 180 K). The scalar couplings between the NH and β-pyrrolic protons were labelled with dashed lines.

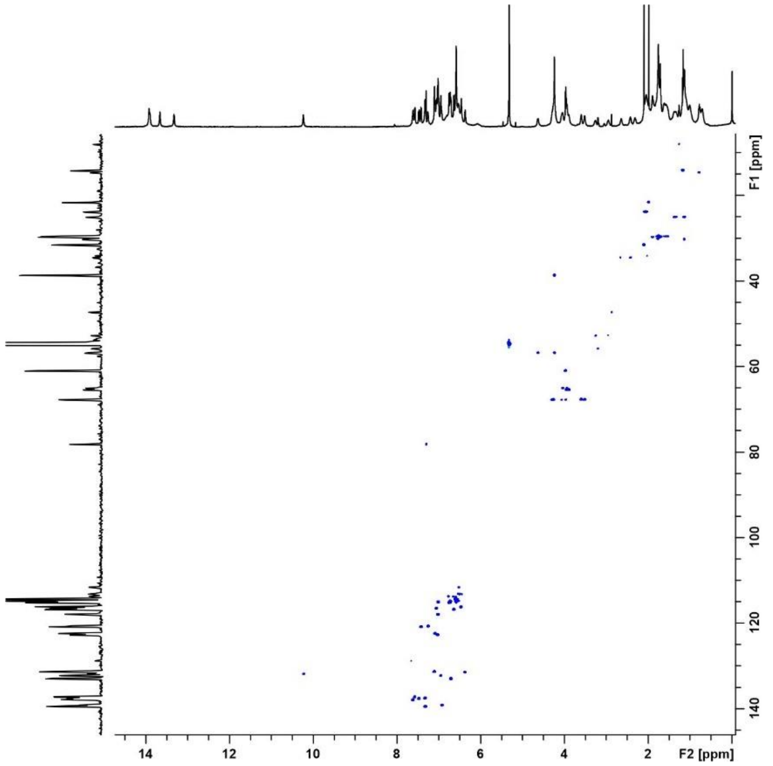

Figure S 157. The ^{1}H-^{13}C HSQC NMR spectrum of **[12-MeH$_2$]$^{3+}$** (600 MHz, [D$_2$]dichloromethane, 180 K).

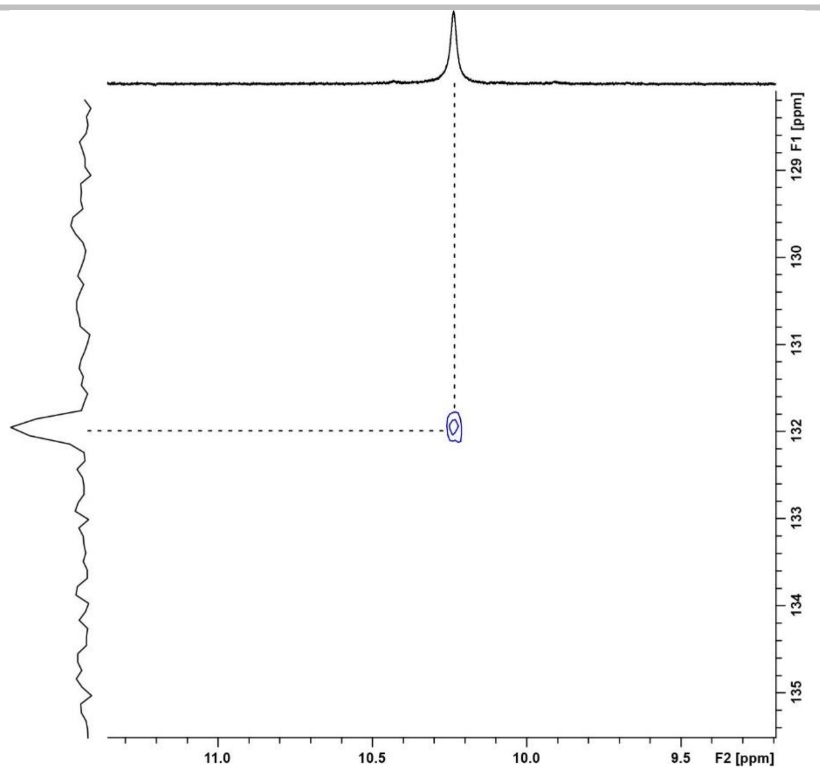

Figure S 158. The ^{1}H-^{13}C HSQC NMR spectrum of **$[12\text{-}MeH_2]^{3+}$** (600 MHz, $[D_2]$dichloromethane, 180 K). The C−H correlation between the triazole proton and the corresponding carbon was marked with a dashed line.

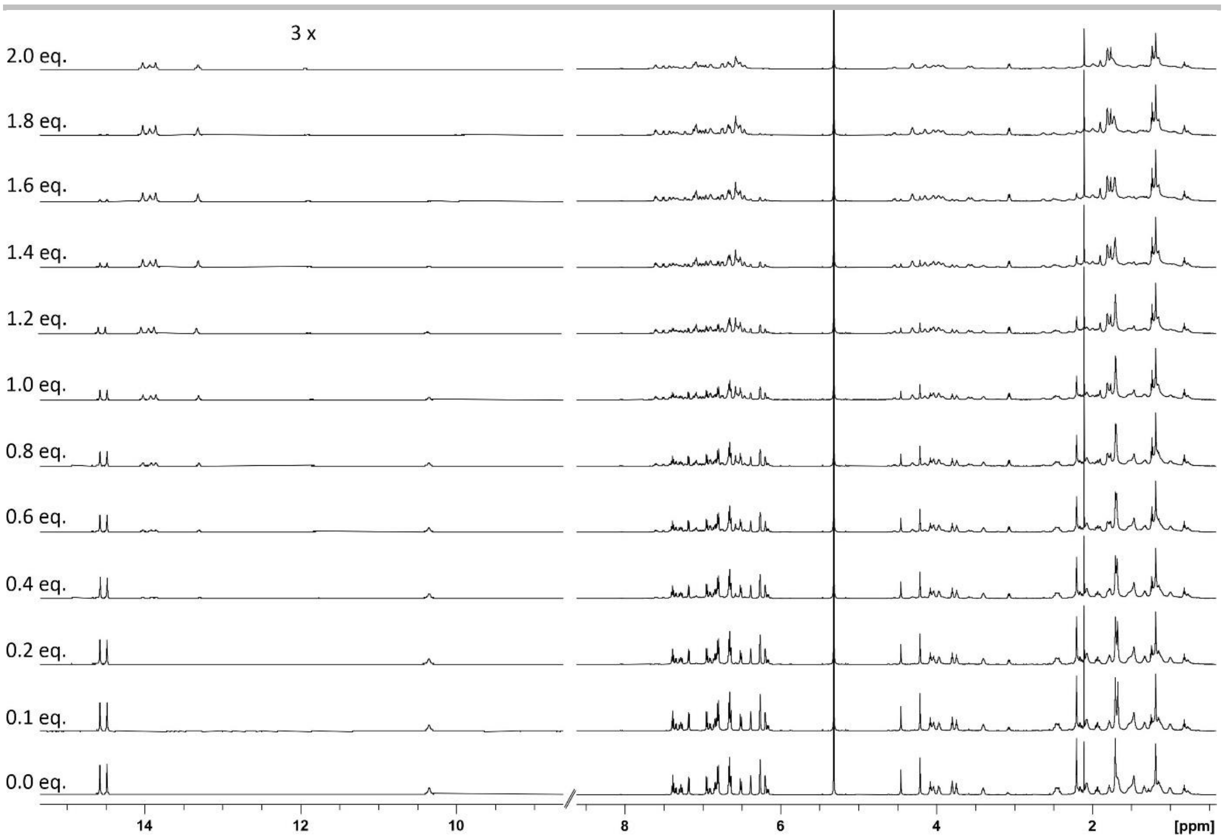

Figure S 159. The ^{1}H NMR spectra recorded during titration of **[12-Me]$^{+}$** with trifluoroacetic acid (600 MHz, [D_2]dichloromethane, 230 K).

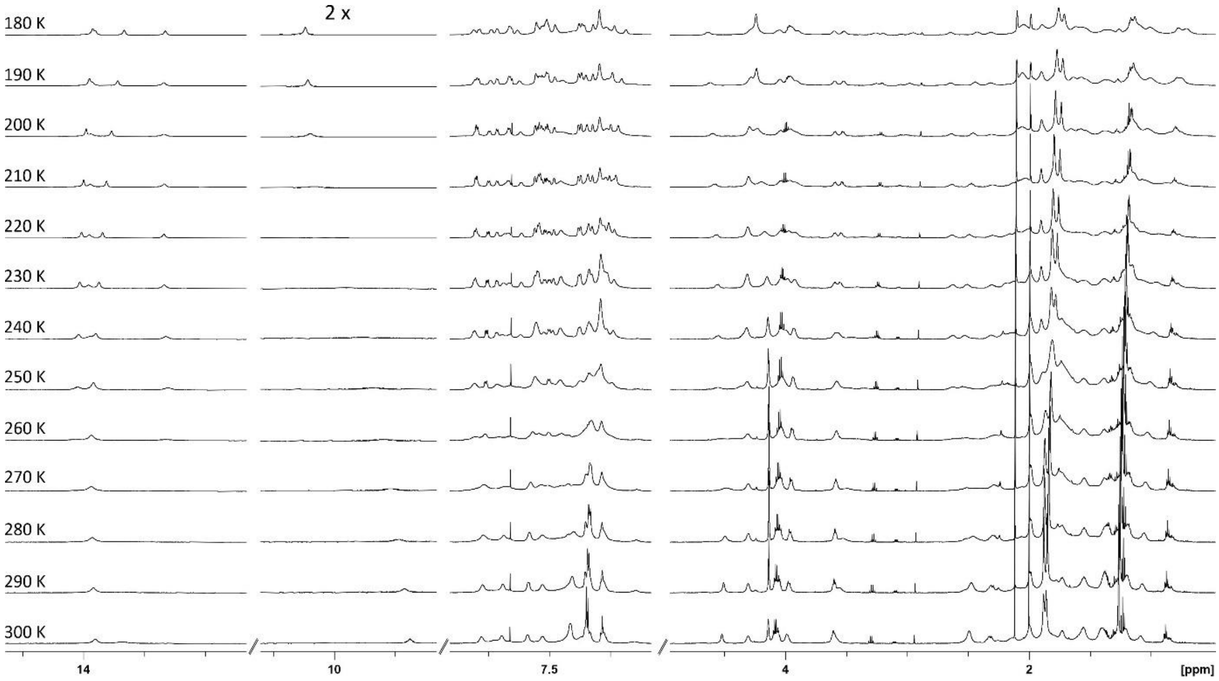

Figure S 160. The aromatic region of the ^{1}H NMR spectra of **[12-MeH$_2$]$^{3+}$** recorded in the 300–180 K temperature range (600 MHz, [D_2]dichloromethane).

Compound [12-MeH$_3$]$^{4+}$

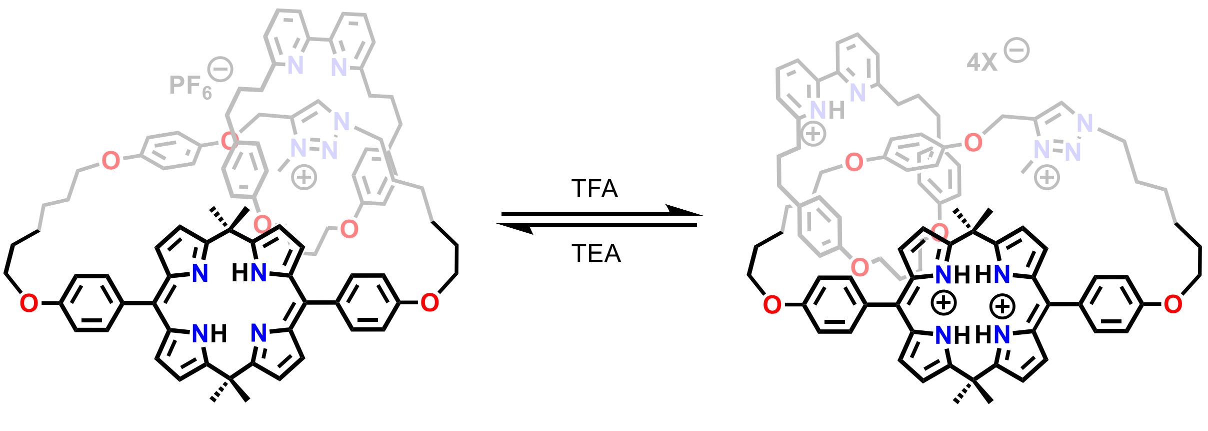

[12-Me]$^+$ **[12-MeH$_3$]$^{4+}$**

The cationic species **[12-MeH$_3$]$^{4+}$** was obtained by acidification of **[12-Me]$^+$** with an excess (4–5 equiv.) of trifluoroacetic acid in [D$_2$]dichloromethane.

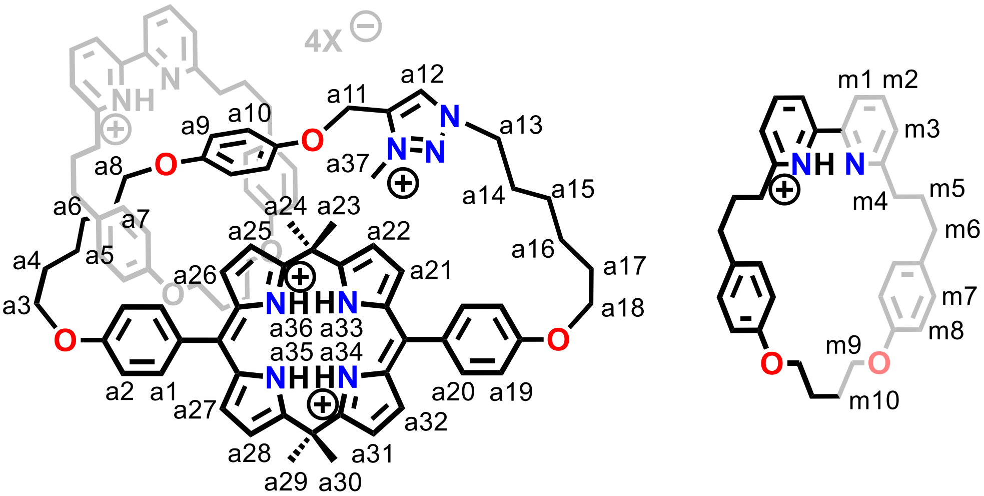

^{1}H NMR (600 MHz, [D$_2$]dichloromethane, 230 K, ppm): δ 10.98–10.82 (overlapped s, 4H, H_{a33-36}), 8.34 (t, ^3J = 8.0 Hz, 1H, H_{m2}), 8.40 (t, ^3J = 8.0 Hz, 1H, H_{m2}), 8.21 (s, 1H, H_{a12}), 7.97–7.92 (m, 2H, H_{m1}), 7.86 (d, ^3J = 8.2 Hz, 1H, H_{m3}), 7.77 (d, ^3J = 8.2 Hz, 1H, H_{m3}), 7.61 (d, ^3J = 8.8 Hz, 1H, H_{a1}/H_{a20}), 7.55 (d, ^3J = 8.8 Hz, 1H, H_{a1}/H_{a20}), 7.52 (d, ^3J = 8.8 Hz, 1H, H_{a1}/H_{a20}), 7.31 (d, ^3J = 8.8 Hz, 1H, H_{a1}/H_{a20}), 7.25 (broad signal, 1H, H_{a21}/H_{a26}/H_{a27}/H_{a32}), 7.21 (d, ^3J = 8.8 Hz, 1H, H_{a2}/H_{a19}), 7.18 (broad signal, 1H, H_{a21}/H_{a26}/H_{a27}/H_{a32}), 7.16 (d, ^3J = 8.8 Hz, 1H, H_{a2}/H_{a19}), 7.09 (d, ^3J = 8.8 Hz, 1H, H_{a2}/H_{a19}), 7.01 (d, ^3J = 8.8 Hz, 1H, H_{a2}/H_{a19}), 6.95 (d, ^3J = 7.9 Hz, 2H, H_{m7}), 6.91 (d, ^3J = 7.9 Hz, 2H, H_{m7}), 6.82 (broad signal, 1H, H_{a22}/H_{a25}/H_{a28}/H_{a31}), 6.80 (broad signal, 1H, H_{a22}/H_{a25}/H_{a28}/H_{a31}), 6.77 (broad signal, 1H, H_{a21}/H_{a26}/H_{a27}/H_{a32}), 6.56 (broad signal, 1H, H_{a22}/H_{a25}/H_{a28}/H_{a31}), 6.47 (d, ^3J = 7.9 Hz, 2H, H_{m8}), 6.41 (d, ^3J = 8.7 Hz, 2H, H_{a10}), 6.38 (broad signal, 1H, H_{a22}/H_{a25}/H_{a28}/H_{a31}), 6.29 (d, ^3J = 7.9 Hz, 2H, H_{m8}), 6.03 (broad signal, 1H, H_{a21}/H_{a26}/H_{a27}/H_{a32}), 5.39 (d, ^3J = 8.7 Hz, 2H, H_{a9}), 5.05 (d, ^3J = 12.7 Hz, 1H, H_{a11}), 4.92 (d, ^3J = 12.7 Hz, 1H, H_{a11}), 4.53–4.22 (m, 6H, H_{a3}, H_{a13}, H_{a18}), 4.19 (s, 3H, H_{a37}), 3.70–3.63 (m, 1H, H_{m9}), 3.49–3.41 (m, 1H, H_{m9}), 3.41–3.33 (m, 1H, H_{m9}), 3.15–3.08 (m, 1H, H_{m9}), 3.01–2.92 (m, 2H, H_{m5}, H_{m6}), 2.92–2.78 (m, 4H, H_{a8}, H_{m5}, H_{m6}), 2.71–2.48 (m, 3H, H_{m5}, H_{m6}), 2.48–2.47 (m, 1H, H_{m6}), 2.25–2.14 (m, 1H, H_{m4}), 2.14–2.03 (m, 1H, H_{m4}), 1.84 (s, 3H, H_{a24}/ H_{a29}), 1.82 (s, 3H, H_{a24}/ H_{a29}),1.76 (s, 3H, H_{a23}/H_{a30}), 1.74 (s, 3H, H_{a23}/H_{a30}), 2.03–1.62 (aliphatic region), 1.49–0.94 (aliphatic region).

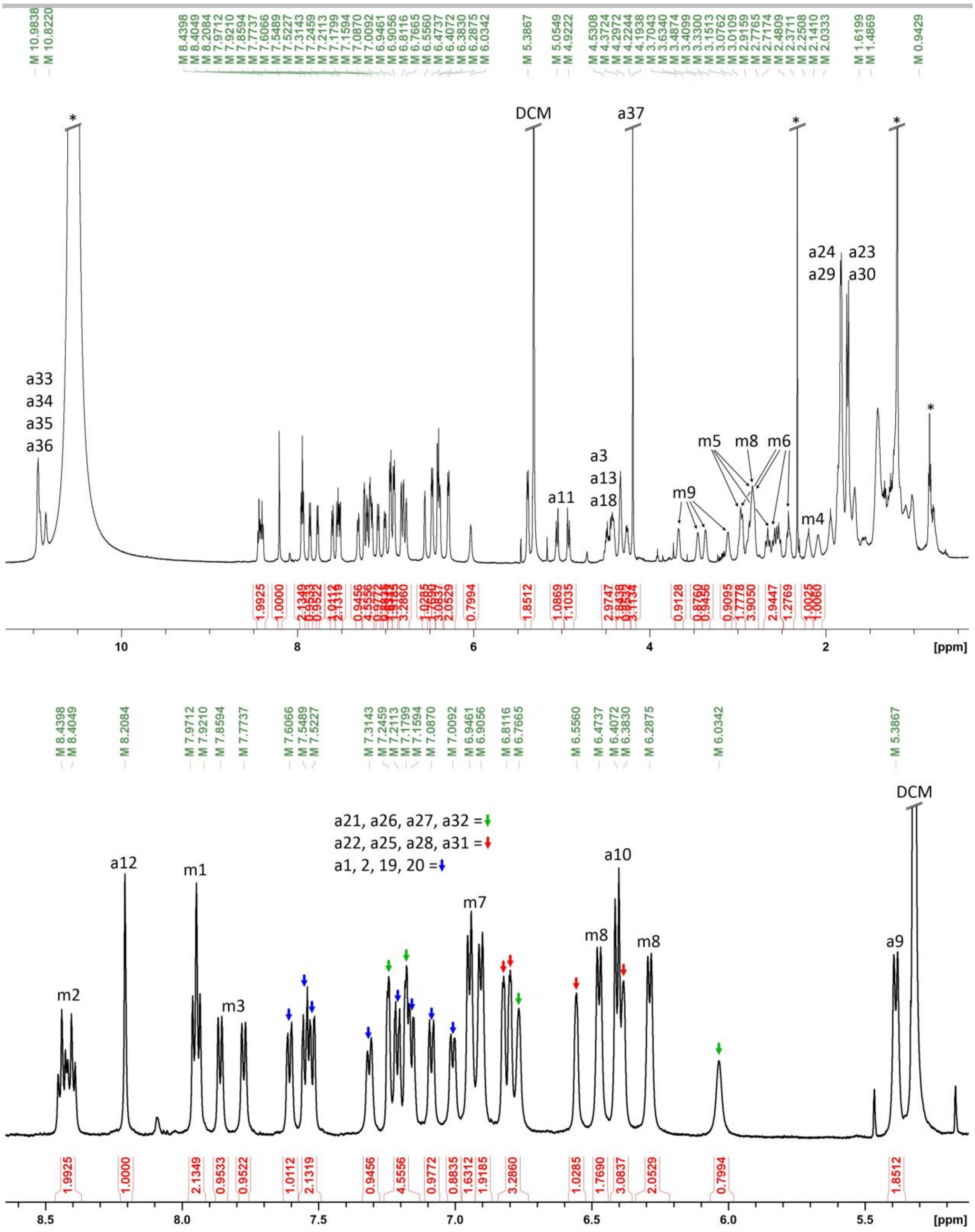

Figure S 161. The ^{1}H NMR spectrum of **$[12\text{-}MeH_3]^{4+}$** (600 MHz, [D$_2$]dichloromethane, 230 K).

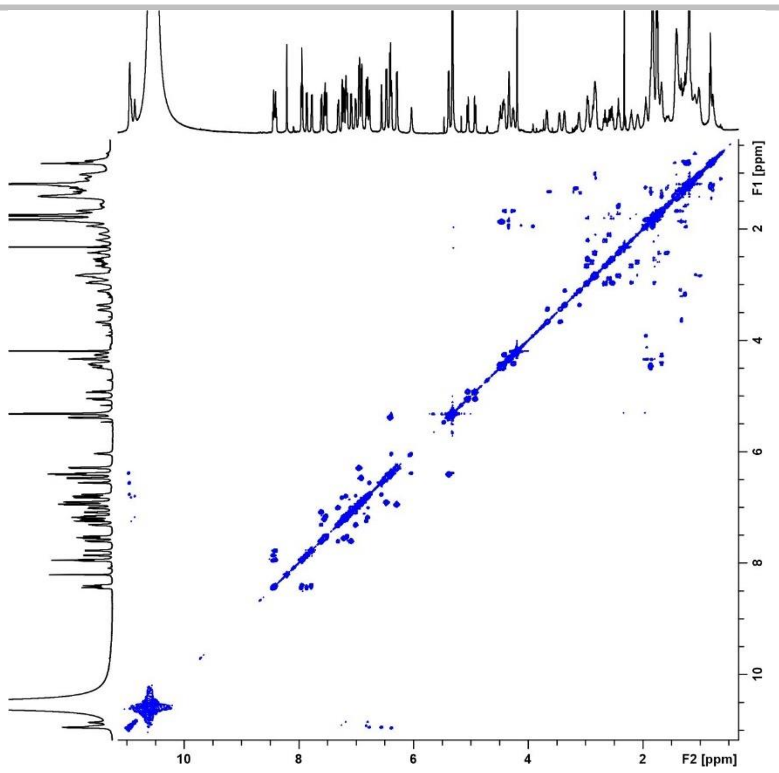

Figure S 162. The ^{1}H-^{1}H COSY NMR spectrum of **[12-MeH$_3$]$^{4+}$** (600 MHz, [D$_2$]dichloromethane, 230 K).

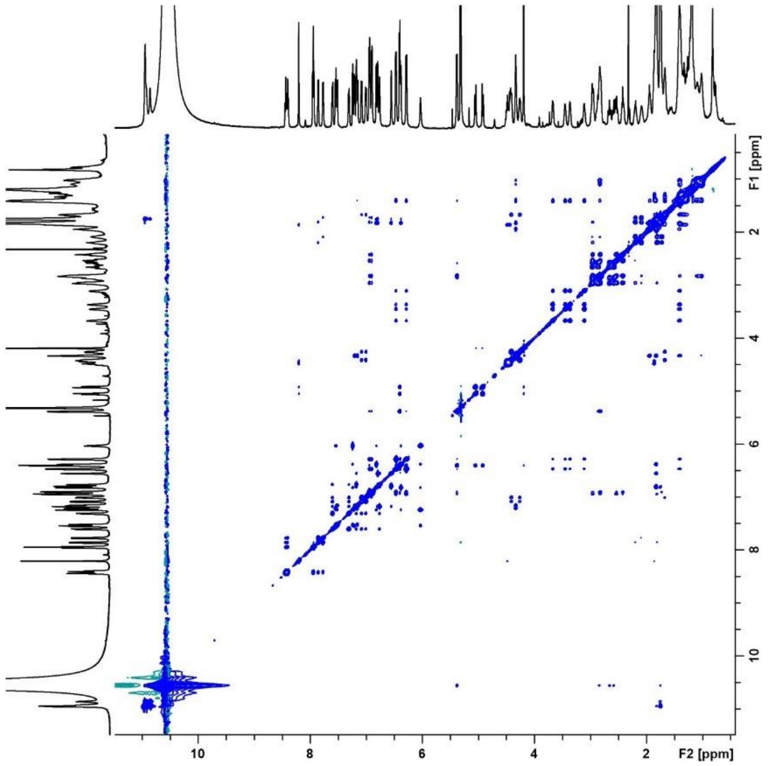

Figure S 163. The ^{1}H-^{1}H NOESY NMR spectrum of **[12-MeH$_3$]$^{4+}$** (600 MHz, [D$_2$]dichloromethane, 230 K).

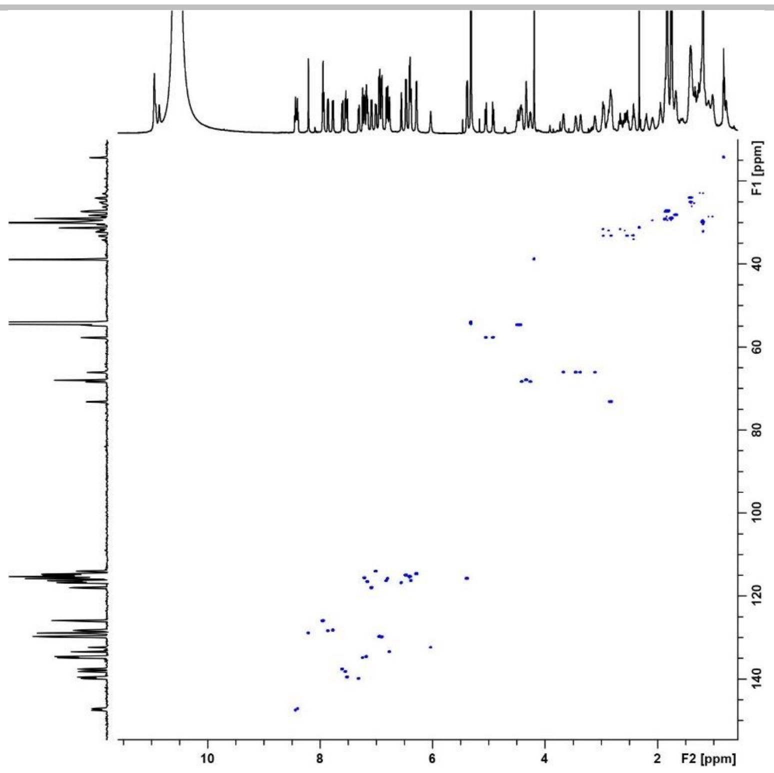

Figure S 164. The ¹H-¹³C HSQC NMR spectrum of **[12-MeH$_3$]$^{4+}$** (600 MHz, [D$_2$]dichloromethane, 230 K).

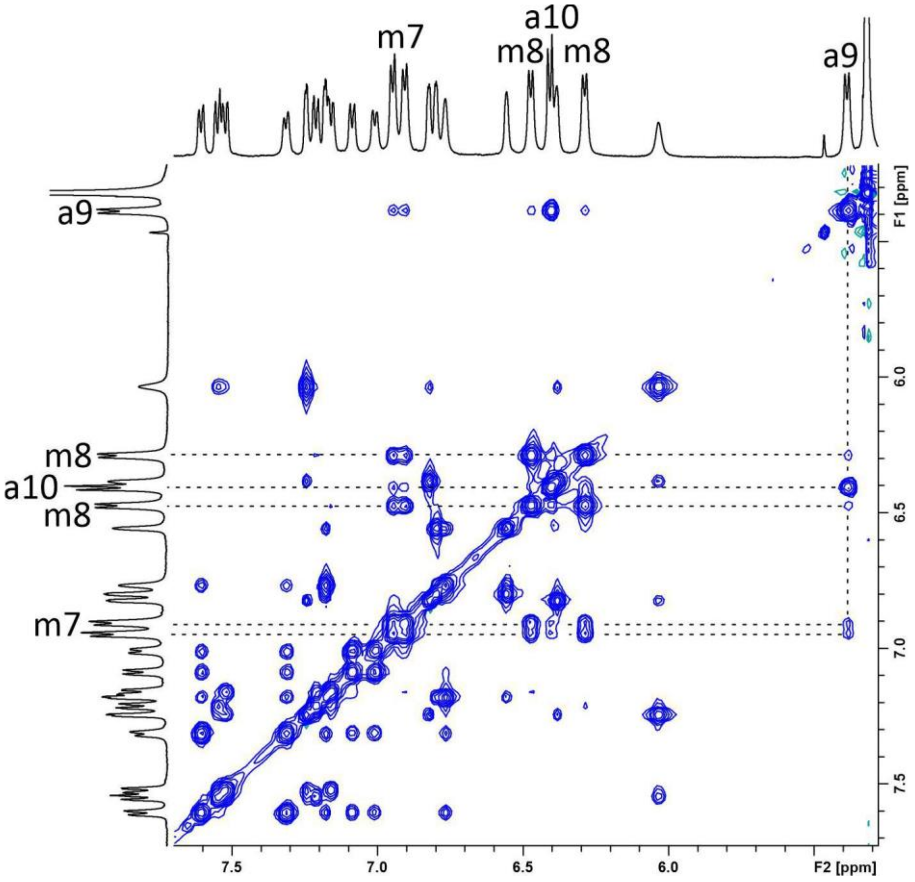

Figure S 165. The ¹H-¹H NOESY NMR spectrum of **[12-MeH$_3$]$^{4+}$** (600 MHz, [D$_2$]dichloromethane, 230 K). The NOE contacts between a9 protons of the axle and m7 and m8 of the macrocycle were marked with dashed lines.

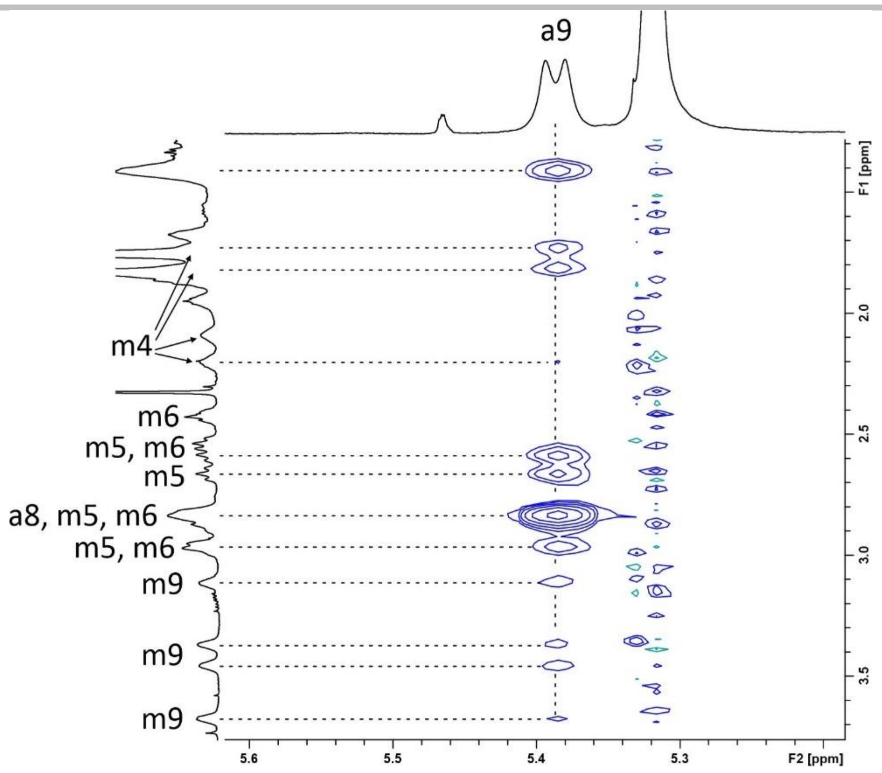

Figure S 166. The ^{1}H-^{1}H NOESY NMR spectrum of **[12-MeH$_3$]$^{4+}$** (600 MHz, [D$_2$]dichloromethane, 230 K). The NOE contacts between the a9 proton of the axle and m4, m5, m6, and m9 of the macrocycle were marked with dashed lines.

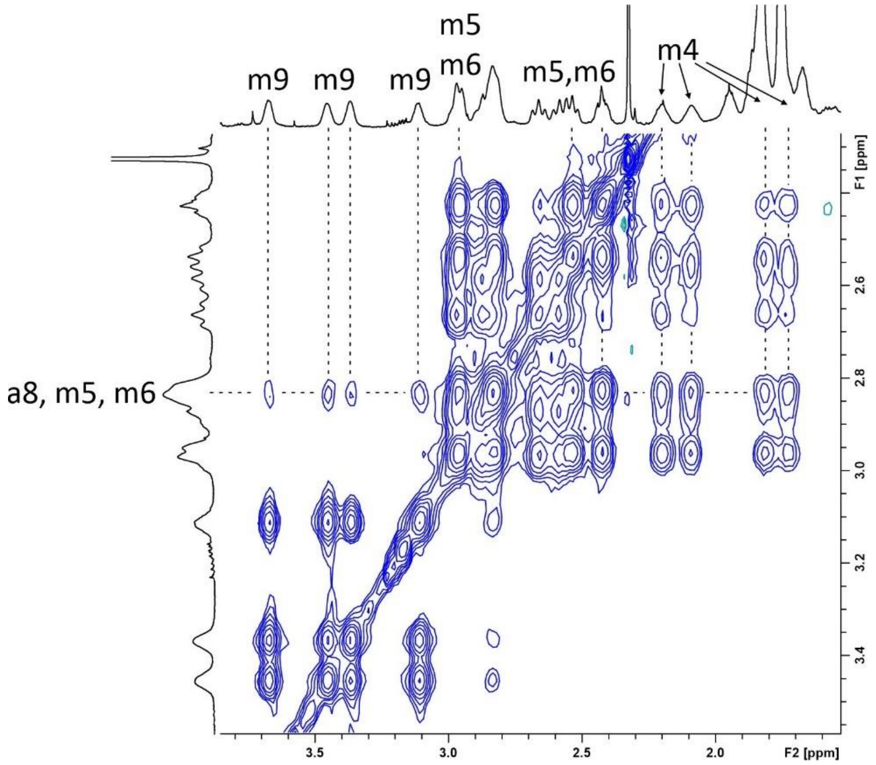

Figure S 167. The ^{1}H-^{1}H NOESY NMR spectrum of **[12-MeH$_3$]$^{4+}$** (600 MHz, [D$_2$]dichloromethane, 230 K). The NOE contacts between the a8 proton of the axle and m4, m5, m6, and m9 of the macrocycle were marked with dashed lines.

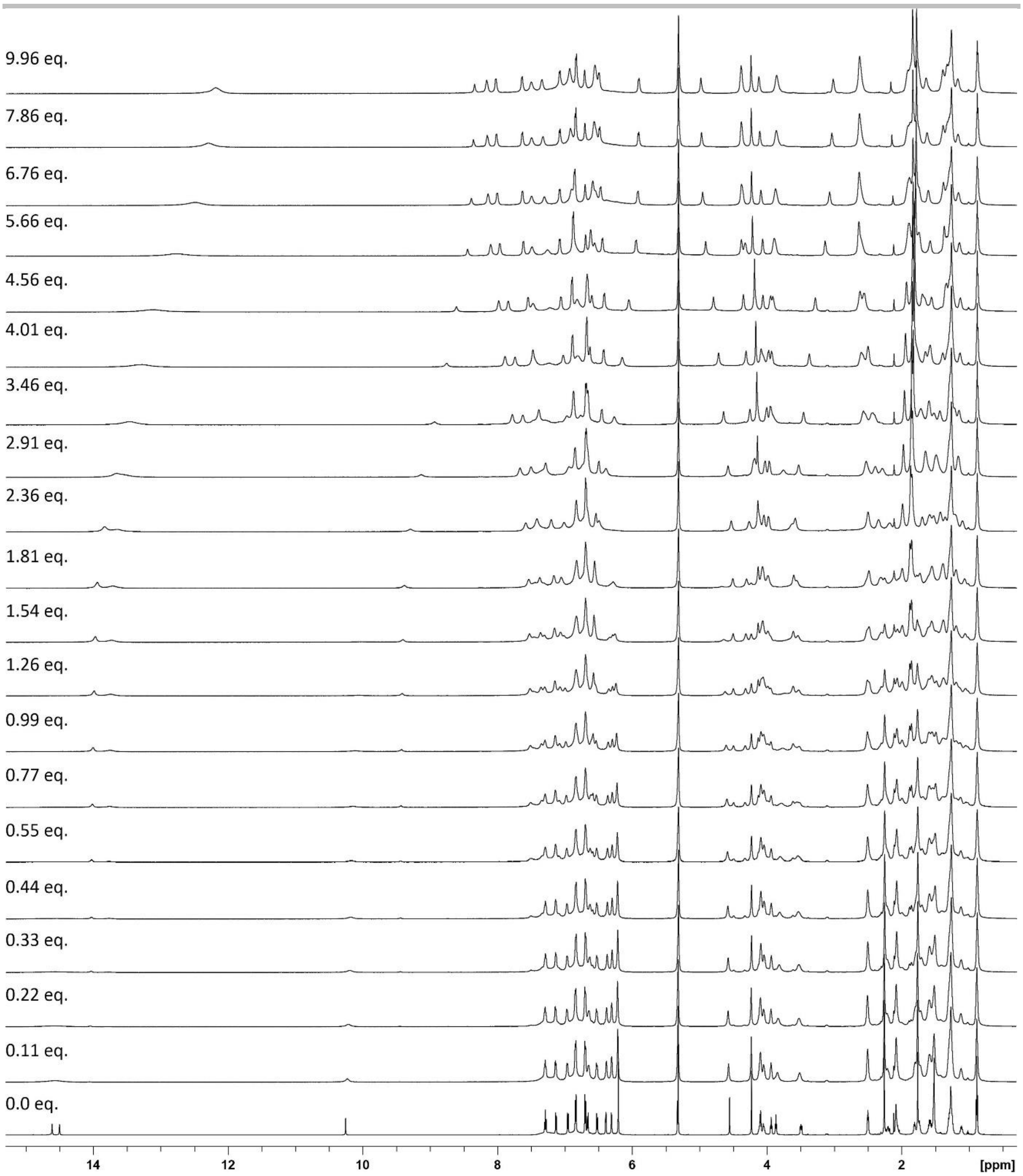

Figure S 168. The ^{1}H NMR spectra recorded during titration of **[12-Me]$^{+}$** with trifluoroacetic acid (600 MHz, [D_2]dichloromethane, 300 K).

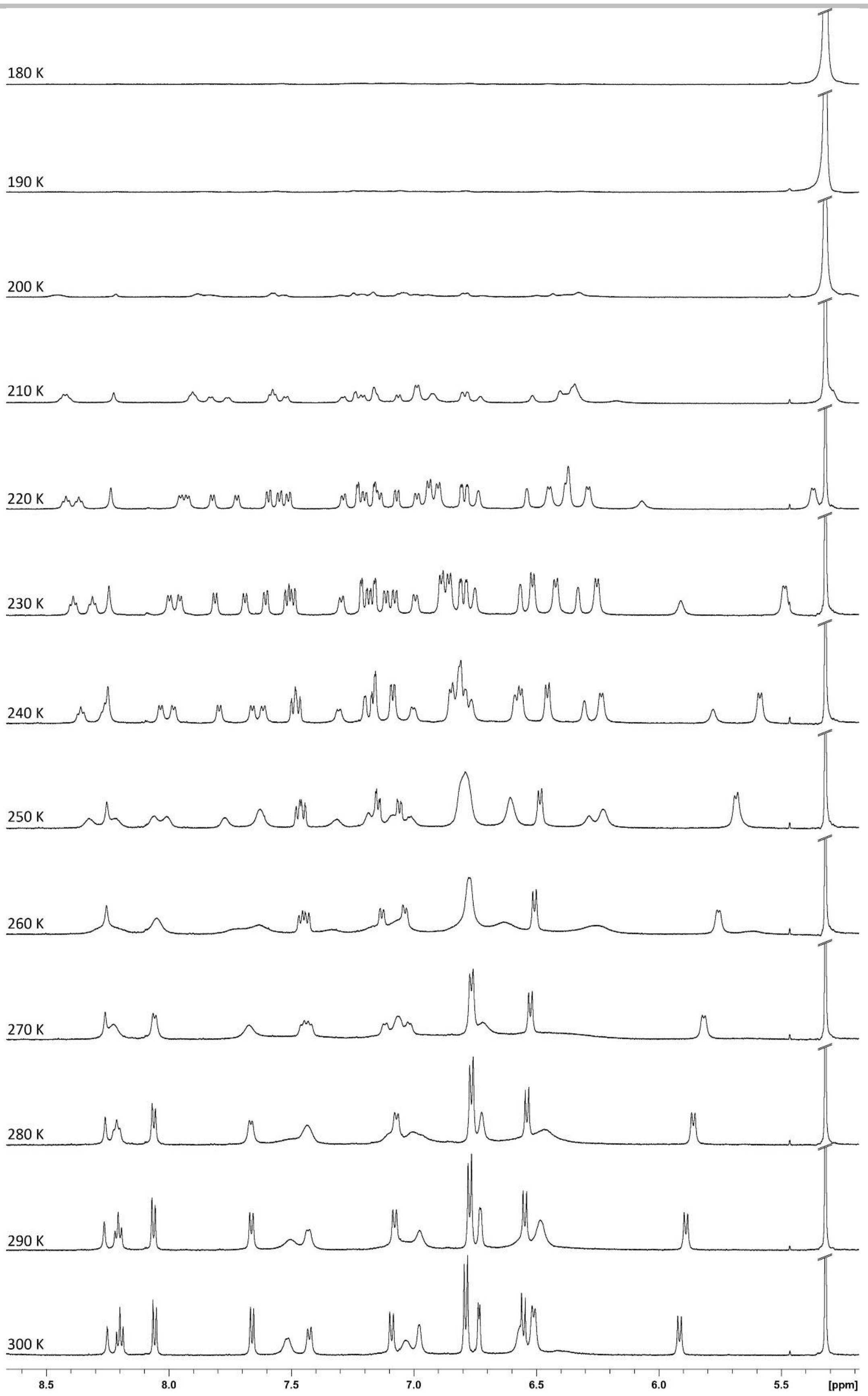

Figure S 169. The aromatic region of the ^{1}H NMR spectra of **[12-MeH$_3$]$^{4+}$** recorded in the 300–180 K temperature range (600 MHz, [D$_2$]dichloromethane).

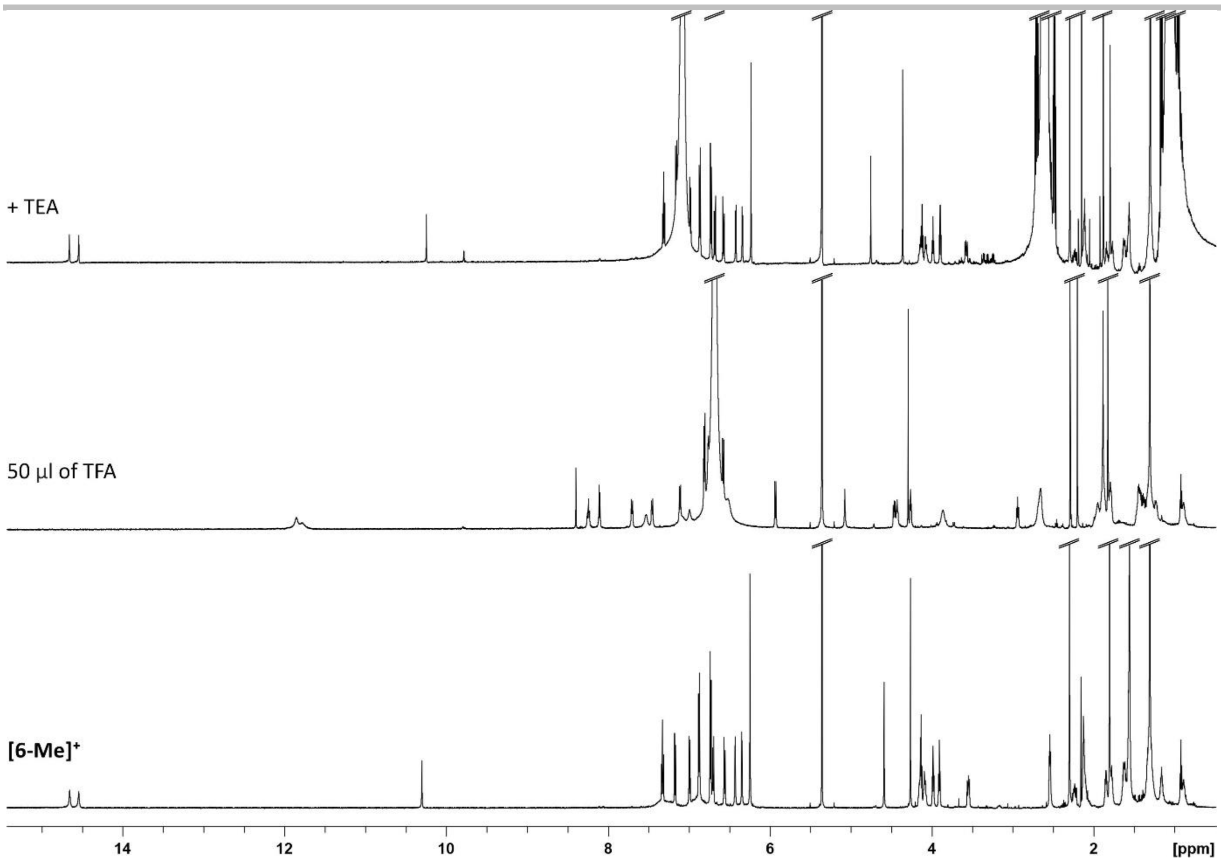

Figure S 170. The ^{1}H NMR spectra recorded upon the addition of 50 µl of trifluoroacetic acid to **[12-Me]+**, followed by the addition of TEA (600 MHz, [D$_2$]dichloromethane, 300 K).

Computational studies

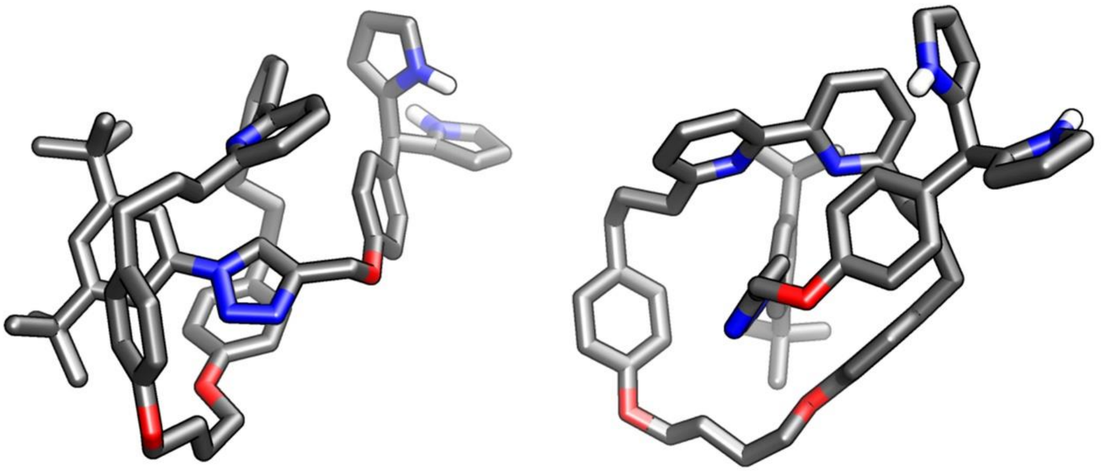

Figure S 171. DFT-optimized molecular geometry of **1**. Only selected protons were shown for clarity.

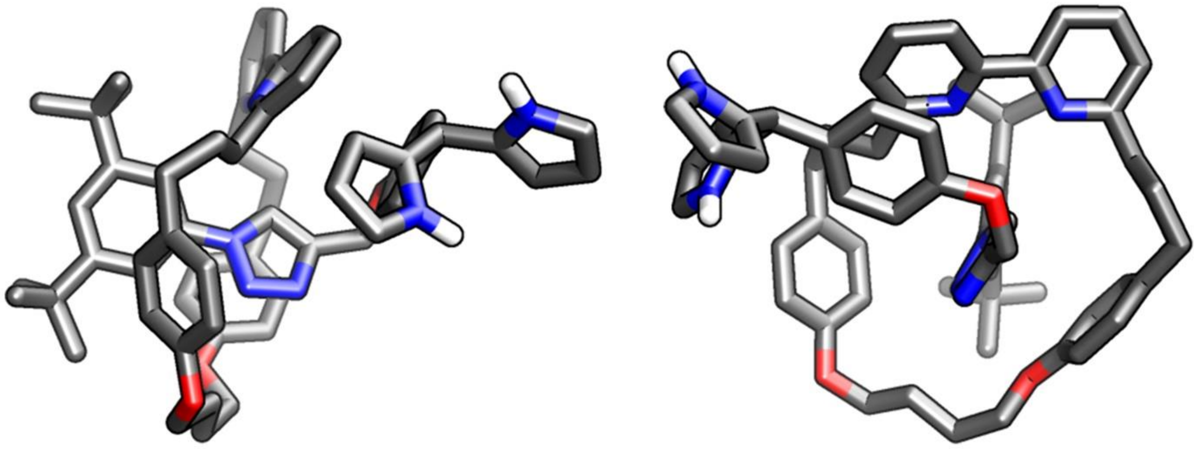

Figure S 172. DFT (PCM)-optimized molecular geometry of **1**. Only selected protons were shown for clarity.

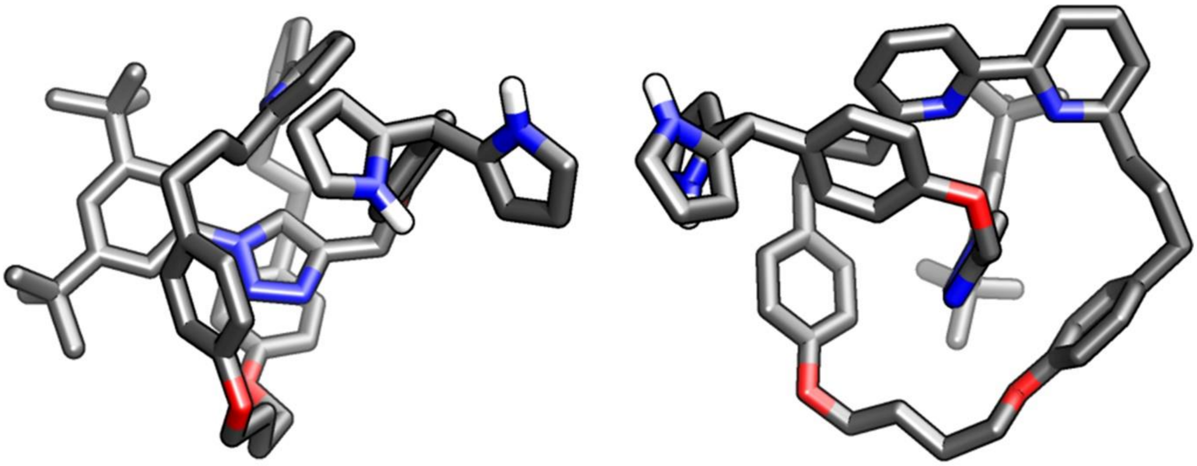

Figure S 173. GFN2-xTB-optimized molecular geometry of **1**. Only selected protons were shown for clarity.

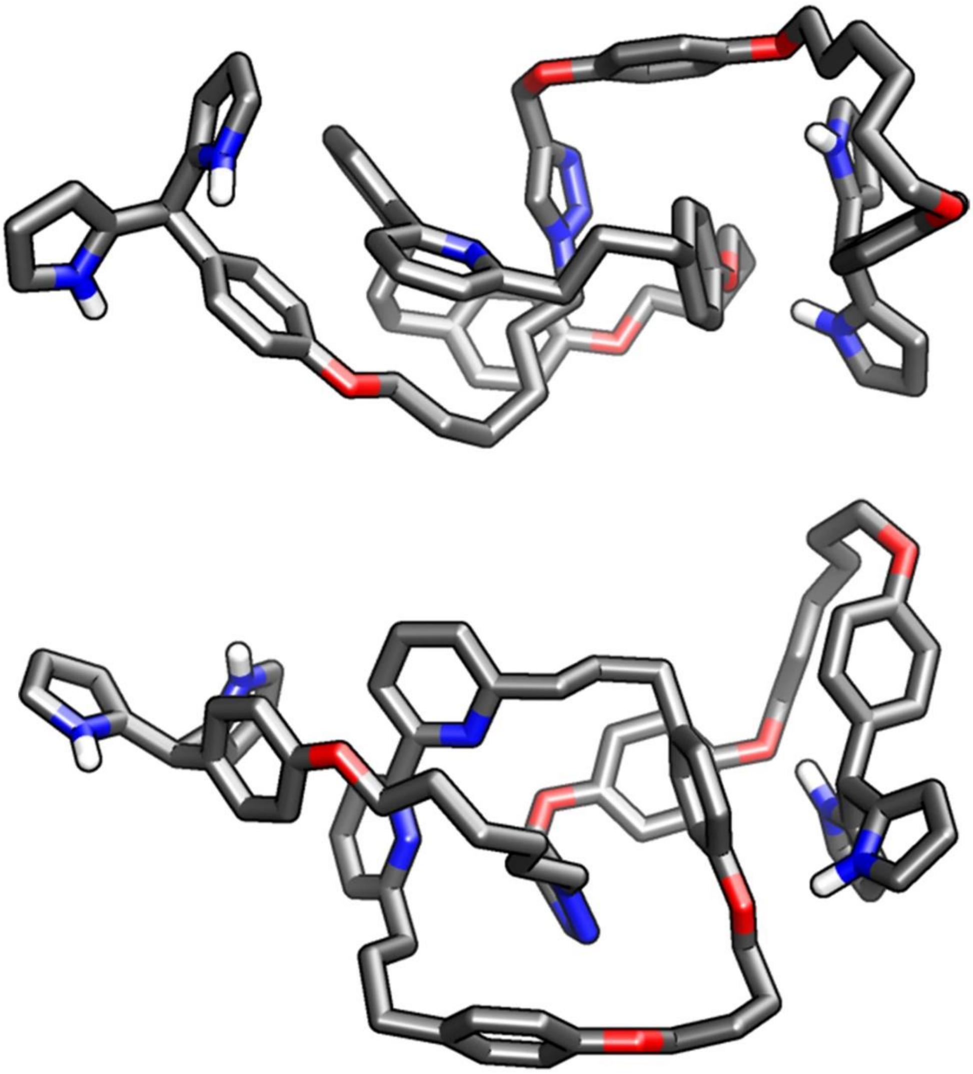

Figure S 174. DFT-optimized molecular geometry of **2**. Only selected protons were shown for clarity.

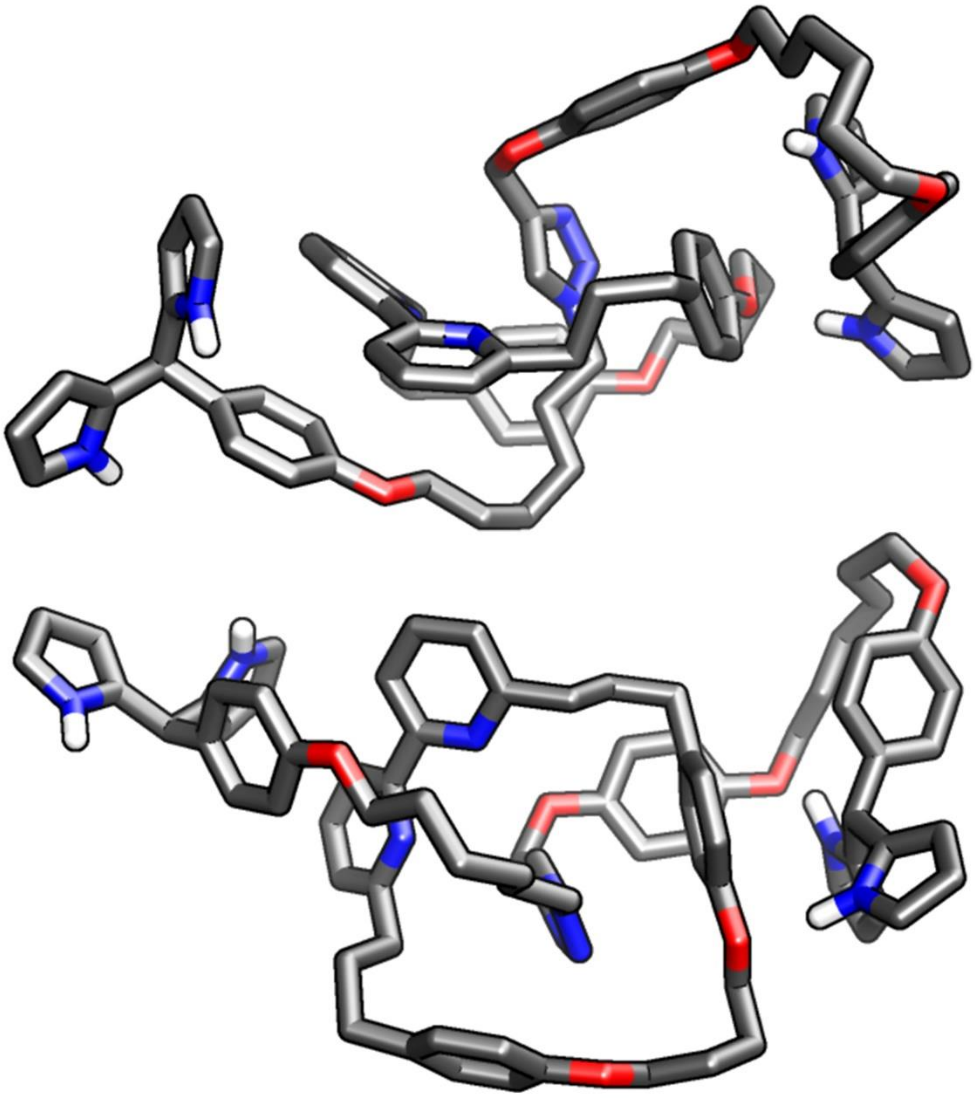

Figure S 175. DFT (PCM)-optimized molecular geometry of **2**. Only selected protons were shown for clarity.

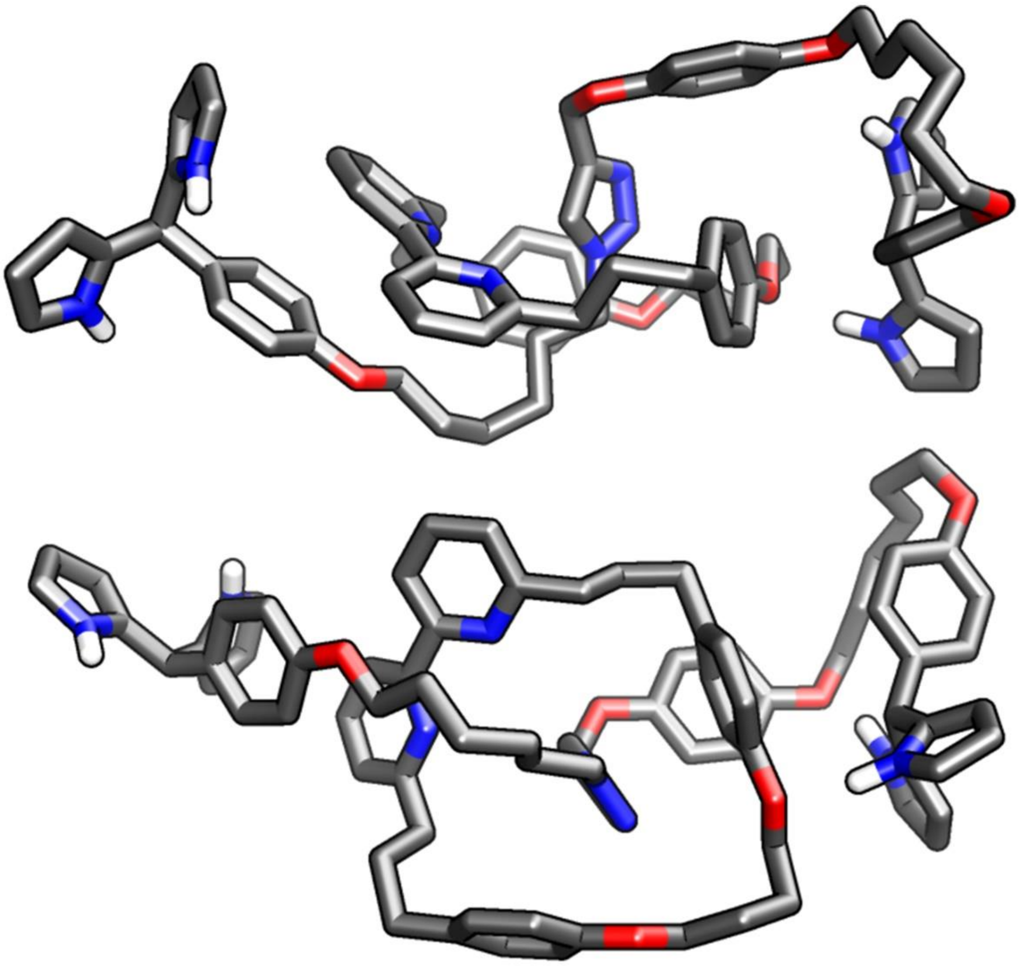

Figure S 176. GFN2-xTB-optimized molecular geometry of **2**. Only selected protons were shown for clarity.

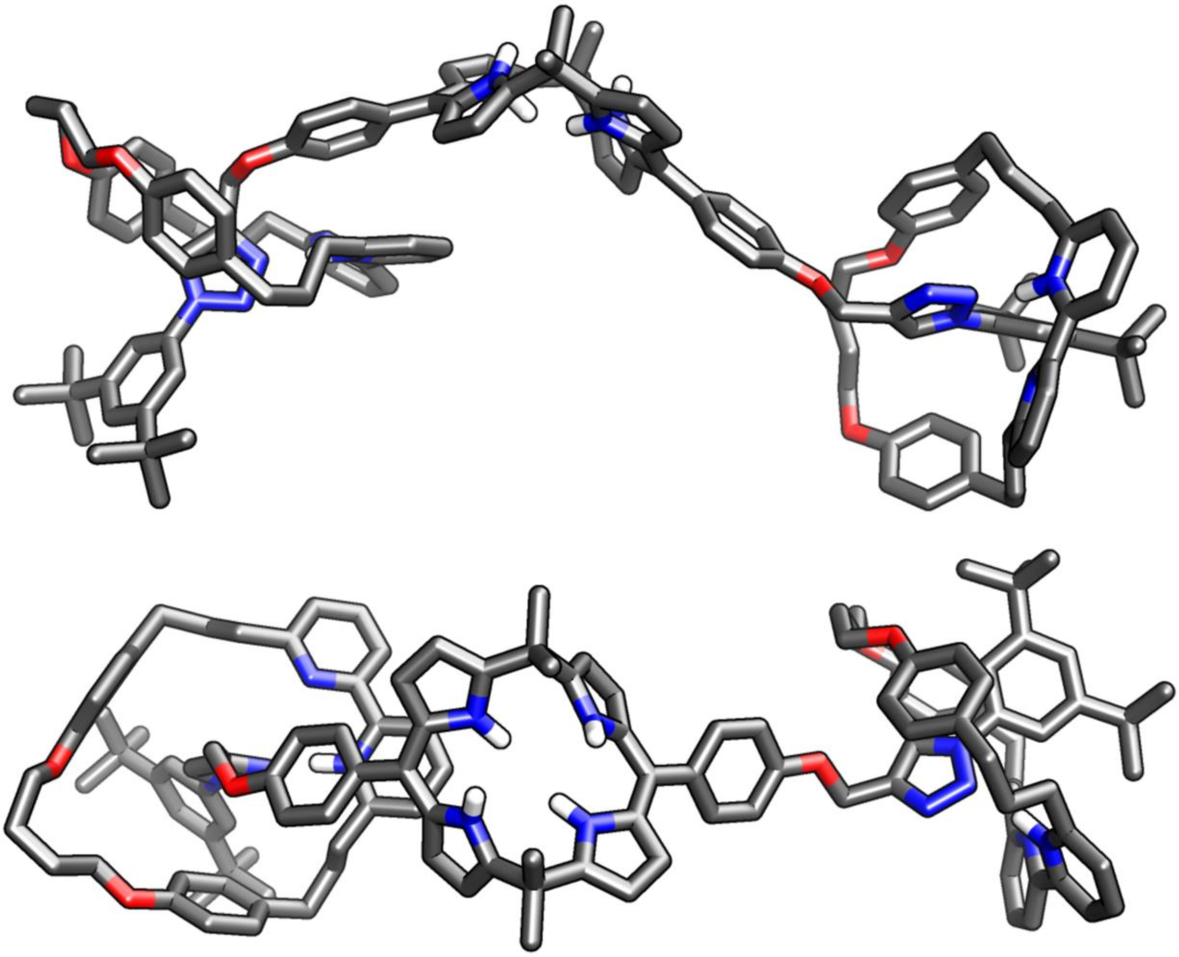

Figure S 177. DFT (PCM)-optimized molecular geometry of **[10-H$_4$]$^{4+}$**. Only selected protons were shown for clarity.

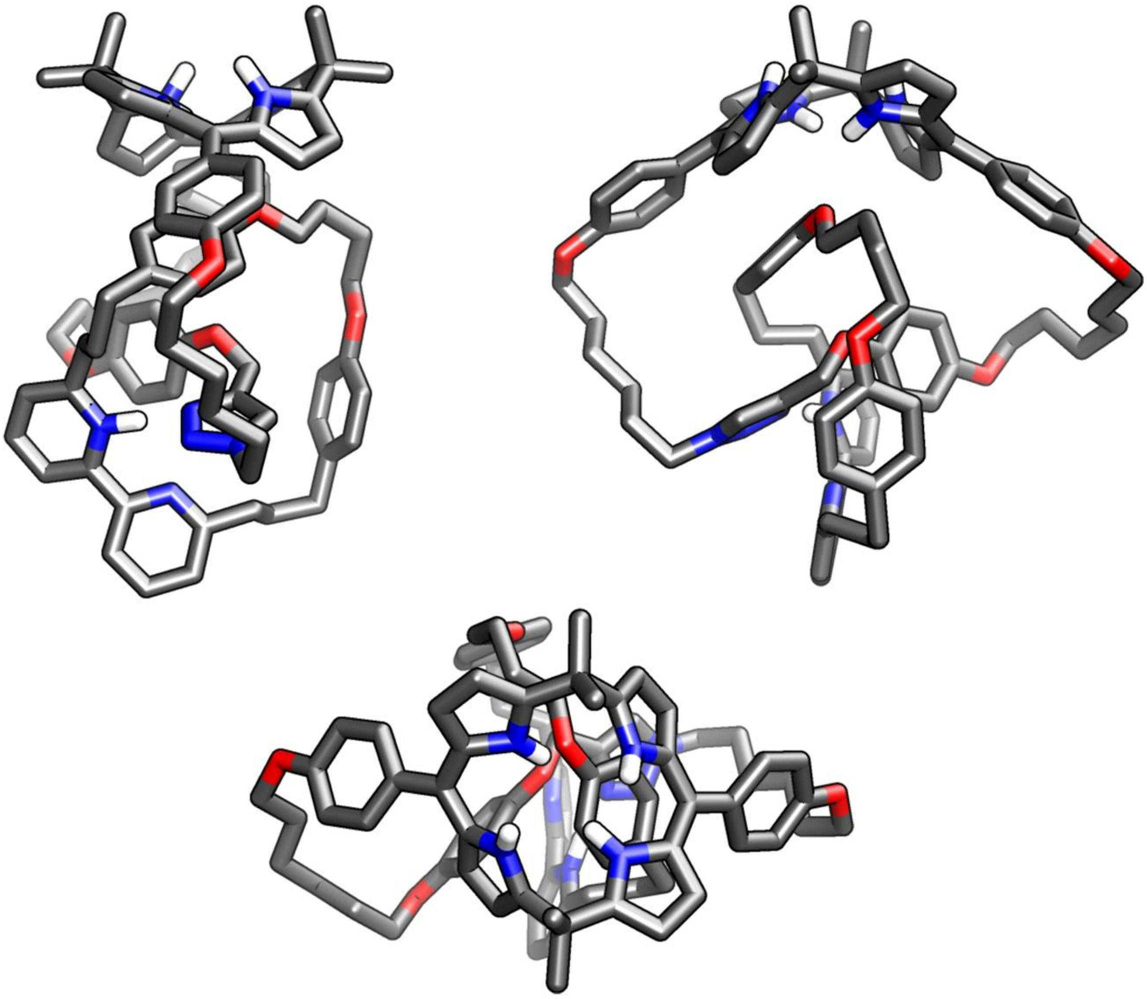

Figure S 178. GFN2-xTB-optimized molecular geometry of **[12-H$_3$]$^{3+}$**. Only selected protons were shown for clarity.

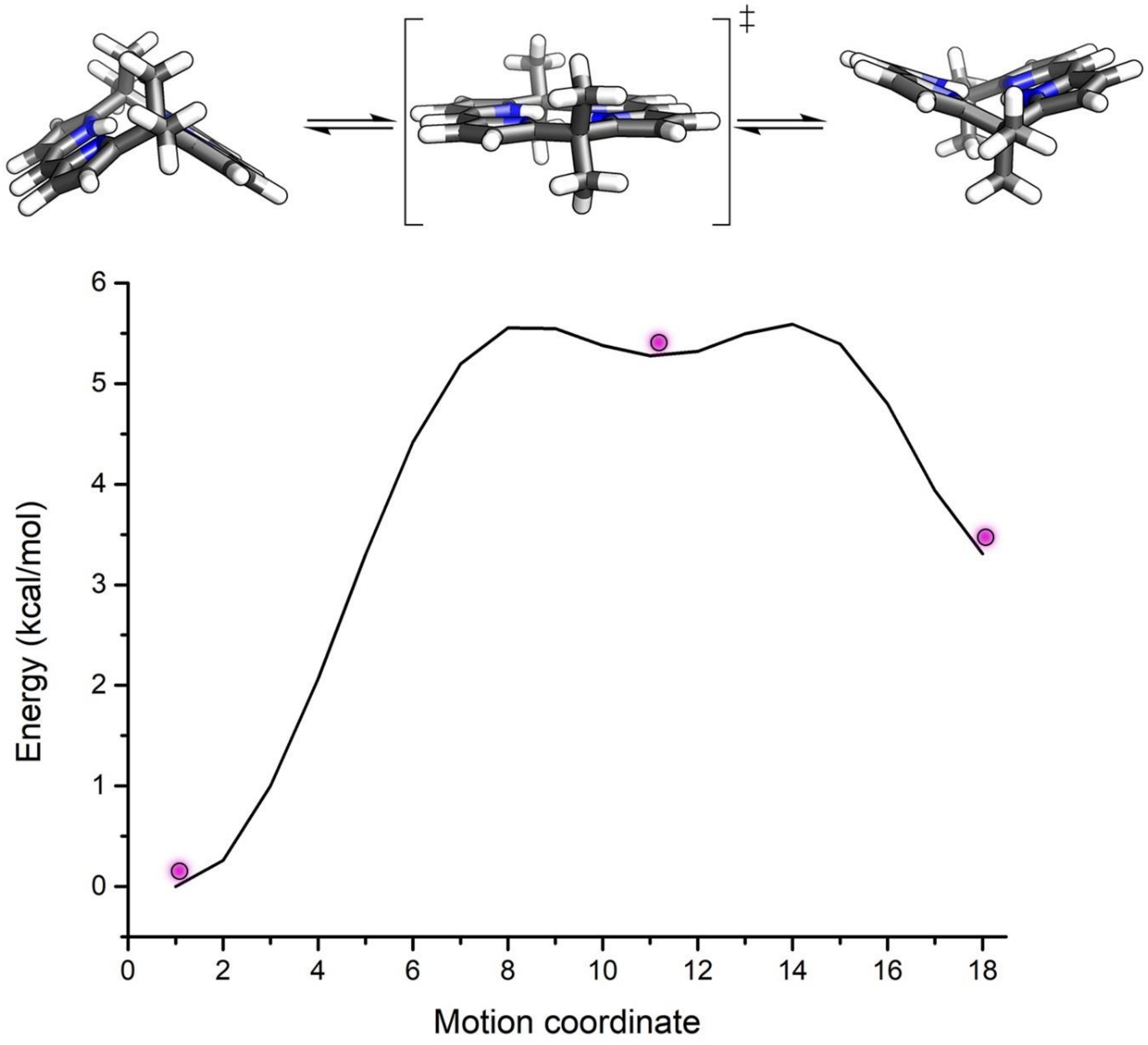

Figure S 179. Potential energy surface at the GFN2-xTB level of theory of the fluttering motion for calix[4]phyrin(**1**.1.**1**.1).

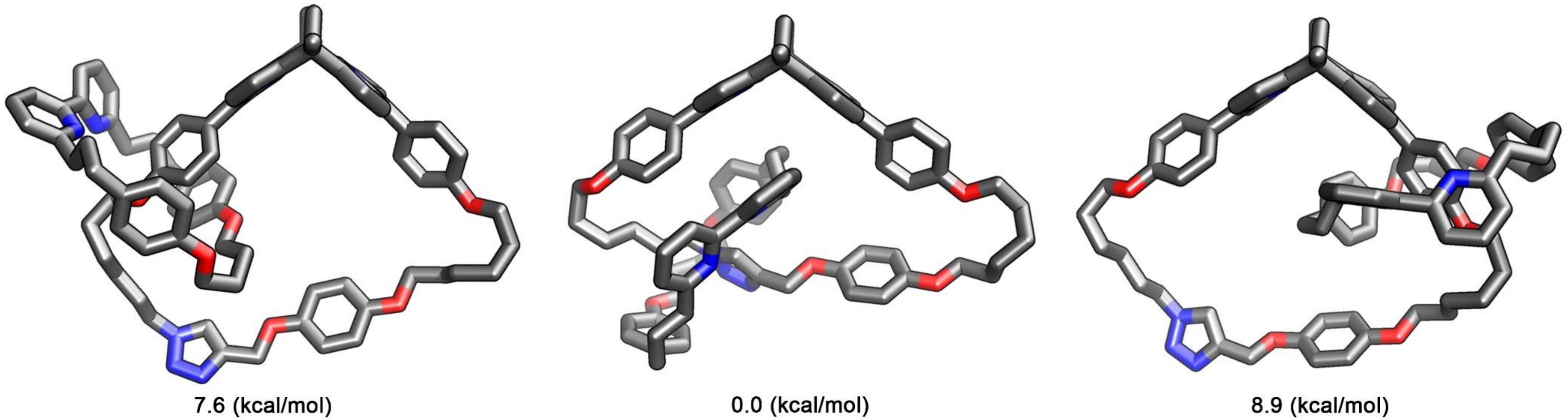

Figure S 180. GFN2-xTB-optimized molecular geometries and the relative energies for the possible co-conformers of **12**. Protons were omitted for clarity.

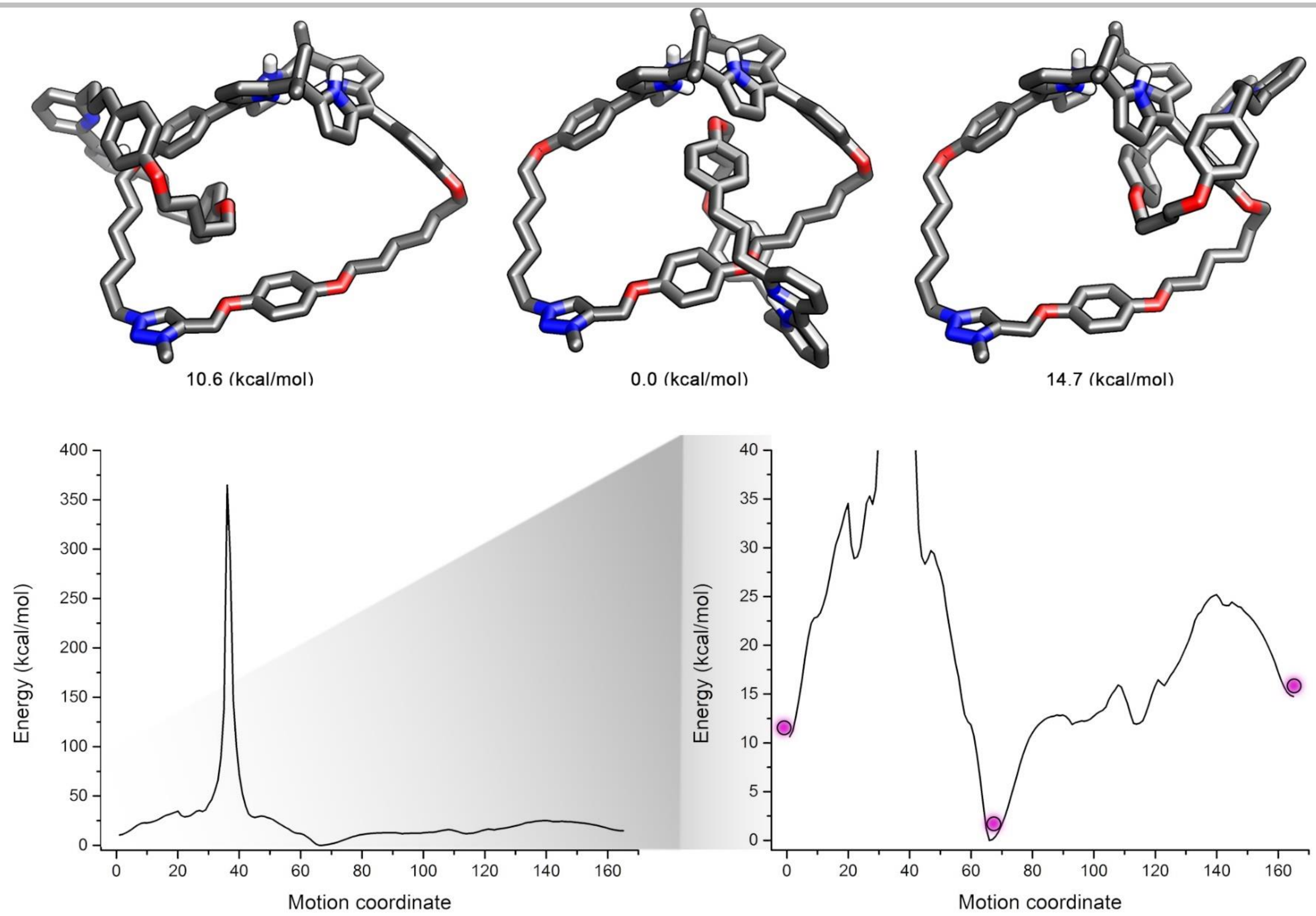

Figure S 181. GFN2-xTB-optimized molecular geometries and the relative energies for the possible co-conformers of **$[12\text{-MeH}_3]^{4+}$** and potential energy surface. Protons were omitted for clarit

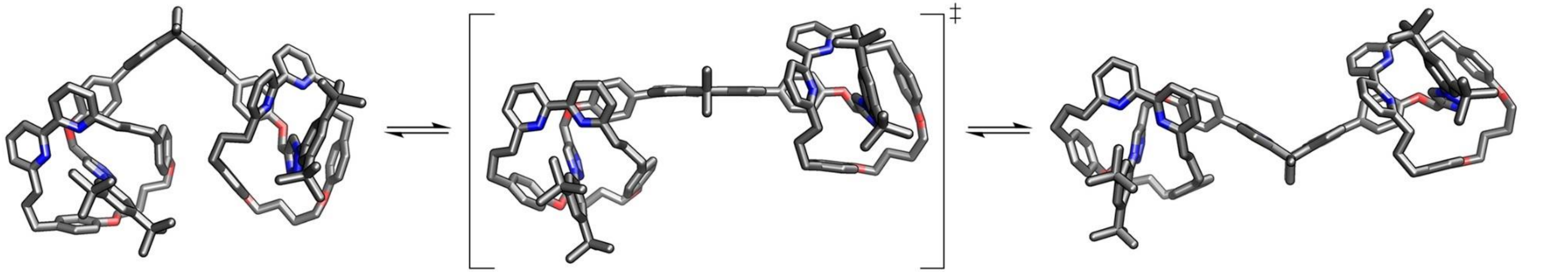

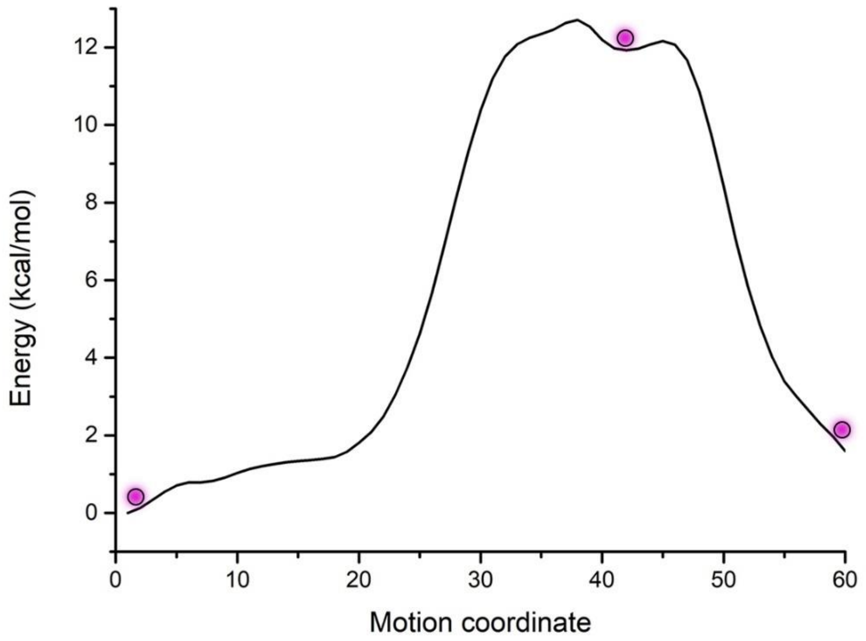

Figure S 182. Potential energy surface at the GFN2-xTB level of theory of the fluttering motion in **10**.

Table S 1. DFT-calculated Cartesian coordinates of **1.**

O	-0.43560	-1.33070	-4.14620
N	1.37540	0.38450	-0.55800
N	-0.15300	1.16890	-1.84470
N	0.74260	1.51610	-0.97050
N	2.33650	-8.20150	-6.47430
C	0.07070	-2.59500	-4.16360
C	2.38530	0.48480	0.44640
N	0.14240	-8.05980	-3.54810
C	0.85450	-0.70620	-1.17900
H	1.22050	-1.70470	-0.98350
C	-0.12960	-0.19110	-1.99970
C	0.71710	-2.95640	-5.35630
H	0.78120	-2.21870	-6.14980
C	3.10320	1.68220	0.53330
H	2.92240	2.44450	-0.20820
C	0.51290	-4.81380	-3.30740
H	0.45260	-5.51830	-2.48490
C	-0.01910	-3.53220	-3.12980
H	-0.47470	-3.28250	-2.17990
C	1.25340	-4.22580	-5.51010
H	1.75080	-4.48230	-6.43910
C	4.03500	1.85280	1.55170
C	1.15100	-5.18790	-4.49030
C	2.59650	-0.56080	1.33310
H	2.00090	-1.45990	1.24080
C	1.55170	-7.16070	-6.03040
C	-1.07670	-0.83470	-2.95930
H	-1.67150	-1.62480	-2.48920
H	-1.76240	-0.06190	-3.31260
C	4.21970	0.80000	2.46770
H	4.92960	0.93880	3.27240
C	3.52450	-0.40530	2.37710
C	1.80980	-6.55820	-4.66650
C	1.42700	-7.57190	-3.61990
C	4.85690	3.14390	1.70910
C	0.54080	-6.99070	-6.95790
H	-0.22520	-6.23120	-6.91050
C	3.69780	-1.54920	3.39110
C	2.16710	-8.23100	-2.66090
C	0.73140	-7.95910	-7.98850
H	0.13040	-8.08450	-8.87790
C	4.19030	-2.81390	2.65140
H	3.47880	-3.12910	1.88510
H	4.31610	-3.64440	3.35590
H	5.15440	-2.63060	2.16490
C	1.84270	-8.69920	-7.65740
C	4.70710	-1.20690	4.50210
H	4.78950	-2.04860	5.19760
H	4.39200	-0.33080	5.07910
H	5.70650	-1.01170	4.09940
C	2.33270	-1.85270	4.05330
H	1.59080	-2.17290	3.31760
H	1.94340	-0.96610	4.56650
H	2.43940	-2.65260	4.79500
C	1.29730	-9.14210	-1.98970
C	0.05280	-9.01700	-2.56680
C	4.49770	3.81040	3.05780
H	3.43340	4.06540	3.09440
H	5.07470	4.73190	3.19560
H	4.71230	3.15170	3.90490
C	6.36300	2.79600	1.68470
H	6.63390	2.31830	0.73710
H	6.63860	2.11320	2.49340
H	6.96700	3.70360	1.79430
C	4.58640	4.15630	0.58050
H	5.22600	5.03490	0.71320
H	3.55060	4.50600	0.58490
H	4.78610	3.73820	-0.40910
H	3.07760	-8.61620	-5.93130
N	1.83370	-3.58770	-0.22860
O	3.16240	2.90480	-2.54480
O	-0.29120	5.81170	0.44720
N	-0.14600	-2.35080	1.30950
C	0.83010	-4.30170	0.30680
C	2.95170	-4.21220	-0.62150
C	-0.37940	-3.52280	0.70140
C	4.31170	-1.02770	-3.42590
C	3.98390	-3.36620	-1.32560
H	4.99520	-3.69690	-1.05710
H	3.87450	-2.32930	-0.99430
C	3.11070	-5.59490	-0.46330
H	4.02980	-6.07350	-0.78550
C	2.07130	-6.33860	0.08500
H	2.15900	-7.41370	0.19370
C	0.90160	-5.68720	0.47180
H	0.07170	-6.23220	0.90880
C	3.79750	-3.45160	-2.85540
H	2.75180	-3.24540	-3.09630
H	3.99300	-4.48170	-3.18240
C	4.64240	1.10410	-2.30010
H	5.20320	1.73240	-1.61730
C	5.03720	-0.20400	-2.55550
H	5.92540	-0.59440	-2.06440
C	3.17650	-0.48410	-4.03530
H	2.58420	-1.10320	-4.70250
C	4.68950	-2.47670	-3.64960
H	5.74170	-2.63580	-3.38240
H	4.59540	-2.72290	-4.71440
C	-1.67120	-3.97300	0.40480
H	-1.82310	-4.91950	-0.10210
C	2.76230	0.82580	-3.79080
H	1.86010	1.19190	-4.26410
C	-1.17280	-1.55210	1.63120
C	3.49260	1.62630	-2.90560
C	0.65010	3.93280	1.72310
H	1.64160	4.04430	1.30810
C	-1.67180	4.57160	1.87640
H	-2.47580	5.22930	1.56250
C	-0.85420	2.68370	3.18050
C	-1.88880	3.54490	2.78660
H	-2.88790	3.39870	3.19170
C	-0.39790	4.77040	1.32900
C	0.41330	2.90300	2.63460
H	1.23870	2.24740	2.89850
C	0.84980	4.73880	-1.44720
H	-0.15120	4.65610	-1.88740
H	1.02700	3.79040	-0.93830
C	-1.73770	0.29620	3.34510
H	-1.94250	-0.50440	4.06700
H	-2.70330	0.58970	2.91530
C	-2.49780	-1.92650	1.36200
H	-3.31530	-1.26390	1.62300
C	1.88980	4.92240	-2.56210
H	1.54490	5.67330	-3.28370
H	2.84360	5.28860	-2.16070
C	-2.74440	-3.15040	0.74750
H	-3.76210	-3.45670	0.52260
C	-1.13510	1.50810	4.09140
H	-1.82730	1.80810	4.88770
H	-0.20790	1.19050	4.58500
C	-0.80070	-0.22440	2.24700
H	-0.72770	0.52140	1.44500
H	0.21260	-0.32190	2.64400
C	0.83300	5.88200	-0.43680
H	1.77400	5.92270	0.13200
H	0.71790	6.84870	-0.93800
C	2.16810	3.61140	-3.29770
H	1.24800	3.01890	-3.36380
H	2.55050	3.78200	-4.31390
H	2.32960	-9.51760	-8.16610

H	-0.57790	-7.81830	-4.21150
H	2.89540	-6.41380	-4.55860
H	3.21390	-8.05770	-2.45550
H	-0.87720	-9.52690	-2.36490
H	1.55540	-9.81490	-1.18370

Table S 2. DFT (PCM)-calculated Cartesian coordinates of **1**.

O	-1.20230	-2.02760	-3.01380
N	1.46010	0.44550	-0.68470
N	0.24410	1.16040	-2.30080
N	0.93830	1.55380	-1.26910
N	-8.44930	-1.55480	-0.34460
C	-2.34310	-1.72800	-2.31560
C	2.33500	0.56590	0.43760
N	-6.36800	1.26090	0.31360
C	1.07660	-0.67500	-1.35080
H	1.33730	-1.67420	-1.02080
C	0.31060	-0.20130	-2.39280
C	-3.14200	-2.82740	-1.97250
H	-2.82270	-3.81880	-2.27730
C	3.08420	1.74000	0.56910
H	3.00080	2.49330	-0.20000
C	-3.90810	-0.27770	-1.18280
H	-4.18190	0.72480	-0.87190
C	-2.74010	-0.44300	-1.93210
H	-2.15120	0.42840	-2.18370
C	-4.30660	-2.63940	-1.24040
H	-4.91470	-3.50140	-0.97760
C	3.94480	1.88590	1.65360
C	-4.70190	-1.36350	-0.81440
C	2.43580	-0.47730	1.34760
H	1.81800	-1.35840	1.21360
C	-7.20310	-1.29460	-0.86820
C	-0.38010	-0.95220	-3.48760
H	-0.96320	-0.24940	-4.09570
H	0.34940	-1.44270	-4.13770
C	4.02220	0.83550	2.58910
H	4.68480	0.95410	3.43700
C	3.29080	-0.34670	2.45640
C	-5.98000	-1.20840	0.01260
C	-6.00170	0.04690	0.84770
C	4.81980	3.13710	1.84990
C	-7.36870	-1.05730	-2.21990
H	-6.56830	-0.83640	-2.91150
C	3.36310	-1.49730	3.47650
C	-5.62480	0.27430	2.15720
C	-8.75930	-1.18460	-2.51470
H	-9.23000	-1.07460	-3.48200
C	3.82010	-2.78840	2.75730
H	3.13040	-3.07030	1.95790
H	3.86900	-3.62120	3.46840
H	4.81460	-2.65810	2.31670
C	-9.40170	-1.49090	-1.33450
C	4.34390	-1.20460	4.62680
H	4.36500	-2.05500	5.31590
H	4.04330	-0.32120	5.20030
H	5.36400	-1.04660	4.26130
C	1.95800	-1.72820	4.08180
H	1.23530	-2.02270	3.31700
H	1.58850	-0.81860	4.56870
H	1.99640	-2.52430	4.83410
C	-5.76240	1.67190	2.41230
C	-6.22550	2.25570	1.25100
C	4.43930	3.82040	3.18400
H	3.38740	4.12490	3.17990
H	5.05260	4.71460	3.34180
H	4.59250	3.15440	4.03820
C	6.30740	2.71460	1.88730
H	6.59390	2.22570	0.94960
H	6.51510	2.02000	2.70640
H	6.94800	3.59310	2.02320
C	4.64460	4.16060	0.71370
H	5.29960	5.01980	0.88970
H	3.61910	4.53670	0.65860
H	4.89330	3.73970	-0.26400
H	-8.63590	-1.73190	0.63090
N	1.70400	-3.62410	-0.26870
O	3.53280	3.07220	-2.49860
O	-0.32820	5.75690	0.29190
N	-0.25660	-2.37220	1.23810
C	0.79990	-4.37060	0.39060
C	2.82010	-4.20040	-0.73800
C	-0.40950	-3.66540	0.90840
C	4.22880	-0.92130	-3.57290
C	3.76890	-3.31230	-1.50690
H	4.79820	-3.66610	-1.37210
H	3.71520	-2.30010	-1.09580
C	3.07750	-5.56640	-0.56110
H	3.99340	-6.00020	-0.94920
C	2.14740	-6.34270	0.12420
H	2.32650	-7.40090	0.28940
C	0.98870	-5.74200	0.61180
H	0.26280	-6.31620	1.17550
C	3.43600	-3.28890	-3.01290
H	2.40350	-2.94250	-3.14770
H	3.47690	-4.31560	-3.39840
C	4.67530	1.04470	-2.20260
H	5.18580	1.51580	-1.37010
C	4.90700	-0.28930	-2.51890
H	5.62050	-0.85580	-1.92600
C	3.34580	-0.14260	-4.32680
H	2.81720	-0.59550	-5.16270
C	4.39140	-2.40170	-3.84420
H	5.42400	-2.71120	-3.64020
H	4.20890	-2.60050	-4.90660
C	-1.63200	-4.33270	1.05090
H	-1.73760	-5.36920	0.75170
C	3.10470	1.20160	-4.03130
H	2.40330	1.76030	-4.63750
C	-1.30080	-1.68870	1.73200
C	3.74670	1.79440	-2.93840
C	0.60650	3.98310	1.71440
H	1.62840	4.18050	1.42070
C	-1.76690	4.40620	1.55920
H	-2.58320	4.97510	1.12490
C	-0.95330	2.64030	3.02660
C	-2.00650	3.38510	2.47110
H	-3.03300	3.14540	2.74020
C	-0.45420	4.71650	1.17480
C	0.34760	2.95690	2.62720
H	1.18670	2.38540	3.01700
C	1.15680	4.85440	-1.44240
H	0.24260	4.74160	-2.03870
H	1.31220	3.89470	-0.94750
C	-1.88240	0.27530	3.28650
H	-2.00860	-0.51700	4.03540
H	-2.88800	0.54040	2.94000
C	-2.55830	-2.28550	1.89600
H	-3.39300	-1.70480	2.26810
C	2.33710	5.14810	-2.37830
H	2.09750	5.99840	-3.02830
H	3.23320	5.42790	-1.81070
C	-2.72040	-3.62190	1.54910
H	-3.68940	-4.10160	1.64930
C	-1.23540	1.49510	3.97470
H	-1.89780	1.83250	4.78220
H	-0.29840	1.17640	4.44800
C	-1.03740	-0.24970	2.11350
H	-1.18850	0.38610	1.23070

H	0.02480	-0.16180	2.35460
C	0.91080	5.94910	-0.40930
H	1.74800	6.03380	0.29720
H	0.79540	6.92360	-0.89370
C	2.67830	3.94370	-3.25470
H	1.75790	3.41890	-3.53490
H	3.20150	4.24110	-4.17320
H	-10.44310	-1.67800	-1.11850
H	-6.71660	1.37690	-0.62740
H	-6.00790	-2.04690	0.72060
H	-5.29620	-0.48470	2.85360
H	-6.47690	3.27980	1.01970
H	-5.56030	2.18520	3.34230

Table S 3. GFN2-xTB-calculated Cartesian coordinates of **1**.

O	-1.20610741868992	-1.90818131999874	-2.73502893443089
N	1.66622239004614	0.39585343221086	-0.47199420251952
N	0.44413097780412	1.17488353911599	-2.02846554356622
N	1.19721281610341	1.50120000049595	-1.04154706464081
N	-8.39055178132727	-0.80863823200767	-0.56640924545153
C	-2.33227035127183	-1.59720134159902	-2.01772787862169
C	2.50472357132210	0.46003017580038	0.65086863495172
N	-6.06099554190249	1.34021192577407	1.08408894184269
C	1.20040353581317	-0.69269313544036	-1.12410819305341
H	1.42088296131326	-1.70308838462555	-0.84619132631777
C	0.41239950052536	-0.17381181062202	-2.12951403951015
C	-3.24997510785568	-2.63375928301003	-1.85436643620219
H	-3.02391319401131	-3.59298708235822	-2.29398408956174
C	3.13053581155505	1.66790408663114	0.93823778213578
H	2.95159566772196	2.49065386835232	0.26924156439703
C	-3.78112194326685	-0.16589054521292	-0.74719171573508
H	-3.96287638937839	0.80414099992418	-0.31153788039410
C	-2.61059164760253	-0.35567380136900	-1.46263152354196
H	-1.92716748697561	0.47069213583294	-1.57700207711497
C	-4.41792293767169	-2.42592904714817	-1.15237552420754
H	-5.12365116134349	-3.23757788861771	-1.05115520476446
C	3.94511841892311	1.77357994586037	2.05290838586988
C	-4.70017385998301	-1.18948981526189	-0.58227862553000
C	2.67412451189161	-0.65086257285326	1.45910782108880
H	2.15370403219902	-1.56282712797858	1.21833748076364
C	-7.07518735876998	-0.55026189053979	-0.80402175300043
C	-0.37627280049167	-0.85294911708205	-3.19694781711960
H	-0.97181953679174	-0.10036446835494	-3.72891400126218
H	0.30096591988904	-1.33962432135263	-3.90641489514072
C	4.10465450033165	0.64755450824561	2.86131195205315
H	4.73398917808733	0.72973836732680	3.73018226452518
C	3.48146291667937	-0.56173514341016	2.58992644579228
C	-6.00720997092004	-0.98611927672008	0.15851088355947
C	-5.89674631394829	0.00423111726283	1.28176800450670
C	4.66250762496257	3.06375138729498	2.42438654115504
C	-6.99742027518610	0.17299373014659	-1.96835084067431
H	-6.09811055976571	0.52386964644304	-2.43670929485679
C	3.63678139863508	-1.78961065572670	3.47591795105844
C	-5.64854881726576	-0.19020911036553	2.61815317880057
C	-8.31348429211088	0.34645606421360	-2.44540850168158
H	-8.60064381661461	0.86415776814458	-3.33998980336040
C	4.27612245880556	-2.91366945616392	2.64888658585715
H	3.65357705851850	-3.17061467993788	1.79752496399907
H	4.40460631969629	-3.80457911022651	3.26057066518175
H	5.25216560345661	-2.60313799845679	2.28136075023817
C	-9.15011648930292	-0.26936716672123	-1.55567167546682
C	4.51697526826249	-1.53387499366117	4.70338869236527
H	4.57741719706804	-2.44280480062300	5.29893526195229
H	4.09724177501071	-0.75065850541007	5.33067566126384
H	5.52794764387715	-1.25821305217375	4.41181966517868
C	2.24921061917295	-2.23263168182928	3.96259346282003
H	1.59042774884565	-2.45880592219205	3.12869100405011
H	1.79028027775201	-1.44639154514425	4.55876877246432
H	2.33728026916850	-3.12420995068058	4.58058193198419
C	-5.66332797957241	1.07602445599227	3.23881262832310
C	-5.92365320989098	1.99937221043899	2.26264983689555
C	4.20531037582431	3.51080915746910	3.82119513123449
H	3.12911953874592	3.67256752192610	3.83112967794045
H	4.69502932096500	4.44367744084593	4.09284705084523
H	4.44699041248608	2.76865098556960	4.57706629111237
C	6.17762952292848	2.81184742979165	2.42917129891884
H	6.50719546903464	2.47673548445613	1.44767788758073

H	6.45253717429867	2.05446448082498	3.15812045918192
H	6.70806429165092	3.72935041209767	2.67562266312121
C	4.36666161444938	4.20146494042581	1.44306408758372
H	4.91263071757675	5.09309294108681	1.74589040762120
H	3.30666534977974	4.44188235216484	1.44032211967521
H	4.67713255992396	3.94469460080912	0.43305832506923
H	-8.74215183050208	-1.31148022839057	0.23163693498912
N	1.88759177580860	-3.64544107682342	-0.20716547662879
O	3.08804846992581	2.97305508902243	-2.99201865877933
O	-1.08666762829283	5.43889204928287	-0.65229626116742
N	-0.11765495515552	-2.42091570983177	1.17110620215402
C	0.95072416983427	-4.39571378518177	0.36947727922823
C	3.01152602938830	-4.20103531011000	-0.63704366442828
C	-0.26845085260455	-3.69328598490203	0.81070690631144
C	4.34385571326115	-0.97715300444559	-3.42473022624680
C	3.99541696112311	-3.29037130143614	-1.30769370416152
H	5.01053270866021	-3.57642835411382	-1.02634042203256
H	3.80973481695895	-2.26595729863206	-0.98600072302542
C	3.25509867853030	-5.56587714135656	-0.49866510348497
H	4.18062813922902	-5.99092369565885	-0.85432322896500
C	2.29686354739668	-6.35266821956007	0.11577878899518
H	2.46195097600433	-7.41166691506665	0.25340571161293
C	1.12325441953807	-5.76567988155481	0.55907648800078
H	0.36962421929111	-6.34986193325078	1.06197243995651
C	3.84997399551885	-3.37487769786419	-2.83116754729013
H	2.81796793037219	-3.14537136395514	-3.10593477966196
H	4.06594222012666	-4.39536480660462	-3.15621752279854
C	4.35054159555968	1.14189682916923	-2.27607033757718
H	4.69478002601512	1.77212928043234	-1.47199304674462
C	4.78751905582003	-0.16077428254574	-2.38968165565493
H	5.49256875545219	-0.54310601672762	-1.66612119355583
C	3.45313022221505	-0.43682941914310	-4.34056205035981
H	3.09890329299136	-1.04569782632124	-5.16086261811286
C	4.79589272313230	-2.40474389756951	-3.55315566936869
H	5.80180928359001	-2.51486655916251	-3.14379488708767
H	4.82705531286451	-2.67407500901736	-4.61105176568844
C	-1.50756522410802	-4.32814857400466	0.84068801648076
H	-1.61235176695495	-5.35272024465992	0.52166507744950
C	3.01329634964292	0.87270667829974	-4.24616106449295
H	2.33312121008646	1.25234945111614	-4.99185070912797
C	-1.16885764636300	-1.71548947503791	1.56845535706812
C	3.45196442233830	1.67698638479801	-3.20048787854763
C	0.11224154290180	3.94382688635055	0.87770553390441
H	1.08240635422931	4.20499670930252	0.49119574195600
C	-2.27723512881134	4.13331034866002	0.88415162176580
H	-3.16867530256689	4.58138699540038	0.47160432145094
C	-1.19586863611098	2.64394483581148	2.44058532369260
C	-2.34828955398526	3.21776869118194	1.91323893800592
H	-3.31221449564611	2.93714188792936	2.31319692423161
C	-1.04281691684684	4.50518494292918	0.35047272848121
C	0.02591334874624	3.02353371514434	1.90776617235306
H	0.93669275874079	2.58954113799161	2.29438558477980
C	0.47897434085154	4.54354966225416	-2.30593406326489
H	-0.38771519379389	4.27797096943989	-2.91543553092700
H	0.72761846386594	3.67196344595694	-1.70008154790528
C	-1.80366500530536	0.27635032619282	3.02322997670084
H	-1.85768103467259	-0.42687545838323	3.85763283720688
H	-2.81073952375981	0.41512251889664	2.62781919335963
C	-2.44214524116945	-2.27649612681460	1.63166483992347
H	-3.28337491637376	-1.68544881924450	1.94994171631775
C	1.66097386306321	4.86956794566157	-3.21577370134259
H	1.36354641292532	5.58772054911206	-3.98077842282766
H	2.47639604550645	5.30335525854360	-2.63450170459975
C	-2.60586207814633	-3.59908711306810	1.26213472863429
H	-3.58170438059989	-4.06010091571852	1.29929917518817
C	-1.28258837828508	1.62318245262239	3.53978392635856
H	-1.95904402932351	1.98264687069908	4.31887006986915
H	-0.29506741877579	1.48106223009332	3.98314711369846
C	-0.88845609412425	-0.28958510778588	1.93956453013578
H	-0.98257214636584	0.32217798215147	1.03712054263993
H	0.15325842486740	-0.22802653600752	2.26034958872020

C	0.08767795186436	5.71179442184533	-1.40183378782276
H	0.91331251178984	5.99052061144327	-0.73617078689741
H	-0.18080636298872	6.58575733373416	-2.00275389157488
C	2.18012417982830	3.59850290249857	-3.88709809082514
H	1.33865281232342	2.93029802962921	-4.09782500220457
H	2.70616151897324	3.83046160683104	-4.82374039149387
H	-10.21799434808546	-0.36344421277134	-1.55838610076645
H	-6.27235644653888	1.75555147753972	0.19089214122988
H	-6.30323376349530	-1.94806793251859	0.59329474588300
H	-5.48171903433832	-1.13225411416745	3.10489808241844
H	-6.02629492337270	3.06496566352837	2.32535606879219
H	-5.50352322892959	1.27423235252331	4.28100152627135

Table S 4. DFT-calculated Cartesian coordinates of **2**.

N	2.17370	-1.09850	0.93900
O	-3.75300	3.05910	0.02240
C	3.41280	-1.40630	0.52240
C	1.67030	-1.73710	2.00500
C	2.40590	-2.69530	2.71360
C	3.69030	-3.01160	2.28340
C	4.20250	-2.36930	1.16090
C	0.23160	-1.44770	2.34850
C	-0.70940	-2.40200	1.58470
C	-2.20010	-2.10170	1.82720
C	-2.59200	-0.72690	1.33510
C	-2.79130	0.34410	2.21760
C	-3.15020	1.60380	1.75000
C	-3.31440	1.81350	0.37530
C	-3.08060	0.77350	-0.52520
C	-2.72400	-0.48150	-0.03480
C	-3.94110	3.38640	-1.36780
C	-4.34080	4.86130	-1.39810
N	3.67110	0.64530	-0.72590
O	-1.07360	6.22700	-0.16210
C	4.90720	0.75260	-2.77760
C	4.14500	1.34940	-1.76420
C	3.94170	-0.66790	-0.65880
C	4.69540	-1.33970	-1.62770
C	5.18150	-0.61030	-2.70700
C	3.84040	2.82780	-1.78580
C	4.65390	3.61950	-0.74240
C	4.47260	5.15270	-0.83800
C	3.03040	5.58040	-0.68620
C	2.17370	5.58270	-1.79040
C	0.80580	5.80650	-1.66200
C	0.26020	6.05200	-0.39670
C	1.10900	6.10930	0.71680
C	2.47290	5.87110	0.56730
C	-3.38230	5.76640	-0.60300
C	-1.97600	5.79460	-1.19230
H	1.96850	-3.17850	3.58170
H	4.28940	-3.74110	2.82110
H	5.20770	-2.56380	0.80980
H	0.00050	-0.41290	2.08090
H	0.06580	-1.55610	3.42780
H	-0.48770	-3.43710	1.87530
H	-0.49640	-2.32380	0.51110
H	-2.42550	-2.19610	2.89740
H	-2.80310	-2.86050	1.31290
H	-2.68530	0.18470	3.28770
H	-3.33950	2.42670	2.43030
H	-3.19440	0.91530	-1.59090
H	-2.56100	-1.28600	-0.74240
H	-4.73400	2.75690	-1.79080
H	-3.00500	3.19920	-1.90580
H	-5.35170	4.95790	-0.98500
H	-4.40220	5.17550	-2.44770
H	5.27220	1.35480	-3.60380
H	4.89460	-2.40250	-1.54920
H	5.77230	-1.10640	-3.46980
H	2.77130	2.96840	-1.60220
H	4.04710	3.22740	-2.78590
H	5.72030	3.38470	-0.85980
H	4.35970	3.28250	0.25760
H	4.86230	5.49330	-1.80600
H	5.09380	5.62510	-0.06750
H	2.57700	5.37190	-2.77730
H	0.17540	5.75520	-2.53960
H	0.67420	6.31300	1.69020
H	3.11310	5.88770	1.44680
H	-3.30900	5.39250	0.42180
H	-3.78690	6.78260	-0.54560
H	-1.92780	6.47890	-2.05190
H	-1.66420	4.80140	-1.53390
O	0.19480	-1.68440	-3.27710
N	0.13310	1.89570	-0.92440
N	-0.23590	1.84560	-3.03890
N	-0.39240	2.58230	-1.96830
C	0.69630	-0.40010	-3.67560
C	0.61950	0.70170	-1.32830
C	0.38850	0.68260	-2.68960
C	6.94780	-0.92220	2.67760
C	7.85050	-2.87110	-2.59950
C	6.97880	-3.99200	-2.73450
C	6.72990	-4.47160	-1.46700
N	7.41940	-3.67650	-0.58040
C	7.88370	-1.46910	1.80620
C	8.07210	-0.94450	0.51660
C	7.34250	0.19020	0.16320
C	6.41440	0.76210	1.03150
C	6.17450	0.17800	2.28060
C	8.93400	-1.66760	-0.52430
C	8.10830	-2.69460	-1.25670
C	10.17330	-2.28670	0.07030
C	10.72720	-3.54390	-0.05770
C	11.93200	-3.56720	0.70410
C	12.08010	-2.32460	1.27700
N	11.01280	-1.55270	0.88090
O	5.21550	0.59280	3.15160
C	4.30430	1.61290	2.71370
C	3.21940	1.73960	3.77540
C	2.29450	2.96050	3.57770
C	1.05810	2.74710	2.68880
C	1.33700	2.42970	1.21310
C	0.01480	2.39950	0.44280
O	-5.24680	-2.66610	-2.79420
C	-1.15940	-1.87180	-3.18760
C	-2.13370	-0.94100	-3.57420
C	-3.48330	-1.25480	-3.43590
C	-3.88630	-2.48730	-2.91560
C	-2.91610	-3.41870	-2.52990
C	-1.56350	-3.10560	-2.66820
C	-5.78320	-3.98150	-2.61110
C	-6.02300	-4.31080	-1.13910
C	-6.56510	-5.74340	-0.96890
C	-7.26650	-5.98390	0.37650
C	-6.35110	-5.83880	1.60200
C	-7.13360	-5.64670	2.90610

O	-8.09300	-4.58130	2.83160
C	-7.64820	-3.33960	2.44440
C	-6.39830	-2.81770	2.78810
C	-6.01730	-1.56950	2.29990
C	-6.85940	-0.81750	1.47640
C	-8.12550	-1.33390	1.17690
C	-8.51930	-2.58180	1.65430
C	-6.37440	0.51480	0.92130
C	-6.70630	0.70420	-0.53940
C	-6.83410	1.68370	1.75320
C	-7.81830	1.80220	2.71280
C	-7.81670	3.15180	3.17020
C	-6.82890	3.81670	2.47740
N	-6.24030	2.92000	1.61980
C	-7.29950	1.75540	-1.21290
C	-7.31900	1.42750	-2.60080
C	-6.74090	0.18390	-2.73410
N	-6.36850	-0.24050	-1.48120
H	1.77510	-0.56030	-3.75870
H	0.31510	-0.11280	-4.66410
H	1.07580	0.00350	-0.64010
H	6.77480	-1.34820	3.66070
H	8.25180	-2.25870	-3.39490
H	6.58240	-4.39960	-3.65370
H	6.13500	-5.30550	-1.12600
H	7.45570	-3.80200	0.41860
H	8.47520	-2.32430	2.12580
H	7.46270	0.61790	-0.82890
H	5.84950	1.62250	0.70760
H	9.23370	-0.92850	-1.28020
H	10.31310	-4.34700	-0.65010
H	12.61110	-4.40070	0.81570
H	12.84430	-1.93070	1.93020
H	10.81180	-0.61950	1.20350
H	3.89010	1.34560	1.73590
H	4.84610	2.56570	2.61000
H	3.72420	1.81490	4.74550
H	2.63390	0.81170	3.79930
H	2.87870	3.80510	3.18470
H	1.93940	3.28180	4.56400
H	0.44310	3.65580	2.74330
H	0.44520	1.94100	3.11860
H	1.83800	1.46450	1.10960
H	1.99490	3.18850	0.77930
H	-0.70270	1.76050	0.96070
H	-0.42440	3.39630	0.36510
H	-1.85780	0.03360	-3.95650
H	-4.24250	-0.53230	-3.71260
H	-3.19800	-4.37640	-2.10720
H	-0.79810	-3.81320	-2.36640
H	-5.13610	-4.72620	-3.09230
H	-6.73620	-3.97990	-3.15100
H	-6.73700	-3.58490	-0.72910
H	-5.08950	-4.17370	-0.58160
H	-5.74770	-6.46450	-1.11010
H	-7.28800	-5.94770	-1.77100
H	-7.72440	-6.98150	0.38080
H	-8.09150	-5.26940	0.46640
H	-5.68090	-4.98480	1.46800
H	-5.70780	-6.72160	1.71250
H	-6.44750	-5.49450	3.75120
H	-7.73880	-6.53020	3.13080
H	-5.71730	-3.38540	3.41330
H	-5.03850	-1.17550	2.55380
H	-8.79270	-0.76140	0.53870
H	-9.48890	-3.00170	1.40600
H	-5.28510	0.47640	0.99580
H	-8.45930	0.99930	3.04640
H	-8.46310	3.58140	3.92280
H	-6.49430	4.84250	2.52410
H	-5.46290	3.12090	1.00170
H	-7.67950	2.65080	-0.74480
H	-7.72680	2.02700	-3.40270
H	-6.58930	-0.45080	-3.59290
H	-5.92080	-1.12760	-1.30530

Table S 5. DFT (PCM)-calculated Cartesian coordinates of **2**.

N	2.14370	-1.14270	0.92900
O	-3.77140	3.16490	0.20630
C	3.38610	-1.46790	0.53340
C	1.60420	-1.78230	1.97760
C	2.30760	-2.75720	2.69530
C	3.60010	-3.08400	2.29620
C	4.14790	-2.44200	1.18940
C	0.15730	-1.48230	2.27450
C	-0.75530	-2.38720	1.42030
C	-2.25670	-2.12680	1.64120
C	-2.65110	-0.72140	1.24930
C	-2.76150	0.30720	2.19600
C	-3.10270	1.60050	1.81250
C	-3.33930	1.88970	0.46210
C	-3.19650	0.89130	-0.50240
C	-2.85930	-0.39910	-0.09470
C	-3.96750	3.59020	-1.15650
C	-4.27950	5.08390	-1.09430
N	3.62160	0.54390	-0.79180
O	-0.86340	6.36200	-0.16690
C	4.90840	0.60390	-2.81610
C	4.09970	1.21680	-1.84880
C	3.92990	-0.75750	-0.65890
C	4.72320	-1.44500	-1.58480
C	5.21710	-0.74620	-2.68190
C	3.76770	2.68670	-1.93790
C	4.72090	3.55150	-1.08560
C	4.55980	5.07080	-1.32230
C	3.15090	5.55210	-1.05680
C	2.19330	5.54920	-2.07550
C	0.85140	5.83500	-1.82750
C	0.43870	6.14280	-0.52600
C	1.39230	6.20870	0.49890
C	2.72650	5.91090	0.23160
C	-3.20890	5.90200	-0.35060
C	-1.87720	5.92410	-1.09010
H	1.84010	-3.24630	3.54390
H	4.17620	-3.82650	2.84050
H	5.15700	-2.65680	0.86310
H	-0.04870	-0.43140	2.05130
H	-0.05790	-1.64510	3.33740
H	-0.52840	-3.43790	1.64110
H	-0.51470	-2.22990	0.36070
H	-2.51010	-2.31180	2.69270
H	-2.83050	-2.84780	1.04650
H	-2.59530	0.08940	3.24760
H	-3.21800	2.39260	2.54480
H	-3.37660	1.08940	-1.55080
H	-2.77620	-1.17550	-0.84470
H	-4.80290	3.03270	-1.59710
H	-3.05710	3.37950	-1.72750
H	-5.24480	5.21660	-0.59250
H	-4.40670	5.44670	-2.12190
H	5.27700	1.18220	-3.65740
H	4.94400	-2.49950	-1.46240
H	5.83780	-1.25520	-3.41170
H	2.73810	2.84090	-1.60470
H	3.82500	3.01110	-2.98350
H	5.75940	3.26910	-1.30110
H	4.54310	3.33120	-0.02600
H	4.83970	5.29630	-2.35890
H	5.27130	5.60050	-0.67780
H	2.49320	5.28720	-3.08690
H	0.13940	5.79010	-2.64150
H	1.06340	6.46050	1.50230
H	3.44710	5.93230	1.04590
H	-3.04580	5.46080	0.63730
H	-3.56500	6.92550	-0.19170
H	-1.92030	6.60120	-1.95450
H	-1.61030	4.92560	-1.45120

O	0.16440	-1.67080	-2.92110
N	0.05340	2.02140	-0.71550
N	-0.31110	1.84290	-2.82370
N	-0.48570	2.63630	-1.79470
C	0.71180	-0.40510	-3.32760
C	0.56830	0.82010	-1.05090
C	0.34320	0.71850	-2.40940
C	6.87450	-0.86910	2.64700
C	7.93370	-2.97730	-2.51960
C	7.07550	-4.10990	-2.64640
C	6.81260	-4.56750	-1.37220
N	7.48340	-3.75210	-0.49090
C	7.82840	-1.44810	1.81800
C	8.06630	-0.95160	0.52630
C	7.36360	0.18320	0.12320
C	6.41550	0.78830	0.94890
C	6.13600	0.23770	2.20470
C	8.96240	-1.70530	-0.46290
C	8.17160	-2.77450	-1.17510
C	10.20090	-2.26650	0.18920
C	10.72030	-3.54610	0.22920
C	11.92870	-3.50090	0.98670
C	12.11190	-2.19530	1.38760
N	11.06150	-1.45660	0.89610
O	5.16520	0.68920	3.04960
C	4.37520	1.81300	2.63010
C	3.35190	2.08380	3.72670
C	2.53430	3.37420	3.49230
C	1.20290	3.19310	2.74540
C	1.34130	2.62670	1.32740
C	-0.00990	2.63090	0.61330
O	-5.29490	-2.66930	-3.06420
C	-1.19050	-1.87250	-3.00630
C	-2.11540	-0.94790	-3.50910
C	-3.47260	-1.26170	-3.51460
C	-3.93160	-2.49080	-3.03110
C	-3.00660	-3.42270	-2.54410
C	-1.64740	-3.10710	-2.53380
C	-5.85270	-3.97820	-2.87310
C	-6.16370	-4.28900	-1.40990
C	-6.69840	-5.72610	-1.26200
C	-7.36730	-6.00960	0.09170
C	-6.40790	-5.95920	1.29070
C	-7.13390	-5.84980	2.63400
O	-8.08320	-4.76370	2.67060
C	-7.62980	-3.50610	2.35530
C	-6.36410	-3.02540	2.70350
C	-5.97430	-1.75270	2.28770
C	-6.82160	-0.94050	1.52920
C	-8.10300	-1.41870	1.22750
C	-8.50770	-2.68670	1.63550
C	-6.33320	0.41190	1.02820
C	-6.64720	0.63130	-0.43320
C	-6.82950	1.55140	1.88340
C	-7.88530	1.63730	2.77110
C	-7.90760	2.96410	3.29340
C	-6.86330	3.64890	2.70830
N	-6.21480	2.78420	1.86140
C	-7.22430	1.70420	-1.08680
C	-7.24450	1.40570	-2.48220
C	-6.68300	0.15660	-2.63960
N	-6.32170	-0.29920	-1.39400
H	1.79190	-0.57420	-3.31340
H	0.41720	-0.15970	-4.35530
H	1.05780	0.16350	-0.34140
H	6.66690	-1.27330	3.63280
H	8.33320	-2.36880	-3.31940
H	6.69460	-4.53730	-3.56360
H	6.21960	-5.39850	-1.02070
H	7.48100	-3.85680	0.51200

H	8.39110	-2.30780	2.17400
H	7.52310	0.58800	-0.87280
H	5.87310	1.64800	0.58470
H	9.26920	-0.99320	-1.24020
H	10.27650	-4.41190	-0.24090
H	12.58480	-4.33090	1.21040
H	12.89000	-1.73460	1.97770
H	10.91060	-0.47410	1.06730
H	3.89760	1.58510	1.67050
H	5.02520	2.68930	2.49270
H	3.90130	2.16090	4.67190
H	2.68740	1.21520	3.81980
H	3.14860	4.10540	2.94850
H	2.31420	3.83040	4.46390
H	0.70050	4.16780	2.69440
H	0.54530	2.53610	3.33210
H	1.72240	1.60200	1.36710
H	2.05830	3.22190	0.75210
H	-0.75040	2.08090	1.19780
H	-0.38140	3.64740	0.46860
H	-1.80090	0.02360	-3.86740
H	-4.19460	-0.54150	-3.88200
H	-3.32860	-4.38340	-2.15980
H	-0.92260	-3.81530	-2.14510
H	-5.19170	-4.73570	-3.31390
H	-6.77890	-3.97480	-3.45620
H	-6.91050	-3.57100	-1.04750
H	-5.26380	-4.14060	-0.80300
H	-5.88050	-6.43840	-1.43800
H	-7.43650	-5.90960	-2.05480
H	-7.85590	-6.99190	0.06150
H	-8.16690	-5.27550	0.24240
H	-5.72260	-5.11250	1.18820
H	-5.77980	-6.85860	1.31750
H	-6.41660	-5.76210	3.46020
H	-7.74610	-6.73710	2.81970
H	-5.67680	-3.64320	3.27100
H	-4.98300	-1.39230	2.54320
H	-8.77510	-0.80120	0.63840
H	-9.48800	-3.07490	1.37770
H	-5.24530	0.38880	1.12110
H	-8.55270	0.82520	3.02180
H	-8.60210	3.36390	4.01970
H	-6.52110	4.66640	2.82880
H	-5.41650	3.03190	1.28690
H	-7.58200	2.60150	-0.60400
H	-7.63530	2.03020	-3.27400
H	-6.52740	-0.45840	-3.51120
H	-5.88820	-1.19550	-1.22830

Table S 6. GFN2-xTB-calculated Cartesian coordinates of **2**.

N	2.40460073295462	-1.11754305328799	1.29630232890806
O	-3.96349254674848	2.84788887092704	0.55835273868773
C	3.66483198179296	-1.43944384949960	1.01064906847443
C	1.82468468940901	-1.64701766452206	2.36598257700822
C	2.50092491587309	-2.51140227668768	3.22394617870483
C	3.81718934498629	-2.83195490883058	2.94617532162509
C	4.40997596950673	-2.29997676540355	1.81494969662809
C	0.37554324808770	-1.33464292658074	2.58448492718997
C	-0.48640540627126	-2.37163971696889	1.85297842454255
C	-1.97776736619477	-2.20588965485213	2.16642665050847
C	-2.49970367330047	-0.86947906799525	1.72220535915920
C	-2.73796599590478	0.15361298675895	2.63378205054693
C	-3.21049766270228	1.38071926664398	2.21592347241269
C	-3.46086428454915	1.61487262872956	0.86343767387863
C	-3.22569250683872	0.60062472335669	-0.05690575115690
C	-2.74661761971252	-0.62242331169864	0.37899516685437
C	-4.10882651378268	3.22356365841880	-0.80705149707942
C	-4.35146615878661	4.73099438630451	-0.84732698074076
N	3.85990900372262	0.36891556299726	-0.55221419463605
O	-0.84912879658389	5.95476938883016	-0.32480023180043
C	5.32348234325435	0.29555496366195	-2.42755721086137
C	4.37069902084590	0.93531856458572	-1.63718598719187
C	4.25727121818089	-0.85483260636223	-0.20681207917173
C	5.18642900392687	-1.57267163396432	-0.95775235420613
C	5.73140735600137	-0.98018934713510	-2.08308910191884
C	3.89546103187374	2.31647198916157	-1.97768482833882
C	4.82219624466564	3.38551122755786	-1.38561969586485
C	4.46973046861526	4.79458780085812	-1.88210225970456
C	3.06119753634727	5.16433876519055	-1.51525217191185
C	2.02086331750457	4.98679807584812	-2.41497387879399
C	0.70798183109140	5.24843498946807	-2.06558846989619
C	0.41072661781681	5.69860554441967	-0.78455688925489
C	1.45302975377414	5.91380024953276	0.11744479603647
C	2.75623482609900	5.64557632692893	-0.24635010618792
C	-3.19620367410849	5.52350405712555	-0.23326395694928
C	-1.94584557399302	5.49513348584237	-1.10778700962504
H	2.00071994160676	-2.91519341953523	4.09059900380186
H	4.37380352363995	-3.48948552587978	3.59824853137035
H	5.43751369420426	-2.51561087620814	1.57400225048215
H	0.14662156693127	-0.34433058464353	2.19521304857351
H	0.15046255583489	-1.36241856552052	3.65181492845711
H	-0.17193113894232	-3.37569336679801	2.14860611940551
H	-0.32381448141531	-2.27196754225525	0.77801518902454
H	-2.13501773378945	-2.32400073372559	3.24041787476598
H	-2.52654455411708	-2.99756001194446	1.65072591931428
H	-2.55876596723258	-0.01223942944671	3.68601041089125
H	-3.40217851281025	2.17259636641197	2.92330777514043
H	-3.41816997678448	0.74432695613484	-1.10727062649510
H	-2.57521043266540	-1.40095586513006	-0.34988431289529
H	-4.96423256898494	2.69376507791792	-1.24592755528124
H	-3.20067193564162	2.96066548976141	-1.35730450261035
H	-5.26439871091507	4.95141025625338	-0.29233289488665
H	-4.50349343545153	5.02573796930318	-1.88576591629118
H	5.72376766706337	0.78980259429614	-3.29924759534685
H	5.46184425021264	-2.57701839153497	-0.68180796058970
H	6.46078628993798	-1.51033405208122	-2.67703051617544
H	2.88782622994825	2.45466241103657	-1.58634574240290
H	3.86982485991305	2.42607400532626	-3.06324933994934
H	5.85509779932708	3.16203433263249	-1.66365798724632
H	4.74351071057773	3.35640675115560	-0.29722782719515
H	4.59010639076061	4.83187076963747	-2.96644499857553
H	5.16851642069377	5.50582321536089	-1.43615638925765
H	2.23147313872466	4.63310291517330	-3.41399470716245
H	-0.07002766586736	5.09204003172851	-2.79443698697133
H	1.21363606975710	6.28499862514813	1.10192882433116
H	3.55037316626445	5.81012643857133	0.46774648925339
H	-2.94612344567057	5.09559360138698	0.73863486184797
H	-3.50103989330890	6.55864234322529	-0.07422971390436
H	-2.07547541518503	6.14300387107629	-1.98487963757232
H	-1.73603331867934	4.47266629207129	-1.43992054748920

O	-0.34506249126033	-1.79632474949603	-2.76212041127726
N	0.00319215453043	1.76725030822755	-0.32680670564660
N	-0.72626111849628	1.58057823147889	-2.31448311478197
N	-0.74525275328764	2.32594415247119	-1.26508736887582
C	0.28302190823473	-0.56366603466732	-3.08873595258642
C	0.52947301517014	0.61408767815040	-0.77804686629421
C	0.05180731296844	0.50426678566189	-2.07169142716024
C	7.44365550005722	-0.86906852148596	2.34498503998529
C	9.09819533451068	-2.40374064592699	-2.83918145182275
C	8.37903712781034	-3.57186440589780	-3.16866209134376
C	8.06935219494956	-4.19805710791137	-1.99209822172663
N	8.57078934119257	-3.45374697749856	-0.97325482962193
C	8.46328320365262	-1.35891064035410	1.55641822802468
C	8.76858045141898	-0.76564853870225	0.33537274859730
C	8.02900822502421	0.33715351813206	-0.06155470493742
C	7.00119559028451	0.83760372916253	0.71870766177959
C	6.68787542788081	0.22785054622322	1.92732279909427
C	9.83841368607518	-1.33717557821636	-0.57624791054948
C	9.20645180465862	-2.35998665253001	-1.47203240986402
C	10.98702094981870	-1.91300335283772	0.19894907714875
C	11.58042605788416	-3.14971044279638	0.16716048870695
C	12.63581398390918	-3.13547842199527	1.10342664746630
C	12.65187607512884	-1.89356483148968	1.67557513737781
N	11.65312656741185	-1.16035039864390	1.11860315038896
O	5.68627026990704	0.61684390850618	2.76572666365698
C	4.88438583927798	1.73717623254566	2.40606400483798
C	3.90431052454031	1.98140748205070	3.54914056190439
C	3.07089205235879	3.25643646483338	3.35949119936776
C	1.65090624918319	3.00895621562728	2.85080668489238
C	1.59060113974970	2.45119331587894	1.43107669045637
C	0.13671572318348	2.37448935843693	0.96866638885589
O	-5.83405715367301	-2.44434421572150	-3.07042384702807
C	-1.71002084504957	-1.89710503758959	-2.86186601986912
C	-2.56945064500321	-0.86790370992633	-3.23102908106451
C	-3.93119280109072	-1.09690792897148	-3.28551658239207
C	-4.46672041480365	-2.34562738758900	-2.98139703843706
C	-3.60576470519025	-3.37529184279958	-2.61834037465986
C	-2.24371940238324	-3.14792257095168	-2.56242619193699
C	-6.44663738371449	-3.72429401464608	-3.02021208150326
C	-6.75908759583664	-4.17030234398020	-1.58988649050929
C	-7.22719700280397	-5.62706065328481	-1.54765646009835
C	-7.94662954393359	-5.97799505026815	-0.24452903429888
C	-7.04827459536525	-5.88506848094268	0.99185320306623
C	-7.86505563236005	-5.65132138082638	2.26626157392569
O	-8.74898503868328	-4.54442077733323	2.15730684934287
C	-8.23400701732325	-3.31153729493642	1.83782186395824
C	-6.94541859974652	-2.88558862096067	2.13575681481372
C	-6.53049943733308	-1.62188936715485	1.75089860251772
C	-7.37969547084628	-0.76215833403567	1.07115936467565
C	-8.67574780647877	-1.18471969897619	0.79898129526252
C	-9.10016738689869	-2.44149973430551	1.17686797574508
C	-6.91093602843272	0.61632587820839	0.66655886280553
C	-7.38730939917300	0.97862445708526	-0.71221301176754
C	-7.36265485717612	1.63374908609044	1.67477839579362
C	-8.48669387043281	1.68949347078327	2.46107377365493
C	-8.41556849053563	2.87951874835921	3.21334959512142
C	-7.25213721608439	3.50829556829420	2.86162812915575
N	-6.62301521062029	2.74614614183966	1.93144739518060
C	-8.08197483983656	2.07348445034578	-1.16227804079596
C	-8.25655551899856	1.92098622305485	-2.55370022944660
C	-7.66377137307736	0.73969573359594	-2.90551579674246
N	-7.13631828211049	0.17891731729536	-1.78639018016465
H	1.34715074753934	-0.81355389518355	-3.12726927807480
H	-0.04531152084126	-0.21162185209840	-4.07324031281762
H	1.18206236773873	-0.00745833142347	-0.19833877697126
H	7.20689610016576	-1.32173610993739	3.29562950288662
H	9.49411621609489	-1.68558216014686	-3.53114047926612
H	8.12356509101911	-3.90446124741256	-4.15630186669839
H	7.53428712039881	-5.10993838955480	-1.81243038137451
H	8.51334670985253	-3.67827003495287	0.00568970976122
H	9.03933369232744	-2.20572343977437	1.90184441537818

H	8.25253176988496	0.81724018598840	-1.00308811279152
H	6.44446783953802	1.69082381669912	0.37000973069409
H	10.21423973143606	-0.53093726837260	-1.21856238457476
H	11.30599001195099	-3.97291270369220	-0.46322222558888
H	13.30012760949207	-3.94889583723494	1.32195657993622
H	13.29588486413429	-1.48445542055124	2.42881434133976
H	11.41829632282098	-0.21119992557583	1.35706059964238
H	4.35586618717694	1.52916018360115	1.46849847616998
H	5.52936931015349	2.61679001899529	2.27506171231445
H	4.49175887681622	2.04999055396503	4.46706597722564
H	3.25364423183489	1.11150127307710	3.64331354532173
H	3.58485551135260	3.92917409355921	2.67132237745054
H	2.99331952438769	3.76963334977845	4.31963606386683
H	1.10999455181917	3.95772003596093	2.87436411564761
H	1.14272435532483	2.32246410885589	3.53137052893386
H	2.04020143735649	1.45890273152453	1.39358211430652
H	2.14557572035995	3.10537261626440	0.75654048237311
H	-0.46012660037973	1.79908930585175	1.68490266209156
H	-0.29514185609522	3.37445666495716	0.88713829797263
H	-2.19292515305180	0.11567116648706	-3.46096909113564
H	-4.60238573225741	-0.30207940153842	-3.57115490400575
H	-3.97547086508601	-4.35773482200438	-2.37424002226816
H	-1.56859966560811	-3.94078544438729	-2.27944747456118
H	-5.82075279337098	-4.46292127569037	-3.53284475142799
H	-7.37597068063780	-3.60824257179664	-3.58567812185976
H	-7.53448138143493	-3.51958553908673	-1.17957083356735
H	-5.86478611835907	-4.03543619872310	-0.97939927839724
H	-6.37369794875383	-6.29366427811953	-1.69010475345034
H	-7.92048496969965	-5.79510155370522	-2.37498513208315
H	-8.35787608647423	-6.98678449506582	-0.31985101712316
H	-8.78615434365573	-5.29096926945327	-0.12055932906952
H	-6.35301011730710	-5.05559942404483	0.87100338554664
H	-6.46769057800292	-6.79978460273726	1.11752955970543
H	-7.20143282691195	-5.53231292898733	3.13197449333887
H	-8.53359765281902	-6.49533790925824	2.45419587695643
H	-6.26144898321311	-3.52537170141467	2.67176348552763
H	-5.52360429701477	-1.30370621513685	1.98197112865465
H	-9.35076630162528	-0.52434482540561	0.27516955726424
H	-10.10438176573558	-2.77676088120941	0.96559158135707
H	-5.81227262927931	0.60919962534919	0.66473818447427
H	-9.27208873906905	0.96048287047024	2.50145867315789
H	-9.13950333009875	3.22338293091527	3.92693488629284
H	-6.83211845703503	4.43379143017580	3.20378593053438
H	-5.73661734586903	2.96220084736695	1.49759627997430
H	-8.43350907595796	2.89040652913082	-0.56282336578528
H	-8.76113265122380	2.60460194746247	-3.20879691436254
H	-7.57826398064439	0.25938313624456	-3.86030923653277
H	-6.65549328738916	-0.70643604580339	-1.76547401944780

Table S 7. DFT (PCM)-calculated Cartesian coordinates of **[10-H$_4$]$^{4+}$**.

O	-6.62910	1.52690	0.87060
N	-10.52120	2.95330	0.83470
N	-9.45710	2.21910	2.56360
N	-10.64830	2.40160	2.06700
N	-0.86630	-3.55680	2.47220
C	-5.53830	0.75080	1.00740
C	-11.67880	3.21370	0.02810
N	-1.43010	-3.96180	-0.27350
C	-9.20890	3.13980	0.55650
H	-8.86060	3.57400	-0.36230
C	-8.53490	2.66140	1.66490
C	-5.30450	-0.11590	-0.07820
H	-5.95140	-0.06400	-0.94350
C	-12.85230	3.59580	0.67610
H	-12.83500	3.75280	1.74250
C	-3.66660	-0.23570	2.17590
H	-2.99860	-0.23810	3.02860
C	-4.68770	0.70320	2.12850
H	-4.81330	1.39650	2.94960
C	-4.29530	-1.04820	-0.02040
H	-4.18920	-1.75100	-0.83540

C	-13.99880	3.87030	-0.07350
C	-3.45280	-1.15190	1.11800
C	-11.61560	3.05600	-1.35800
H	-10.70990	2.68510	-1.81270
C	-2.02980	-2.78840	2.38530
C	-7.07200	2.45790	1.89110
H	-6.48100	3.37490	1.80440
H	-6.93390	2.03360	2.88810
C	-13.91350	3.74250	-1.46400
H	-14.79320	3.96540	-2.05550
C	-12.75550	3.31790	-2.12940
C	-2.44280	-2.19340	1.16720
C	-1.89580	-2.66000	-0.07020
C	-15.30900	4.35100	0.57180
C	-2.63190	-2.86490	3.66160
H	-3.58600	-2.43480	3.92290
C	-12.81370	3.12850	-3.65710
C	-1.67120	-1.96980	-1.27210
H	-1.86110	-0.91830	-1.42220
C	-1.82540	-3.66260	4.47350
H	-2.01750	-3.93860	5.49810
C	-13.17890	4.47170	-4.33470
H	-14.14860	4.84890	-3.99770
H	-13.23260	4.34060	-5.42070
H	-12.42940	5.24030	-4.12350
C	-0.71990	-4.07570	3.71180
C	-11.48740	2.62690	-4.25270
H	-10.67160	3.33620	-4.09800
H	-11.59880	2.49540	-5.33340
H	-11.17550	1.67200	-3.82700
C	-13.90260	2.07900	-3.98560
H	-13.96100	1.92550	-5.06820
H	-14.89100	2.39370	-3.63900
H	-13.67100	1.11630	-3.51700
C	-1.09820	-2.86310	-2.18360
H	-0.78330	-2.63910	-3.19040
C	-0.99410	-4.10790	-1.55960
C	-15.56150	5.81750	0.14640
H	-15.63410	5.91500	-0.94110
H	-14.75070	6.46900	0.49020
H	-16.49760	6.18430	0.58180
C	-16.48300	3.46480	0.09450
H	-16.31790	2.41860	0.37350
H	-16.62060	3.50870	-0.98950
H	-17.41660	3.79850	0.55950
C	-15.25950	4.28900	2.10930
H	-16.22050	4.61150	2.52060
H	-14.48500	4.93920	2.52330
H	-15.07010	3.27020	2.46280
C	-0.46150	-5.42160	-2.09910
O	9.26790	-3.69160	-0.12250
N	11.21860	0.11730	-1.22990
N	9.33420	-0.86160	-1.07940
N	9.89940	0.31660	-1.04770
N	1.98890	-5.03720	2.07240
C	7.96180	-4.02890	-0.12760
C	12.14530	1.20650	-1.18180
N	1.58680	-4.94860	-0.72970
C	11.49210	-1.20480	-1.37080
H	12.48840	-1.58680	-1.50610
C	10.27100	-1.83150	-1.27430
C	7.47180	-4.51000	1.10320
H	8.17430	-4.64980	1.91750
C	11.96900	2.19290	-0.21320
H	11.11650	2.13080	0.44820
C	5.73580	-4.13850	-1.05020
H	5.06030	-3.92140	-1.86830
C	7.08320	-3.86650	-1.21490
H	7.41520	-3.42860	-2.14760
C	6.12550	-4.76600	1.26130
H	5.76740	-5.15090	2.20770

C	12.90520	3.22580	-0.10360
C	5.21270	-4.57170	0.19010
C	13.20790	1.22710	-2.08100
H	13.28210	0.44320	-2.82340
C	3.20160	-4.48870	1.64850
C	9.94870	-3.28870	-1.33250
H	9.34180	-3.51670	-2.20860
H	10.86810	-3.87280	-1.38840
C	13.96840	3.23910	-1.01790
H	14.69750	4.03880	-0.94680
C	14.14200	2.26720	-2.01410
C	3.78500	-4.75480	0.37390
C	2.96440	-5.16860	-0.70520
C	12.82360	4.30060	0.99670
C	3.61970	-3.62690	2.67810
H	4.50060	-3.00660	2.63940
C	15.33680	2.37630	-2.97870
C	3.25610	-5.91000	-1.86970
H	4.22870	-6.30270	-2.12260
C	2.66730	-3.67680	3.70050
H	2.68170	-3.11390	4.62140
C	16.65030	2.32060	-2.16460
H	17.51280	2.40070	-2.83480
H	16.72920	1.37550	-1.61700
H	16.71340	3.13730	-1.44060
C	1.67420	-4.58520	3.31970
C	15.36280	1.23460	-4.01030
H	15.46510	0.25740	-3.52690
H	16.22110	1.36160	-4.67690
H	14.46040	1.22690	-4.63130
C	15.25490	3.71910	-3.74220
H	14.33170	3.77880	-4.32830
H	16.10240	3.81280	-4.42960
H	15.28090	4.57650	-3.06330
C	2.06100	-6.11380	-2.56270
H	1.93450	-6.66820	-3.47890
C	1.03340	-5.48700	-1.84220
C	12.69480	5.69620	0.34350
H	13.54910	5.92350	-0.30100
H	12.64450	6.47050	1.11660
H	11.78580	5.76050	-0.26420
C	11.61920	4.08420	1.93310
H	11.66780	3.11430	2.44070
H	10.66920	4.14540	1.39260
H	11.61140	4.86100	2.70390
C	14.11350	4.24480	1.85020
H	14.22640	3.26300	2.32220
H	14.07230	5.00110	2.64130
H	15.00890	4.43780	1.25230
C	-1.12670	-6.62520	-1.37710
C	-0.74920	-5.51280	-3.61130
C	0.41920	-5.02180	4.05380
C	0.68050	-5.01430	5.57450
C	-0.00730	-6.45070	3.61580
H	-0.87620	-6.76730	4.19690
H	0.90740	-4.01040	5.93860
H	-0.19860	-5.38090	6.10830
H	1.52040	-5.67240	5.80840
H	-0.29120	-6.49020	2.55880
H	0.80790	-7.15780	3.79510
H	-1.82370	-5.42680	-3.78970
H	-0.41440	-6.47640	-4.00030
H	-0.23680	-4.72410	-4.16570
H	-0.77380	-7.55860	-1.82110
H	-2.21400	-6.57580	-1.48810
H	-0.87540	-6.65570	-0.31170
H	-0.14240	-3.55550	1.77190
H	1.11860	-4.27770	-0.14100
N	-12.03940	4.91030	3.55230
O	-7.72290	5.98310	-3.29400
O	-9.34790	0.88670	-2.29300

N	-12.67580	2.27750	4.04030
C	-12.12840	4.51110	4.82980
C	-11.54910	6.12800	3.27420
C	-12.79150	3.19990	5.03060
C	-10.72220	7.68950	-0.90250
C	-11.53520	6.51670	1.81190
H	-12.42730	6.08540	1.34990
H	-10.68830	6.01580	1.32430
C	-11.09720	6.97360	4.29920
H	-10.70640	7.95640	4.06370
C	-11.15810	6.54140	5.62130
H	-10.80060	7.17930	6.42330
C	-11.69060	5.28580	5.90820
H	-11.75120	4.92170	6.92610
C	-11.49090	8.01840	1.50870
H	-10.51940	8.43770	1.79560
H	-12.24770	8.53850	2.10750
C	-8.43860	7.60600	-1.75830
H	-7.42770	8.00020	-1.79410
C	-9.41270	8.19530	-0.96080
H	-9.15130	9.06610	-0.36390
C	-11.01750	6.57460	-1.68960
H	-12.01600	6.14590	-1.66630
C	-11.74810	8.31750	0.01650
H	-12.75060	7.96300	-0.25200
H	-11.75220	9.40540	-0.12160
C	-13.58820	2.89310	6.12770
H	-13.71460	3.61930	6.91900
C	-10.05350	5.97730	-2.50780
H	-10.33310	5.12260	-3.10420
C	-13.31490	1.08660	4.02770
C	-8.75160	6.48390	-2.53930
C	-9.05460	-0.36620	-0.20330
H	-7.98990	-0.20040	-0.17080
C	-11.20630	-0.13200	-1.27460
H	-11.79870	0.22660	-2.10810
C	-11.05190	-1.25550	0.88130
C	-11.80370	-0.80590	-0.21370
H	-12.87720	-0.97550	-0.23360
C	-9.82850	0.12070	-1.25980
C	-9.67190	-1.03630	0.85290
H	-9.05830	-1.38080	1.68220
C	-7.70320	2.16440	-3.48980
H	-8.38550	1.99640	-4.33080
H	-6.68420	2.06150	-3.88080
C	-11.95930	-0.87660	3.23150
H	-12.25310	-1.41860	4.13790
H	-11.02980	-0.34190	3.46150
C	-14.13480	0.76860	5.10900
H	-14.66170	-0.17770	5.10910
C	-7.89090	3.56650	-2.89570
H	-8.84610	3.60200	-2.36740
H	-7.10390	3.75770	-2.15510
C	-14.25840	1.66900	6.16360
H	-14.89500	1.43020	7.00900
C	-11.70530	-1.88690	2.09110
H	-11.06180	-2.68190	2.48270
H	-12.65410	-2.35950	1.80970
C	-13.06090	0.15020	2.88140
H	-12.77960	0.71380	1.98800
H	-13.99320	-0.37820	2.65760
C	-7.93270	1.07290	-2.44470
H	-7.48000	0.12160	-2.75190
H	-7.48810	1.38010	-1.49110
C	-7.85140	4.69960	-3.92120
H	-6.96130	4.61780	-4.55140
H	-8.72690	4.67570	-4.58370
N	6.63090	-0.79250	-0.78570
O	12.62860	-3.92790	2.62910
O	14.47230	-4.37030	-1.36510
N	7.44760	-1.40240	-3.42460

C	5.77750	-0.67840	-1.83110
C	6.23920	-0.82170	0.50940
C	6.34180	-0.66210	-3.20520
C	9.93260	-0.73920	2.96900
C	7.29130	-0.91110	1.57600
H	8.06790	-1.62070	1.28130
H	6.82580	-1.30420	2.48540
C	4.87750	-0.73570	0.79340
H	4.55370	-0.75040	1.82660
C	3.96330	-0.62700	-0.25090
H	2.90030	-0.57400	-0.03920
C	4.41030	-0.59920	-1.57080
H	3.71110	-0.54070	-2.39320
C	7.94880	0.45520	1.87680
H	7.17380	1.20310	2.08110
H	8.49320	0.78990	0.98840
C	11.97320	-1.65730	1.98420
H	12.83050	-1.51730	1.33600
C	11.05260	-0.61920	2.14110
H	11.22180	0.30710	1.60520
C	9.76540	-1.95080	3.65770
H	8.90670	-2.07790	4.31270
C	8.91180	0.37420	3.08260
H	9.41800	1.34180	3.17380
H	8.32480	0.23750	3.99850
C	5.72040	0.10660	-4.19320
H	4.85560	0.71730	-3.96700
C	10.67350	-2.99650	3.52190
H	10.54180	-3.93050	4.05910
C	7.97630	-1.41500	-4.65710
C	11.78890	-2.85510	2.68510
C	12.76260	-4.75060	-3.08730
H	12.50080	-5.73510	-2.71880
C	14.12290	-2.75300	-3.02450
H	14.93550	-2.19460	-2.56960
C	12.37140	-2.96310	-4.71020
C	13.42670	-2.25090	-4.11820
H	13.71120	-1.28600	-4.52880
C	13.78110	-4.00210	-2.48320
C	12.06980	-4.22160	-4.18290
H	11.27830	-4.81460	-4.63460
C	15.08110	-5.80170	0.45600
H	15.96790	-5.15720	0.44710
H	15.43930	-6.82460	0.61330
C	10.41410	-1.46220	-5.44130
H	10.74190	-0.75290	-4.67200
H	10.10980	-0.86610	-6.30870
C	7.40180	-0.67870	-5.70870
H	7.84360	-0.71850	-6.69830
C	14.13910	-5.43560	1.61270
H	13.30280	-6.14550	1.63510
H	14.66630	-5.54140	2.56730
C	6.27120	0.09070	-5.47400
H	5.82340	0.67430	-6.27170
C	11.60330	-2.35540	-5.86580
H	11.23700	-3.14510	-6.53360
H	12.28810	-1.74100	-6.46110
C	9.20470	-2.26280	-4.91690
H	8.94270	-3.02800	-5.66030
H	9.48510	-2.79110	-4.00440
C	14.41910	-5.73560	-0.91690
H	14.93420	-6.36630	-1.65090
H	13.37850	-6.07730	-0.84380
C	13.55430	-4.02950	1.53640
H	14.34190	-3.26860	1.62310
H	13.02470	-3.85880	0.59130
H	1.55780	-5.83850	1.63210
H	-1.67190	-4.73590	0.33090
H	7.68190	-0.85920	-0.98240
H	-12.01870	2.47290	3.25390

Table S 8. GFN2-xTB-calculated Cartesian coordinates of **[12-H$_3$]$^{3+}$**.

C	-5.27250384149964	-1.52933127540756	2.24483849733431
C	-4.22097928690027	-2.11148645851174	2.94761018265875
H	-3.97753031412364	-1.94273544935673	3.97649730614415
C	-3.55412255941467	-2.96304122027295	2.07969448710481
H	-2.67432192045641	-3.53360204615649	2.29641300522091
C	-4.19315303661456	-2.89047356809528	0.83140549858520
C	-3.87727465801358	-3.54221345764020	-0.38240650556170
C	-4.31940204037857	-3.02188634275272	-1.62044873308400
C	-4.55512999322935	-3.67028025097713	-2.84398966309638
H	-4.47757059016897	-4.72441693044001	-3.01980016063550
C	-4.98629913422146	-2.71354193349569	-3.75231503804101
H	-5.26382792171835	-2.89278661009701	-4.77088172085031
C	-4.99009250002179	-1.48371460764550	-3.09870228052579
C	-5.35747637813521	-0.11527756342228	-3.61248091216288
C	-6.86789721976835	0.09830276481319	-3.38668031504809
H	-7.14877897070707	1.10979336577751	-3.67072000285106
H	-7.16029165427067	-0.07918316595880	-2.35515069512030
H	-7.42994071069297	-0.59288418968146	-4.00939005860963
C	-5.05247552544241	-0.01399843875436	-5.11211471859447
H	-5.71603609345199	-0.66194032351360	-5.67684503226929
H	-4.02842495176480	-0.30600445172022	-5.32505527477500
H	-5.20684535409039	1.00543733878967	-5.45631858977578
C	-4.55963814944363	0.91597431120388	-2.86065898880045
C	-3.34108890816010	1.50443552705320	-3.19042952310119
H	-2.76656126618712	1.31791656942045	-4.07399559365938
C	-3.00874953291747	2.38842434823449	-2.17177318571513
H	-2.11094185275367	2.96666292453671	-2.09614392706890
C	-4.02270251551723	2.32408126823269	-1.20412771253621
C	-4.18236372620007	3.02475771452004	0.01621137769902
C	-4.97486333139998	2.45631762745534	1.04354221356429
C	-5.79334279421083	3.07345929148778	1.99888034170722
H	-5.95799460129197	4.12791770096308	2.09693943079487
C	-6.41827967675983	2.06829669527174	2.72558632298102
H	-7.12754777691014	2.21298768222046	3.51473928667547
C	-5.95050521103040	0.84693558157062	2.24795495638715
C	-6.30485539593907	-0.54178950740651	2.71478547276657
C	-7.68820218610216	-0.90690220918868	2.13537901820124
H	-7.72589975420574	-0.79203460862724	1.05545436420301
H	-8.44633073368809	-0.25605975721860	2.56241190485681
H	-7.94046547613964	-1.93249814347070	2.39411967355435
C	-6.38304072234148	-0.56492851065259	4.24777836468199
H	-7.20477600939631	0.05408952104304	4.59447243591044
H	-5.46577743061930	-0.19183657897107	4.69505764796266
H	-6.55755564022383	-1.57927409305005	4.59714572690670
C	-3.06408838955734	-4.72870926117784	-0.35908207012037
C	-2.19617510201987	-5.02114254686166	-1.42312273903828
H	-2.15806856568124	-4.35789707138034	-2.27144614543160
C	-1.33661599260277	-6.09289449997362	-1.37617452323264
H	-0.68940565169539	-6.28285573059337	-2.21603632909382
C	-1.29840192661007	-6.91756508989580	-0.24528272951000
C	-2.21840442756572	-6.67782966807051	0.79114372920799
H	-2.22555245395296	-7.36000989110387	1.62683137568237
C	-3.07843689671735	-5.61367392496059	0.73578424651238
H	-3.80278449213323	-5.47959688965144	1.52350746446897
C	0.58684953694527	-8.23303347411075	-0.96615119610037
H	0.17348970249390	-8.36194686359533	-1.97245251531228
H	0.97301616471075	-9.19609749396218	-0.62419587473091
C	1.70844896569664	-7.19141723719928	-0.97046576727548
H	1.32581272115174	-6.23618474441985	-1.33200871380003
H	2.47377403986328	-7.53563463059781	-1.66733441220987
C	2.32164694810668	-6.97564821377242	0.41232029658065
H	1.52911472003877	-6.73461700232888	1.12511168057826
H	2.79846880892067	-7.89681181381403	0.75179947517353
C	3.34701867437524	-5.84340915010354	0.38245727163925
H	2.87277251756317	-4.94295716852476	-0.01522076629679
H	4.15993490646506	-6.11136174933698	-0.29490195392569
C	3.92037077174381	-5.54786547136304	1.76660703265255
H	3.11155661933247	-5.30406704433371	2.45930702075514
H	4.42891623740215	-6.43315902219403	2.14941000870481
C	4.93213606646444	-4.39725171702951	1.73538113893712

H	5.39583357218534	-4.27913949080058	2.71710924338815
H	5.70453693099602	-4.59813519308948	0.99077656504088
C	3.55999902281713	-2.33937415543470	2.13976276097829
H	3.36698917247146	-2.52053729727835	3.17456067630543
C	3.18217028605978	-1.30737372671038	1.29883504718475
C	2.35327809100170	-0.10918018741198	1.62732081175409
H	1.67970526580965	-0.37969480488489	2.44545655981131
H	2.99407324833137	0.70665065952708	1.98376835587535
C	2.00136841457384	1.25576117718284	-0.32252250900819
C	3.31692380088291	1.69614220709234	-0.39836683907723
H	4.08865132959239	1.29326000807524	0.23603508353801
C	3.66460004366569	2.65205167840327	-1.33296252906585
H	4.68002238326230	3.01099048696463	-1.39095236122803
C	2.72918083690198	3.17293538140608	-2.22542619203913
C	1.42201853620784	2.69749554244113	-2.17193229967921
H	0.67565111910228	3.04588457273681	-2.86599947011110
C	1.06460059194319	1.76102166326477	-1.21996057530657
H	0.05204607521387	1.39472800757598	-1.16329509941851
C	2.34317208665442	4.72938788136145	-4.03202117781826
H	1.76594847314668	3.97391817089911	-4.57895699559706
H	3.02003135425636	5.22312365439329	-4.73415400533682
C	1.40950178530394	5.76428014760017	-3.39956608008957
H	0.78049349394935	5.27161834853305	-2.65909576057699
H	0.77008703483618	6.16232722805777	-4.18945278262714
C	2.17528567160542	6.90836538992221	-2.73451391446607
H	2.76543842705569	7.42595766851696	-3.49333011985846
H	2.87185725827231	6.49967378340860	-1.99939263346642
C	1.24684435430672	7.91972011267869	-2.05547907260933
H	1.81525926264086	8.81810665132333	-1.80925562541887
H	0.46441517678417	8.21164824454837	-2.75988461281985
C	0.61441831989704	7.36423937883793	-0.78053534542390
H	0.15656725169905	6.39605927761810	-0.98863641724422
H	1.38550521920272	7.20852601794053	-0.02359975485799
C	-0.46326311304572	8.29066151199408	-0.21100279748657
H	-0.01660795565879	9.17018825812267	0.25641239204460
H	-1.14726433417625	8.62944866576591	-0.99632808066056
C	-1.98583821038961	6.61567994627848	0.56560402918667
C	-2.41640307441464	5.85881175806471	1.67110777702429
H	-2.10712112219722	6.17996733039730	2.65317023151492
C	-3.16790444665360	4.72780266815773	1.49748027092707
H	-3.43754777672626	4.13593552246715	2.35735865896109
C	-3.52930366908357	4.28673164574054	0.20792979264791
C	-3.17855349766514	5.10486407557158	-0.88124002980816
H	-3.53998203423007	4.86103794395749	-1.86784101703636
C	-2.42305108423122	6.23849301206793	-0.71286155964973
H	-2.18321267812049	6.84306427597676	-1.57236178111730
N	-5.21713553683986	-1.97798053773146	0.97292396788475
N	-4.63728856688256	-1.69649968903750	-1.81620147946116
H	-4.39963498226126	-0.96966904980611	-1.15926518437132
N	-4.92597729609543	1.38214856114243	-1.64788701964205
N	-5.13132305652998	1.09289235533039	1.20298236591550
H	-4.46005520307242	0.42671202740223	0.84524365073163
N	4.30794192695739	-3.15374284608746	1.37032339627895
N	4.38037445957041	-2.67771783936039	0.14773186602007
N	3.71597182734798	-1.57072049386043	0.09105665317379
O	-0.45245787842287	-7.93657443116341	-0.03965078932977
O	1.52066823255357	0.33184960179557	0.57615576027624
O	3.21373729699832	4.09985274267085	-3.10487091693043
O	-1.18496061264550	7.65363206152571	0.83965652764203
C	3.53919093300579	-0.48561199749913	-3.27998450812030
C	3.87887713910019	0.00561442398368	-4.53380748910436
H	3.15480050155120	-0.00576844503154	-5.33168963193290
C	5.15028068215519	0.51302066312425	-4.74736118653487
H	5.42391619885880	0.91374157229401	-5.71095346609546
C	6.07546831766446	0.49793074590931	-3.71532050641296
H	7.06426184342425	0.89749560347238	-3.86310823612858
C	5.70349850221823	-0.01628270013316	-2.47839732001817
C	6.64754001439444	-0.08479383717776	-1.35165261327151
C	8.00773465404574	-0.27748555110697	-1.57990300545370
H	8.39303999594433	-0.41143977662390	-2.57682847274240
C	8.85830288584039	-0.33470954176655	-0.48549577052271

H	9.91723798986955	-0.49307644107608	-0.62320178351478
C	8.32704936380894	-0.18584638456388	0.78100835500391
H	8.95864983600450	-0.21894685416452	1.65468210577385
C	6.94939268515350	0.00203663158342	0.92055757433227
C	6.35409671941388	0.20356838872243	2.28189221888742
H	6.82367207467771	-0.48828343833495	2.98390366495785
H	5.28370748374426	0.00420507046554	2.25353523981126
C	6.59096355857271	1.63933416491492	2.76884965290995
H	6.06621573750267	2.33614967565694	2.11221792416543
H	7.65728514386022	1.86732085169358	2.71488588022762
C	6.11828450845465	1.84157335334154	4.21533013441616
H	6.62162862496683	1.12081696606691	4.86227793145810
H	6.41334822153146	2.84307767071191	4.53567273321276
C	4.62944348933487	1.69525412210129	4.35090209103892
C	3.78319293075800	2.73180522120499	3.98251057291155
H	4.20454539907142	3.66104175654233	3.62722917270069
C	2.40585597369593	2.61020387679837	4.06603875536989
H	1.78867077520949	3.44673609565816	3.78006214754725
C	1.84555242254360	1.42266376217154	4.52065333923363
C	2.68736754549998	0.38385608858915	4.91855074758228
H	2.24081400244314	-0.52135520136486	5.30054950963039
C	4.05723617960336	0.52232905225097	4.83264532694712
H	4.69366736256644	-0.28753441981052	5.15727911804347
C	-0.42012409287271	2.21798474949314	4.38180778807382
H	-0.26524779209525	2.68069107974557	3.39905617127821
H	-0.25873819521594	2.97608555451460	5.15835096431355
C	-1.83214528010291	1.63975781734482	4.48250136535707
H	-1.80264630067528	0.81353943247432	5.19474416889937
H	-2.49273267243022	2.40736744592556	4.88606165343367
C	-2.39765325093977	1.16758193306774	3.14369423124100
H	-2.39049507048742	1.99850144048421	2.43631132223585
H	-3.42877077907234	0.84932615801096	3.29703813018605
C	-1.61368972744107	0.01308498798904	2.53008938943641
H	-0.55925729252072	0.28148327593890	2.42159104262393
H	-1.68872646136970	-0.87623948450109	3.16500251571731
C	-1.41340254366560	-1.00406036321041	0.37148009611893
C	-1.64413588735911	-0.79135697284226	-0.98342222011154
H	-2.34199693282330	-0.02676651571130	-1.28105564316095
C	-0.93896282474766	-1.50018263272630	-1.93355814312236
H	-1.12272289155750	-1.30786221005582	-2.97949075824395
C	0.01821842735650	-2.43674830475825	-1.56447832722283
C	0.22420170528886	-2.65759088935761	-0.20953130329269
H	0.95334982273656	-3.38934462148150	0.10524794083379
C	-0.47840050044145	-1.95654505562656	0.75441753422245
H	-0.28420902874671	-2.14671908744255	1.79781372678534
C	0.82848902583800	-3.15142827712061	-2.60863058287734
H	0.31849401667105	-3.08377856583002	-3.57099052603138
H	0.91799572560818	-4.20665416578300	-2.34718471751617
C	2.24403012783898	-2.57252202224561	-2.75025804783338
H	2.82051386291745	-2.78123069622549	-1.85135512847812
H	2.73875740074356	-3.06280853682151	-3.59032046636583
C	2.19567295232381	-1.05448712323838	-2.98460533667931
H	1.52788420424597	-0.84249266798800	-3.81847808255333
H	1.79449838582595	-0.58311936887138	-2.08728630998871
N	4.45343750387102	-0.46612332647477	-2.30549654595759
N	6.13692806404158	0.03854889094705	-0.12393691685696
O	0.50427561076465	1.15477130323287	4.57431921314125
O	-2.16477109983188	-0.24722231986997	1.24098762948571
H	4.17870276017263	-0.82249895408139	-1.35112490965635
H	-5.95604398610186	-1.85773446569570	0.29753440119664
H	-5.83469294752938	1.24701083379062	-1.23235982775720

Table S 9. DFT-calculated Cartesian coordinates of **12-I** (*E* = 7.6 kcal/mol with respect to **12-II**)

C -6.885759 16.657201 -1.098277
C -5.885215 15.869793 -1.747483
H -6.054531 15.050451 -2.418715
C -4.683617 16.376608 -1.348582
H -3.702915 16.065625 -1.652191
C -4.973281 17.468517 -0.462549
C -4.064482 18.285005 0.198299
C -4.478750 19.425746 0.928026
C -3.756013 20.362747 1.677800
H -2.693916 20.369842 1.815097
C -4.675174 21.250058 2.224195
H -4.446358 22.090758 2.848595
C -5.946478 20.848804 1.814239
C -7.307374 21.380474 2.177824
C -8.173497 21.509851 0.913934
H -8.329534 20.537830 0.457226

H -7.690088 22.167830 0.195085
H -9.144608 21.925010 1.174994
C -7.193487 22.745491 2.857651
H -8.181803 23.110498 3.128338
H -6.734931 23.458649 2.176249
H -6.587030 22.684287 3.757070
C -7.912590 20.348431 3.099970
C -8.210680 20.485150 4.492478
H -8.109990 21.375644 5.081727
C -8.650747 19.264364 4.910167
H -8.994286 18.988383 5.888734
C -8.615763 18.402351 3.761636
C -8.952814 17.057726 3.700772
C -8.990458 16.346105 2.477745
C -9.238676 14.996100 2.190185
H -9.474733 14.228022 2.899658
C -9.097282 14.834710 0.818179
H -9.225645 13.918960 0.276261
C -8.756886 16.075704 0.278470
C -8.383687 16.461088 -1.127512
C -9.068239 17.782516 -1.513094
H -10.148028 17.685173 -1.424316
H -8.820547 18.036712 -2.541525
H -8.728907 18.589887 -0.872170
C -8.791675 15.369246 -2.117175
H -8.313881 14.424071 -1.874976
H -8.504247 15.655257 -3.126549
H -9.870282 15.231406 -2.089156
C -2.645927 17.916236 0.194404
C -2.281133 16.605234 0.509136
H -3.047263 15.890732 0.769403
C -0.960908 16.224235 0.524864
H -0.687228 15.206958 0.749503
C 0.049996 17.141986 0.233623
C -0.300920 18.450588 -0.081765
H 0.454893 19.170467 -0.347265
C -1.632021 18.823152 -0.102459
H -1.890651 19.831113 -0.391122
C 2.425627 17.545104 0.203199
H 2.459526 18.129832 1.130080
H 2.313050 18.226919 -0.645725
C 3.700729 16.719958 0.022339
H 4.553662 17.397055 0.082479
H 3.681201 16.286705 -0.977554
C 3.839172 15.586493 1.040512
H 2.872570 15.084101 1.104176
H 4.565205 14.860330 0.670012
C 4.268426 16.049156 2.433337
H 3.713505 16.946347 2.708319
H 5.330309 16.301316 2.427900
C 3.996637 14.951353 3.458690
H 2.921821 14.749728 3.481679
H 4.496749 14.028812 3.157964
C 4.450160 15.319462 4.876310
H 5.536491 15.265315 4.959203
H 4.110105 16.322986 5.140902
C 2.668543 14.462771 6.400444
H 2.035507 15.321586 6.351438
C 2.517388 13.248394 7.036884
C 1.352237 12.731216 7.807658
H 1.126014 11.711226 7.469448
H 1.588461 12.696611 8.881447
C -0.963106 13.303461 8.090545
C -1.961737 14.245406 7.864854
H -1.716476 15.137812 7.310537
C -3.245185 14.050304 8.337197
H -3.987246 14.807124 8.142564
C -3.559038 12.892982 9.043817
C -2.556382 11.952961 9.270418
H -2.804350 11.060023 9.822457
C -1.270204 12.151338 8.804636
H -0.521716 11.402572 9.007703
C -5.825733 13.559729 9.434117
H -5.988154 13.811424 8.378331
H -5.528601 14.467866 9.976729
C -7.108400 12.986009 10.027371
H -7.812851 13.808154 10.147100
H -6.885039 12.563392 11.008184
C -7.719148 11.917616 9.123233
H -6.991225 11.113537 8.992519
H -7.905593 12.354823 8.140832
C -9.014506 11.317749 9.672873
H -9.346583 10.536855 8.983895
H -8.806051 10.842263 10.633817
C -10.163799 12.310603 9.876248
H -11.040768 11.753697 10.210705
H -9.907275 13.030414 10.653689
C -10.547844 13.090916 8.619528
H -10.509617 12.441083 7.736818
H -11.565455 13.491417 8.722436
C -9.569151 14.850514 7.300785
C -8.460446 15.686881 7.149671
H -7.746924 15.741683 7.956823
C -8.280564 16.406356 5.991342
H -7.400310 17.019711 5.871106
C -9.203383 16.322633 4.945790
C -10.317421 15.506017 5.113221
H -11.061142 15.457904 4.332249
C -10.505364 14.776249 6.274038
H -11.384244 14.158589 6.372932
N -6.346816 17.606776 -0.362523
N -5.799065 19.769291 1.034073
H -6.505158 19.157225 0.623551
N -8.172615 19.131822 2.671577
N -8.712269 16.957577 1.285595
H -8.409295 17.932192 1.287948
N 3.880331 14.387757 5.812240
N 4.434420 13.214353 6.071253
N 3.633952 12.523955 6.803659
O 1.316066 16.650014 0.276808
O 0.268133 13.606944 7.562971
O -4.793061 12.590557 9.547404
O -9.631612 14.167441 8.474021
C 1.548231 21.744526 -0.288960
C 1.000527 22.951384 -0.715294
H 0.785109 23.738543 -0.012029
C 0.727352 23.120845 -2.062230
H 0.284024 24.039378 -2.419483
C 1.029024 22.100413 -2.946459
H 0.810758 22.194135 -3.998472
C 1.599554 20.932024 -2.441240
C 1.975375 19.832805 -3.354605
C 2.552379 20.129657 -4.588362
H 2.746452 21.152598 -4.869812
C 2.902758 19.083108 -5.424786
H 3.364122 19.277859 -6.382435
C 2.657769 17.788148 -5.008842
H 2.921706 16.947026 -5.631162
C 2.076405 17.577611 -3.757833
C 1.799652 16.178781 -3.288038
H 1.548520 16.188305 -2.229213
H 2.706124 15.586003 -3.433059
C 0.658094 15.531131 -4.083026
H 0.766184 15.780261 -5.141499
H -0.294825 15.938376 -3.739477
C 0.646826 14.000957 -3.948821
H -0.117913 13.604079 -4.620330
H 1.615221 13.610589 -4.269131
C 0.356330 13.545447 -2.546988
C 1.376860 13.273973 -1.648971
H 2.405716 13.357875 -1.965951
C 1.112431 12.888715 -0.345105

H 1.935558 12.672707 0.317819
C -0.202336 12.774551 0.087222
C -1.237386 13.007230 -0.819052
H -2.255044 12.893497 -0.478124
C -0.956267 13.388994 -2.113010
H -1.771329 13.573890 -2.797835
C 0.370611 12.514164 2.406594
H 1.066372 13.340018 2.224921
H 0.942068 11.576808 2.446630
C -0.375155 12.729463 3.723514
H -1.083833 11.910500 3.852195
H 0.346036 12.693390 4.541489
C -1.144556 14.050487 3.763446
H -1.782229 14.069061 4.648304
H -1.785905 14.128635 2.884967
C -0.222715 15.263867 3.815656
H 0.426698 15.189526 4.698769
H 0.395541 15.323016 2.912352
C -0.411380 17.646558 3.826796
C 0.961277 17.839674 3.744742
H 1.635271 16.999195 3.732299
C 1.477976 19.124061 3.675570
H 2.549557 19.256882 3.616014
C 0.654162 20.237958 3.696061
C -0.718845 20.032088 3.795903
H -1.382549 20.883409 3.834398
C -1.249234 18.761613 3.856331
H -2.313906 18.602196 3.927150
C 1.208956 21.630193 3.583293
H 2.271734 21.629516 3.832164
H 0.693404 22.280996 4.293193
C 1.021084 22.202487 2.169920
H -0.032204 22.126444 1.893379
H 1.296842 23.258574 2.184270
C 1.871544 21.450211 1.148290
H 2.929130 21.679880 1.311471
H 1.749127 20.376341 1.295352
N 1.834209 20.766414 -1.143545
N 1.754740 18.581865 -2.954617
O -0.585943 12.447884 1.361097
O -1.026685 16.428666 3.905010

Table S 10. DFT-calculated Cartesian coordinates of **12-II** (*E* = 0.0 kcal/mol)

C -5.278027 17.541607 -0.589167
C -4.319969 16.971612 -1.486044
H -4.532505 16.380707 -2.355551
C -3.094296 17.343326 -1.021114
H -2.134665 17.130729 -1.451973
C -3.325821 18.134926 0.155197
C -2.374972 18.764541 0.943688
C -2.723957 19.628337 2.008468
C -1.935390 20.359372 2.908734
H -0.864079 20.391630 2.929682
C -2.799822 21.021583 3.770614
H -2.514361 21.676005 4.570103
C -4.102409 20.683429 3.403967
C -5.419999 21.062590 4.021338
C -6.353412 21.619467 2.930753
H -6.549393 20.866230 2.174163
H -5.899256 22.488150 2.459142
H -7.301847 21.915196 3.374128
C -5.222043 22.122676 5.105859
H -6.180340 22.389270 5.545810
H -4.782189 23.017032 4.670568
H -4.566412 21.760622 5.893501
C -6.025641 19.800249 4.587265
C -6.312253 19.513092 5.960905
H -6.141439 20.162651 6.796551
C -6.846593 18.260813 5.986070
H -7.203270 17.711309 6.835800
C -6.880582 17.804019 4.623998
C -7.405244 16.613986 4.149355
C -7.482067 16.314442 2.768242
C -7.933845 15.169868 2.097209
H -8.326030 14.282488 2.554173
C -7.761541 15.396832 0.737220
H -8.014451 14.716021 -0.051109
C -7.205661 16.667378 0.587439
C -6.781262 17.411549 -0.648605
C -7.407624 18.817777 -0.638005
H -8.491621 18.743745 -0.584977
H -7.134956 19.347849 -1.548002
H -7.046999 19.389384 0.211242
C -7.231385 16.667795 -1.906817
H -6.807622 15.667627 -1.942593
H -6.916195 17.211960 -2.794294
H -8.315990 16.587181 -1.918353
C -0.952356 18.503505 0.690484
C -0.493922 17.187221 0.627207
H -1.190125 16.376900 0.781427
C 0.839579 16.912783 0.421985
H 1.194624 15.894913 0.383919
C 1.760811 17.950349 0.281191
C 1.309795 19.267422 0.321103
H 2.001051 20.084468 0.178904
C -0.031448 19.534496 0.524049
H -0.374952 20.557840 0.525054
C 4.083022 18.393320 0.678122
H 3.643873 19.085095 1.401794
H 4.583182 18.973989 -0.106800
C 5.090670 17.473698 1.375896
H 5.780954 18.098416 1.944314
H 5.658939 16.936531 0.614821
C 4.422254 16.446050 2.293243
H 3.751415 15.831723 1.690220
H 5.193820 15.793597 2.705084
C 3.631068 17.060786 3.445514
H 2.853826 17.720130 3.054944
H 4.299181 17.655801 4.071619
C 2.964614 15.980227 4.294025
H 2.293405 15.387700 3.668952
H 3.725083 15.314376 4.706577
C 2.161643 16.603135 5.435308
H 2.815778 17.167595 6.102314
H 1.395668 17.272859 5.034509
C 0.508407 14.780352 5.905796
H 0.052179 14.742604 4.936296
C 0.266399 14.051638 7.056487
C -0.772764 13.014609 7.303069
H -1.050781 12.552291 6.347915
H -0.387678 12.254580 7.994396
C -2.962790 12.895227 8.270124
C -3.948048 13.541212 9.011319
H -3.815164 14.585667 9.245459
C -5.072425 12.866727 9.449245
H -5.804115 13.399687 10.035687
C -5.238588 11.519741 9.143045
C -4.250102 10.872919 8.405082
H -4.382823 9.826865 8.177166
C -3.123091 11.545328 7.974734
H -2.379821 11.008116 7.408359
C -7.359730 11.344930 10.259166
H -7.760957 12.212849 9.722750
H -6.979373 11.666019 11.239074
C -8.446704 10.288319 10.425938
H -9.158258 10.646577 11.170051
H -7.976947 9.386606 10.820916
C -9.165738 9.978340 9.102372
H -9.031532 8.924746 8.852808

H -8.708926 10.558772 8.300951
C -10.665162 10.275314 9.150261
H -11.135953 9.865597 8.253344
H -11.098276 9.761430 10.011475
C -11.009224 11.762606 9.248747
H -12.073274 11.857931 9.472924
H -10.449211 12.226571 10.061010
C -10.740521 12.556172 7.970724
H -11.011391 11.963421 7.087516
H -11.341833 13.474643 7.980737
C -8.953501 13.781479 6.947256
C -7.619804 14.190663 7.017387
H -6.986856 13.780274 7.788535
C -7.120014 15.104608 6.116798
H -6.087718 15.411457 6.188776
C -7.925447 15.622379 5.102990
C -9.242212 15.184331 5.016792
H -9.881581 15.577911 4.240927
C -9.759607 14.282396 5.928931
H -10.789796 13.978931 5.834585
N -4.691152 18.228304 0.367888
N -4.028136 19.863116 2.347678
H -4.772185 19.377818 1.847949
N -6.359288 18.795420 3.806785
N -7.052750 17.191553 1.810991
H -6.623394 18.072625 2.090824
N 1.515138 15.606271 6.241659
N 1.870761 15.399289 7.501247
N 1.132580 14.471774 8.001507
O 3.062332 17.594249 0.084849
O -1.898653 13.664469 7.882844
O -6.306107 10.752627 9.511502
O -9.364016 12.909599 7.909922
C 1.534045 12.598225 2.402730
C 1.824854 12.472278 1.046280
H 2.747324 12.026880 0.714972
C 0.907026 12.938424 0.119475
H 1.114572 12.867666 -0.938883
C -0.278860 13.493974 0.563430
H -1.007623 13.875392 -0.133981
C -0.497482 13.575040 1.939688
C -1.765728 14.122263 2.463849
C -2.963319 13.866346 1.797698
H -2.976927 13.256554 0.908046
C -4.136113 14.386534 2.318844
H -5.082775 14.210954 1.829058
C -4.075488 15.132236 3.480214
H -4.968486 15.553474 3.912757
C -2.839502 15.325772 4.095885
C -2.746102 16.123598 5.361426
H -1.776300 15.961372 5.829918
H -3.519413 15.780345 6.052190
C -2.946155 17.615198 5.082275
H -3.910970 17.766351 4.593508
H -2.169548 17.961755 4.398122
C -2.903559 18.449557 6.366767
H -3.206915 19.467818 6.114960
H -3.625280 18.046064 7.079335
C -1.534471 18.478586 6.984286
C -1.219250 17.715091 8.097514
H -1.970215 17.076008 8.538477
C 0.042891 17.748353 8.668549
H 0.241830 17.137883 9.534380
C 1.030940 18.552671 8.114773
C 0.724550 19.321866 6.990542
H 1.498098 19.947485 6.572037
C -0.538205 19.283305 6.439770
H -0.762483 19.886314 5.572909
C 2.700601 17.890971 9.702585
H 2.463709 16.837623 9.521494
H 2.154745 18.231639 10.593414
C 4.201403 18.081962 9.916721
H 4.392717 19.143251 10.083887
H 4.486425 17.532959 10.815014
C 5.046249 17.616944 8.730436
H 6.092175 17.874737 8.906013
H 4.717578 18.137720 7.830193
C 4.939840 16.113195 8.494629
H 5.286372 15.560170 9.378450
H 3.901504 15.842055 8.280952
C 5.490519 14.623918 6.702760
C 4.607560 13.637892 7.128514
H 4.088378 13.722202 8.069068
C 4.371725 12.532596 6.328213
H 3.669141 11.786075 6.670523
C 5.010209 12.367268 5.108736
C 5.929068 13.335587 4.714663
H 6.459575 13.219176 3.780546
C 6.168085 14.447936 5.494191
H 6.871291 15.206532 5.186130
C 4.697114 11.198441 4.218147
H 4.189926 10.422340 4.794152
H 5.626922 10.778879 3.826718
C 3.811667 11.610399 3.032820
H 4.318810 12.397968 2.472875
H 3.686444 10.744149 2.379521
C 2.446173 12.111024 3.494170
H 1.921249 11.311638 4.025775
H 2.575241 12.923337 4.212808
N 0.398666 13.149123 2.823423
N -1.717139 14.834684 3.588520
O 2.308250 18.669100 8.580662
O 5.757805 15.779957 7.376770

Table S 11. DFT-calculated Cartesian coordinates of **12-III** (8.9 kcal/mol with respect to **12-II**)

C -5.084726 16.974467 -1.007320
C -3.844010 16.462489 -1.504024
H -3.711522 15.838383 -2.366162
C -2.874519 16.934475 -0.670411
H -1.814288 16.786489 -0.746111
C -3.541802 17.728747 0.323961
C -2.969580 18.423611 1.379934
C -3.726268 19.284572 2.211159
C -3.376504 20.021381 3.352226
H -2.410673 20.052768 3.816548
C -4.520112 20.681403 3.782756
H -4.587956 21.332417 4.631540
C -5.557766 20.342303 2.913267
C -7.014026 20.719298 2.934186
C -7.458328 21.151127 1.525687
H -7.364788 20.326407 0.826100
H -6.849397 21.983497 1.179609
H -8.500448 21.462800 1.548846
C -7.259591 21.865494 3.916601
H -8.311919 22.140308 3.913809
H -6.675155 22.734538 3.622824
H -6.977135 21.580759 4.926754
C -7.771678 19.474832 3.333481
C -8.605891 19.287974 4.475394
H -8.874524 20.031309 5.200980
C -8.998323 17.980058 4.446171
H -9.675829 17.480004 5.111375
C -8.406331 17.397721 3.279600
C -8.541531 16.081313 2.843382
C -8.145339 15.688914 1.542325
C -8.241350 14.473200 0.848562
H -8.667519 13.559992 1.210968
C -7.669512 14.668724 -0.401227
H -7.591506 13.931716 -1.175411

C -7.221258 15.988000 -0.464316
C -6.470977 16.715910 -1.546512
C -7.160068 18.060684 -1.837907
H -8.187235 17.891844 -2.153537
H -6.628452 18.583048 -2.630549
H -7.159946 18.688866 -0.952886
C -6.427549 15.880979 -2.826873
H -5.942874 14.924256 -2.650986
H -5.881027 16.413434 -3.602166
H -7.439598 15.697751 -3.180450
C -1.540048 18.248873 1.662983
C -0.995278 16.966831 1.764197
H -1.639631 16.108341 1.653573
C 0.341866 16.785959 2.033166
H 0.764246 15.796312 2.113696
C 1.184289 17.885576 2.213518
C 0.651461 19.167582 2.117583
H 1.278023 20.036672 2.241945
C -0.693691 19.339043 1.842634
H -1.088258 20.338393 1.739004
C 3.349052 18.612575 2.955937
H 2.890952 19.091656 3.829838
H 3.509899 19.375772 2.183382
C 4.687220 17.969093 3.324547
H 5.299449 18.720662 3.824670
H 5.190510 17.674119 2.402193
C 4.538377 16.727147 4.206093
H 3.860221 16.034658 3.705004
H 5.512464 16.240489 4.284385
C 4.013851 17.015500 5.612083
H 3.092457 17.596491 5.552446
H 4.748831 17.602332 6.166290
C 3.723977 15.711934 6.353126
H 2.947085 15.163495 5.814665
H 4.619031 15.087689 6.374941
C 3.253204 15.958246 7.790153
H 4.079152 16.319345 8.404886
H 2.440315 16.688148 7.802630
C 1.549654 14.188969 8.254528
H 0.741128 14.657274 7.735884
C 1.635448 12.992852 8.940552
C 0.584380 11.957248 9.145966
H 0.361069 11.465888 8.188355
H 0.943086 11.208658 9.862571
C -1.775747 11.964192 9.612913
C -2.879991 12.726149 9.981927
H -2.723776 13.751085 10.279698
C -4.153315 12.191425 9.960399
H -4.979810 12.813308 10.261286
C -4.350509 10.874976 9.553370
C -3.240006 10.103836 9.218609
H -3.395239 9.076709 8.927577
C -1.963860 10.634726 9.252211
H -1.129168 10.002222 8.995436
C -6.736909 11.053816 9.614654
H -6.676146 11.936079 8.965941
H -6.819704 11.379706 10.660656
C -7.952138 10.217972 9.227743
H -8.841639 10.799121 9.471080
H -7.956270 9.304279 9.825083
C -7.945848 9.863107 7.741754
H -7.041355 9.288205 7.531135
H -7.904787 10.779864 7.152732
C -9.166794 9.043319 7.322481
H -8.978675 8.611998 6.336454
H -9.295994 8.216685 8.025082
C -10.473912 9.837705 7.260719
H -11.297445 9.137528 7.109233
H -10.644704 10.361335 8.200950
C -10.527853 10.861871 6.128241
H -10.083585 10.447771 5.213336
H -11.571124 11.136154 5.924723
C -9.626449 13.010727 5.598293
C -8.774750 14.050953 5.971152
H -8.335969 14.041380 6.956230
C -8.476813 15.057126 5.082010
H -7.784444 15.830959 5.374958
C -9.010522 15.068056 3.788884
C -9.903161 14.057776 3.451893
H -10.379657 14.080045 2.486925
C -10.212467 13.046493 4.338805
H -10.915820 12.287722 4.047527
N -4.900305 17.727422 0.055374
N -5.056384 19.514932 1.986970
H -5.534639 18.997365 1.249365
N -7.680911 18.370244 2.618626
N -7.531495 16.576903 0.697736
H -7.298277 17.510624 1.038975
N 2.773565 14.737953 8.380812
N 3.558109 13.935386 9.083402
N 2.889796 12.892802 9.428983
O 2.487809 17.593764 2.468479
O -0.570818 12.620162 9.635699
O -5.567676 10.261483 9.454795
O -9.814861 12.027540 6.517218
C -10.715805 10.725280 1.881466
C -10.763958 9.365232 2.174589
H -11.706647 8.843519 2.207348
C -9.579034 8.691158 2.419306
H -9.587869 7.636759 2.656443
C -8.382729 9.384374 2.351558
H -7.437479 8.899464 2.540461
C -8.422691 10.739449 2.032430
C -7.153243 11.495380 1.866844
C -6.364032 11.227963 0.751982
H -6.672780 10.483101 0.034541
C -5.198351 11.955640 0.581562
H -4.566458 11.786869 -0.278780
C -4.863940 12.905469 1.528177
H -3.971958 13.502727 1.421772
C -5.701555 13.093311 2.626058
C -5.355277 14.103091 3.680535
H -6.253156 14.337293 4.249979
H -5.000314 15.012729 3.190429
C -4.267840 13.582339 4.629274
H -3.438352 13.178274 4.043464
H -4.681213 12.769951 5.229765
C -3.723355 14.682785 5.552621
H -2.936896 14.247886 6.175023
H -3.282118 15.473916 4.942951
C -4.790153 15.265582 6.435368
C -5.329341 16.518217 6.187151
H -4.950533 17.112443 5.368412
C -6.367720 17.025713 6.953774
H -6.767810 17.999852 6.718059
C -6.891546 16.269177 7.995274
C -6.309482 15.036330 8.292638
H -6.703156 14.477403 9.128261
C -5.279160 14.544351 7.520320
H -4.849627 13.581499 7.755104
C -8.821432 17.654755 8.288421
H -8.953355 17.551105 7.207477
H -8.375787 18.637459 8.496506
C -10.166249 17.540217 9.004318
H -9.986493 17.628533 10.076825
H -10.791209 18.377028 8.688677
C -10.890422 16.218251 8.748239
H -11.733284 16.137513 9.437291
H -10.212889 15.387997 8.950442
C -11.446101 16.062292 7.332629
H -12.036923 16.945630 7.050420
H -10.639714 15.915660 6.604224

C -12.740092 14.379493 6.183413
C -12.641800 14.966548 4.929757
H -12.182179 15.934612 4.805948
C -13.122108 14.297691 3.812480
H -13.029677 14.773386 2.846925
C -13.702914 13.043645 3.910456
C -13.840141 12.487506 5.180968
H -14.318104 11.524201 5.292735
C -13.372072 13.140012 6.300208
H -13.469908 12.707361 7.284160
C -14.148921 12.259408 2.705539
H -14.217238 12.909707 1.832766
H -15.143172 11.851234 2.901944
C -13.190435 11.090887 2.406997
H -12.882528 10.648260 3.356724
H -13.726509 10.326115 1.842069
C -11.952825 11.540719 1.620169
H -12.165812 11.503681 0.547704
H -11.717401 12.576464 1.860276
N -9.560680 11.383013 1.805717
N -6.819458 12.394870 2.785702
O -7.961941 16.630631 8.766510
O -12.277835 14.911798 7.354541

Table S 12. DFT-calculated Cartesian coordinates of **[12-MeH$_3$]$^{4+}$-I** (E = 10.6 kcal/mol with respect to **[12-MeH$_3$]$^{4+}$-II**).

N 3.315704 -9.008672 4.387111
O 0.107377 -3.525258 -0.471293
C 2.583790 -8.643083 6.582895
C 2.663348 -8.230919 5.257667
C 3.901472 -10.175236 4.714735
C 3.824131 -10.620662 6.027311
C 3.160134 -9.843210 6.965694
C 2.060430 -6.949235 4.791538
C 0.548668 -6.883684 5.060887
C -0.017995 -5.471461 4.856144
C 0.003803 -5.001964 3.427526
C -1.104627 -5.162558 2.608985
C -1.121760 -4.702239 1.301182
C -0.006632 -4.052924 0.785872
C 1.115754 -3.891803 1.599663
C 1.118004 -4.360179 2.895752
C -1.006265 -3.521714 -1.349914
C 4.646117 -10.876510 3.662281
C 4.957277 -12.230711 3.748448
C -1.138624 -4.816881 -2.158268
N 5.027941 -10.110928 2.633369
C 5.770540 -10.641207 1.674001
C 6.137061 -11.990278 1.687843
C 5.713541 -12.793409 2.730035
C 6.178046 -9.750582 0.540307
C 5.051775 -9.683640 -0.499060
C 5.473586 -8.902344 -1.745065
C 4.318769 -8.339633 -2.530204
C 3.052661 -8.910678 -2.514440
C 1.992230 -8.327211 -3.187240
C 2.183056 -7.157380 -3.912532
C 3.463026 -6.616539 -3.993951
C 4.509263 -7.203163 -3.309589
O 1.179139 -6.529714 -4.620000
C -0.038529 -6.275806 -3.933281
C 0.111848 -5.074666 -2.995184
H 2.087389 -8.023377 7.311046
H 4.297970 -11.540796 6.322242
H 3.106042 -10.168744 7.992713
H 2.247039 -6.803026 3.729521
H 2.534549 -6.135369 5.345507
H 0.343239 -7.176425 6.090278
H 0.027400 -7.586110 4.409260
H 0.546866 -4.771590 5.475058
H -1.049588 -5.466673 5.213561
H -1.986692 -5.642389 3.006716
H -2.015113 -4.824326 0.709695
H 1.967702 -3.373056 1.191712
H 1.987332 -4.199602 3.514337
H -1.927693 -3.305655 -0.800887
H -0.812868 -2.688477 -2.031690
H 4.610054 -12.839406 4.566029
H -2.004122 -4.716564 -2.814022
H -1.307722 -5.659889 -1.486306
H 6.742863 -12.393768 0.892017
H 5.972983 -13.840760 2.757707
H 7.084622 -10.141695 0.077933
H 6.375788 -8.747341 0.918679
H 4.197401 -9.196951 -0.033750
H 4.758564 -10.698007 -0.776110
H 6.067688 -9.548951 -2.397684
H 6.120131 -8.073173 -1.447885
H 2.883911 -9.839068 -1.990770
H 1.023780 -8.804917 -3.168660
H 3.612683 -5.742322 -4.608020
H 5.502013 -6.783141 -3.387145
H -0.369095 -7.161117 -3.380009
H -0.766876 -6.064443 -4.719809
H 0.340023 -4.194480 -3.599236
H 0.961416 -5.252435 -2.333148
C -2.196003 -8.445716 0.945334
C -3.273887 -9.427159 0.487356
C -4.689990 -9.015482 0.908316
O -4.547684 -3.790856 0.043687
C -4.454229 -2.411389 0.075661
C -3.591847 -1.887272 1.030708
C -3.388664 -0.523591 1.128898
C -4.051522 0.343221 0.264074
C -4.926621 -0.189119 -0.681922
C -5.130767 -1.551438 -0.779924
O -3.913825 1.697939 0.249273
C -3.019742 2.309119 1.166587
C -2.922337 3.791314 0.818148
C -1.941972 4.510207 1.742492
C -1.632424 5.929183 1.267987
C -0.590504 6.596000 2.169957
C 0.098870 7.785693 1.494445
C -5.524225 -4.375193 -0.792821
C -0.795508 -8.978926 0.644097
N -6.062096 -6.772588 -1.343202
C -5.483339 -5.841062 -0.548683
C -4.877641 -6.561741 0.459199
N -5.137657 -7.854217 0.188133
N -5.847225 -7.971156 -0.889688
O 2.710096 -7.898636 1.570070
C 4.312232 -6.220633 1.766206
C 3.252930 -6.783223 1.037646
C 2.844473 -6.164319 -0.144512
N 5.191268 -1.440579 -1.969673
N 6.417781 -1.247427 0.608318
C 4.901627 -5.053715 1.354317
C 3.446846 -4.999522 -0.561074
C 4.469775 -4.398701 0.187397
C 5.037528 -3.129697 -0.198763
C 5.486659 -2.244656 0.813186
C 5.097461 -2.752389 -1.554152
C 5.092458 -3.534133 -2.724437
C 5.163359 -2.674236 -3.808292
C 5.186611 -1.370180 -3.312470
C 5.054700 -2.118200 2.142052
C 5.744991 -1.057339 2.711880
C 6.621199 -0.556886 1.751965
C 4.613979 -0.169303 -5.423393
C 8.076695 0.719038 3.308744

C 5.243346 -0.047696 -4.030181
C 7.599838 0.582398 1.857049
C 6.922600 1.853870 1.413536
C 7.167186 3.176162 1.774246
C 6.257678 3.974563 1.092748
C 4.481192 0.963557 -3.213541
C 3.121114 1.254197 -3.227759
C 2.889442 2.243826 -2.280000
C 4.112579 2.534504 -1.661097
C 5.471567 3.135161 0.288184
C 4.400398 3.421727 -0.590669
C 3.549525 4.553860 -0.371652
C 3.340445 5.065534 0.922285
C 2.413530 6.049740 1.162575
C 1.648909 6.572640 0.108874
C 1.929815 6.143299 -1.202677
C 2.845978 5.155029 -1.436100
N 5.942100 1.854693 0.489264
N 5.051151 1.700412 -2.232862
O 0.650280 7.452250 0.224157
C 6.722472 0.361677 -4.174094
C 8.817573 0.341226 0.941952
C 0.291062 -8.035500 1.156625
C 1.680205 -8.614684 0.894445
H -2.288064 -8.288528 2.021342
H -2.320009 -7.482431 0.448636
H -3.079862 -10.403637 0.931954
H -3.239942 -9.549109 -0.596525
H -4.717807 -8.796540 1.976881
H -5.386507 -9.823748 0.681714
H -3.081066 -2.558385 1.702878
H -2.722699 -0.151796 1.889780
H -5.445814 0.490212 -1.338927
H -5.819563 -1.912504 -1.525824
H -2.028590 1.843927 1.082119
H -3.389521 2.180032 2.192310
H -3.913151 4.241287 0.888978
H -2.593227 3.880486 -0.219090
H -1.009478 3.940304 1.779081
H -2.351066 4.544343 2.754008
H -2.545780 6.525074 1.248781
H -1.249760 5.884398 0.245960
H 0.155235 5.850150 2.448170
H -1.057117 6.958561 3.086052
H 0.869037 8.210500 2.147217
H -0.630572 8.564922 1.263663
H -5.312840 -4.164508 -1.851380
H -6.525635 -3.984018 -0.557211
H -0.681989 -9.123693 -0.431953
H -0.675679 -9.956084 1.116600
H -4.325635 -6.222158 1.309268
H 4.669229 -6.729799 2.646622
H 2.049044 -6.581269 -0.738251
H 5.733692 -4.654718 1.912394
H 3.077694 -4.507364 -1.446062
H 5.084095 -4.603841 -2.759198
H 5.192169 -2.956877 -4.840681
H 4.283319 -2.698832 2.604911
H 5.631656 -0.695783 3.713156
H 5.222425 -0.805633 -6.059404
H 4.551202 0.810074 -5.890804
H 3.615461 -0.593138 -5.368962
H 8.874441 1.452504 3.376595
H 8.463406 -0.231710 3.666684
H 7.267588 1.037038 3.959980
H 7.915929 3.520963 2.457754
H 6.203244 5.044337 1.123607
H 2.383792 0.808591 -3.863408
H 1.941212 2.670013 -2.021922
H 3.869263 4.633063 1.756975
H 2.271446 6.405062 2.169946
H 1.387659 6.607712 -2.011334
H 3.054296 4.855735 -2.451074
H 6.793207 1.352490 -4.616563
H 7.230231 -0.334821 -4.836521
H 7.248149 0.352656 -3.222826
H 9.554273 1.123336 1.105705
H 8.552281 0.360533 -0.111933
H 9.283666 -0.611643 1.181639
H 0.165805 -7.890432 2.229908
H 0.197195 -7.056657 0.683811
H 1.884213 -8.662956 -0.178247
H 1.740141 -9.627460 1.302980
H 5.068411 -0.644109 -1.365150
H 5.461848 1.029701 0.167834
C -6.836268 -6.559292 -2.527630
H -6.238840 -6.053968 -3.286842
H -7.711755 -5.949056 -2.305346
H -7.157809 -7.527927 -2.904641
H 7.036227 -1.215421 -0.188068
H 6.045630 1.829485 -2.122589
H 3.412284 -8.717004 3.406641

Table S 13. DFT-calculated Cartesian coordinates of **[12-MeH$_3$]$^{4+}$-II** (*E* = 0.0 kcal/mol)

N -6.156944 3.493791 -0.301271
O -0.541654 0.159263 4.235878
C -7.930088 4.364833 0.962760
C -6.781407 3.585362 0.877076
C -6.569464 4.121528 -1.418251
C -7.708713 4.912350 -1.362876
C -8.392804 5.029742 -0.160404
C -6.230731 2.871319 2.063617
C -5.726824 3.861260 3.128427
C -5.355807 3.159529 4.442919
C -4.068595 2.386149 4.367284
C -2.859780 3.000957 4.665492
C -1.661187 2.306198 4.634472
C -1.653826 0.957379 4.300751
C -2.861890 0.332277 3.989118
C -4.047594 1.037892 4.022919
C 0.657391 0.550833 4.888495
C -5.799344 3.891398 -2.648092
C -5.864486 4.755943 -3.738647
C 1.629249 1.306047 3.976483
N -5.043989 2.789750 -2.650484
C -4.348031 2.477275 -3.732914
C -4.363964 3.284072 -4.873213
C -5.124826 4.439025 -4.868776
C -3.511005 1.235438 -3.669493
C -2.196587 1.525270 -2.933400
C -1.364285 0.257071 -2.767050
C -0.144250 0.395439 -1.894846
C 0.224735 1.584629 -1.282414
C 1.322456 1.650317 -0.440882
C 2.085231 0.517536 -0.200398
C 1.747321 -0.675185 -0.830420
C 0.647204 -0.727631 -1.662918
O 3.208713 0.526574 0.617739
C 3.042985 1.171945 1.880079
C 1.994737 0.477100 2.747622
H -8.458673 4.438798 1.898792
H -8.075198 5.409157 -2.244480
H -9.286579 5.631311 -0.106011
H -5.422290 2.205948 1.771650
H -7.029656 2.275661 2.509920
H -6.507319 4.591873 3.342714
H -4.856187 4.400741 2.753695
H -6.167139 2.491863 4.737891

H -5.257155 3.922262 5.217822
H -2.849522 4.043775 4.949045
H -0.749583 2.822001 4.892096
H -2.849052 -0.718625 3.747257
H -4.971372 0.523168 3.805032
H 0.431190 1.140300 5.782873
H 1.115391 -0.391241 5.206493
H -6.451800 5.657860 -3.709591
H 2.522374 1.530493 4.561681
H 1.182011 2.249984 3.660550
H -3.790520 3.006202 -5.743438
H -5.148087 5.085422 -5.733068
H -3.306336 0.876390 -4.678508
H -4.056401 0.468146 -3.119291
H -2.453982 1.931685 -1.957583
H -1.630655 2.277096 -3.487706
H -1.052482 -0.101873 -3.752188
H -1.997755 -0.521726 -2.331227
H -0.335320 2.487894 -1.459488
H 1.577854 2.592093 0.019537
H 2.334587 -1.558876 -0.637184
H 0.384244 -1.663384 -2.133462
H 2.780389 2.225197 1.736528
H 4.022831 1.118893 2.357008
H 2.374070 -0.496980 3.059137
H 1.093009 0.307126 2.155211
C -2.560968 -8.422432 0.857927
C -3.685064 -9.353424 0.407014
C -5.077513 -8.872964 0.834071
O -4.623491 -3.675689 -0.207834
C -4.461633 -2.308524 -0.178751
C -3.355044 -1.849478 0.528194
C -3.094171 -0.497416 0.640583
C -3.943529 0.423051 0.038274
C -5.043642 -0.036613 -0.680903
C -5.304030 -1.390167 -0.792693
O -3.789535 1.784027 0.095484
C -2.678117 2.328349 0.797173
C -2.622002 3.828674 0.522933
C -1.511883 4.475304 1.347144
C -1.272240 5.938383 0.974429
C -0.212689 6.561942 1.887872
C 0.470772 7.783959 1.266023
C -5.740139 -4.210109 -0.888098
C -1.188686 -9.005783 0.519564
N -6.342133 -6.593058 -1.442097
C -5.741541 -5.678299 -0.644528
C -5.172971 -6.415057 0.373742
N -5.474655 -7.698862 0.105218
N -6.175296 -7.795748 -0.980320
O 2.363864 -8.056237 1.411018
C 3.873015 -6.326070 1.744306
C 2.845952 -6.888665 0.965506
C 2.427137 -6.208683 -0.185064
N 4.917966 -1.576914 -1.971629
N 6.080767 -1.360036 0.655852
C 4.445159 -5.131566 1.397603
C 3.019841 -5.019710 -0.540432
C 4.033688 -4.443280 0.240475
C 4.640991 -3.192952 -0.135173
C 5.110175 -2.314624 0.867991
C 4.777504 -2.866398 -1.503019
C 4.880162 -3.711434 -2.620651
C 5.069709 -2.910159 -3.736579
C 5.052694 -1.582955 -3.312177
C 4.717933 -2.192960 2.210028
C 5.477842 -1.182270 2.780450
C 6.349550 -0.704307 1.803928
C 4.623269 -0.502803 -5.514520
C 7.786924 0.582211 3.371193
C 5.233973 -0.324140 -4.118556
C 7.409165 0.359796 1.900675
C 6.900585 1.646381 1.303703
C 7.314735 2.953001 1.546926
C 6.513223 3.796897 0.788521
C 4.564739 0.823857 -3.415038
C 3.303684 1.375055 -3.632148
C 3.142169 2.422730 -2.740912
C 4.304372 2.499299 -1.953260
C 5.635913 2.993282 0.047429
C 4.628664 3.357418 -0.880915
C 3.872848 4.563204 -0.668730
C 3.661025 5.038139 0.637264
C 2.768280 6.050625 0.897084
C 2.038088 6.629883 -0.148734
C 2.351932 6.260348 -1.469100
C 3.245482 5.253542 -1.723163
N 5.938224 1.683420 0.357829
N 5.130473 1.482254 -2.383617
O 1.037506 7.511750 -0.012494
C 6.747730 -0.058985 -4.258466
C 8.667164 -0.072509 1.117700
C -0.059991 -8.139425 1.074733
C 1.309741 -8.730062 0.734283
H -2.623444 -8.279120 1.938651
H -2.655052 -7.447419 0.375660
H -3.532876 -10.335970 0.853958
H -3.659749 -9.479230 -0.676628
H -5.084664 -8.643795 1.900742
H -5.811541 -9.650769 0.619614
H -2.703623 -2.569755 0.996480
H -2.238328 -0.178816 1.210489
H -5.688723 0.676168 -1.168318
H -6.165639 -1.701755 -1.360192
H -1.751599 1.854840 0.454177
H -2.789945 2.142980 1.871465
H -3.577382 4.285353 0.779616
H -2.445596 3.998891 -0.540926
H -0.585953 3.916569 1.193800
H -1.767403 4.405435 2.405970
H -2.201655 6.504906 1.047687
H -0.933574 5.995798 -0.062435
H 0.537441 5.804009 2.115908
H -0.660873 6.879112 2.829833
H 1.230841 8.188237 1.943410
H -0.262263 8.567632 1.062460
H -5.675134 -4.005292 -1.966132
H -6.676136 -3.776339 -0.503759
H -1.083861 -9.095593 -0.563841
H -1.107377 -10.009252 0.940790
H -4.612243 -6.095407 1.226373
H 4.203188 -6.872715 2.613405
H 1.638767 -6.606211 -0.802743
H 5.258391 -4.743761 1.990186
H 2.663040 -4.497598 -1.414535
H 4.881453 -4.782683 -2.597394
H 5.205939 -3.253221 -4.741687
H 3.937397 -2.752197 2.685223
H 5.416443 -0.845983 3.795264
H 5.186708 -1.236898 -6.082376
H 4.655913 0.437420 -6.058742
H 3.592011 -0.837722 -5.450299
H 8.636163 1.254475 3.446123
H 8.066908 -0.361692 3.831624
H 6.961345 1.015000 3.929479
H 8.101954 3.259093 2.205126
H 6.591252 4.864087 0.725685
H 2.590794 1.055924 -4.363351
H 2.271190 3.034365 -2.638466
H 4.161835 4.560073 1.464717
H 2.630221 6.381535 1.913436
H 1.857101 6.784468 -2.271597

H 3.490825 5.006059 -2.743423
H 6.914068 0.885709 -4.770656
H 7.203055 -0.847248 -4.852141
H 7.254114 -0.038291 -3.297165
H 9.462816 0.649806 1.279501
H 8.488113 -0.129294 0.047314
H 9.013680 -1.039535 1.474357
H -0.142069 -8.080558 2.162281
H -0.137435 -7.125164 0.677885
H 1.486521 -8.739878 -0.346083
H 1.370578 -9.757478 1.102133
H 4.699064 -0.759916 -1.421217
H 5.295985 0.925106 0.168414
C -7.094430 -6.365069 -2.638531
H -6.472819 -5.880928 -3.392026
H -7.959082 -5.734884 -2.430756
H -7.432581 -7.328262 -3.014838
H 6.667586 -1.334133 -0.163713
H 6.098558 1.400651 -2.113258
H -5.319348 2.897495 -0.388864

Table S 14. DFT-calculated Cartesian coordinates of **[12-MeH$_3$]$^{4+}$-III** (E = 14.7 kcal/mol with respect to **[12-MeH$_3$]$^{4+}$-II**).

N 4.723136 6.387126 2.999821
O 0.426948 1.365949 -0.691207
C 5.285595 6.097068 5.255337
C 4.506287 5.753399 4.157466
C 5.649572 7.348687 2.824239
C 6.450220 7.704020 3.902419
C 6.260049 7.073737 5.124353
C 3.480490 4.675359 4.240480
C 4.144750 3.287311 4.177761
C 3.104844 2.158720 4.248840
C 2.353886 1.964534 2.959939
C 2.918110 1.222524 1.925934
C 2.245566 1.028629 0.738211
C 0.977027 1.579901 0.542719
C 0.395297 2.297218 1.578525
C 1.083567 2.485734 2.766954
C -0.934880 1.681666 -0.947783
C 5.748416 7.946347 1.486125
C 6.534621 9.066959 1.236101
C -1.172822 1.560776 -2.455966
N 5.024264 7.345375 0.526552
C 5.062067 7.823507 -0.708288
C 5.843494 8.936261 -1.042383
C 6.580455 9.564910 -0.059317
C 4.246373 7.156095 -1.775836
C 5.123801 6.335550 -2.742302
C 4.686312 6.438207 -4.213504
C 3.369400 5.776127 -4.513814
C 3.304165 4.413727 -4.781459
C 2.099364 3.778925 -5.036159
C 0.914489 4.508923 -5.031355
C 0.976379 5.884754 -4.816211
C 2.185413 6.503469 -4.561787
O -0.329204 3.973272 -5.278287
C -0.655250 2.718345 -4.692525
C -0.779533 2.852169 -3.172473
H 5.126085 5.607482 6.201912
H 7.214291 8.454413 3.801657
H 6.871952 7.346761 5.969751
H 2.749253 4.758162 3.440961
H 2.958764 4.759654 5.195114
H 4.829390 3.186031 5.020392
H 4.722246 3.191604 3.257395
H 2.403624 2.364153 5.058565
H 3.622418 1.229495 4.493180
H 3.884694 0.767102 2.078213
H 2.658273 0.427697 -0.055832
H -0.596610 2.704566 1.481487
H 0.600508 3.031486 3.563795
H -1.576579 0.980531 -0.398952
H -1.161636 2.703633 -0.626452
H 7.087338 9.559884 2.016656
H -2.224979 1.341316 -2.634063
H -0.584135 0.721665 -2.830397
H 5.854183 9.300211 -2.057323
H 7.178070 10.433885 -0.288262
H 3.739459 7.939907 -2.339961
H 3.490098 6.522673 -1.322086
H 5.115519 5.289610 -2.435543
H 6.153387 6.695518 -2.684505
H 5.460677 5.972963 -4.828406
H 4.633485 7.490547 -4.497675
H 4.215046 3.834002 -4.802793
H 2.087507 2.722756 -5.254020
H 0.060978 6.454960 -4.863205
H 2.200927 7.571965 -4.406180
H 0.081408 1.953381 -4.960612
H -1.613865 2.449444 -5.141800
H -1.521625 3.625155 -2.960593
H 0.178749 3.197741 -2.786264
C -1.964289 -7.999507 0.641587
C -3.077518 -8.920988 0.143883
C -4.474942 -8.518258 0.629610
O -4.782950 -3.231883 0.076340
C -4.819786 -1.857805 0.178283
C -3.972915 -1.307684 1.132799
C -3.911100 0.059534 1.323284
C -4.700291 0.908268 0.550565
C -5.553814 0.350648 -0.399103
C -5.617952 -1.017137 -0.587905
O -4.698650 2.271265 0.636061
C -3.743649 2.881356 1.489199
C -3.724525 4.389380 1.240484
C -2.418828 4.991517 1.760856
C -2.445358 6.516445 1.849670
C -1.082462 7.074570 2.291109
C -0.263218 7.642090 1.128293
C -5.640474 -3.864464 -0.848460
C -0.589715 -8.573140 0.291803
N -5.966341 -6.261540 -1.528797
C -5.459729 -5.331852 -0.684706
C -4.801902 -6.056996 0.287437
N -4.969198 -7.349679 -0.048549
N -5.666194 -7.462733 -1.135385
O 2.964399 -7.771261 1.318642
C 4.562307 -6.119393 1.647248
C 3.537981 -6.658784 0.846468
C 3.209079 -6.007746 -0.349671
N 5.730237 -1.426599 -2.092318
N 6.716112 -1.069795 0.538671
C 5.203006 -4.964427 1.287129
C 3.873617 -4.863254 -0.719812
C 4.871853 -4.298492 0.091326
C 5.493048 -3.054120 -0.273405
C 5.854329 -2.130102 0.737645
C 5.652697 -2.723479 -1.638477
C 5.747425 -3.541447 -2.778015
C 5.854095 -2.713542 -3.888211
C 5.804503 -1.397579 -3.436671
C 5.342190 -1.988645 2.036318
C 5.913259 -0.851192 2.593120
C 6.790767 -0.312609 1.656116
C 5.414659 -0.263493 -5.632465
C 7.999617 1.233927 3.181862
C 5.811943 -0.076740 -4.164518
C 7.601184 0.954669 1.727147
C 6.761232 2.089585 1.194054
C 6.828449 3.455569 1.446455

C 5.805716 4.070240 0.727095
C 4.799797 0.784883 -3.455223
C 3.409907 0.736989 -3.545037
C 2.893999 1.641699 -2.630077
C 3.974608 2.223064 -1.947807
C 5.135349 3.073403 0.011875
C 4.013435 3.130134 -0.863537
C 2.952276 4.056312 -0.609498
C 2.767542 4.615286 0.667736
C 1.766657 5.522875 0.923487
C 0.876932 5.896953 -0.094272
C 1.037872 5.322329 -1.367787
C 2.053230 4.440668 -1.621972
N 5.778606 1.889876 0.292074
N 5.118465 1.642678 -2.464850
O -0.132587 6.764490 0.015503
C 7.225111 0.527775 -4.086419
C 8.871671 0.842155 0.862080
C 0.549053 -7.753245 0.896231
C 1.902595 -8.414578 0.623126
H -2.036296 -7.902221 1.726745
H -2.062391 -7.005209 0.201473
H -2.890311 -9.928497 0.516189
H -3.071089 -8.969087 -0.946026
H -4.457227 -8.318244 1.701846
H -5.174068 -9.328932 0.419053
H -3.366624 -1.968002 1.732158
H -3.257528 0.443205 2.089316
H -6.170672 1.011994 -0.986580
H -6.296210 -1.398816 -1.333383
H -2.755925 2.460069 1.266035
H -3.988315 2.670882 2.538661
H -4.580657 4.850252 1.734151
H -3.827154 4.569581 0.168948
H -1.603288 4.691513 1.099403
H -2.222043 4.588198 2.757789
H -3.215034 6.814068 2.562761
H -2.720424 6.934816 0.880300
H -0.526777 6.288844 2.804159
H -1.223503 7.895186 2.994941
H 0.724675 7.966902 1.471422
H -0.788134 8.508628 0.716368
H -5.393571 -3.566128 -1.878511
H -6.691654 -3.600772 -0.654014
H -0.474944 -8.616755 -0.793301
H -0.525067 -9.595554 0.668810
H -4.267821 -5.723223 1.151537
H 4.827601 -6.648622 2.548742
H 2.427443 -6.388361 -0.986306
H 6.005357 -4.590058 1.903150
H 3.575885 -4.356637 -1.624552
H 5.787411 -4.612243 -2.778377
H 5.954084 -3.033272 -4.905376
H 4.596576 -2.612405 2.485911
H 5.725513 -0.473485 3.577081
H 6.173388 -0.836663 -6.157712
H 5.323719 0.702202 -6.123362
H 4.468248 -0.788352 -5.722226
H 8.672262 2.084799 3.232937
H 8.517114 0.373431 3.598216
H 7.128048 1.445803 3.793398
H 7.540815 3.944805 2.078994
H 5.613207 5.121465 0.645884
H 2.845392 0.110846 -4.205609
H 1.860252 1.790458 -2.402753
H 3.397847 4.259354 1.463595
H 1.629775 5.929092 1.912552
H 0.364539 5.632710 -2.150004
H 2.210329 4.094151 -2.627590
H 7.236230 1.517452 -4.536978
H 7.916512 -0.097063 -4.646433
H 7.591568 0.588106 -3.064900
H 9.487975 1.726491 1.001730
H 8.643698 0.766551 -0.198137
H 9.457373 -0.022354 1.165959
H 0.418756 -7.684000 1.978331
H 0.539014 -6.739217 0.491501
H 2.114956 -8.465634 -0.449312
H 1.901231 -9.432331 1.021781
H 5.561900 -0.614313 -1.521489
H 5.400561 0.987775 0.051562
C -6.749494 -6.044642 -2.706714
H -6.191669 -5.448410 -3.429024
H -7.673645 -5.522816 -2.457662
H -6.987343 -7.013361 -3.141015
H 7.385532 -1.031056 -0.214989
H 6.050769 1.984667 -2.286705
H 4.172434 6.163262 2.165050

References

[1] Rigaku OD (2020). CrysAlis PRO. Rigaku Oxford Diffraction Ltd, Yarnton, Oxfordshire, England.
[2] G. M. Sheldrick, *Acta Crystallogr. A Found. Adv.* **2015**, 71, 3–8.
[3] G. M. Sheldrick, *Acta Crystallogr. C Struct. Chem.* **2015**, 71, 3–8.
[4] P. Van Der Sluis, A. L. *Spek, Acta Crystallogr. A Found. Crystallogr.* **1990**, 46, 194–201.
[5] O. V. Dolomanov, L. J. Bourhis, R. J. Gildea, J. A. K. Howard, H. Puschmann, *J. Appl. Crystallogr.* **2009**, 42, 339–341.
[6] A. D. Becke, *J. Chem. Phys.* **1992**, 96, 2155–2160.
[7] C. Lee, W. Yang, R. G. Parr, *Phys. Rev. B* **1988**, 37, 785–789.
[8] S. Grimme, J. Antony, S. Ehrlich, H. Krieg, *J. Chem. Phys.* **2010**, 132, 154104.
[9] R. Ditchfield, W. J. Hehre, J. A. Pople, *J. Chem. Phys.* **1971**, 54, 724–728.
[10] W. J. Hehre, R. Ditchfield, J. A. Pople, *J. Chem. Phys.* **1972**, 56, 2257–2261.
[11] J. Tomasi, B. Mennucci, R. Cammi, *Chem. Rev.* **2005**, 105, 2999–3094.
[12] A. D. Bochevarov, E. Harder, T. F. Hughes, J. R. Greenwood, D. A. Braden, D. M. Philipp, D. Rinaldo, M. D. Halls, J. Zhang, R. A. Friesner, *Int. J. of Quantum Chem.* **2013**, 113, 2110–2142.
[13] C. Bannwarth, S. Ehlert, S. Grimme, *J. Chem. Theory Comput.* **2019**, 15, 1652–1671.
[14] P. Pracht, F. Bohle, S. Grimme, *Phys. Chem. Chem. Phys.* **2020**, 22, 7169–7192.
[15] P. M. Zimmerman, *J. Chem. Phys.* **2013**, 138, 184102.
[16] J. Winn, A. Pinczewska, S. M. Goldup, *J. Am. Chem. Soc.* **2013**, 135, 13318–13321.
[17] J. E. M. Lewis, R. J. Bordoli, M. Denis, C. J. Fletcher, M. Galli, E. A. Neal, E. M. Rochette, S. M. Goldup, *Chem. Sci.* **2016**, 7, 3154–3161.
[18] I. Pryjomska-Ray, D. Zornik, M. Pätzel, K. B. Krause, L. Grubert, B. Braun-Cula, S. Hecht, C. Limberg, *Chem. Eur. J.* **2018**, 24, 5341–5349.
[19] J.-B. Giguère, D. Thibeault, F. Cronier, J.-S. Marois, M. Auger, J.-F. Morin, *Tetrahedron Lett.* **2009**, 50, 5497–5500.
[20] T.-W. Wang, P.-R. Huang, J. L. Chow, W. Kaminsky, M. R. Golder, *J. Am. Chem. Soc.* **2021**, 143, 7314–7319.
[21] Babeș-Bolyai University, Faculty of Chemistry and Chemical Engineering, Supramolecular Organic and Organometallic Chemistry Centre, 11 Arany Janos Str., RO-400028-Cluj-Napoca, Roumania et al. Click synthesis and complexation properties of a new unsymmetrical macrocycle bearing 1,4-dioxabenzene and triazole units. *Rev. Roum. Chim.* **2020**, 65, 567–572.
[22] M. Jurášek, S. Rimpelová, E. Kmoníčková, P. Drašar, T. Ruml, *J. Med. Chem.* **2014**, 57, 7947–7954.
[23] F. Gutzeit, M. Dommaschk, N. Levin, A. Buchholz, E. Schaub, W. Plass, C. Näther, R. Herges, *Inorg. Chem.* **2019**, 58, 12542–12546.
